# Differential roles of FOXC2 in the trabecular meshwork and Schlemm’s canal in glaucomatous pathology

**DOI:** 10.26508/lsa.202201721

**Published:** 2023-07-06

**Authors:** Naoto Ujiie, Pieter R Norden, Raymond Fang, Lisa Beckmann, Zhen Cai, Junghun Kweon, Ting Liu, Can Tan, Megan S Kuhn, W Daniel Stamer, Kazushi Aoto, Susan E Quaggin, Hao F Zhang, Tsutomu Kume

**Affiliations:** 1 Feinberg Cardiovascular and Renal Research Institute, Department of Medicine, Feinberg School of Medicine, Northwestern University, Chicago, IL, USA; 2 Department of Biomedical Engineering, Northwestern University, Evanston, IL, USA; 3 Duke Eye Center, Duke University , Durham, NC, USA; 4 Department of Biochemistry, Hamamatsu University School of Medicine, Hamamatsu, Japan; 5 Division of Nephrology and Hypertension, Northwestern University Feinberg School of Medicine, Northwestern University, Chicago, IL, USA; 6 Department of Ophthalmology, Northwestern University, Chicago, IL, USA

## Abstract

Impaired development and maintenance of Schlemm’s canal (SC) are associated with perturbed aqueous humor outflow and intraocular pressure. The angiopoietin (ANGPT)/TIE2 signaling pathway regulates SC development and maintenance, whereas the molecular mechanisms of crosstalk between SC and the neural crest (NC)-derived neighboring tissue, the trabecular meshwork (TM), are poorly understood. Here, we show NC-specific forkhead box (*Fox)c2* deletion in mice results in impaired SC morphogenesis, loss of SC identity, and elevated intraocular pressure. Visible-light optical coherence tomography analysis further demonstrated functional impairment of the SC in response to changes in intraocular pressure in NC-*Foxc2*^*-/-*^ mice, suggesting altered TM biomechanics. Single-cell RNA-sequencing analysis identified that this phenotype is predominately characterized by transcriptional changes associated with extracellular matrix organization and stiffness in TM cell clusters, including increased matrix metalloproteinase expression, which can cleave the TIE2 ectodomain to produce soluble TIE2. Moreover, endothelial-specific *Foxc2* deletion impaired SC morphogenesis because of reduced TIE2 expression, which was rescued by deleting the TIE2 phosphatase VE-PTP. Thus, Foxc2 is critical in maintaining SC identity and morphogenesis via TM–SC crosstalk.

## Abstract (precis)

FOXC2 is required in the Schlemm’s canal endothelium and neural crest-derived trabecular meshwork cells for the morphogenesis and maintenance of Schlemm’s canal.

## Introduction

Glaucoma is the second leading cause of visual impairment, affecting 3.6 million adults aged 50 or older among the 33.6 million recorded cases of visual impairment in 2020 (1, 2), and it is estimated that ∼76 million people suffer from glaucoma globally as of 2020 (3). Primary congenital glaucoma (PCG) is characterized as developmental glaucoma occurring before 3 yr of age because of obstructed drainage of aqueous humor via the conventional outflow pathway without overt structural defects of the eye. In contrast, developmental glaucoma occurs secondarily to observed malformations of the anterior segment of the eye (iridocorneal angle, ciliary muscle, etc.) (4). Aqueous humor nourishes the tissues of the anterior segment and maintains pressure and the proper shape of the eye. The ciliary body secretes it, circulates into the anterior chamber, and returns to the circulation in part by the conventional outflow pathway consisting of flow through the trabecular meshwork (TM), which is derived from a neural crest (NC) lineage, and Schlemm’s canal (SC) (5). Intraocular pressure (IOP) naturally results from outflow resistance generation within the conventional outflow pathway, and elevated IOP is recognized as a critical risk factor contributing to optic neuropathy and the pathophysiology of glaucoma, including PCG and developmental glaucomas (4, 6, 7). because elevated IOP is the primary and only modifiable risk factor for glaucoma, current treatments focus on lowering IOP by topical drugs, lasers or surgical intervention.

Axenfeld-Rieger (AR) malformations refer to autosomal dominant developmental abnormalities of the anterior eye segment associated with mutations in the transcription factors paired-like homeodomain transcription factor 2 (*PITX2*) and forkhead box (*FOX*)*C1* and often result in the progression of glaucomatous blindness (8). *FOXC2* mutations are predominately associated with lymphatic vascular dysfunction and the progression of the autosomal dominant lymphedema-distichiasis syndrome (9, 10, 11). Of note, genetic evidence shows that FOXC2 variants are associated with functional alterations as modifier factors in congenital glaucoma (12, 13). During development, both *Foxc1* and *Foxc2* share overlapping expression patterns and function cooperatively and complementary to one another in various aspects of tissue development, including blood and lymphatic vascular growth and maintenance (14, 15, 16). Notably, our group reported generating and analyzing neural crest (NC)-specific *Foxc2* mutant mice (17, 18). It demonstrated that NC-derived periocular mesenchymal cells require the expression of *Foxc2* for proper TM formation (18). However, its role in SC’s development, maintenance, and function has yet to be thoroughly investigated. Because NC-specific *Foxc2* mutant mice (NC-*Foxc2*^*-/-*^) are viable and survive into adulthood (17, 18), this mutant mouse line is a valuable animal model for elucidating the molecular and cellular mechanisms underlying SC formation and function via TM–SC crosstalk.

The SC is an extensive, hybrid vasculature with features characteristic of both lymphatic and venous vasculature (19, 20, 21, 22). In contrast to the limbal and conjunctival lymphatics originating from emergent lymphatic vessels on the nasal side of the developing eye, SC morphogenesis is initiated from the blood limbal and radial vascular plexuses during postnatal development (20, 23). The SC endothelium is known to express FOXC2 (19, 21), whereas several key lymphatic vascular signaling pathways directly regulate SC morphogenesis and maintenance, such as VEGF-C/VEGF receptor (VEGFR)-3 (19), PROX1 (21), and angiopoietin (ANGPT)/TIE2 (22, 24, 25, 26, 27). Of clinical importance, *TIE2* mutations have been previously identified in a subset of patients with PCG (24). Recently, TIE2 has emerged as a popular target for therapeutic intervention in PCG as TIE2 activation, and the use of small-molecule inhibitors of negative TIE2 regulation have shown beneficial effects of improved SC morphology, increased outflow facility, and reduction of IOP in animal models of glaucoma (28, 29). Here, we report that Foxc2 is required in both NC-derived TM cells and the SC endothelium for SC formation and maintenance. For the first time, to the best of our knowledge, we also demonstrated morphological and functional impairments of the SC in a transgenic mouse mutant (i.e., NC-*Foxc2*^*-/-*^) model using visible light optical coherence tomography (vis-OCT). Immunohistochemical analysis of SC morphology in NC-*Foxc2*^*-/-*^ mice further revealed that these mice develop hypoplastic SC vasculature during morphogenesis with reduced expression of key lymphatic markers such as VEGFR-3, PROX1, and TIE2. Single-cell RNA-sequencing (scRNA-seq) analysis of the anterior eye segment identified that transcriptional changes in TM cell cluster populations associated with NC-specific deletion of *Foxc2* were characterized by reduction of pro-angiogenic factors and increased expression of ECM remodeling genes, including matrix metalloproteinases (MMPs). We then show that chemical inhibition of MMP activity in cultured human lymphatic endothelial cells (LECs) impaired cleavage of the ectodomain of TIE2 expressed on LECs, thereby reducing the production of soluble TIE2 (sTIE2), which is capable of binding angiopoietins and preventing them from activating TIE2 (30), suggesting a potential regulatory mechanism of TIE2 signaling activity in the SC vasculature. On the other hand, early postnatal endothelial-specific deletion of *Foxc2* resulted in hypoplastic SC vasculature with reduced TIE2 expression, whereas conditional endothelial-specific deletion of one allele of *Ptprb*, encoding vascular endothelial protein tyrosine phosphatase (VE-PTP) that specifically dephosphorylates and deactivates TIE2, was able to rescue this phenotype. Finally, using a conditional knock-in mouse line, we show that *Foxc2* can functionally substitute for *Foxc1* in properly developing the anterior segment and SC vasculature. Collectively, our data demonstrate that Foxc2 is required in the SC endothelium and NC-derived TM cells for the morphogenesis and maintenance of the SC through cell-autonomous and cell-non-autonomous mechanisms, respectively.

## Results

### *Foxc2*^+^ cell descendants are observed in the trabecular meshwork and Schlemm’s canal vasculature

**Figure S1.**
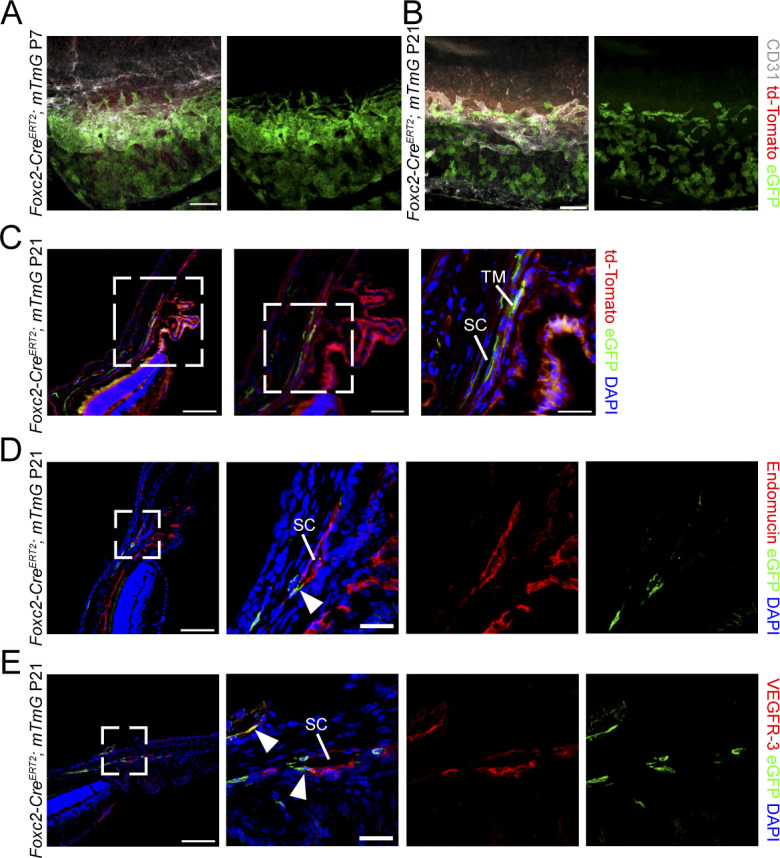
Fate-mapping of *Foxc2* expression in cells of the trabecular meshwork and SC vasculature. **(A, B)** Representative image of the SC vasculature from P7 (A) and P21 (B) *Foxc2-Cre*^*ERT2*^*; mTmG* mice treated with tamoxifen from postnatal day (P1) to P5 demonstrating Foxc2-GFP expression in the cells of the TM proximal to the SC and the SC vasculature denoted by CD31 immunostaining. Scale bar is 100 μm. **(C)** Representative cross-sectional images of eGFP-expressing, *Foxc2-Cre*-mediated recombined cells in the SC and TM of a P21 *Foxc2-Cre*^*ERT2*^*; mTmG* mouse. Boxed areas denote magnified regions in sequential panels. TM, trabecular meshwork; SC, Schlemm’s canal. Pink, dashed line denotes the SC vessel. Scale bars are 100, 50, and 25 μm, respectively. **(D, E)** Representative cross-sectional images of eGFP-expressing, *Foxc2-Cre*-mediated recombined cells in the SC of a P21 *Foxc2-Cre*^*ERT2*^*; mTmG* mouse immunostained with endomucin (D) or VEGFR-3 (E). Boxed areas denote magnified regions in the next panels. SC, Schlemm’s canal. Arrowheads denote GFP-positive and endomucin- or VEGFR-3- positive cells. Scale bars are 100 and 25 μm, respectively.

Aqueous humor drainage from the anterior segment of the eye into the systemic circulation is mediated through the TM, which is derived from the NC lineage (20, 31). There is evidence that Foxc2 is expressed in NC-derived periocular mesenchymal cells in the anterior eye segment (31) and the SC endothelium (19, 21). Furthermore, lineage-tracing analysis using a tamoxifen-inducible *Foxc2*^*CreERT2*^*; R26R* reporter mouse line previously identified that descendants of mesenchymal *Foxc2*-expressing cells undergo division and proliferation to generate cells within the periocular and corneal mesenchymes during embryonic ocular development (32). Thus, we sought to investigate how descendants of *Foxc2*-expressing cells contribute to the development of tissues comprising the conventional outflow pathway, including the TM and SC vasculature, beginning at early postnatal development when SC morphogenesis is initiated. To accomplish this, we crossed *Foxc2*^*CreERT2*^ knock-in mice (32) with the *ROSA*^*mT/mG*^ reporter strain to generate a tamoxifen-inducible *Foxc2*^*CreERT2*^*; mTmG* strain and performed tamoxifen administration daily from postnatal day (P)1 to P5 during early SC morphogenesis, which occurs from P1 to ∼P15–P17 where the SC vasculature has reached its mature morphology (20). Immunostaining analysis of CD31 expression in the limbal region of flat-mounted tissue from *Foxc2*^*CreERT2*^*; mTmG* mice showed *Foxc2-Cre* mediated, eGFP-positive expression (indicative of *Foxc2*^*+*^ cell descendants) in the CD31^+^ SC vasculature and adjacent TM at both P7 and P21 (Fig S1A and B). Similarly, cryosection immunostaining analysis of the iridocorneal angle from *Foxc2*^*CreERT2*^*; mTmG* mice identified *Foxc2-Cre* mediated, eGFP-positive expression in the TM (Fig S1C), and the SC endothelium where select eGFP-positive cells also exhibited positive endomucin and VEGFR-3 expression (Fig S1D and E). Although postnatal *Foxc2*^*CreERT2*^-mediated recombination in the anterior segment of the eye appears less effective compared with the previous lineage analyses with *Foxc2*^*CreERT2*^ mice during embryonic eye development (32), these lineage-tracing observations indicate that *Foxc2*^*+*^ cell descendants contribute to the SC and TM components during postnatal development (33, 34).

### Neural crest-specific deletion of *Foxc2* impairs TM development and indirectly impairs Schlemm’s canal morphology

**Figure S2.**
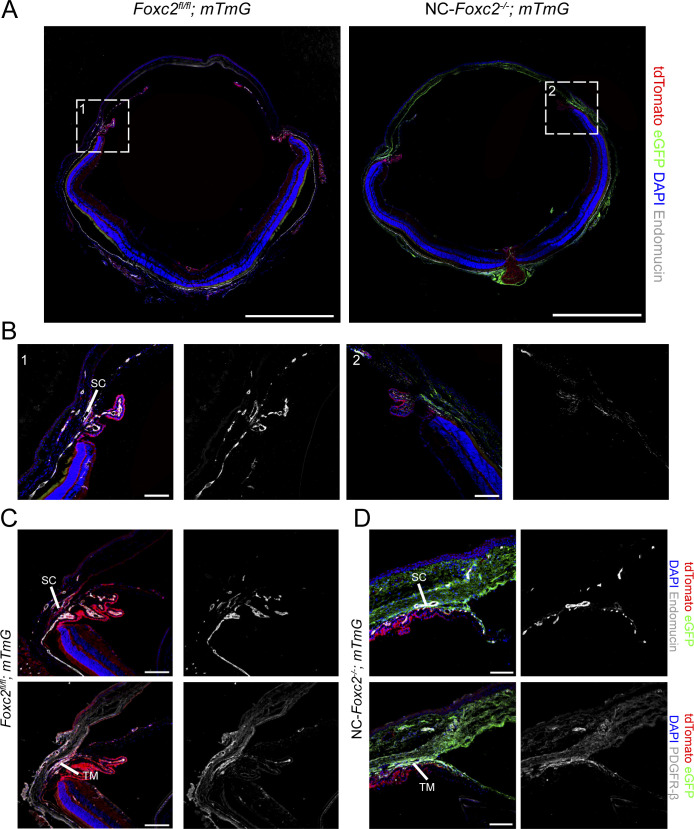
Fate-mapping of neural crest-derived cell populations in NC-*Foxc2*^*-/-*^ mice. **(A)** Representative images of eye sections from 3-wk-old control *Foxc2*^*fl/fl*^*; mTmG* and NC-*Foxc2*^*-/-*^; mTmG mice immunostained with antibody against endomucin where eGFP expression is regulated by *Wnt1-Cre*-mediated recombination. Scale bars are 1 mm. **(A, B)** Magnified images of boxed regions denoted in (A). Scale bars are 100 μm. **(C, D)** Representative images of sections of the iridocorneal angle from a 3-wk-old NC-*Foxc2*^*-/-*^*; mTmG* individual with a severe phenotype (D) compared with a control *Foxc2fl/fl*; *mTmG* individual immunostained with antibody against endomucin or PDGFR-β. Scale bars are 100 μm.

Our group previously reported that NC-*Foxc2*^*-/-*^ mice (*Wnt1-Cre; Foxc2*^*fl/fl*^) were characterized by ocular abnormalities in the anterior segment, including hypoplasia of the TM (18). To assess the direct contribution of NC-derived cell populations more carefully to the abnormal development of the conventional outflow pathway in NC-*Foxc2*^*-/-*^ mice, we performed lineage-tracing analysis by generating NC-*Foxc2*^*-/-*^*; mTmG* mice (*Wnt1-Cre; Foxc2*^*fl/fl*^*; ROSA*^*mT/mG*^). Cryosection immunostaining analysis of 3-wk-old NC-*Foxc2*^*-/-*^*; mTmG* mice showed similar abnormal defects in anterior segment tissues as to what our group reported with eGFP-positive expression observed in the corneal stroma, TM, ciliary processes, and scleral tissue of NC-*Foxc2*^*-/-*^*; mTmG* mice compared with Cre-negative *Foxc2*^*fl/fl*^*; mTmG* controls (Fig S2A and B). Notably, analysis of sections showed regions of the iridocorneal angle where the SC appeared to be nearly absent or characterized by abnormal morphology with reduced area and a nearly closed vessel in NC-*Foxc2*^*-/-*^*; mTmG* mice compared with *Foxc2*^*fl/fl*^*; mTmG* controls (Fig S2C and D). As characterized in previously published reports (20), eGFP-positive expression was not observed in the SC endothelium after recombination mediated by the *Wnt1-Cre* driver. In contrast, eGFP expression was observed in hypoplastic, PDGFRβ-positive TM (21) (Fig S2C and D). Thus, these results indicate that loss of *Foxc2* expression and transcriptional activity in NC-derived TM cells indirectly impairs SC morphogenesis during early postnatal development and maturation.

### NC-*Foxc2*^*-/-*^ mice exhibit corneal neovascularization, reduced SC size, and normal relationship between SC and IOP

**Figure 1.**
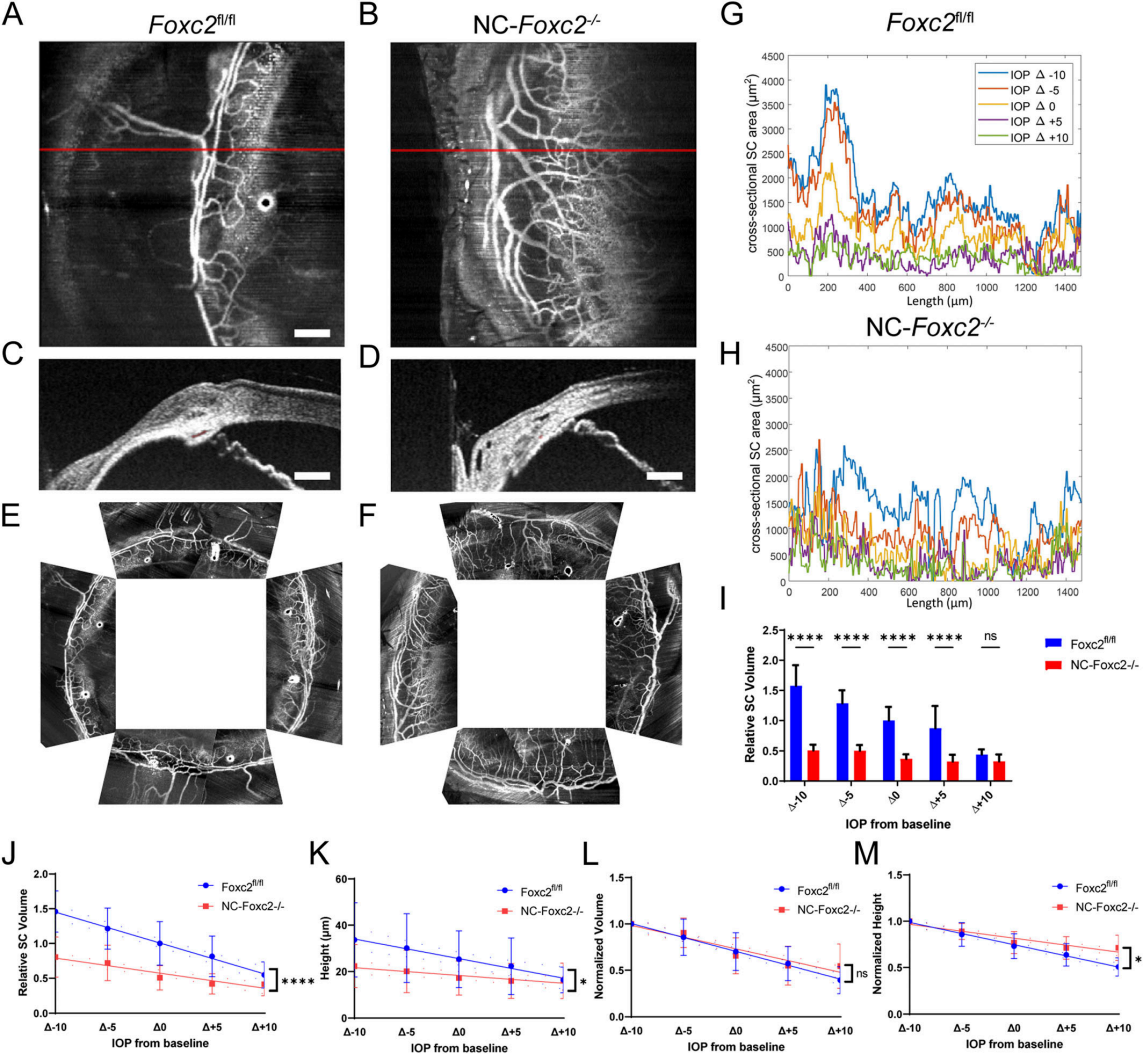
Vis-OCTA and vis-OCT identify corneal neovascularization and reduced SC area and volume in NC-*Foxc2*^-/-^ mice. **(A, B)** Representative angiograms of an individual vis-OCTA raster scan from adult, 7-mo-old *Foxc2*^*fl/fl*^ control (A) and NC-*Foxc2*^*-/-*^ (B) mice. **(C, D)** Representative cross-sectional vis-OCT B-scan images from *Foxc2*^*fl/fl*^ control (C) and NC-*Foxc2*^*-/-*^ (D) mice. Red shaded area denotes the SC area. **(E, F)** Representative compound circumlimbal scans composed of eight separate raster angiograms each from *Foxc2*^*fl/fl*^ control (E) and NC-*Foxc2*^*-/-*^ (F) mice. Scale bar: 200 μm. **(G)** Representative plot of SC area versus length from one adult *Foxc2*^*fl/fl*^ control mouse at IOP levels ranging from 10 mmHg below baseline IOP to 10 mmHg above baseline IOP. SC area is measured from segmented vis-OCT B-scan images. **(H)** Representative plots of the SC area versus length from one adult NC-*Foxc2*^*-/-*^ mice at IOP levels ranging from 10 mmHg below baseline IOP to 10 mmHg above baseline IOP. **(I)** Relative SC volume plotted against change in IOP, where the cycle of IOP changes was repeated three times in the same mouse to give a mean and standard error for N = 3 Control and N = 4 NC-*Foxc2*^*-/-*^ mice. SC volume for each IOP and group was normalized by the mean *Foxc2*^*fl/fl*^ control SC volume at baseline IOP. **(J, K)** Linear fit of relative SC volume and height as a function of IOP with 95% confidence interval of fits given by dotted lines for N = 6 Control and N = 6 NC-*Foxc2*^*-/-*^ mice where each IOP level was repeated three times. **(L, M)** Linear fit of normalized volume and SC height relative to mean height at 10 mmHg below baseline IOP for each eye with 95% confidence error of fits given for N = 6 Control and N = 6 NC-*Foxc2*^*-/-*^ mice where each IOP level was repeated three times. Statistical analysis: two-way ANOVA with Šídák’s multiple comparisons test. *P < 0.05, **P < 0.01, ***, *P* < 0.001, ****P < 0.0001. Source data are available for this figure.

Our group has previously used corneal flat-mount immunostaining and OCT imaging to show that NC-*Foxc2*^*-/-*^ mice exhibit corneal neovascularization (18). However, the characteristics of SC morphology in these individuals are unknown. To carefully assess possible phenotypes in SC morphology in these individuals, we used vis-OCT to acquire compound circumlimbal scans to visualize the SC in live mice (35). We used this system to acquire vis-OCT angiography (vis-OCTA) volumes of the limbal region of both 6–8-wk-old *Foxc2*^*fl/fl*^ control and NC-*Foxc2*^*-/-*^ mice with *mild* ocular phenotypes, as mice with severe ocular phenotypes could not be accurately measured with vis-OCTA and often lacked SC. Representative *en-face* projections of the volumes show modest corneal neovascularization in NC-*Foxc2*^*-/-*^ mice as previously reported (18) (Fig 1A, B, E, and F). Representative B-scan images from each location marked in red on the *en-face* vis-OCTA images in Fig 1A and B are shown in Fig 1C and D, respectively. Here, the SC is clearly visible in the cross-sectional B-scan images showing the reduced area in NC-*Foxc2*^*-/-*^ mice with *mild* ocular phenotypes. For these mild phenotypes, the cross-sectional B-scan images showed no gross abnormalities in the corneal structure. Indeed, the peripheral corneal thickness was not statistically different between NC-*Foxc2*^*-/-*^ mice (100 ± 26 μm) and *Foxc2*^*fl/fl*^ mice (102 ± 20 μm).

To investigate the in vivo functional behavior of the SC in NC-*Foxc2*^*-/-*^ mice, we assessed changes in SC morphology in response to changes from baseline IOP under deep anesthesia (35). Cross-sectional area versus length for 5 IOP levels relative to the measured baseline IOP, at −10 mmHg, −5 mmHg, 0 mmHg, +5 mmHg, and +10 mmHg, was quantified for both *Foxc2*^*fl/fl*^ control and NC-*Foxc2*^*-/-*^ mice (Fig 1G and H). In both, there is an inverse relationship between IOP and SC cross-sectional area (36). In addition, in both, conserved regions of the higher cross-sectional area across all IOP levels can be observed. Overall, the SC volume is smaller in NC-*Foxc2*^*-/-*^ mice at IOP levels of Δ−10 mmHg, Δ−5 mmHg, Δ0 mmHg, and Δ+5 mmHg (Fig 1I). A linear fit of relative SC volume, the volume normalized to the mean SC volume in *Foxc2*^fl/fl^ mice, as a function of IOP had a slope of −0.044 relative SC volume per mmHg in *Foxc2*^*fl/fl*^ and −0.022 relative SC volume per mmHg in NC-*Foxc2*^*-/-*^ with the slopes statistically different with *P* < 0.0001 (Fig 1J). Similarly, a linear fit of SC height as a function of IOP had a slope of −0.85 μm per mmHg in *Foxc2*^*fl/fl*^ and −0.34 μm per mmHg in NC-*Foxc2*^*-/-*^ with the slopes statistically different with *P* = 0.025 (Fig 1K). Overall, the SC and TM deform less with changes in IOP, suggesting that the TM of NC-*Foxc2*^*-/-*^ mice experience less strain owing to natural variations in IOP. The slope of a linear fit of normalized volume as a function of IOP, the volume relative to the Δ−10 mmHg volume for each eye, was −0.025 per mmHg in NC-*Foxc2*^*-/-*^ and −0.03 per mmHg in *Foxc2*^*fl/fl*^ but not statistically significant (Fig 1L). Quantification of the slope of normalized height as a function of IOP, the height relative to the Δ−10 mmHg height for each eye, was −0.015 per mmHg in NC-*Foxc2*^*-/-*^ and −0.024 mmHg in Foxc2^*fl/fl*^ with the slopes statistically different with *P* = 0.0002 (Fig 1M). Collectively, these data demonstrate that NC-specific loss of *Foxc2* results in abnormal SC morphology and impaired functional sensing of IOP changes.

### The SC endothelium of NC-*Foxc2*^*-/-*^ mice is characterized by abnormal morphology and failure to properly establish SC endothelial identity

**Figure 2.**
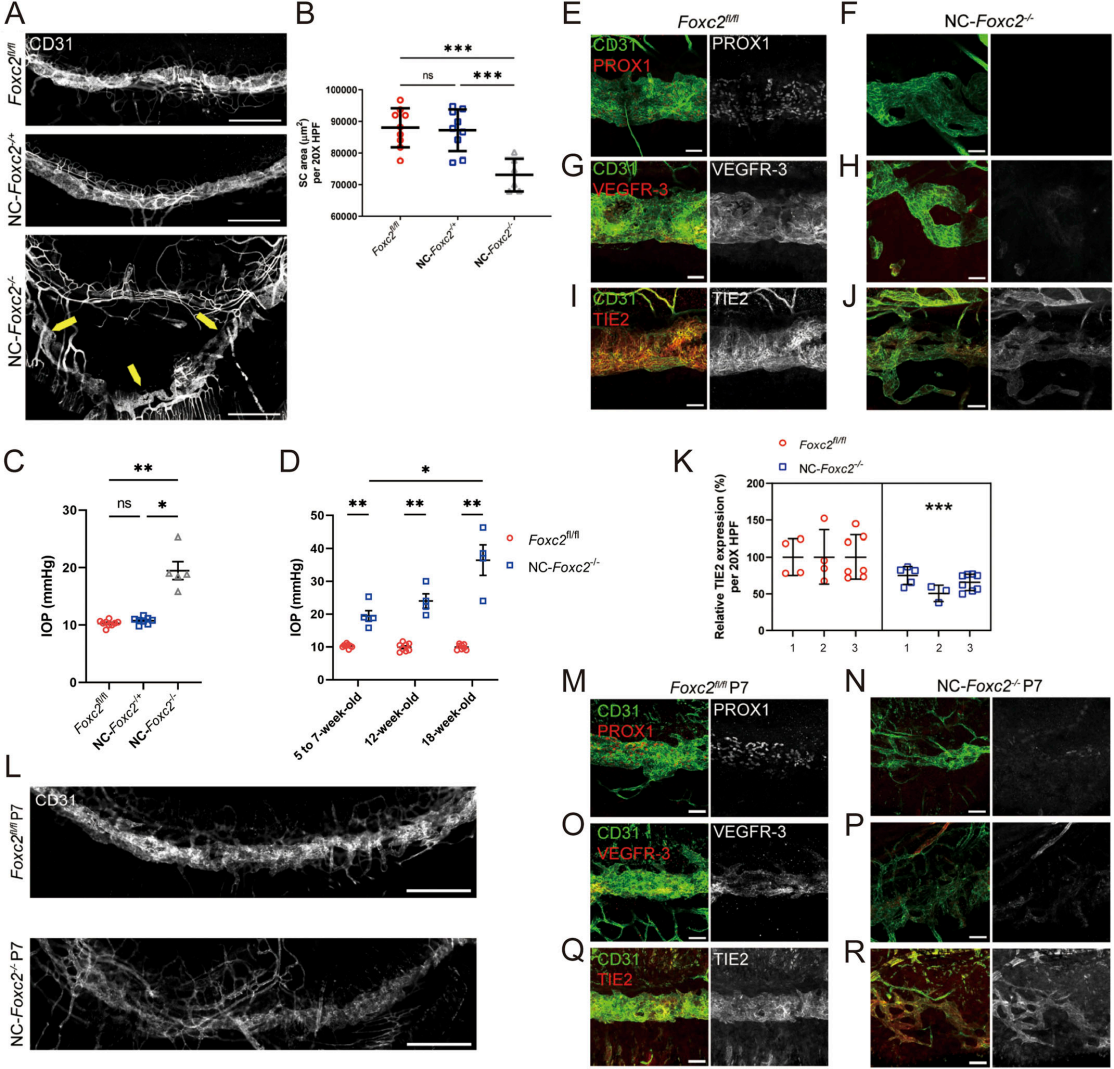
Neural crest-derived *Foxc2* is required for proper morphogenesis of the SC and establishment of SC identity. **(A)** Representative images of SC vasculature immunostained with CD31 antibody in adult, *Foxc2*^*fl/fl*^ control, NC-*Foxc2*^*-/+*^, and NC-*Foxc2*^*-/-*^ mice. Yellow arrows highlight abnormal SC morphology and displacement in a NC-*Foxc2*^*-/-*^ individual. Scale bars are 500 μm. **(B)** Quantification of SC area per 20X high-power field (HPF). N = 9 for *Foxc2*^*fl/fl*^ controls. N = 9 for NC-*Foxc2*^*-/+*^. N = 6 for NC-*Foxc2*^*-/-*^. Data are mean ± SD. Statistical analysis: Mann–Whitney *U* test. ***P < 0.001. **(C)** Quantification of IOP in 5–7-wk-old mice measured by a rebound tonometer. N = 9 for *Foxc2*^*fl/fl*^ controls. N = 8 for NC-*Foxc2*^*-/+*^. N = 5 for NC-*Foxc2*^*-/-*^. Data are mean ± SD. Statistical analysis: Mann-Whitney *U* test. *P < 0.05. **P < 0.01. **(D)** Quantification of IOP in 5–7-wk-old, 12-wk-old, and 18-wk-old mice measured by a rebound tonometer. Data are mean ± SD. Statistical analysis: Mann–Whitney *U* test. *P < 0.05. **P < 0.01. **(E, F, G, H, I, J)** Representative images of CD31 and PROX1 (E, F), VEGFR-3 (G, H) or Tie2 (I, J) expression in the SC of adult *Foxc2*^*fl/fl*^ Control (E, G, I) and NC-*Foxc2*^*-/-*^ mice (F, H, J). Scale bars are 50 μm. **(K)** Quantification of the relative expression of Tie2 in the SC of *Foxc2*^*fl/fl*^ Control and NC-*Foxc2*^*-/-*^ mice per 20X high-power field. N = 3 for *Foxc2*^*fl/fl*^ controls and N = 3 for NC-*Foxc2*^*-/-*^. Symbols depict technical replicates per individual. Data are mean ± SD. Statistical analysis: nested unpaired *t* test. ***P < 0.001. **(L)** Representative images of SC vasculature immunostained with CD31 antibody show abnormal morphology and reduced area in P7 NC-*Foxc2*^*-/-*^ mice compared with P7 *Foxc2*^*fl/fl*^ Control mice. Scale bars are 500 μm. **(M, N, O, P, Q, R)** Representative images of CD31 and PROX1 (M, N), VEGFR-3 (O, P), or Tie2 (Q, R) expression in the SC of P7 *Foxc2*^*fl/fl*^ Control (M, O, Q) and NC-*Foxc2*^*-/-*^ mice (N, P, R). Scale bars are 50 μm. Source data are available for this figure.

**Figure S3.**
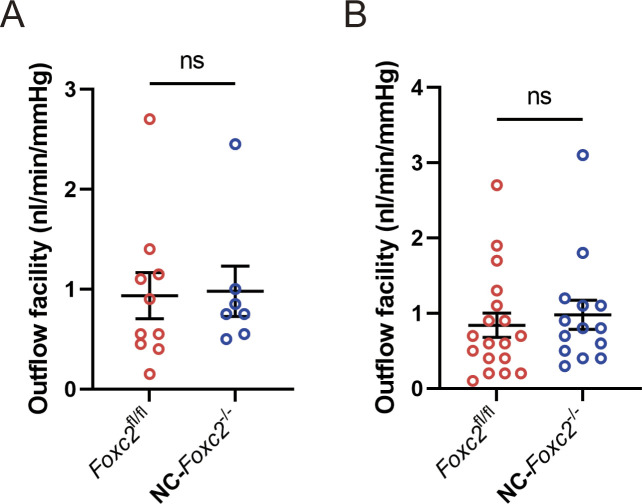
Quantification of outflow facility. **(A, B)** Quantification of outflow facility for individual mice (A) and each eye (B) measured by an iPerfusion system in adult 9–11-wk-old *Foxc2*^*fl/fl*^ control and NC-*Foxc2*^*-/-*^ mice. **(A)** Data for individual mice were calculated as the average value of the right and left eyes. N = 10 for *Foxc2*^*fl/fl*^ controls. N = 7 for NC-*Foxc2*^*-/-*^. **(B)** Data for each eye. N = 18 eyes for *Foxc2*^*fl/fl*^ controls. N = 14 eyes for NC-*Foxc2*^*-/-*^. Data are mean ± SD. Statistical analysis: Mann–Whitney *U* test. Source data are available for this figure.

We additionally characterized morphological and phenotypic changes in the SC endothelium associated with NC-specific loss of *Foxc2* in adult mice. We obtained several ocular phenotypes in NC-*Foxc2*^*-/-*^ mice: (1) mild, eyes with dull light reflection; (2) moderate, opaque eyes; (3) severe, opaque eyes without SC. Flat mount immunostaining analysis of CD31 expression within the limbal region of 6–8-wk-old *Foxc2*^*fl/fl*^, NC-*Foxc2*^*-/+*^ (*Wnt1-Cre; Foxc2*^*fl/+*^), and NC-*Foxc2*^*-/-*^ mice demonstrated that retainment of one allelic copy of *Foxc2* is sufficient to maintain proper SC morphogenesis; however, the total loss of NC-specific *Foxc2* expression in mice (i.e., NC-*Foxc2*^-/-^ mice, but not NC-*Foxc2*^+/-^ mice) results in a hypoplastic SC vasculature, typically displaced from the limbal blood and lymphatic vascular plexus (Fig 2A). Because of a mixed genetic background, the penetrance of the severity of the SC phenotype was variable in NC-*Foxc2*^*-/-*^ mice with a subset of mice characterized by the absence of SC and severe corneal neovascularization (10/26, 38.5%). Quantification of the CD31-immunostained SC area was significantly reduced in NC-*Foxc2*^*-/-*^ mice with *mild* or *moderate* ocular phenotypes (by excluding those with the absence of SC) compared with *Foxc2*^*fl/fl*^ controls. In contrast, no differences were observed between control and NC-*Foxc2*^*-/+*^ mice (Fig 2B). We next measured IOP in adult (5–7-wk-old) control (*Foxc2*^*fl/fl*^), NC-*Foxc2*^*-/+*^, and NC-*Foxc2*^*-/-*^ with *mild* or *moderate* ocular phenotypes and observed increased IOP in NC-*Foxc2*^*-/-*^ mice, indicative of increased outflow resistance, whereas there were no differences between control and NC-*Foxc2*^*-/+*^ mice (Fig 2C). IOP in the different time points was also examined in 5–7-wk-old, 12-wk-old, and 18-wk-old control and NC-*Foxc2*^*-/-*^ mice, showing increased IOP in NC-*Foxc2*^*-/-*^ mice at any age; moreover, IOP was significantly higher in the 18-wk-old NC-*Foxc2*^*-/-*^ mice compared with the 5–7-wk-old NC-*Foxc2*^*-/-*^ mice. (Fig 2D). We also evaluated the outflow facility in adult control and NC-*Foxc2*^*-/-*^ mice (9–11-wk-old) with *mild* ocular phenotypes (excluding other than *mild* ocular phenotype because of technical difficulty), indicating no differences were observed (Fig S3A and B).

As SC morphogenesis was impaired in NC-*Foxc2*^*-/-*^ mice, we sought to characterize the expression of key molecular regulators of SC morphogenesis and maintenance (Fig 2E–J). PROX1 and VEGFR-3 expressions were nearly absent in the hypoplastic SC vasculature of adult NC-*Foxc2*^*-/-*^ mice (Fig 2F and H), whereas TIE2 expression was modestly, but significantly, reduced (Fig 2J and K) implying that the SC vasculature in adult NC-*Foxc2*^*-/-*^ mice fails to properly establish or maintain SC endothelial identity. To address this discrepancy, we characterized SC vasculature in NC-*Foxc2*^*-/-*^ mice and *Foxc2*^*fl/fl*^ controls at P7 during the mid-stage of SC morphogenesis (Fig 2L–R). Like adult mice, the SC vasculature was absent in neonates compared with *Foxc2*^*fl/fl*^ controls (Fig 2L), PROX1 and VEGFR-3 expressions were severely decreased (Fig 2N and P), and TIE2 expression was modestly reduced in neonatal NC-*Foxc2*^*-/-*^ mice (Fig 2R). Thus, early morphogenesis of the SC, and establishment and maintenance of SC endothelial identity, is impaired in NC-*Foxc2*^*-/-*^ mice.

### Single-cell transcriptome analysis identifies molecular signaling pathway alterations in TM populations of NC-*Foxc2*^*-/-*^ mice

**Figure 3.**
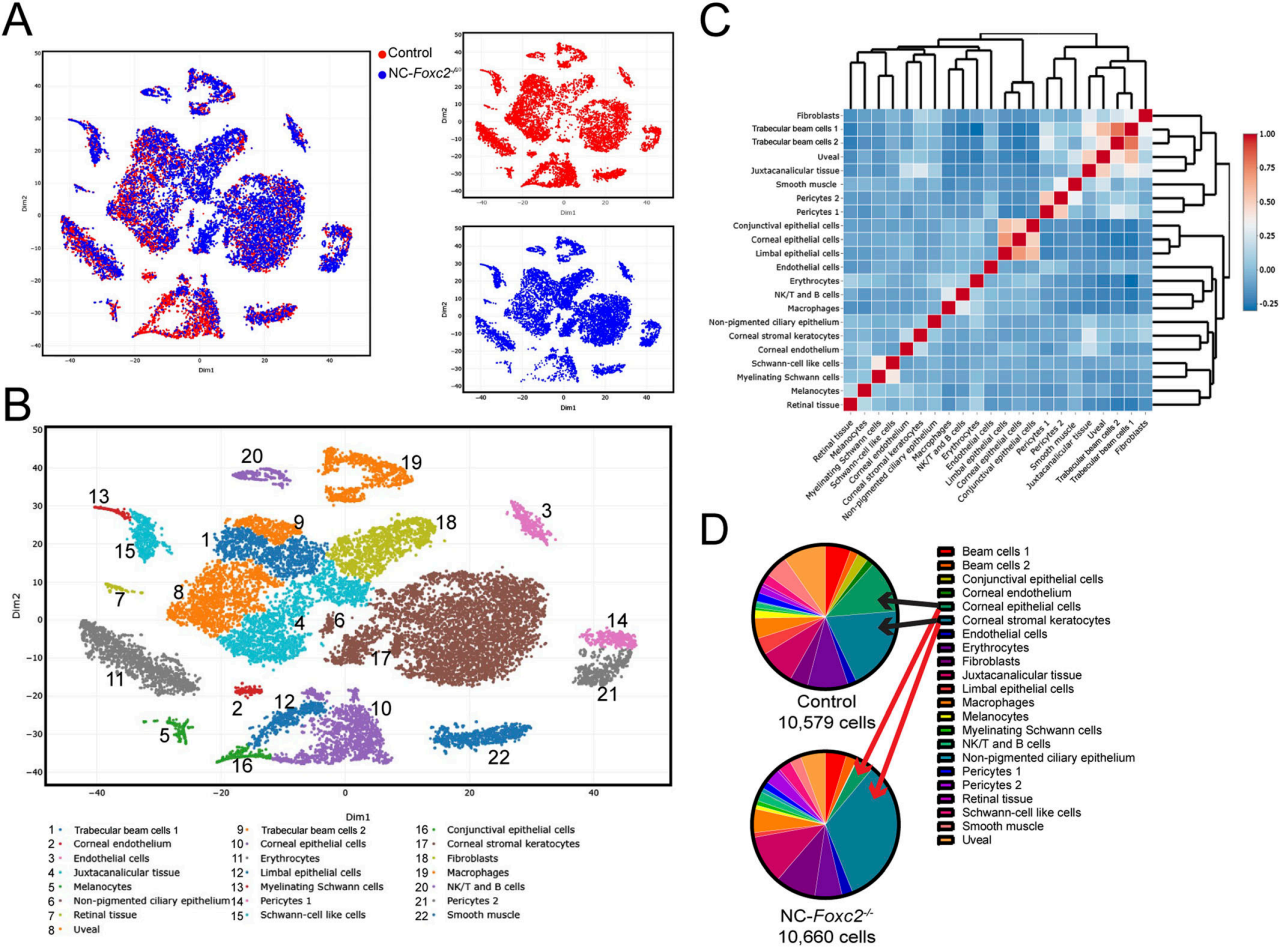
Single-cell transcriptome profiling of cell populations in the anterior eye segment of Control and NC-*Foxc2*^*-/-*^ mice. **(A, B)** Visualization of individual cell contribution (A) and unsupervised clustering analysis of 22 transcriptionally distinct cell populations (B) by t-distributed stochastic neighbor embedding in the anterior eye segment of 3–4-wk-old *Foxc2*^*fl/fl*^ Control and NC-*Foxc2*^*-/-*^ mice. **(C)** Correlation heatmap and hierarchical clustering of gene-expression signatures of all cell populations. **(D)** Pie charts demonstrating the proportion of the total cell number analyzed contributing to each individual cell cluster for Control and NC-*Foxc2*^*-/-*^ mice. Source data are available for this figure.

**Figure S4.**
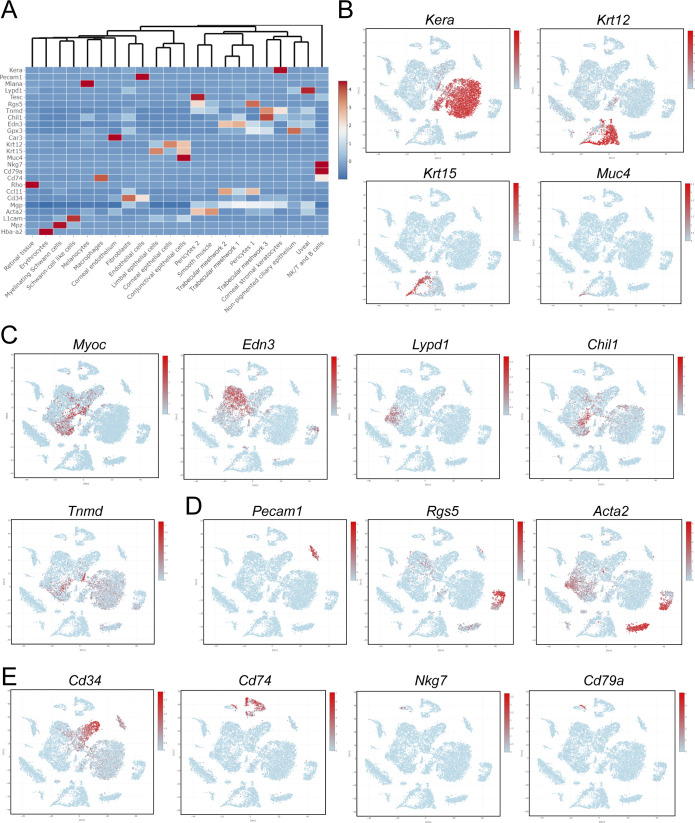
Characterization of cluster phenotypes. **(A)** Heatmap and hierarchical clustering of cell populations based on selective genes uniquely expressed in each cell population. **(B, C, D, E)** t-SNE plots colorized by the Seurat-normalized expression of *Kera*, *Krt12*, *Krt15*, and *Muc4* identifying corneal stromal keratocytes and epithelial cell populations (B), *Myoc*, *Edn3*, *Lypd1*, *Chil1*, and *Tnmd* identifying trabecular meshwork cell populations (C), *Pecam1*, *Rgs5*, and *Acta2* identifying endothelial, pericyte, and smooth muscle cell populations (D), or *Cd34*, *Cd74*, *Nkg7*, and *Cd79a* identifying fibroblast, macrophage, NK/T, and B cell populations (E).

To understand the contribution of NC-*Foxc2* transcriptional regulation of development of the conventional outflow pathway at the molecular level, we performed scRNA-seq analysis of pooled anterior eye segments from 3–4-wk-old *Foxc2*^*fl/fl*^ control mice and NC-*Foxc2*^*-/-*^ mice with *moderate* ocular phenotypes. Although the SC develops a largely mature appearance by P17 after its initial morphogenesis after birth, we found it impractical to confidentially dissect the anterior segment of the eye from neonatal mice for the scRNA-seq study. Thus, young mice just after weaning were used to optimize the total cell number used for single-cell sequencing, as recently performed at 6 wk of age by Thomson et al (37), and NC-*Foxc2*^*-/-*^ mice with *moderate* phenotypes were selected so that the anterior eye segment could be definitively dissected from the posterior segment. T-distributed stochastic neighbor embedding (t-SNE) visualization and clustering analysis of cells from both Foxc2^*fl/fl*^ control and NC-*Foxc2*^*-/-*^ mice identified 22 transcriptionally distinct cell populations including the uveal meshwork, in which aqueous humor first traverses through the TM, three transcriptionally distinct trabecular meshwork populations, and endothelial cells among other cell populations (Figs 3A–D and S4A–E) characterized from previously reported scRNA-seq analysis studies of the murine conventional outflow pathway and anterior eye segment (37, 38, 39, 40, 41).

**Figure S5.**
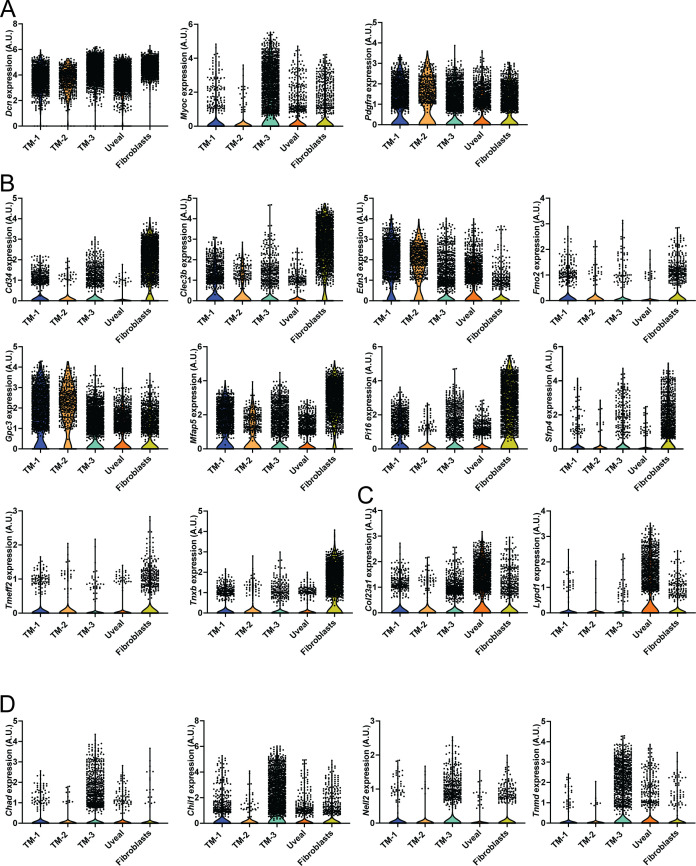
Characterization of trabecular meshwork clusters. **(A, B, C, D)** Violin plots showing the expression of *Dcn*, *Myoc*, and *Pdgfra* (A), *Cd34*, *Clec3b*, *Edn3*, *Fmo2*, *Gpc3*, *Mfap5*, *Pi16*, *Sfrp4*, *Tmeff2*, and *Tnxb* (B), *Col23a1,* and *Lypd1* (C), *Chad*, *Chil1*, *Nell2*, and *Tnmd* (D) from trabecular meshwork, uveal meshwork, and fibroblast clusters. Clusters are color coded as in Fig 3. Source data are available for this figure.

As NC-derived cells do not contribute to the development of the SC vasculature (20, 31), we focused our analyses on the assessment of the uvea meshwork and TM cell populations provided their anatomical proximity to SC. Previously reported single-cell analyses of the outflow pathway have identified uniquely expressed markers within different cell populations comprising the TM, such as fibroblast-like trabecular “beam” cells comprising the inner part of the posterior filtering region of the TM, the juxtacanalicular tissue (JCT) comprising the outer part of the posterior filtering region located adjacent to the SC, and the possible presence of *Cd34*-positive corneal stromal cells/fibroblasts clustering within TM cell populations as recently characterized by Thomson et al (37, 39, 40). Our initial analysis showed that known marker genes of TM, such as *Myoc* and *Chil1*, were predominately expressed in the trabecular meshwork 3 (TM-3) cluster, with less prominent expression in the trabecular meshwork 1 and 2 (TM-1 and TM-2) clusters (Figs S4C and S5A–D). However, TM-1 and TM-2 highly expressed *Dcn* and *Pdgfra* (Fig S5A), consistent with TM populations characterized in previously reported single-cell analyses (39, 40). TM-1 and TM-2 also contained high levels of *Edn3*, which may suggest that they are more closely related to the “Beam” cell populations previously characterized by van Zyl et al (40) (Fig S5B). In contrast, TM-3 showed higher expression of *Chad*, *Chil1*, *Nell2*, and *Tnmd* (Fig S5D), which were previously reported to be characteristics of the JCT. The uveal meshwork cluster also exhibited higher expression of *Col23a1* and *Lypd1* (Fig S5C), consistent with previous reports (37, 40). Our analysis also showed that our putative TM clusters expressed several fibroblast markers, such as *Cd34*, *Clec3b*, *Mfap5*, *Pi16*, and *Tnxb*, although their expression was particularly enriched in a separate cluster more characteristic of corneal stromal fibroblasts (Fig S5B).

**Figure 4.**
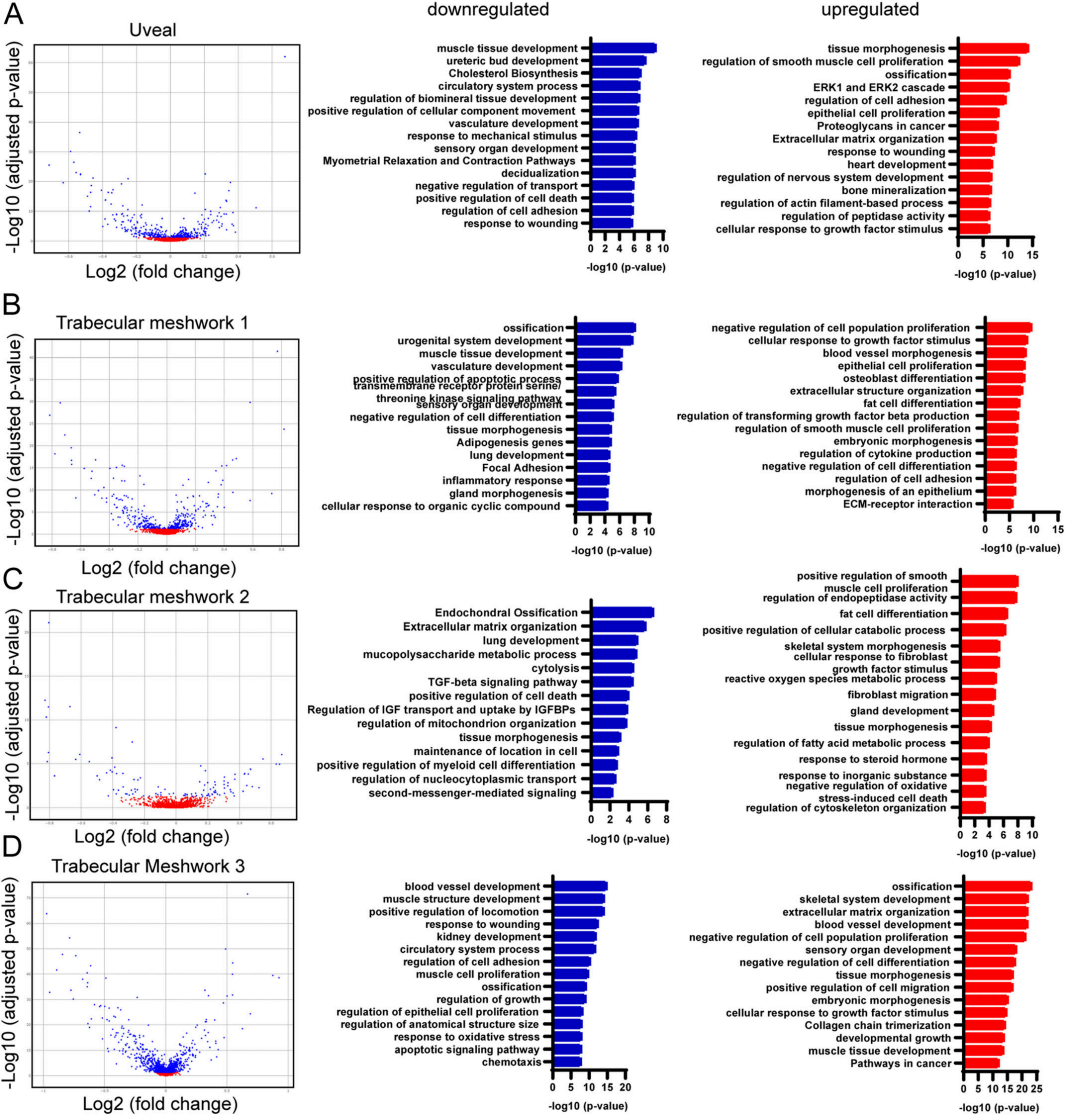
Differentially expressed genes in trabecular meshwork cell populations of NC-*Foxc2*^*-/-*^ mice compared with *Foxc2*^*fl/fl*^ Controls and GO over-representation analysis. **(A, B, C, D)** Volcano plots showing DEGs between NC-*Foxc2*^*-/-*^ and *Foxc2*^*fl/fl*^ Control mice in the trabecular meshwork 1 (A), trabecular meshwork 2 (B), trabecular meshwork 3 (C), and uveal meshwork clusters (D), left panels. Blue points denote DEGs with adjusted *P*-value < 0.05. Bar plots of subsets of GO gene sets that were overrepresented among the genes down-regulated (blue bars) or up-regulated (red bars) in NC-*Foxc2*^*-/-*^ mice compared with *Foxc2*^*fl/fl*^ Control mice, right panels. Values on the x-axis are represented as the –log_10_(*P*-value) of each associated GO gene set.

**Figure S6.**
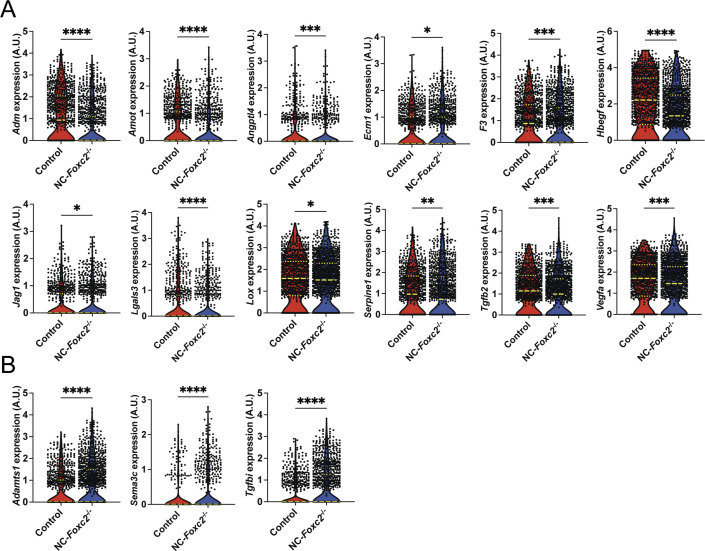
sc-RNA seq analysis identifies differentially expressed genes related to angiogenesis and ECM organization between Control and NC-*Foxc2*^*-/-*^ mice in cells comprising the trabecular meshwork 3 cluster. **(A, B)** Violin plots showing differential expression of *Adm*, *Amot*, *Angptl4*, *Ecm1*, *F3*, *Hbegf*, *Jag1*, *Lgals3*, *Lox*, *Serpine1*, *Tgfb2*, and *Vegfa* (A), *Adamts1*, *Sema3c*, and *Tgfbi* (B) between Control and NC-*Foxc2*^*-/-*^ mice. Long, dashed lines denote median values. Statistical analysis: Mann–Whitney Test. *P < 0.05, **P < 0.01, ***P < 0.001, ****P < 0.0001. Source data are available for this figure.

To investigate transcriptional changes in signaling pathways potentially contributing to impaired SC morphogenesis, we performed analysis of differentially expressed genes (DEGs) in uveal and TM cell populations and identified significant transcriptional changes within these populations (Fig 4A–D and Table S1) and, in particular, TM-3 (Fig 4D). Analysis of gene ontology (GO) biological processes from DEGs within these populations elucidated several meaningful biological changes associated with NC-specific loss of *Foxc2*. Down-regulated processes that were common among TM populations included blood vessel and vasculature development (Fig 4A, B, and D) and regulation of cell adhesion (Fig 4A and D). Similarly, up-regulated processes that were common among TM populations included blood vessel development (Fig 4D) and morphogenesis (Fig 4B), and regulation of cell adhesion (Fig 4A and B). However, GO terms associated with ECM function were most up-regulated among TM populations, including ECM organization, collagen chain trimerization, extracellular structure organization, and ECM–receptor interaction (Fig 4A, B, and D). The JCT is located adjacent to the inner wall endothelium of the SC (42). Its cells can extend processes that communicate with both the SC inner wall endothelium and TM cells in the corneoscleral meshwork (43). Although GO analysis identified that blood vessel development was associated with both down-regulated and up-regulated genes in TM-3 (characteristic of a putative JCT population), pro-angiogenic growth factors, and secreted proteins such as *Adm*, *Amot*, *Angptl4*, *Ecm1*, *F3*, *Hbegf*, *Jag1*, *Lgals3*, *Lox*, *Serpine1*, *Tgfb2*, and *Vegfa* were significantly down-regulated (Fig S6A and Table S1). whereas several anti-angiogenic signaling factors including *Adamts1*, *Sema3C*, and *Tgfbi* were significantly up-regulated (Fig S6B), possibly contributing to abnormal endothelial cell signaling and impaired SC morphogenesis.

Table S1 Differentially expressed genes (DEGs) in selected cell clusters from scRNA-seq analysis in Foxc2fl/fl and NC-Foxc2-/- mice.

**Figure 5.**
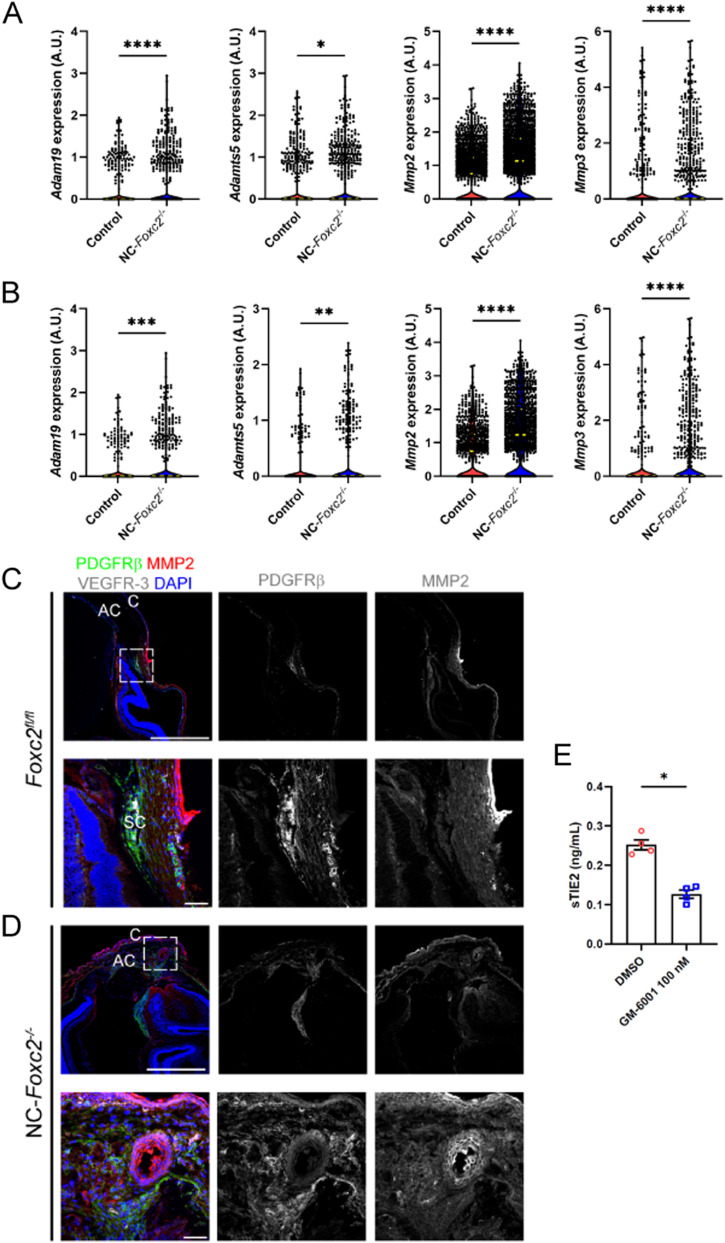
sc-RNA seq analysis of genes related to MMP. **(A, B)** sc-RNA seq analysis identifies differentially expressed genes related to MMPs between Control and NC-*Foxc2*^*-/-*^ mice in cells comprising the trabecular meshwork (A) and trabecular meshwork 3 (B) clusters. Violin plots showing differential expression of *Adam19*, *Adamts5*, *Mmp2*, and *Mmp3* in the TM cluster (A) and TM-3 cluster (B) between Control and NC-*Foxc2*^*-/-*^ mice. Long, dashed lines denote median values. Statistical analysis: Mann–Whitney Test. *P < 0.05, **P < 0.01, ***P < 0.001, ****P < 0.0001. **(C, D)** Representative cross-section images of the iridocorneal angle and anterior chamber of a *Foxc2*^*fl/fl*^ control (C) and NC-*Foxc2*^*-/-*^ individual (D) immunostained with PDGFRβ, MMP2, VEGFR-3, and DAPI. Boxed regions in top panels denote magnified regions in lower panels. Scale bars are 500 μm and 50 μm, respectively. C, cornea; AC, anterior chamber; SC, Schlemm’s canal. **(E)** Quantification of sTIE2 in conditioned media from cultured HDLECs treated with DMSO vehicle or GM-6001 at 100 nM by ELISA (E). Data are mean ± SD from three biological replicates. Statistical analysis: Mann–Whitney *U* test. *P < 0.05. Source data are available for this figure.

The TM primarily functions to regulate bulk aqueous humor outflow from the anterior chamber, which is accomplished by generating resistance to outflow (44). A previously reported work has demonstrated that forces generated by increased IOP likely act on the ECM and attached cells of the JCT and SC inner wall endothelium by stretching and distorting them, which in turn stimulates ECM processing and turnover initiated by MMP signaling in the TM (45, 46). Our scRNA-seq analysis revealed that ECM-related genes are significantly up-regulated in TM populations of NC-*Foxc2*^*-/-*^ mice, including several collagen genes and MMPs such as *Adam19*, *Adamts1*, *Adamts5*, *Mmp2*, and *Mmp3* in the TM cluster (Fig 5A) including TM-3 (Figs 5B and S5B), among other up-regulated ECM-related components (Table S1). In partial validation of our scRNA-seq observations, immunostaining of MMP-2 demonstrated its expression was increased throughout the anterior segment of NC-*Foxc2*^*-/-*^ mice compared with *Foxc2*^*fl/fl*^ controls (Fig 5C and D). Co-localization of increased MMP-2 expression with PDGFRβ+ TM cells and increased expression in the cornea was observed in NC-*Foxc2*^*-/-*^ mice with particularly *severe* anterior segment phenotypes, which lacked a mature SC as assessed by VEGFR-3 expression and anatomical features (Fig 5D), but it was weakly detected in PDGFRβ+ TM of *Foxc2*^*fl/fl*^ control mice (Fig 5C). Although our scRNA-seq analysis determined that increased *Mmp2* expression was limited to TM-3, it is possible that dosage-dependent effects of *Mmp2* expression among other genes may be attributable to the differences we observe in penetrance and severity of the phenotype observed in NC-*Foxc2*^*-/-*^ mice as mice with *moderate* ocular phenotypes were used for scRNA-seq analysis.

Of particular note, previously reported work has demonstrated that MMP activity mediates cleavage of the TIE2 ectodomain to produce soluble TIE2 (sTIE2) in cultured HUVECs (30, 47). Moreover, sTIE2 is capable of binding angiopoietins as a ligand trap to prevent them from activating TIE2 signaling (30). Provided the increased expression of MMPs in NC-*Foxc2*^*-/-*^ mice and the crucial role of ANGPT/TIE2 signaling in SC development (26, 27, 28, 37), we hypothesized that increased MMP activity in the TM may mediate cleavage of TIE2 in SC endothelium, resulting in higher production of sTIE2, impairment of ANGPT/TIE2 signaling, and impaired SC morphogenesis. To investigate this mechanism, we collected conditioned media from cultured human dermal LECs (HDLECs) treated with either the DMSO vehicle or the MMP inhibitor GM-6001 as shown to inhibit sTIE2 shedding in cultured HUVECs (30) or impair tubulogenesis in three-dimensional culture assays (48). Quantification of sTIE2 concentration in conditioned media from cultured HDLECs by ELISA demonstrated a significant reduction of sTIE2 by GM-6001 treatment compared with control (DMSO) treatment (Fig 5E). Thus, these results suggest that, like blood ECs, MMP activity also mediates TIE2 shedding in LECs, and the role of MMP signaling and potential cleavage of the TIE2 ectodomain within the SC warrants further investigation.

**Figure 6.**
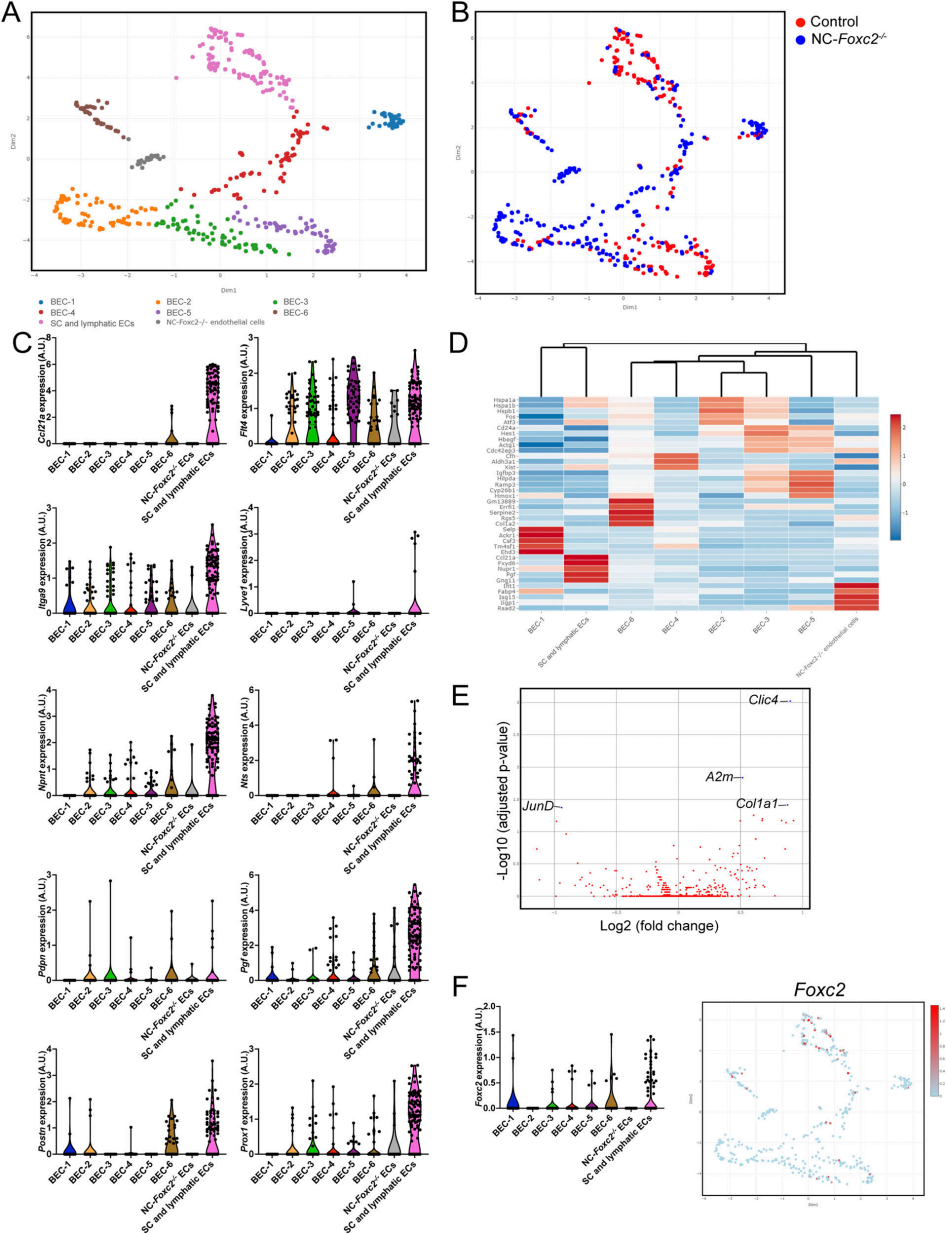
Single-cell transcriptome profiling of endothelium in the anterior eye segment of Control and NC-*Foxc2*^*-/-*^ mice. **(A, B)** Visualization of unsupervised subclustering analysis of eight transcriptionally distinct endothelial cell populations (A) and individual cell contribution (B) by Uniform Manifold Approximation and Projection of the endothelial cell cluster identified in Fig 4B. **(C)** Violin plots showing the expression of gene markers from the endothelial subclusters. **(D)** Feature heatmap and hierarchical clustering of gene-expression levels of the top five marker genes for each endothelial subcluster. Colors represent row-wise scaled gene expression with a mean of 0 and SD of 1 (Z scores). **(E)** Volcano plot showing DEGs between NC-*Foxc2*^*-/-*^ and *Foxc2*^*fl/fl*^ Control mice in the lymphatic-like endothelium subcluster. Blue points denote DEGs with adjusted *P*-value < 0.05. **(F)** Violin plot (left panel) and Uniform Manifold Approximation and Projection (right panel) showing the expression of *Foxc2* in each endothelial subcluster. Source data are available for this figure.

**Figure S7.**
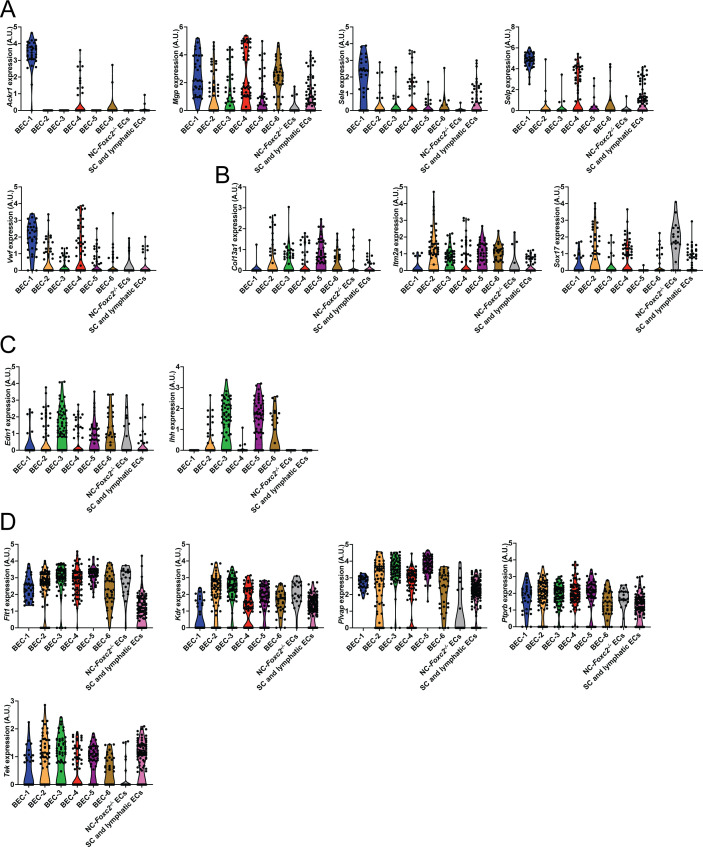
Characterization of endothelial subclusters. **(A, B, C, D)** Violin plots showing the expression of *Ackr1*, *Mgp*, *Sele*, *Selp*, and *Vwf* (A), *Col13a1*, *Itm2a*, and *Sox17* (B), *Edn1* and *Ihh* (C), *Flt1*, *Kdr*, *Plvap*, *Ptprb*, and *Tek* (D) from endothelial subclusters. Clusters are color coded as in Fig 6. Source data are available for this figure.

To directly investigate potential transcriptional changes in the SC endothelium secondarily associated with the loss of NC-*Foxc2* transcriptional activity, we performed a subclustering analysis of the endothelial cell cluster (Fig 3B) identified in our dataset to identify the population of SC endothelium. Uniform Manifold Approximation and Projection (UMAP) visualization and clustering analysis identified eight transcriptionally unique clusters consisting of six blood endothelial cell (BEC) clusters, a cluster specific to NC-*Foxc2*^*-/-*^ mice, and a cluster comprised of both SC and limbal lymphatic ECs (Fig 6A and B). The BEC-1 and BEC-4 clusters exhibited higher expression of venous endothelial markers such as *Ackr1*, *Mgp*, *Sele*, *Selp*, and *VWF* (Fig S7A). The BEC-1 cluster exhibited the strongest expression of these genes, potentially indicating that this cluster may be characteristic of collector channel vessels that exhibited a similarly high expression of these genes in a human dataset (40). The other BEC clusters showed stronger expression for markers recently reported to be associated with the arterial limbal endothelium (37), except BEC-3, which exhibited higher expression of *Ihh*, a recently identified marker of the choriocapillaris (37, 49), that may have been incorporated during dissection and tissue dissociation (Fig S7B and C). The SC and lymphatic EC cluster showed high expression of several markers identified in SC ECs, such as *Ccl21a*, *Flt4*, and *Prox1*. However, only a few cells exhibited positive expression of the classic lymphatic markers *Lyve1* and *Pdpn* (Fig 6C), which are absent in the SC, implying that this cluster predominately consists of SC ECs with few limbal lymphatic ECs that were not independently clustered during analysis. In support of this observation, the SC and lymphatic EC cluster exhibited high expression of *Npnt*, *Nts*, *Pgf*, and *Postn* and modest expression of *Itga9* and *Nts* (Fig 6C and D) and other endothelial marker genes, including *Flt1*, *Kdr*, *Plvap*, *Ptprb*, and *Tek* (Fig S7D), which were recently shown to be characteristic of the SC endothelial transcriptional profile by Thomson et al (37).

Analysis of DEGs in the SC and lymphatic EC cluster identified several transcriptional changes, including significantly decreased expression of the AP-1 transcription factor component *JunD*, which has been previously implicated in the regulation of vascular injury response (50) and protection against aging-induced oxidative stress and endothelial dysfunction (51). In contrast, the expression of *Col1a1*, which was shown to be increased in both normal and glaucomatous SC cells in response to substrate stiffness (52), was significantly up-regulated in the SC and lymphatic EC cluster (Fig 6E).

Collectively, these data demonstrate that loss of NC-*Foxc2* expression results in several transcriptional changes altering TM composition and the ECM environment that may be associated with increased matrix stiffening and reduction in TIE2 signaling, which has been associated with the progression of glaucoma.

### Endothelial *Foxc2* expression is required for SC morphogenesis via regulation of TIE2 expression

**Figure 7.**
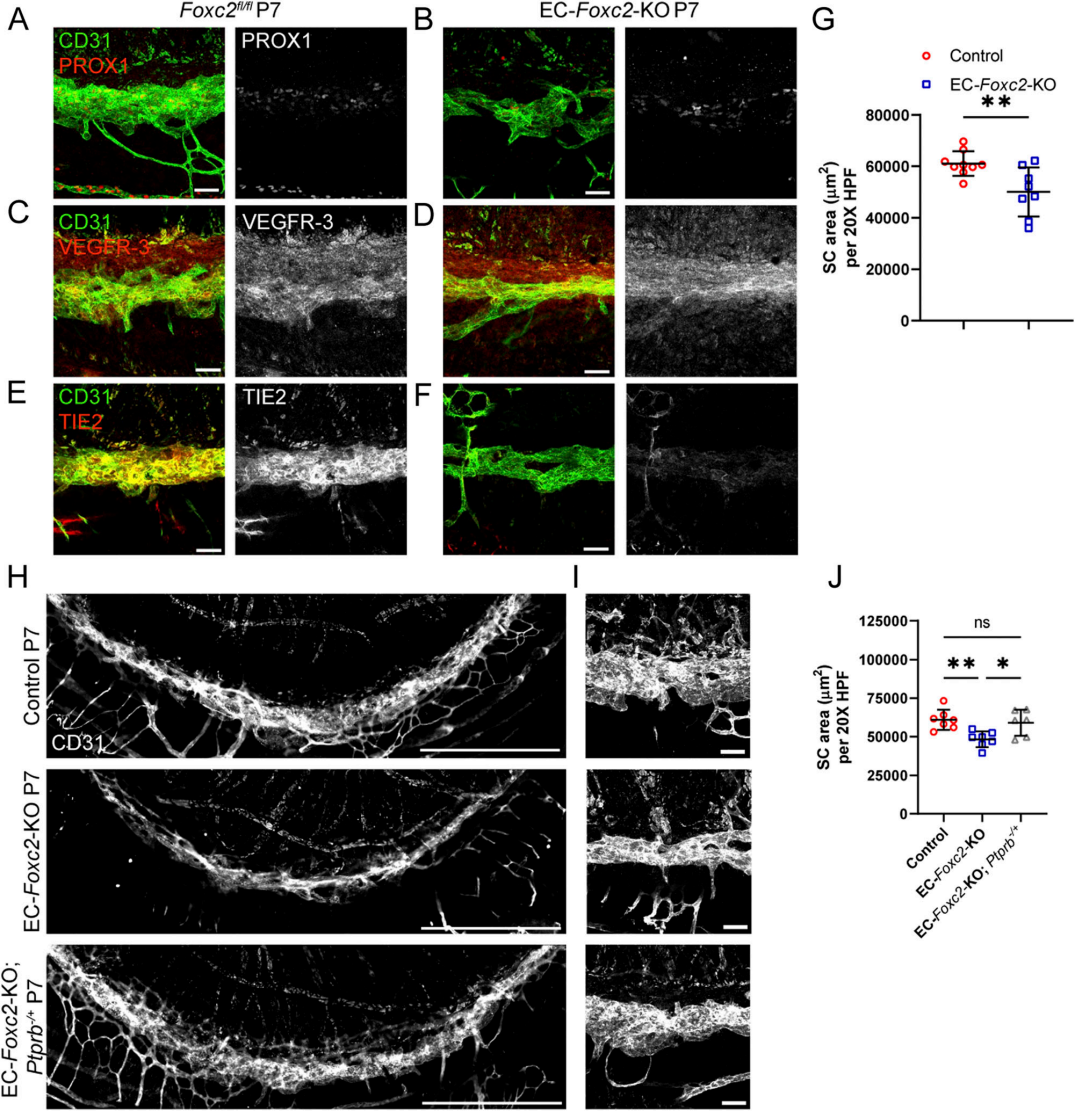
SC morphogenesis is impaired by early postnatal, endothelial-specific deletion of *Foxc2*, which is accompanied by reduced TIE2 expression and rescued by reduction of endothelial *Ptprb* expression. **(A, B, C, D, E, F)** Representative images of CD31 and PROX1 (A, B), VEGFR-3 (C, D) or Tie2 (E, F) expression in the SC of P7 *Foxc2*^*fl/fl*^ control (A, C, E) and EC-*Foxc2*-KO mice (B, D, F). Scale bars are 50 μm. **(G)** Quantification of SC area per 20X high-power field in P7 *Foxc2*^*fl/fl*^ control and EC-*Foxc2*-KO mice. N = 9 for Control and N = 8 for EC-*Foxc2*-KO mice. Data are mean ± SD. Statistical analysis: unpaired *t* test. **P < 0.01. **(H, I)** Representative images of SC vasculature immunostained with CD31 antibody in P7 Control, EC-*Foxc2*-KO, and EC-*Foxc2*-KO; *Ptprb*^*-/+*^ mice. **(H, I)** Scale bars are 500 μm (H) and 50 μm (I). **(J)** Quantification of SC area per 20X high-power field in P7 Control, EC-*Foxc2*-KO, and EC-*Foxc2*-KO; *Ptprb*^*-/+*^ mice. N = 7 for Control, N = 7 for EC-*Foxc2*-KO, and N = 6 for EC-*Foxc2*-KO; *Ptprb*^*-/+*^ mice. Statistical analysis: one-way ANOVA with Tukey’s multiple comparison test. *P < 0.05, **P < 0.01. Source data are available for this figure.

**Figure S8.**
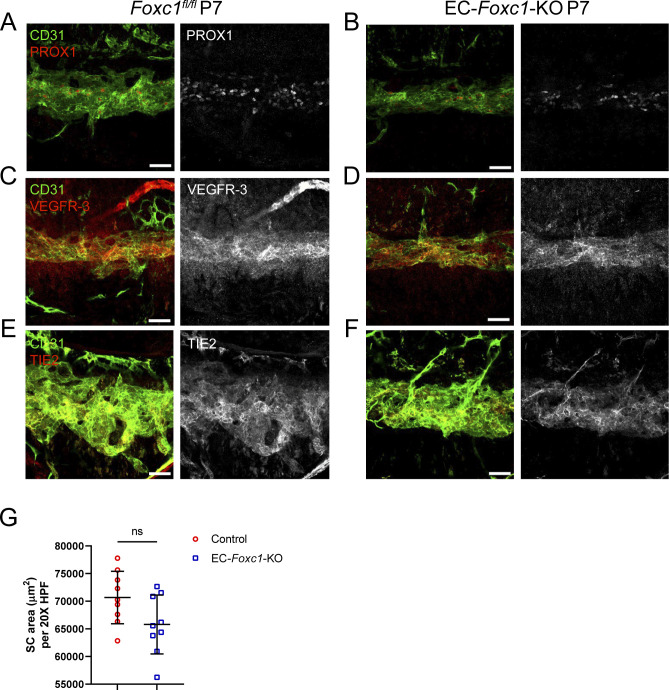
SC morphogenesis is not severely impacted by early postnatal, endothelial-specific deletion of *Foxc1*. **(A, B, C, D, E, F)** Representative images of CD31 and PROX1 (A, B), VEGFR-3 (C, D) or Tie2 (E, F) expression in the SC of P7 *Foxc1*^*fl/fl*^ control (A, C, E) and EC-*Foxc1*-KO mice (B, D, F). Scale bars are 50 μm. **(G)** Quantification of SC area per 20X high-power field in P7 *Foxc1*^*fl/fl*^ control and EC-*Foxc1*-KO mice. N = 9 for Control and N = 9 for EC-*Foxc1*-KO mice. Data are mean ± SD. Statistical analysis: unpaired *t* test. Source data are available for this figure.

*Foxc2* is a critical regulator of early lymphatic development and lymphatic maturation, maintenance, and function (10, 11, 15, 16). Foxc2+/Prox1+ cell expression was previously observed in SC endothelium by immunostaining analysis as early as P7 continuing to 2 mo of age (21). Moreover, our linage-tracing analysis demonstrated that *Foxc2*+ cell descendants contribute to the formation of the SC vasculature (Fig S1), and our scRNA-seq analysis shows that *Foxc2* was more highly expressed in the SC and lymphatic EC cluster compared with the other BEC clusters (Fig 6F). As the SC vasculature shares characteristics of lymphatic endothelium, we sought to investigate the direct role of endothelial-*Foxc2* signaling in SC morphogenesis, as its role is unknown (Fig 7). Because early, inducible postnatal blood and/or lymphatic endothelial-specific deletion of *Foxc2* results in lymphatic dysfunction and eventual mortality (10, 16), we performed the analysis at the approximate time point (P7) of SC morphogenesis after the administration of tamoxifen from P1–P5 to delete *Foxc2* during SC morphogenesis initiation. Compared with *Foxc2*^*fl/fl*^ controls, inducible, endothelial-specific deletion of *Foxc2* (*Cdh5-Cre*^*ERT2*^*; Foxc2*^*fl/fl*^, EC-*Foxc2*-KO) resulted in a hypoplastic SC vasculature (Fig 7A–F) with reduced SC area (Fig 7G), in addition to markedly impaired lymphatic valve development and maturation in the mesentery which we previously reported (16). Although P7 EC-*Foxc2*-KO mice appeared to maintain expression of PROX1 and VEGFR-3 (Fig 7B and D), reduced TIE2 expression was detected in the SC vasculature compared with *Foxc2*^*fl/fl*^ controls (Fig 7F). Because TIE2 signaling regulates PROX1 expression in the SC endothelium (25), unaltered levels of PROX1 in the EC-*Foxc2*-KO mice may be because of the early time point (P7) examined before the reported postnatal lethality (10, 16). As *Foxc1* and *Foxc2* share cooperative roles in lymphatic development and maintenance (15, 16), we sought to also characterize possible roles for endothelial-*Foxc1* transcriptional activity during SC morphogenesis (Fig S8). Compared with Cre-negative *Foxc1*^*fl/fl*^ controls, *Cdh5-Cre*^*ERT2*^*; Foxc1*^*fl/fl*^ (EC-*Foxc1*-KO) mice trended toward a reduction in SC area, but the difference was not statistically significant (Fig S8G). In addition, EC-Foxc1-KO mice normally expressed PROX1, VEGFR-3, and TIE2 (Fig S8A–F). Thus, these data indicate that similar to their individual roles in the mesenteric lymphatic vasculature (16), SC endothelial morphogenesis is predominately regulated by *Foxc2* compared with its closely related family member *Foxc1*.

*Ptprb* encodes receptor-type tyrosine-protein phosphatase β, which is also known as VE-PTP. VE-PTP negatively regulates ANGPT-TIE2 signaling by dephosphorylating the TIE2 receptor (53, 54). Notably, deletion of one *Ptprb* allele was able to rescue impaired SC morphogenesis and retinal ganglion cell loss associated with *Tie2/Tek* haploinsufficiency (28), and a small molecule inhibitor of VE-PTP, AKB-9778 was shown to activate TIE2 signaling in the SC, increase outflow facility, and reduce IOP (29). Previous studies have also demonstrated that *FOXC2* directly binds to the *TIE2/TEK* locus (55) and that siRNA-mediated knockdown of *FOXC2* reduces TIE2 expression in cultured HDLECs (22). Therefore, to more directly assess the role of *Foxc2* in the regulation of *TIE2/TEK* signaling in the SC vasculature, we generated *Cdh5-Cre*^*ERT2*^*; Foxc2*^*fl/fl*^*; Ptprb*^*fl/+*^ (EC-*Foxc2*-KO; *Ptprb*^*-/+*^) mice and performed conditional deletion postnatally from P1–P5. Compared with the development of a hypoplastic SC in postnatal EC-*Foxc2*-KO mice, EC-*Foxc2*-KO; *Ptprb*^*-/+*^ mice appeared to have normal SC vasculature (Fig 7H and I), and quantitative analysis revealed that the SC area returned to levels similar to Cre-negative *Foxc2*^*fl/fl*^ controls (Fig 7J). Thus, these results demonstrate that endothelial-*Foxc2* transcriptional activity regulates TIE2 expression to promote activation of ANGPT-TIE2 signaling during SC morphogenesis.

### *Foxc2* can functionally substitute for *Foxc1* during the development of the anterior eye segment and SC morphogenesis

**Figure 8.**
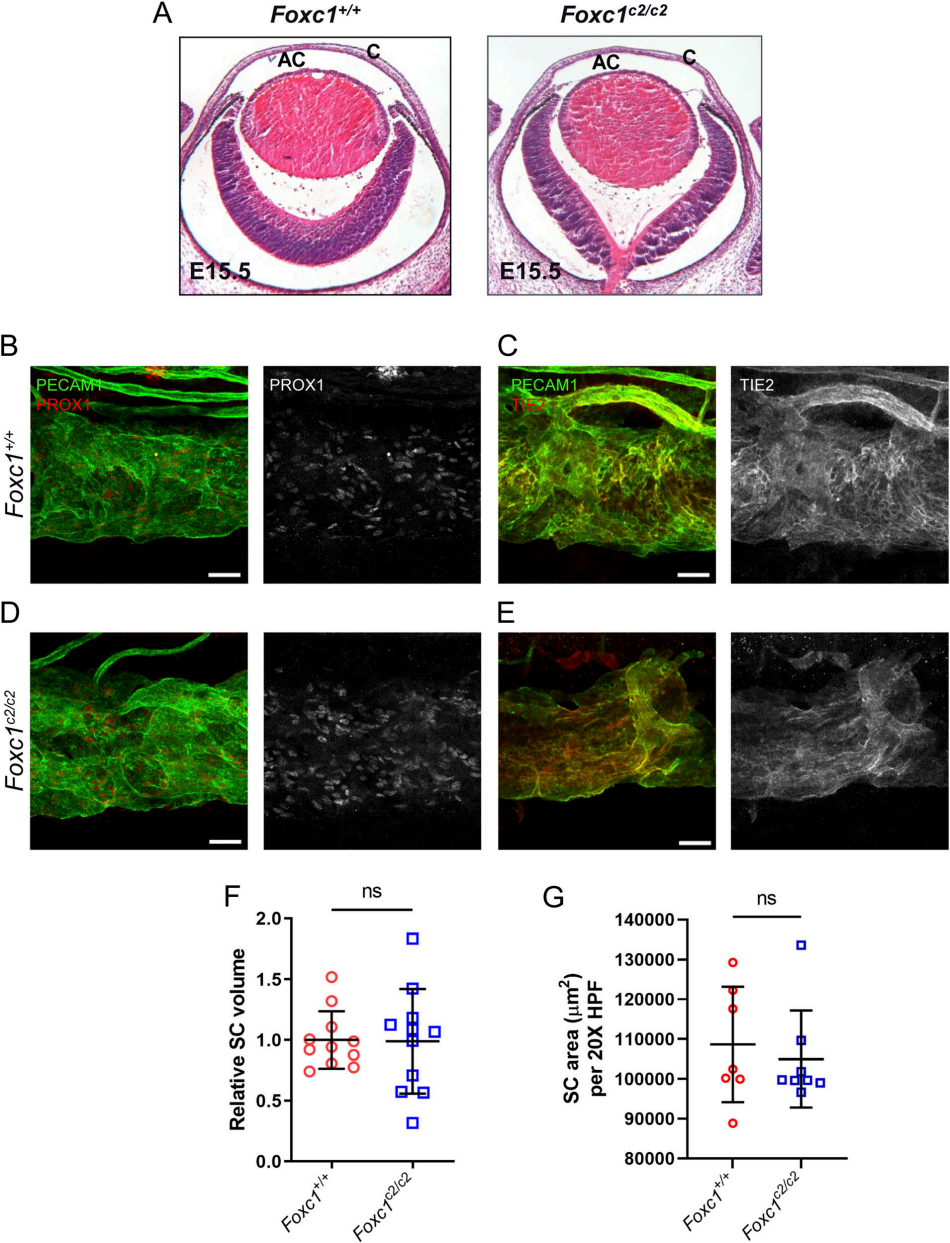
Substitution of *Foxc2* into the *Foxc1* locus does neither impair anterior eye segment development nor SC morphogenesis. **(A, E)** Representative images of hematoxylin and eosin-stained transverse eye sections from embryonic day (E) 15.5 *Foxc1*^*+/+*^ and *Foxc1*^*c2/c2*^ mice show no difference in the normal development of the anterior chamber. AC, anterior chamber, C, cornea. **(B, C, D, E)** Representative images of CD31 and PROX1 (B, D) or Tie2 (C, E) expression in the SC of adult *Foxc1*^*+/+*^ (B, C) and *Foxc1*^*c2/c2*^ mice (D, E). Scale bars are 50 μm. **(F)** Relative SC volumes of *Foxc1*^*c2/c2*^ and *Foxc1*^*+/+*^ mice in a 1.5 mm × 1.5 mm field of view. SC volume for both groups was normalized by mean *Foxc1*^*+/+*^ SC volume. N = 11 volumes from 11 individuals for *Foxc1*^*+/+*^ and N = 11 volumes from 11 individuals for *Foxc1*^*c2/c2*^ mice. **(G)** Quantification of SC area per 20X high-power field. N = 7 for *Foxc1*^*+/+*^ and N = 8 for *Foxc1*^*c2/c2*^ mice. Data are mean ± SD. Statistical analysis: unpaired *t* test. Source data are available for this figure.

FOXC1 and FOXC2 share nearly identical forkhead DNA-binding domains and function cooperatively during early cardiovascular (14) and ocular (18) development, and during embryonic lymphangiogenesis (15) and postnatal lymphatic valve development and maintenance (16). However, mutations in *FOXC1* are predominately associated with the ocular autosomal dominant disorder Axenfeld-Rieger syndrome and progression of secondary glaucoma (56), and NC-*Foxc1*^*-/-*^ mice is perinatal lethal (17, 57) compared with NC-*Foxc2*^*-/-*^ mice, thus underscoring the critical role for NC-*Foxc1* function. We previously reported the generation of mice that carry a *Foxc2* knock-in allele (*Foxc1*^*c2*^) in which the *Foxc1*-coding region has been replaced with the cDNA coding for *Foxc2* and that these mice appear to develop normally and do not exhibit abnormal development of the mesenteric lymphatic vasculature (16). To investigate whether the development of the anterior eye segment and SC morphogenesis is similarly conserved, we assessed ocular phenotypes in homozygous *Foxc1*^*c2/c2*^ mice and WT (*Foxc1*^*+/+*^) controls by immunohistochemical analysis (Fig 8). At embryonic day 15.5, the development of the anterior chamber and cornea appeared morphologically similar to *Foxc1*^*+/+*^ controls (Fig 8A). Analysis of 6–8-wk-old adult mice showed that there were no obvious morphological differences in the SC between *Foxc1*^*+/+*^and *Foxc1*^*c2/c2*^, and the expressions of key SC markers such as PROX1 and TIE2 were maintained (Fig 8B–E). Quantitative analysis of relative SC volume by vis-OCT and quantification of the CD31-immunostained SC area did not identify significant differences between either group (Fig 8F and G). Thus, these data demonstrate that, like our group’s previous observations regarding lymphatic development and maturation, FOXC2 can functionally substitute for FOXC1 during anterior segment development.

## Discussion

**Figure 9.**
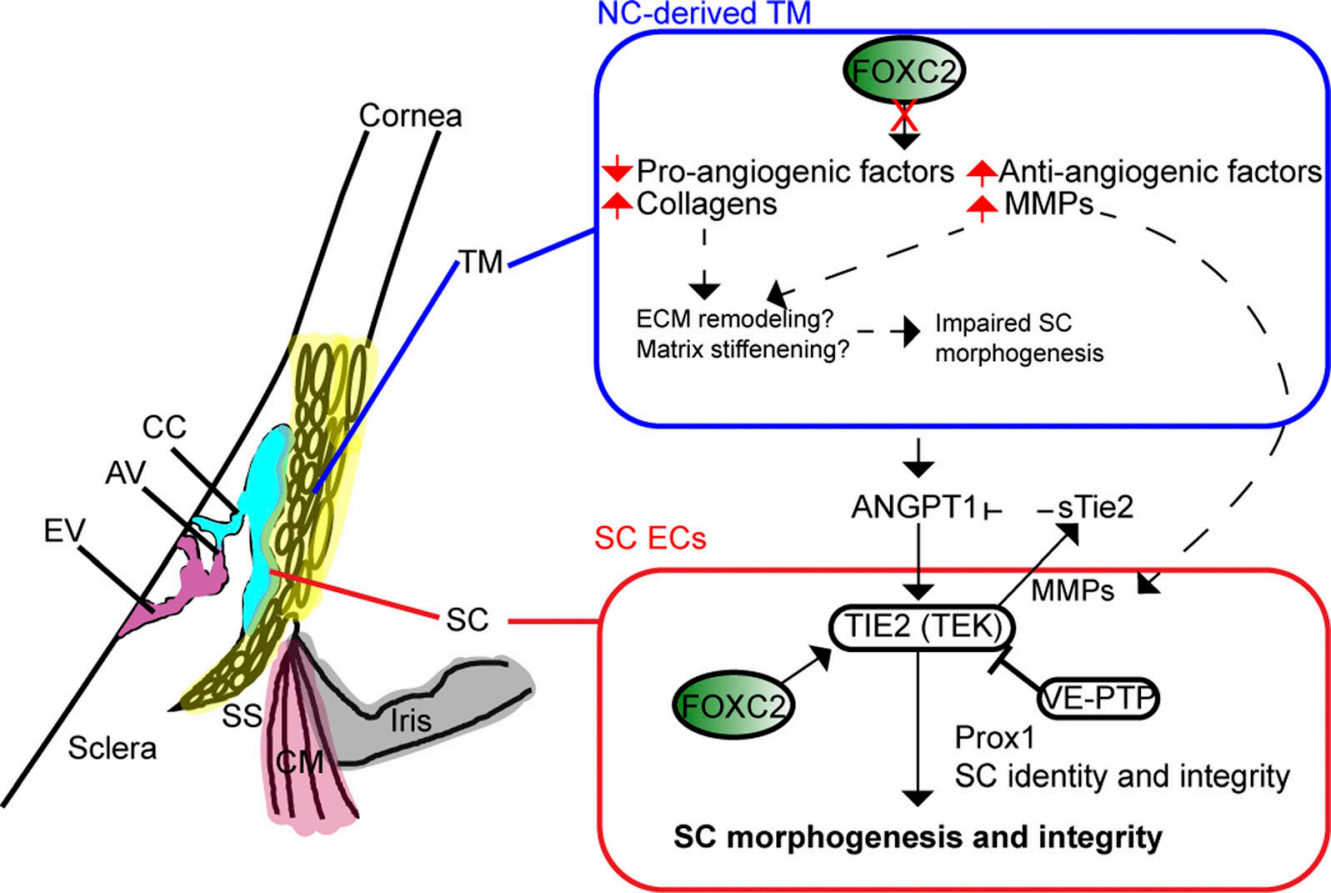
Neural crest- and endothelial- derived *Foxc2* differentially regulate proper morphogenesis of the SC. Neural crest-specific deletion of *Foxc2* results in several transcriptional changes in the trabecular meshwork including the decrease in expression of pro-angiogenic factors and in contrast an increase in the expression of anti-angiogenic factors, collagens, and MMPs that likely result in abnormal ECM remodeling, matrix stiffening, and induction of TIE2 shedding that potentially contribute to impaired SC morphogenesis. In contrast, endothelial-derived Foxc2 regulates the expression of TIE2 to promote ANGPT1-mediated activation of the TIE2 receptor and, in turn, proper SC morphogenesis and maintenance of SC integrity. TM, trabecular meshwork; SC, Schlemm’s canal; SS, scleral spur; CM, ciliary muscle; CC, collector channel; AV, aqueous veins; EV, episcleral veins.

Critical to the development of glaucoma is ocular hypertension resulting from abnormally increased IOP, which is tightly regulated by control of the outflow facility in part through the conventional outflow pathway consisting of the TM and SC that drain into the ocular veinous circulation (6). Although several recent studies have implicated the direct roles of key vascular signaling events contributing to the morphogenesis and functional maintenance of the SC during early postnatal development and adulthood (21, 22, 24, 25, 26), the role of paracrine signaling events from the TM and environmental cues contributing to proper morphogenesis and maintenance of SC is not well understood. In this study, we identify a critical role for NC-*Foxc2* in maintenance of SC endothelial identity and its proper morphogenesis in a non-cell autonomous manner. In contrast, we also demonstrate that endothelial-*Foxc2* is required for TIE2 expression in the SC endothelium in a cell-autonomous manner, thus highlighting key differences in the functional role of FOXC2 transcriptional regulation in TM-SC crosstalk (Fig 9).

Our group had previously reported that NC-specific deletion of *Foxc2* is associated with several anterior segment abnormalities, including ectopic corneal neovascularization (18); however, the outcome on development and changes within the conventional outflow pathway, and in particular TM-SC regulatory pathways, was not well elucidated. Whereas SC/TM malformations were observed in mice with *global Foxc2* haploinsufficiency (58), NC-*Foxc2* heterozygous mice normally developed the SC vasculature (Fig 2A and B). Although the reason(s) for this discrepancy remains unclear, potential factors contributing to the phenotypic differences between the global and NC-*Foxc2* mutant lines could be the timing of *Foxc2* deletion induced by *Wnt1-Cre* compared with global *Foxc2* deletion and unidentical genetic backgrounds between the two lines.

By applying an in vivo vis-OCT imaging technique (35) for the first time to characterize SC phenotypes in a transgenic knockout model of abnormal anterior segment development, we identified significant SC volume differences between *Foxc2*^*fl/fl*^ control and NC-*Foxc2*^*-/-*^ - mice at every IOP level from Δ−10 mmHg to Δ+5 mmHg (Fig 1I). Such observations indicate potential increased outflow resistance in SC in NC-*Foxc2*^*-/-*^ mice at physiological IOPs. Further analysis quantifying changes in SC volume and height in response to IOP modulation suggests a decreased TM strain in NC-*Foxc2*^*-/-*^ mice resulting from natural variations in IOP (Fig 1J and K). As sensing of the TM strain has been postulated as one of the biomechanical cues for TM signaling (59), NC-*Foxc2*^-/-^ mice likely have an impaired capability of their TM to remodel correctly in response to IOP changes. Indeed, the slope of SC volume and height as a function of IOP was lesser in magnitude in NC-*Foxc2*^*-/-*^ mice, indicating lesser deformations of SC and TM tissue in response to changes in IOP. Further analysis quantifying the normalized SC volume and height revealed patterns possibly suggesting increased TM stiffness in NC-*Foxc2*^*-/-*^ mice compared with the *Foxc2*^*fl/fl*^ control mice (Fig 1L and M). In cases where the TM tissue is stiffer, one expects normalized volume and height to decrease at a slower rate in response to increases in IOP (60). The height of the SC is primarily influenced by the pressure difference across the TM, with higher IOPs leading to reductions in height. At typical IOPs, the tension in the TM keeps the SC open, although at higher IOPs, septae within the SC prevent the complete collapse of the SC (61). The magnitude of the slope of a linear fit of normalized SC height versus IOP was greater for *Foxc2*^*fl/fl*^ mice (Fig 1M), indicating that the TM of *Foxc2*^*fl/fl*^ may be more responsive to compression and thus less stiff (60, 62). However, the magnitude of the slope for the linear fit of normalized SC volume versus IOP was not statistically different for *Foxc2*^*fl/fl*^ mice, although it was greater in magnitude. Thus, although the relation between IOP and SC height change suggests differences in TM stiffness, we are unable to make definitive conclusions based on vis-OCT data only.

**Figure S9.**
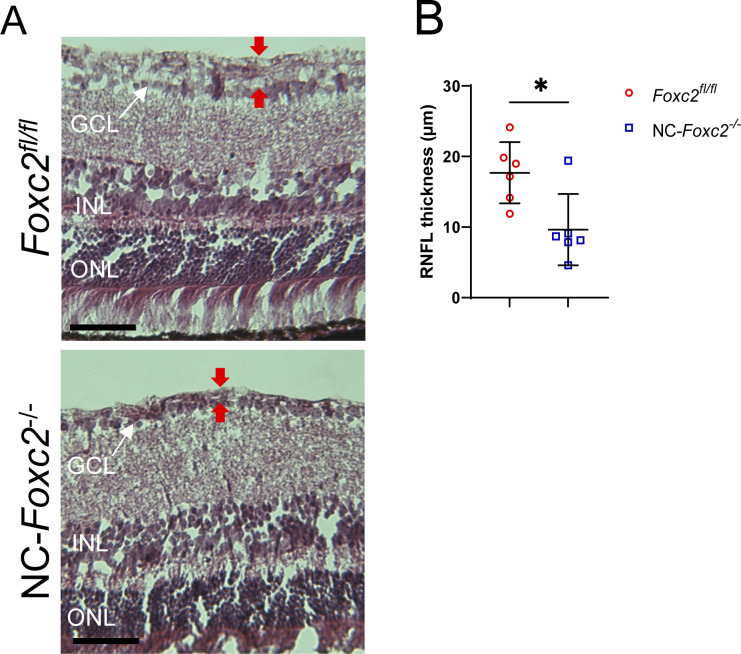
Characterization of retina nerve fiber layer thickness in NC-*Foxc2*^*-/-*^ mice. **(A, B)** Representative images and quantification of retina nerve fiber layer thickness from hematoxylin and eosin-stained sections. Scale bars are 50 μm. N = 6 *Foxc2*^*fl/fl*^ control and N = 6 for NC-*Foxc2*^*-/-*^ mice. Data are mean ± SD. Statistical analysis: unpaired *t* test. *P < 0.05. ONL, outer nuclear layer, INL, inner nuclear layer, GCL, ganglion cell layer. Source data are available for this figure.

In support of a change in ECM composition potentially leading to increased TM stiffness, our scRNA-seq analysis between *Foxc2*^*fl/fl*^ and NC-*Foxc2*^*-/-*^ mice identified several DEGs which provide additional evidence of differences in ECM matrix composition in the TM. For example, many of the DEGs up-regulated in NC-*Foxc2*^*-/-*^ are involved in an ECM organization and collagen chain trimerization (Fig 4). A the scRNA-seq data does not directly prove increased TM stiffness in NC-*Foxc2*^*-/-*^ mice, there is substantial evidence of differences in the ECM organization leading to altered TM elasticity in NC-*Foxc2*^*-/-*^ mice, as is illustrated by the trends in SC volume and height. Although changes in SC area and height with IOP changes provide clues related to TM stiffness (60, 62), further work is required to numerically quantify the stiffness. Given the relevance of TM biomechanics in glaucoma development and the observation of elevated IOP in NC-*Foxc2*^*-/-*^ mice, further studies focused on the role of Foxc2 on TM biomechanics are warranted. In addition, it is important to consider whether the elevations in IOP we observe in NC-*Foxc2*^*-/-*^ mice are associated with loss of retinal ganglion cells and the progression of glaucomatous neuropathy, as previously demonstrated (63, 64). Characterization of the retina nerve fiber layer of NC-*Foxc2*^*-/-*^ mice demonstrated that it was thinner compared with *Foxc2*^*fl/fl*^ controls (Fig S9A and B), consistent with other models of impaired SC morphogenesis leading to glaucomatous neuropathy (25). However, it is important to consider that this phenotype may be secondary to potential ocular development defects present in the posterior segment of NC-*Foxc2*^*-/-*^ mice.

Additional changes in ECM matrix composition and signaling may be attributable to impaired SC morphogenesis observed in NC-*Foxc2*^*-/-*^ mice as well. Changes in ECM composition and matrix stiffness have dynamic effects on vascular signaling (65), and increases in collagen density impaired neovessel length and interconnectivity in an in vitro model of sprouting angiogenesis (66). Similarly, recent evidence has shown that reduced matrix stiffness primes LECs to form cord-like structures and increases the expression of lymphatic markers such as LYVE-1 and PROX1 in vitro in response to VEGF-C (67) and promotes increased GATA2 expression and GATA2-dependent up-regulation of genes involved in cell migration and lymphangiogenesis (68). Notably, we show increased expression of *Mmp2* and *Mmp3* in TM-3 (Fig 5B). Evidence has shown that the TM increases secretion of MMP-2, -3, and -14 in response to IOP elevation and mechanical stretching forces, although these changes were not accompanied by increases in their mRNA levels but likely by selective translation mediated by mammalian target of rapamycin (69). However, it is possible that increased expression of genes associated with ECM remodeling may be in response to abnormal development of the anterior segment as opposed to other mechanisms generating outflow resistance. Whereas increased expression of MMP-2 and MMP-14 is associated with stimulating angiogenesis via ECM turnover (70), the increased expression of MMPs may have a negative impact on SC morphogenesis. Several studies have demonstrated that MMPs mediate cleavage and shedding of the TIE2 ectodomain to produce sTIE2, which acts as an endogenous inhibitor by trapping ANGPT1 (30, 47). Consistently, as the TIE2 antibody used for this study recognizes the extracellular domain of TIE2, reduced TIE2 levels in the SC endothelium of NC-*Foxc2*^*-/-*^ mice (Fig 2) are likely to be because of, at least in part, TIE2 shedding by excess MMPs. Thus, provided the critical role of ANGPT/TIE2 signaling in SC morphogenesis and maintenance and our observations that (1) TIE2 expression is reduced in the SC endothelium of NC-*Foxc2*^*-/-*^ mice and (2) MMP activity mediates TIE2 shedding in cultured HDLECs, it is reasonable to speculate that activation of ANGPT/TIE2 signaling is reduced, resulting in loss of SC identity establishment (Fig 9).

Our scRNA-seq analysis also shows a significant increase of several pro-angiogenic factors in the TM (Fig S6 and Table S1), including *Vegfa*. We previously reported that loss of NC-*Foxc1* is associated with increased MMP signaling and VEGF-A bioavailability in the corneal stroma (57). Notably, we also observed a significant increase in *Vegfa* mRNA expression within the corneal stroma keratocyte population in our scRNA-seq analysis (Table S1). As SC sprouting morphogenesis is not initiated from the limbal lymphatic vasculature and instead from the blood limbal vascular plexus and radial vessels, which requires VEGFR-2 function (20, 23), it is possible that the severe cornea neovascularization and subsequent impairment in SC morphogenesis may be attributable to alterations in VEGF-A bioavailability and alterations in VEGF gradient signaling in NC-*Foxc2*^*-/-*^ mice.

In contrast to the role of NC-*Foxc2* expression in the TM cells, our results demonstrate that endothelial-*Foxc2* is necessary for regulation of TIE2 expression in SC endothelium (Fig 7) and support previous findings identifying a role for FOXC2 in the regulation of TIE2 expression in both blood and lymphatic endothelia (22, 55). The Foxc2–TIE2 axis in the SC endothelium is further supported by our finding that *Ptprb* haploinsufficiency rescued the SC phenotype observed in endothelial-specific *Foxc2* mutant mice. Although the significance of maintenance of ANGPT/TIE2 signaling activity in the SC is well understood, it is plausible that FOXC2 has other critical functional roles in the SC endothelium as well. Because FOXC2 regulates gene expression under shear stress in the lymphatic vasculature (10, 11, 15, 16), whether FOXC2 participates in fluid shear stress-dependent regulation in the SC endothelium remains to be elucidated and will be the focus of further investigation.

Together, the data presented in this report present a unique bi-functional role for *Foxc2* in both neural crest-derived TM and SC endothelial cells in the proper morphogenesis and maintenance of the SC. Given that our evidence also demonstrates that *Foxc2* can functionally substitute for *Foxc1* during ocular morphogenesis, including the SC vasculature (Fig 8), this work may also offer insight into pathological signaling mechanisms associated with *Foxc1* mutations in Axenfeld-Rieger syndrome and the progression of secondary glaucoma to identify novel therapeutic targets.

## Materials and Methods

### Animal generation and husbandry

Mice were housed and kept under normal lighting conditions with 12-h on, 12-h off cycles in the Center for Comparative Medicine at Northwestern University. *Wnt1-Cre; Foxc2*^*fl/fl*^ (NC-*Foxc2*-KO) mice were generated and maintained on a mixed genetic background, as reported previously (17, 18). Endothelial-specific *Foxc1* and *Foxc2* knockout mice were generated and induced with tamoxifen dissolved in corn oil as previously described (16). Briefly, neonates were orally administered 75 μg of tamoxifen dissolved in corn oil from postnatal day (P)1 – P5 to induce gene deletion and mice were euthanized at the indicated time points for analysis. For cell fate mapping, *Wnt1-Cre; Foxc2*^*fl/fl*^ and *Foxc2-Cre*^*ERT2*^ mice (32) were crossed with mTmG reporter mice (The Jackson Laboratory). *Cdh5-Cre*^*ERT2*^*; Foxc2*^*fl/fl*^*; Ptprb*^*fl/+*^ (EC-*Foxc2*-KO; *Ptprb*^*+/-*^) mice were generated by crossing *Ptprb*^*fl/fl*^ mice (71), acquired from Northwestern University’s NU GoKidney Preclinical Models Core, with *Cdh5-Cre*^*ERT2*^*; Foxc2*^*fl/fl*^ mice through several generations. *Foxc2* knock-in mice (*Foxc1*^*c2/c2*^) were generated as described previously (16). Genotyping of mice for use in analysis was performed by Transnetyx Inc using real-time PCR. All experimental protocols and procedures used in this study were approved by the Institutional Animal Care and Use Committee (IACUC) at Northwestern University.

### IOP measurements

IOP measurements were performed in adult mice using a Tonolab rebound tonometer as previously described (24, 26, 37). Mice were restrained in a soft plastic cone, and average IOPs will be recorded from three sets of six recordings performed. Finding no difference between left and right eyes, all IOP measurements will be recorded as single averaged values for each animal.

### Outflow facility measurements

Outflow facility was measured in enucleated and shipped adult mouse eyes using the iPerfusion system as described previously (72, 73, 74). Briefly, mice were euthanized, and eyes enucleated and shipped overnight to Duke University in low-glucose DMEM in a container with ice packs for ex vivo outflow facility measurements. The next morning, the eyes were submerged in warm DBG (PBS++ with glucose) in a temperature-controlled perfusion chamber. The anterior chambers of the enucleated eyes were cannulated with a pulled, beveled, and sharpened glass pipet containing degassed DBG. The eyes first underwent an acclimation phase of 30 min at 12 mmHg before starting outflow facility measurement using nine steps, which started at 5 mmHg, increased 1.5 mmHg each step until reaching 17 mmHg, then decreased to 8 mmHg for the final step. Perfusion data were analyzed using iPerfusion software and traces were generated using MATLAB software.

### In Vivo imaging of Schlemm’s canal using visible-light OCT

In vivo imaging of the mouse SC was performed using a custom-built anterior segment vis-OCT microscopy system as previously described (35). Briefly, mice were anesthetized by intraperitoneal injection (10 ml/kg bodyweight) of ketamine xylazine cocktail (ketamine: 11.45 mg/l; xylazine: 1.7 mg/ml, in saline) before imaging procedures. During imaging, body temperature was maintained by a heating lamp. The entire 360 degrees of the SC and surrounding vasculature were captured in eight separate raster scans, with a rotating two-mirror assembly used to change the field of view between scans as previously described (35). Each raster scan had a 1.8 mm × 1.8 mm field of view. The spatial resolutions of the system in tissue are 7 μm laterally and 1.3 μm axially. Vis-OCTA detecting motion contrast from flowing blood cells was used to visualize the surrounding vasculature, with each B-scan repeated five times and processed as previously described (75).

To assess changes in SC volume in response to alterations in IOP, the anterior chamber was cannulated with a 34-gauge needle, and the IOP level was manometrically set before acquiring a vis-OCT raster image as previously described (35). The 34-gauge needle was connected to a saline column, whose position relative to the eye controls the IOP level. Only the nasal most raster scan was used for volume calculation. One vis-OCT dataset was acquired at each of 5 IOP levels, and each IOP level was repeated 3 times. A 1.5 mm length of the SC was segmented from each individual raster scan, with SC considered the largest connected lumen positioned immediately lateral to the iridocorneal angle. All SC volumes are reported as volumes normalized by the average control mouse volume at baseline IOP as done by the same person conducting each experiment. For calculation of SC width and height, an ellipse was fitted to the segmented SC in every segmented cross-sectional B-scan image using the *regionprops* function in MATLAB 2020b (MathWorks). In cases where the SC was composed of multiple parts, an ellipse was fitted to every part of the SC with an area at least 20% that of the largest SC area. SC width was calculated by summing the major axis length of the fitted ellipses within each cross-section and averaging the value across all cross-sections. SC height was calculated by taking the weighted average by area of the minor axis length of the fitted ellipses and averaging across all cross-sections. Peripheral cornea thickness was found by manually segmenting the cornea at the iridocorneal angle in three different cross-sections for each eye and taking the average.

### Tissue section processing, histologic and immunohistochemical analyses

Whole embryos were fixed in 4% PFA for 2 h at 4°C, dehydrated with methanol, embedded in paraffin, and cut into 8-μm sections. Adult eyeballs were immersion fixed in 4% PFA overnight at 4°C, followed by immersion in 30% sucrose (wt/vol in PBS) overnight at 4°C. Tissues were then embedded in OCT Compound (Tissue-Tek) and frozen in an ethanol/dry ice bath. Frozen tissues were cut into 8-μm sections. Both paraffin sections from whole embryos and frozen sections from adult eyeballs were stained with hematoxylin and eosin (H&E) and subject to immunohistochemistry. H&E staining images were acquired using an Olympus Vanox AHBT3 Research Microscope using a 4X or 40X objective. For immunohistochemical analysis of markers from frozen sections, sections were blocked with 10% normal donkey serum (Sigma-Aldrich Corp.) in PBS with 0.1% Triton X-100 (Sigma-Aldrich Corp.). After blocking, sections were then incubated in a blocking buffer with primary antibodies listed in Table S2 overnight at 4°C. Sections were then washed with PBS/0.1% Tween 20 and incubated with secondary antibodies conjugated to AlexaFluor 488, Alexafluor 568 or AlexaFluor 647 listed in Table S2 for 2 h at room temperature. Wash steps were repeated; then, the sections were counterstained with 4′6-diamidino-2-phenylindole (DAPI) and mounted with PermaFluor aqueous mounting media (Thermo Fisher Scientific). Images of the iridocorneal angle were captured on a Nikon A1R confocal microscope at the Northwestern University Center for Advanced Microscopy using a 20X objective with a numerical aperture of 0.75 and a pinhole of 1.2 Airy units to collect Z-stacks. Images were acquired using Nikon NIS-elements software and are shown as maximum intensity projections, which were generated using Fiji software and post-processed using Adobe Photoshop. Large-field images of eyes were obtained by stitching images captured using a 10x objective with a numerical aperture of 0.3 and pinhole of 1.2 Airy units. Images were post-processed using Adobe Photoshop.

Table S2 Antibodies and dyes used for immunohistochemistry analysis.

### Wholemount immunostaining analysis of SC morphology

Mice were euthanized at the indicated time points, eyes were enucleated, and then immersion was fixed in 2% PFA overnight at 4°C. Eyes were then bisected from the optic nerve to the center of the cornea, and the lens and retina tissue were removed. Tissues were then blocked in a buffer containing 5% donkey serum, 2.5% (BSA; Sigma-Aldrich Corp.), and 0.5% Triton X-100 in TBS pH 7.4 overnight at 4°C on a shaker. After blocking, the tissues were incubated with primary antibodies listed in Table S2 diluted in the blocking buffer overnight at 4°C on a shaker. Tissues were then washed in 0.05% Tris-buffered Tween-20 (TBST) solution and then incubated with secondary antibodies listed in Table S2 and diluted in the blocking buffer overnight at 4°C on a shaker. Wash steps were repeated, then the tissues were further processed by making additional cuts in the cornea and scleral regions to flat-mount tissues on glass microscope slides with PermaFluor aqueous mounting medium to visualize the SC vasculature. Flat-mounted tissues were imaged using a Nikon A1R confocal microscope at the Northwestern University Center for Advanced Microscopy. Images of SC vasculature were captured using a 20X objective with a numerical aperture of 0.75 and a pinhole of 1.2 Airy units to collect Z-stacks. Images were acquired using Nikon NIS-elements software and are shown as maximum intensity projections, which were generated using Fiji software and post-processed using Adobe Photoshop. Large-field images of SC morphology were obtained by stitching images captured using the same 20x objective and a fully opened 150-μm pinhole. Images were post-processed using Adobe Photoshop.

### Preparation of single-cell suspension from mouse anterior eye segment for scRNA-seq analysis

For scRNA-seq, eyes were pooled from 4–6 mice of both 3–4-wk-old *Foxc2*^*fl/fl*^ control and NC-*Foxc2*^*-/-*^ mice. The anterior segment from each eye was dissected in ice-cold dye-free DMEM containing 10% FBS and the iris was gently removed using fine forceps. Pooled tissues from each group were then chopped with Vannas Scissors in ice-cold DMEM, then digested in DMEM containing 10% FBS, 1 mg/ml Collagenase A (Millipore Sigma), and 10 μM Y-27632 (R&D Systems) for 2 h at 37°C. Tissues were then washed in 1X PBS solution, then further digested in 0.25% Trypsin solution containing 10 μM Y-27632 and 0.2 mg/ml DNAse I (Sigma-Aldrich) at 37°C for 25 min with shaking and gentle trituration using a P1000 pipettor and wide-bore pipette tips. After dissociation, the tissues were centrifuged, and the supernatant was removed. Pelleted cells were washed and resuspended in warm DMEM containing 10% FBS, then passed through a Flowmi 40 μm cell strainer before repeating centrifugation. Washing and centrifugation were repeated once more to pellet cells, which were then resuspended in 100 μl of ice-cold HBSS containing 1% BSA. Cell viability was assessed using the Cellometer Auto 2000 Cell Viability Counter. Cell viability of 70% was used as a minimum threshold.

### Single-cell 3’ gene expression library construction and sequencing

Single-cell 3′ gene expression libraries were constructed from samples using the Chromium Next GEM Single Cell 3′ Reagent Kits v3.1 (10x Genomics) according to the manufacturer’s instructions. Libraries were then assessed for quality (TapeStation 4200; Agilent) and then processed for paired-end sequencing on an Illumina HiSeq 4000 platform (Illumina); 10,000 cells were targeted for each sample with a sequencing depth of 20,000 read pairs per cell.

### Pre-processing of single-cell RNA data

After library construction and sequencing, raw sequencing data were de-multiplexed and mapped to the mouse reference genome (mm10) using the CellRanger toolkit (10X Genomics, version 4.0.0). Gene expression matrices were then generated from *Foxc2*^*fl/fl*^ control and NC-*Foxc2*^*-/-*^ mice. The matrix files were then used for data processing and downstream analysis using the BIOMEX browser-based software platform and its incorporated packages developed in R (76). Quality control and data pretreatment was performed in BIOMEX with the following manually set parameters: (i) genes with a row average of <0.005 were excluded for downstream analysis and (ii) cells in which over 8% of unique molecular identifiers were derived from the mitochondrial genome were considered as dead cells and removed from downstream analysis. The data were then normalized in BIOMEX using similar methodology to the *NormalizeData* function as implemented in the *Seurat* package (77).

### Variable gene identification, dimensionality reduction, clustering analysis, and differential gene expression analysis

After data pretreatment, BIOMEX was used for downstream dimensionality reduction of data and clustering analysis using the incorporated R packages. First, highly variable genes (HVGs) were identified utilizing the following feature selections: mean lower threshold = 0.01, mean higher threshold = 8, dispersion threshold = 0.5. Data (using HVGs only) were then auto-scaled and summarized by principal component analysis, followed by visualization using t-distributed stochastic neighbor embedding (t-SNE; top 15 principal components [PCs]) to reduce the data into a two-dimensional space. Graph-based clustering was then performed in BIOMEX to cluster cells according to their respective gene expression profiles using a methodology similar to the *FindClusters* function in *Seurat* (clustering resolution = 0.8, k-nearest neighbors = 25). Clusters formed by doublets were then removed before further analysis. Clusters containing doublets could be identified by high-expression levels of marker genes characteristic of several cell types and the lack of uniquely expressed genes.

For analysis of specific endothelial cell types, dimensionality reduction was repeated on the endothelial cell cluster, characterized by high expression levels of *Pecam1* and *Cdh5*, through utilization of principal component analysis on identified HVGs (mean lower threshold = 0.01, mean higher threshold = 8, dispersion threshold = 0.5) followed by UMAP. Graph-based clustering was then repeated in BIOMEX, using cluster resolution = 0.8 and k-nearest neighbors = 25.

Marker set analysis was performed in BIOMEX on HVGs to identify gene markers highly expressed in each initial cluster using a similar methodology described previously (78). Marker genes were then compared with previously reported scRNA-seq data characterizing the tissues of the anterior eye segment and aqueous humor outflow pathway (38, 39, 40, 41) to identify unique cell populations. Clusters with highly similar expression patterns indicative of the same cell phenotype were merged into the same cluster. Marker set analysis was then repeated on characterized cell clusters to identify top marker genes, which were used for generation of heatmap visualization.

Differential gene expression analysis between Control and NC-*Foxc2*^*-/-*^ mice for individual cell clusters was performed in BIOMEX using the Model-based Analysis of Single-cell Transcriptomics package.

### scRNA-seq data visualization

BIOMEX implementation of *Plotly* package was used for t-SNE and UMAP visualization. BIOMEX implementation of the *Heatmaply* package was used for heatmap visualization. Heatmaps were based on cluster-averaged gene expression and data were autoscaled for visualization.

### Cell culture, GM-6001 administration, and ELISA

HDLECs (C12216; PromoCell) were cultured from passages 5–7 on fibronectin-coated plates with Endothelial Cell Growth Medium MV2 (PromoCell). Cells were cultured in 6-well plates until confluent, washed with cold PBS three times, and then supplemented with serum-free DMEM in the presence of DMSO or the MMP inhibitor GM-6001 (Tocris Biosciences) at 100 nM concentrations. Cells were cultured 24 h before collecting conditioned media that were centrifuged (13,000*g*, 5 min) two times. Quantification of the concentration of sTIE2 in conditioned media was performed by ELISA using a human TIE-2 Quantikine ELISA kit (R&D) following instructions from the manufacturer.

### Quantification and statistical analysis

Statistical analyses were performed using GraphPad Prism 9. For quantification of the SC area, 4–8 20X high-power fields were acquired per individual and the CD31^+^ area was measured and averaged. For comparisons of average measurements between two groups, two-tailed, unpaired *t* test was used. For comparisons of measurements between more than two groups, one-way ANOVA was used with Tukey’s multiple comparisons test. For comparisons of mean values between two groups of mice at multiple IOP levels, two-way ANOVA was used with Šídák’s multiple comparisons test. For quantification of TIE2 expression, 3–8 high-power fields were acquired per individual and mean TIE2 levels were assessed using ImageJ. Statistical analysis was then performed using a nested *t* test. Statistical analysis for comparison of gene expression between *Foxc2*^*fl/fl*^ and NC-*Foxc2*^*-/-*^ in scRNA-seq violin plot datasets was completed using a Mann–Whitney test. For quantification of sTIE2 concentration, three biological replicates were measured by ELISA. Statistical analysis was then performed using a Kruskal–Wallis test with Dunn’s multiple comparisons. Pathway enrichment analysis on DEGs was performed using Metascape (79) on the KEGG, Canonical Pathways, GO, Reactome, and CORUM databases. *P* < 0.05 was determined to be statistically significant.

## Supplementary Material

Reviewer comments
