# Supplementary material for: Differential roles of FOXC2 in the trabecular meshwork and Schlemm’s canal in glaucomatous pathology
Source: Life Sci Alliance. 2023 Jul 6;6(9):e202201721. doi: 10.26508/lsa.202201721 (PMC10326420; doi:10.26508/lsa.202201721)
Supplement: Supplementary file 19 [file LSA-2022-01721_TableS1.pdf]

Supplementary Table 1. Differentially expressed genes (DEGs) in selected cell clusters from scRNA-seq analysis in *Foxc2$^{fl/fl}$* and NC-*Foxc2$^{-/-}$* mice.

Cluster	Feature	Log fold change	Average expression	Pvalue	Adjusted pvalue
Trabecular meshwork 1	Phlda1	-0.815053604	2.567940233	1.94E-30	1.19E-27
Trabecular meshwork 1	Ptn	-0.779570683	1.381639775	2.41E-21	7.05E-19
Trabecular meshwork 1	Tsc22d1	-0.742176734	2.536521369	2.75E-33	1.93E-30
Trabecular meshwork 1	Bpgm	-0.709999646	1.395163667	8.53E-26	3.63E-23
Trabecular meshwork 1	Ier3	-0.66591131	2.664731247	8.25E-23	2.90E-20
Trabecular meshwork 1	Spp1	-0.664377516	1.273393057	7.73E-20	1.97E-17
Trabecular meshwork 1	Mgp	-0.662904287	3.710025446	7.52E-19	1.68E-16
Trabecular meshwork 1	Gm13889	-0.633501267	1.497634589	7.84E-11	6.02E-09
Trabecular meshwork 1	Ifrd1	-0.575086978	2.850403238	7.52E-18	1.45E-15
Trabecular meshwork 1	Ctsc	-0.52645664	1.080784995	1.66E-15	2.48E-13
Trabecular meshwork 1	Csrp1	-0.521127438	1.150211866	3.35E-17	6.02E-15
Trabecular meshwork 1	Gem	-0.501288696	2.150173803	1.26E-11	1.16E-09
Trabecular meshwork 1	Itih5	-0.49574502	1.095090749	5.89E-20	1.56E-17
Trabecular meshwork 1	Gadd45b	-0.475704962	2.366046617	1.27E-12	1.32E-10
Trabecular meshwork 1	Id3	-0.45257448	3.285296665	9.01E-11	6.76E-09
Trabecular meshwork 1	Tmem140	-0.438459018	0.827976645	2.59E-13	2.95E-11
Trabecular meshwork 1	Col3a1	-0.419887011	2.216354749	8.61E-10	5.52E-08
Trabecular meshwork 1	Pim1	-0.419821206	1.473632005	9.11E-11	6.76E-09
Trabecular meshwork 1	Myl9	-0.39600073	0.69582398	7.15E-18	1.39E-15
Trabecular meshwork 1	Tnfaip6	-0.385448148	1.147138994	5.83E-07	2.15E-05
Trabecular meshwork 1	Fgl2	-0.379863446	1.578526366	2.87E-11	2.43E-09
Trabecular meshwork 1	Isg15	-0.373651796	1.709579982	2.06E-05	0.000513303
Trabecular meshwork 1	Bdnf	-0.370664008	0.425905863	1.52E-15	2.29E-13
Trabecular meshwork 1	Icam1	-0.369446165	1.653800325	1.00E-11	9.40E-10
Trabecular meshwork 1	Junb	-0.363694151	3.478369024	3.32E-09	1.96E-07
Trabecular meshwork 1	Pim3	-0.351485532	0.848057461	1.02E-10	7.52E-09
Trabecular meshwork 1	Pde10a	-0.351032061	0.383132243	4.44E-18	8.78E-16
Trabecular meshwork 1	Mat2a	-0.350606121	1.776158866	1.75E-08	9.15E-07
Trabecular meshwork 1	Sparc	-0.347461814	3.260259288	9.10E-11	6.76E-09
Trabecular meshwork 1	Ier2	-0.346087446	1.685154449	1.54E-08	8.17E-07
Trabecular meshwork 1	Ifit3	-0.339582814	1.136766304	4.39E-06	0.000132073
Trabecular meshwork 1	Jun	-0.338931382	2.063583813	0.0006151	0.008375062
Trabecular meshwork 1	Manf	-0.333421818	1.100546207	2.24E-10	1.55E-08
Trabecular meshwork 1	Lum	-0.326305199	2.209593852	4.88E-06	0.00014583
Trabecular meshwork 1	Hspa5	-0.321144358	2.776503835	1.82E-10	1.28E-08
Trabecular meshwork 1	Eva1b	-0.31333363	0.46747597	6.51E-15	8.87E-13
Trabecular meshwork 1	Mpp7	-0.310613414	0.311442221	1.27E-18	2.70E-16
Trabecular meshwork 1	Angptl4	-0.309822716	0.488797214	3.04E-10	2.08E-08
Trabecular meshwork 1	Ckb	-0.308885391	1.024586797	8.75E-08	3.84E-06
Trabecular meshwork 1	Arl4a	-0.303466062	1.073619239	1.66E-07	6.90E-06
Trabecular meshwork 1	Rgs2	-0.303270577	0.821467946	3.85E-07	1.47E-05
Trabecular meshwork 1	Col10a1	-0.301926262	0.673591694	4.66E-07	1.77E-05
Trabecular meshwork 1	Ifitm1	-0.298305208	0.946038075	1.68E-08	8.84E-07
Trabecular meshwork 1	Ogfrl1	-0.296349524	0.407449288	1.93E-11	1.71E-09
Trabecular meshwork 1	Ddx3y	-0.296112207	0.304354242	2.31E-18	4.70E-16
Trabecular meshwork 1	Errfi1	-0.293926207	2.809614989	0.0001299	0.002425197
Trabecular meshwork 1	P2ry14	-0.293527863	0.403727845	7.09E-15	9.57E-13
Trabecular meshwork 1	Tmem100	-0.291931512	0.86557096	3.37E-08	1.64E-06
Trabecular meshwork 1	Neat1	-0.289835454	0.582668391	8.87E-09	4.86E-07
Trabecular meshwork 1	Gdnf	-0.281415188	0.366687621	2.54E-11	2.19E-09
Trabecular meshwork 1	Jag1	-0.278344148	0.712131404	2.14E-08	1.09E-06
Trabecular meshwork 1	Lbp	-0.277820723	0.924405481	3.17E-07	1.24E-05
Trabecular meshwork 1	Ccl7	-0.277174269	1.095973247	0.0015672	0.017531664
Trabecular meshwork 1	Rgs16	-0.274576033	0.500748702	7.71E-08	3.43E-06
Trabecular meshwork 1	Cyp1b1	-0.272706313	1.041177383	2.23E-06	7.29E-05
Trabecular meshwork 1	Kctd1	-0.269706518	0.514476132	6.20E-11	4.89E-09
Trabecular meshwork 1	Cebpd	-0.268782932	3.604917724	8.48E-06	0.000239034
Trabecular meshwork 1	Sgk1	-0.267303771	1.559014307	0.0005144	0.007302444
Trabecular meshwork 1	Rcan1	-0.260957078	0.695291945	1.34E-06	4.60E-05
Trabecular meshwork 1	Ecm1	-0.258175358	1.112884927	2.23E-05	0.000549398
Trabecular meshwork 1	Penk	-0.25001794	0.517484069	8.35E-05	0.001677304
Trabecular meshwork 1	Eln	-0.248005394	0.595477904	1.02E-07	4.43E-06

Trabecular meshwork 1	Plekhf1	-0.24381029	0.531787422	1.36E-08	7.28E-07
Trabecular meshwork 1	Foxs1	-0.241244038	0.321514172	1.42E-10	1.02E-08
Trabecular meshwork 1	Rtp4	-0.236265882	0.768847088	3.50E-06	0.000109223
Trabecular meshwork 1	Crabp2	-0.232111975	0.636132659	0.0004058	0.00606647
Trabecular meshwork 1	Fam46a	-0.22478505	0.834870317	0.0001361	0.002530421
Trabecular meshwork 1	Spry1	-0.223615654	0.58403685	6.27E-06	0.00018186
Trabecular meshwork 1	Tmem37	-0.223040757	0.513541871	2.34E-06	7.61E-05
Trabecular meshwork 1	Ar	-0.22174259	0.657130056	2.90E-05	0.000688678
Trabecular meshwork 1	Pi16	-0.220927412	0.693968029	0.0002834	0.004536499
Trabecular meshwork 1	Arhgdib	-0.218641485	0.445512303	1.25E-07	5.33E-06
Trabecular meshwork 1	Dut	-0.217494271	0.518058223	2.68E-06	8.59E-05
Trabecular meshwork 1	Odc1	-0.208366358	1.880365801	0.0014601	0.016582749
Trabecular meshwork 1	Sat1	-0.207444702	2.070048142	0.0040709	0.037378361
Trabecular meshwork 1	Adora2b	-0.19814643	0.32043928	4.82E-08	2.25E-06
Trabecular meshwork 1	Mafb	-0.197713901	0.892486471	0.0011297	0.013446629
Trabecular meshwork 1	Lamc3	-0.197029512	0.475772927	2.76E-07	1.11E-05
Trabecular meshwork 1	Arl4d	-0.196180441	0.42694833	1.14E-05	0.000303536
Trabecular meshwork 1	Arl6ip5	-0.195806351	0.887086892	0.0003483	0.00536747
Trabecular meshwork 1	Dclk1	-0.189283577	0.456598063	7.91E-06	0.000224636
Trabecular meshwork 1	Pqlc1	-0.189050133	1.035526791	0.0029929	0.029178831
Trabecular meshwork 1	Tbx3	-0.188793742	0.536539376	0.000259	0.004212884
Trabecular meshwork 1	D10Wsu102e	-0.187256785	0.624980805	0.0005172	0.007333716
Trabecular meshwork 1	Krt12	-0.186823082	0.152732058	4.20E-16	6.62E-14
Trabecular meshwork 1	Fhl2	-0.18369642	0.930973325	0.0017579	0.019310293
Trabecular meshwork 1	Hilpda	-0.181371422	0.526965335	0.0002239	0.003782869
Trabecular meshwork 1	Nr2f1	-0.180976346	0.790929376	0.0002236	0.003782869
Trabecular meshwork 1	Fjx1	-0.17536257	0.544059377	0.0001167	0.002204344
Trabecular meshwork 1	Ncald	-0.174817118	0.307232709	8.11E-07	2.90E-05
Trabecular meshwork 1	Fndc1	-0.174307047	0.654263736	0.0001861	0.003241481
Trabecular meshwork 1	Ctxn3	-0.170193288	0.242666728	1.19E-07	5.11E-06
Trabecular meshwork 1	Ptgir	-0.168583753	0.326232346	3.40E-06	0.00010645
Trabecular meshwork 1	Fem1b	-0.167054121	0.706687052	0.0016402	0.018217044
Trabecular meshwork 1	Irf2bpl	-0.166715369	0.648637492	0.0011728	0.013871176
Trabecular meshwork 1	Bach1	-0.164616331	0.705811276	0.0014748	0.016711129
Trabecular meshwork 1	C1qtnf7	-0.1636544	0.411151365	2.80E-05	0.000666572
Trabecular meshwork 1	Rhob	-0.162529137	0.57887931	0.0026549	0.026622461
Trabecular meshwork 1	Pdgfa	-0.162203239	0.34073092	4.75E-05	0.001023973
Trabecular meshwork 1	Arrdc3	-0.161486681	1.169420882	0.0021352	0.022437061
Trabecular meshwork 1	Foxc2	-0.158982864	0.127105935	4.75E-14	5.90E-12
Trabecular meshwork 1	Wisp1	-0.158256885	0.350813559	6.08E-06	0.000177054
Trabecular meshwork 1	G0s2	-0.15813377	0.372872335	4.20E-05	0.000936967
Trabecular meshwork 1	Adh5	-0.156842552	0.2865768	6.68E-09	3.70E-07
Trabecular meshwork 1	Igf1	-0.156137083	0.55808849	0.0013033	0.015154176
Trabecular meshwork 1	Sox11	-0.154230947	0.372595958	7.69E-05	0.001566118
Trabecular meshwork 1	Clu	-0.154081199	0.531040469	0.0048361	0.042275155
Trabecular meshwork 1	Fam107b	-0.152435677	0.446750432	0.0008079	0.01041456
Trabecular meshwork 1	Clec3b	-0.152122294	0.625094884	0.0038722	0.035811375
Trabecular meshwork 1	Tmem51	-0.149931249	0.271977879	8.94E-06	0.000246941
Trabecular meshwork 1	Ndufs2	-0.149309774	0.490812998	0.0004525	0.006652403
Trabecular meshwork 1	Dot1l	-0.148667663	0.463886527	0.001239	0.014531176
Trabecular meshwork 1	Crym	-0.146708957	0.28984317	3.12E-05	0.000733574
Trabecular meshwork 1	Entpd2	-0.142887391	0.504913384	0.0041415	0.037853197
Trabecular meshwork 1	Birc3	-0.142460268	0.410309763	0.0005442	0.007625208
Trabecular meshwork 1	Vcan	-0.142272756	0.261270347	5.73E-05	0.001209307
Trabecular meshwork 1	Mpp6	-0.140818841	0.258590302	1.02E-05	0.000277589
Trabecular meshwork 1	Aqp1	-0.13967922	0.424385972	4.34E-05	0.000961219
Trabecular meshwork 1	Chp2	-0.138893901	0.180190263	2.84E-06	8.99E-05
Trabecular meshwork 1	4930523C07Rik	-0.137632594	0.584256837	0.0027016	0.026956733
Trabecular meshwork 1	Stx11	-0.135879203	0.497482833	0.0013902	0.016057671
Trabecular meshwork 1	Med24	-0.13517875	0.30095617	0.000216	0.003676355
Trabecular meshwork 1	Rtn1	-0.13086414	0.224223128	3.87E-05	0.000871529
Trabecular meshwork 1	Anxa1	-0.129501963	0.733797204	0.0035002	0.032891489
Trabecular meshwork 1	Fgf18	-0.128232071	0.32682498	0.0018877	0.020424463

Trabecular meshwork 1	Ndufa4l2	-0.126593113	0.299765718	0.0008775	0.011015349
Trabecular meshwork 1	Ptgs2os	-0.124772927	0.228054615	0.0002069	0.003550096
Trabecular meshwork 1	Mras	-0.124078645	0.391158221	0.0010662	0.012911076
Trabecular meshwork 1	Prdx6	-0.12300299	0.880699944	0.0028414	0.028012626
Trabecular meshwork 1	44443	-0.121499342	0.173005043	1.15E-05	0.000306123
Trabecular meshwork 1	Itih2	-0.121185105	0.321230372	0.0006951	0.009214309
Trabecular meshwork 1	Sod3	-0.120573838	0.281499186	0.000437	0.006471454
Trabecular meshwork 1	Pid1	-0.115622928	0.251136513	0.000442	0.006525426
Trabecular meshwork 1	D16Ertd472e	-0.115377248	0.38761346	0.0060327	0.049557118
Trabecular meshwork 1	Slc7a11	-0.112275042	0.170367661	0.0002001	0.003446599
Trabecular meshwork 1	Cxxc5	-0.110705909	0.408467092	0.0032024	0.030730296
Trabecular meshwork 1	Sostdc1	-0.110627323	0.123938411	5.47E-06	0.000161421
Trabecular meshwork 1	Cyp51	-0.109901619	0.289968718	0.0005417	0.007604996
Trabecular meshwork 1	Rasl11b	-0.109790945	0.326501644	0.0045745	0.040492992
Trabecular meshwork 1	Zbtb11	-0.108574797	0.295605012	0.0023302	0.02401849
Trabecular meshwork 1	Lpin2	-0.108331858	0.293615143	0.002611	0.026259895
Trabecular meshwork 1	Ptgis	-0.108195662	0.463418311	0.0039526	0.036459199
Trabecular meshwork 1	Crip2	-0.106344528	0.345703121	0.0026902	0.026881329
Trabecular meshwork 1	Filip1l	-0.103554103	0.219700628	0.0014088	0.016132748
Trabecular meshwork 1	Akap7	-0.103487435	0.241960187	0.0003299	0.005163441
Trabecular meshwork 1	Ehd1	-0.100997293	0.444245724	0.0023717	0.024356698
Trabecular meshwork 1	Adamts10	-0.098692463	0.317647235	0.0043434	0.039088177
Trabecular meshwork 1	Prr7	-0.095336057	0.157600826	0.0004673	0.006826807
Trabecular meshwork 1	Kcnip4	-0.094900427	0.097247546	3.71E-06	0.000114746
Trabecular meshwork 1	Hes1	-0.092552086	1.047411069	0.0027155	0.027057138
Trabecular meshwork 1	Ccrl2	-0.090869007	0.152075454	0.0014088	0.016132748
Trabecular meshwork 1	Abracl	-0.089767697	0.456193842	0.0015149	0.017123927
Trabecular meshwork 1	Pelo	-0.089734121	0.149239277	0.0005532	0.007728178
Trabecular meshwork 1	Inhba	-0.088174823	0.281810121	0.0031093	0.030125159
Trabecular meshwork 1	Igsf10	-0.086429661	0.469229455	0.0044496	0.039586544
Trabecular meshwork 1	C4b	-0.082561841	0.295541053	0.0025154	0.025515711
Trabecular meshwork 1	Srxn1	-0.081354772	0.550772715	0.0039292	0.036267225
Trabecular meshwork 1	Tcim	-0.078732481	0.227696378	0.00547	0.046373174
Trabecular meshwork 1	Crlf1	-0.078065687	0.088163193	0.0002793	0.004491482
Trabecular meshwork 1	Kcns3	-0.07803355	0.082782358	2.72E-05	0.000651945
Trabecular meshwork 1	Mkx	-0.077879335	0.145053583	0.0021594	0.022644073
Trabecular meshwork 1	Igfbp4	-0.073259661	2.934851938	0.0019132	0.020613182
Trabecular meshwork 1	Nfkbie	-0.067849093	0.09955667	0.0014633	0.016593855
Trabecular meshwork 1	Zfp467	-0.064413228	0.114667904	0.003415	0.032262829
Trabecular meshwork 1	4632427E13Rik	-0.056011625	0.126682584	0.0055924	0.047069656
Trabecular meshwork 1	Lurap1l	-0.054920991	0.07043828	0.0043647	0.039194065
Trabecular meshwork 1	Mbd6	-0.054151484	0.066557109	0.0013667	0.015818411
Trabecular meshwork 1	Brinp1	-0.053731447	0.165360567	0.0031584	0.030558901
Trabecular meshwork 1	Ring1	-0.051774618	0.119874842	0.0052018	0.044775372
Trabecular meshwork 1	Kcnk2	-0.051158347	0.160896422	0.0010966	0.013170345
Trabecular meshwork 1	Ifit1	-0.050477801	1.285700193	0.0047084	0.0413388
Trabecular meshwork 1	C2cd4b	-0.048727253	0.044211103	0.0006112	0.008362685
Trabecular meshwork 1	Htra3	-0.047184688	0.171029758	0.0041738	0.038024244
Trabecular meshwork 1	St6galnac2	-0.044013379	0.037516691	0.0006564	0.008793331
Trabecular meshwork 1	Il1r2	-0.028044102	0.027812391	0.005375	0.045883915
Trabecular meshwork 1	Cxadr	-0.016742418	0.020694392	0.0029521	0.028861899
Trabecular meshwork 1	Ctss	-0.016495368	0.009066497	0.0031699	0.030598423
Trabecular meshwork 1	Lrrc61	-0.0121394	0.006672288	0.0060673	0.04975413
Trabecular meshwork 1	Vmp1	0.008955694	0.478054936	0.0046291	0.04079564
Trabecular meshwork 1	Wisp2	0.010436445	0.022381679	0.0022645	0.023444551
Trabecular meshwork 1	Prph	0.01171778	0.008917064	0.0022387	0.023261196
Trabecular meshwork 1	Dusp1	0.012428867	1.20764672	0.004462	0.039672165
Trabecular meshwork 1	Ptgs2	0.013884695	1.828036999	0.0036051	0.033696405
Trabecular meshwork 1	Ttc36	0.017032079	0.007670584	0.0005899	0.008126792
Trabecular meshwork 1	Alpk2	0.01855921	0.009651231	0.0050958	0.044024637
Trabecular meshwork 1	Csmd1	0.02269166	0.010219439	0.0001084	0.002087489
Trabecular meshwork 1	Slc12a8	0.024319297	0.01397217	0.0052087	0.044806622
Trabecular meshwork 1	Ildr2	0.024743852	0.021078242	0.0018954	0.020469255

Trabecular meshwork 1	Tyk2	0.026245854	0.014691237	0.0026425	0.02651762
Trabecular meshwork 1	Gata3	0.026939066	0.017164956	0.0040856	0.037427996
Trabecular meshwork 1	Sox8	0.029309742	0.042584401	0.0053434	0.045741498
Trabecular meshwork 1	Rasgrf2	0.030299225	0.021019719	0.0046167	0.040739651
Trabecular meshwork 1	Gfod2	0.031475147	0.111594882	0.0008659	0.010941869
Trabecular meshwork 1	Ces1d	0.031624667	0.021252257	0.0027634	0.027404649
Trabecular meshwork 1	Nell2	0.031744605	0.050169478	0.0008969	0.011242174
Trabecular meshwork 1	Alx1	0.032047564	0.023216986	0.0025229	0.025573528
Trabecular meshwork 1	Tex14	0.032219998	0.021170337	0.0023851	0.024459236
Trabecular meshwork 1	Flt1	0.032242074	0.037081008	0.0038457	0.035683618
Trabecular meshwork 1	Fam83g	0.033167873	0.020384657	0.0009023	0.01128023
Trabecular meshwork 1	G530011O06Rik	0.033182764	1.07667577	0.0017519	0.019260348
Trabecular meshwork 1	Tmprss2	0.033504844	0.023813868	0.0013987	0.016081751
Trabecular meshwork 1	Bcat1	0.033771114	0.025700445	0.0038649	0.035791271
Trabecular meshwork 1	Plvap	0.034573927	0.050717222	0.0021966	0.022945358
Trabecular meshwork 1	Apobec1	0.037333485	0.027958525	0.0016067	0.017873451
Trabecular meshwork 1	Deptor	0.038417448	0.026650262	0.0007705	0.00997869
Trabecular meshwork 1	Cbx7	0.04147027	0.040965101	0.0022754	0.023540376
Trabecular meshwork 1	Gal	0.042268647	0.044592463	0.00319	0.030674448
Trabecular meshwork 1	Smpd3	0.043015161	0.047755702	0.0046195	0.040739651
Trabecular meshwork 1	Pank4	0.04316039	0.04420249	0.0049971	0.043331761
Trabecular meshwork 1	Gt(ROSA)26Sor	0.045013348	0.386784275	0.0059645	0.049256326
Trabecular meshwork 1	Foxj1	0.045482445	0.056077372	0.0054387	0.046190696
Trabecular meshwork 1	Tcaf2	0.046399454	0.048038531	0.0013972	0.016077781
Trabecular meshwork 1	Ccnb1ip1	0.047047902	0.024364856	6.96E-05	0.001439398
Trabecular meshwork 1	Tceal8	0.047523694	0.799736941	0.0040811	0.037427332
Trabecular meshwork 1	Pi15	0.048770603	0.052678495	0.0058043	0.048475223
Trabecular meshwork 1	Zfp46	0.05013272	0.061218608	0.0042913	0.038807291
Trabecular meshwork 1	Ccdc3	0.052428407	0.721395098	0.0057013	0.047779777
Trabecular meshwork 1	Tead3	0.052997961	0.060426645	0.0021183	0.022309842
Trabecular meshwork 1	Nanos1	0.053854158	0.055665018	0.0015231	0.017175238
Trabecular meshwork 1	Nrn1	0.055627392	0.047558414	0.0001532	0.002795274
Trabecular meshwork 1	Bhlhe22	0.056232546	0.049704661	0.0003583	0.005500353
Trabecular meshwork 1	Mbtd1	0.056764431	0.104522336	0.0059197	0.04911445
Trabecular meshwork 1	Scn7a	0.057757532	0.061810194	3.15E-05	0.000737058
Trabecular meshwork 1	Gm32031	0.060183045	0.066940088	0.0010967	0.013170345
Trabecular meshwork 1	Shisa3	0.061205791	0.056314149	0.0001898	0.003294235
Trabecular meshwork 1	Csrnp2	0.061624566	0.05964216	0.0002697	0.00436246
Trabecular meshwork 1	Camp	0.062859758	0.059431649	0.0043951	0.039350971
Trabecular meshwork 1	Gm973	0.062969444	0.0720751	0.0017672	0.019397826
Trabecular meshwork 1	Klhl15	0.063626898	0.067975548	0.0002431	0.004014644
Trabecular meshwork 1	Itga8	0.064460496	0.335463402	0.002187	0.022896216
Trabecular meshwork 1	Slc47a1	0.06505687	0.042892185	7.49E-06	0.000214667
Trabecular meshwork 1	Cfh	0.065479144	1.326132963	1.50E-11	1.36E-09
Trabecular meshwork 1	Mfn1	0.065516922	0.074196311	0.0006346	0.008558685
Trabecular meshwork 1	Trp53inp1	0.065589453	0.07517248	0.0006903	0.009177181
Trabecular meshwork 1	Kera	0.065749651	0.069778951	1.40E-06	4.79E-05
Trabecular meshwork 1	Ano1	0.067258591	0.093034762	0.0021956	0.022945358
Trabecular meshwork 1	Ntrk2	0.06770152	0.104348673	0.0003675	0.005620616
Trabecular meshwork 1	Sned1	0.067727359	0.114708033	0.0003585	0.005500353
Trabecular meshwork 1	Caprin2	0.068268406	0.113258055	0.0059252	0.04911445
Trabecular meshwork 1	Igf2bp2	0.069005471	0.082894156	0.0002811	0.004509527
Trabecular meshwork 1	Kcnj8	0.069675476	0.092534479	0.004106	0.037577673
Trabecular meshwork 1	Six2	0.071905	0.03836649	6.66E-08	3.01E-06
Trabecular meshwork 1	Angpt2	0.071960596	0.055804442	2.37E-05	0.000580218
Trabecular meshwork 1	Npr3	0.071983111	0.074167517	0.0002402	0.003985268
Trabecular meshwork 1	Edem1	0.072316392	0.146008673	0.0006483	0.008718426
Trabecular meshwork 1	Mfap5	0.072920788	1.357154714	2.28E-05	0.000560419
Trabecular meshwork 1	Fbxo32	0.073390329	0.088214037	0.0006173	0.008381855
Trabecular meshwork 1	Dkk2	0.073782618	0.408908122	7.51E-10	4.88E-08
Trabecular meshwork 1	Paip2b	0.074037623	0.11708979	0.0020494	0.021731003
Trabecular meshwork 1	Epyc	0.074559613	0.035380081	1.92E-07	7.94E-06
Trabecular meshwork 1	Zfp869	0.074622833	0.14170058	0.0009072	0.011320623

Trabecular meshwork 1	Pappa2	0.07501934	0.05362401	1.41E-05	0.000366251
Trabecular meshwork 1	Wsb1	0.075351605	0.734890843	0.0036291	0.033898468
Trabecular meshwork 1	Agl	0.076589061	0.073991372	0.0005395	0.007581503
Trabecular meshwork 1	Cebpb	0.077894453	3.365470445	0.0035358	0.033158594
Trabecular meshwork 1	Svep1	0.077899412	0.16382956	0.0001939	0.003355784
Trabecular meshwork 1	Rab26os	0.078066855	0.059893327	3.97E-06	0.000120909
Trabecular meshwork 1	Dpt	0.078633839	0.062087424	0.0001464	0.002693744
Trabecular meshwork 1	Il17ra	0.081595952	0.169318044	0.0033801	0.03206572
Trabecular meshwork 1	Parp1	0.081669369	0.118199199	0.0005613	0.007809354
Trabecular meshwork 1	Grin3a	0.081679379	0.07142648	5.78E-06	0.000169357
Trabecular meshwork 1	Hist1h1c	0.082252523	0.141921171	0.0018682	0.020268811
Trabecular meshwork 1	Smad6	0.082855437	0.113851536	0.0003333	0.005184578
Trabecular meshwork 1	Col6a5	0.083774817	0.042854903	3.93E-06	0.000120054
Trabecular meshwork 1	Atxn7	0.083988096	0.180624131	0.0038648	0.035791271
Trabecular meshwork 1	Steap4	0.084666659	0.099380174	0.0001743	0.003105172
Trabecular meshwork 1	Etv3	0.085773831	0.135906677	0.000368	0.005621223
Trabecular meshwork 1	Xdh	0.085944188	0.070130493	1.45E-06	4.95E-05
Trabecular meshwork 1	Vit	0.086543785	0.103373114	3.48E-05	0.00079402
Trabecular meshwork 1	C1ra	0.086851455	0.280417972	0.0025625	0.025881147
Trabecular meshwork 1	Tmcc3	0.087057709	0.194706987	0.0053944	0.04598191
Trabecular meshwork 1	Cd274	0.087560261	0.093528411	0.0001848	0.003237204
Trabecular meshwork 1	Plekho2	0.08988674	0.107797942	2.53E-05	0.000614618
Trabecular meshwork 1	C2	0.090775919	0.106388493	4.87E-05	0.001047634
Trabecular meshwork 1	Elovl1	0.091054342	0.210329804	0.0026883	0.026880944
Trabecular meshwork 1	Slc26a7	0.091224641	0.113240578	8.67E-05	0.001726643
Trabecular meshwork 1	Aox3	0.092050626	0.369798743	0.0054794	0.04639116
Trabecular meshwork 1	Zfp516	0.094271821	0.291897885	0.0042049	0.038258606
Trabecular meshwork 1	Hist1h2bc	0.095428666	0.465600539	0.0050593	0.043763309
Trabecular meshwork 1	Nckap5l	0.095436141	0.113068001	2.84E-05	0.000675417
Trabecular meshwork 1	Bhlhe41	0.097443588	0.239558681	0.0013943	0.016070917
Trabecular meshwork 1	Rab8b	0.098314466	0.314193108	0.0023928	0.02450269
Trabecular meshwork 1	Slc45a4	0.098535179	0.099117267	2.17E-06	7.09E-05
Trabecular meshwork 1	Lrrc32	0.099144547	0.410821573	0.0020206	0.021490118
Trabecular meshwork 1	Abcc9	0.101774531	0.059241214	7.13E-10	4.65E-08
Trabecular meshwork 1	Gcc1	0.103341879	0.578310382	0.000402	0.006027076
Trabecular meshwork 1	Foxd1	0.104228134	0.093318647	5.36E-07	2.01E-05
Trabecular meshwork 1	Adamtsl1	0.105781035	0.169799417	0.0003793	0.005744787
Trabecular meshwork 1	Smoc2	0.106252008	0.182022692	0.0010625	0.012880932
Trabecular meshwork 1	Peg10	0.106255297	0.182654418	0.0006657	0.008892672
Trabecular meshwork 1	Igfbp3	0.106820093	0.147167862	0.000534	0.007511266
Trabecular meshwork 1	Atxn2l	0.107528064	0.268595176	0.0001129	0.002149806
Trabecular meshwork 1	Arl5b	0.109906527	0.484383839	0.000363	0.005562729
Trabecular meshwork 1	S100b	0.110871143	0.254742701	0.0014155	0.016182021
Trabecular meshwork 1	B4galt5	0.111124407	0.190942157	4.66E-05	0.001011268
Trabecular meshwork 1	Prss23	0.112975786	0.312463461	0.0044277	0.03946707
Trabecular meshwork 1	Tbx15	0.113002681	0.057213778	7.62E-14	9.14E-12
Trabecular meshwork 1	Foxp2	0.113397099	0.201342932	0.0002555	0.004166234
Trabecular meshwork 1	Maf	0.113459391	0.241877518	0.0007705	0.00997869
Trabecular meshwork 1	Serpina3n	0.114684673	0.10481757	8.96E-07	3.18E-05
Trabecular meshwork 1	Hhip	0.116225662	0.122997864	2.11E-06	6.94E-05
Trabecular meshwork 1	Slc26a2	0.11689718	0.209055449	4.70E-05	0.00101512
Trabecular meshwork 1	Ncoa3	0.116912688	0.238927988	0.0003938	0.005932473
Trabecular meshwork 1	Nts	0.117756683	0.055915469	1.28E-08	6.88E-07
Trabecular meshwork 1	Gclc	0.118068393	0.238563939	0.0004634	0.006783394
Trabecular meshwork 1	Id4	0.118373251	0.238921261	0.000546	0.007642276
Trabecular meshwork 1	Gdf15	0.12032491	0.199134201	0.0007507	0.009849865
Trabecular meshwork 1	Hmgb2	0.120532213	0.419347102	0.001248	0.01461274
Trabecular meshwork 1	Trib1	0.122187763	0.682274216	7.61E-05	0.001557842
Trabecular meshwork 1	Vtn	0.122820741	0.075993119	2.87E-08	1.40E-06
Trabecular meshwork 1	Ly6a	0.123107283	0.252618189	3.18E-05	0.000742773
Trabecular meshwork 1	Rell1	0.125977522	0.299810584	0.000287	0.004588427
Trabecular meshwork 1	Pthlh	0.126134212	0.132706644	5.01E-06	0.00014931
Trabecular meshwork 1	Tspan11	0.127169685	0.282965276	0.0002761	0.00444976

Trabecular meshwork 1	Ntn4	0.128599489	0.156911862	1.16E-06	4.03E-05
Trabecular meshwork 1	Pttg1	0.129601759	0.26643134	2.69E-05	0.000648269
Trabecular meshwork 1	Gas1	0.129972049	0.516783969	0.0011687	0.013834555
Trabecular meshwork 1	Cd34	0.130801047	0.262106202	0.000178	0.003148071
Trabecular meshwork 1	Gtf2a1	0.131670886	0.180547223	1.94E-06	6.46E-05
Trabecular meshwork 1	Abca1	0.131776624	0.292412511	0.0002594	0.004214384
Trabecular meshwork 1	Col15a1	0.131883272	0.275130125	0.0011202	0.013395961
Trabecular meshwork 1	Gdf10	0.132196109	0.479874825	9.91E-05	0.001930091
Trabecular meshwork 1	Wnt11	0.132918232	0.258766038	4.81E-05	0.001036096
Trabecular meshwork 1	Igf2	0.137580247	0.663034603	0.0019245	0.020719594
Trabecular meshwork 1	Sirt1	0.138742608	0.456824392	0.0004992	0.007165758
Trabecular meshwork 1	Emb	0.140569412	0.15676446	5.49E-07	2.05E-05
Trabecular meshwork 1	Col6a3	0.142627997	0.507193415	0.0002668	0.004319937
Trabecular meshwork 1	Tcf21	0.144669608	0.107494001	6.37E-08	2.89E-06
Trabecular meshwork 1	Tpbg	0.145130349	0.667300676	0.00135	0.015650335
Trabecular meshwork 1	Olfml2b	0.145339266	0.119768259	7.02E-11	5.44E-09
Trabecular meshwork 1	Smoc1	0.145497909	0.954213061	3.76E-06	0.000115607
Trabecular meshwork 1	Usp53	0.145913189	0.382286671	0.0004657	0.006809648
Trabecular meshwork 1	Itga5	0.148393591	0.377429336	0.000908	0.011320935
Trabecular meshwork 1	5430416N02Rik	0.150833809	0.242482008	8.57E-07	3.05E-05
Trabecular meshwork 1	Nampt	0.151283415	0.547257878	0.0001144	0.002170039
Trabecular meshwork 1	Snhg9	0.151603631	0.094002315	3.12E-15	4.51E-13
Trabecular meshwork 1	Rbm27	0.152047383	0.40735534	9.49E-05	0.00185995
Trabecular meshwork 1	Dact1	0.152649672	0.704852178	0.000651	0.008746339
Trabecular meshwork 1	Map3k8	0.163007434	0.465976073	2.12E-06	6.94E-05
Trabecular meshwork 1	Pgf	0.164766917	0.803014198	5.63E-05	0.001193037
Trabecular meshwork 1	Plaur	0.165533563	0.719784333	0.0018082	0.019739341
Trabecular meshwork 1	1500015O10Rik	0.16754112	0.484747931	0.0001086	0.002087796
Trabecular meshwork 1	Thbs1	0.169559896	1.880972423	0.0005295	0.007468094
Trabecular meshwork 1	Glul	0.173219776	1.144222752	0.0005598	0.007796752
Trabecular meshwork 1	Fn1	0.174167383	0.557514889	0.0009546	0.011776169
Trabecular meshwork 1	Polr2l	0.175501955	0.318880389	7.19E-07	2.60E-05
Trabecular meshwork 1	Zfp36l2	0.176769554	1.156268012	0.0002523	0.004131372
Trabecular meshwork 1	Suco	0.177979967	0.452888831	9.82E-06	0.000268871
Trabecular meshwork 1	Thbd	0.177991582	0.413843656	0.0001795	0.003165994
Trabecular meshwork 1	Sfrp4	0.183683662	0.102602711	2.47E-11	2.14E-09
Trabecular meshwork 1	Fzd7	0.186475279	0.214585689	3.71E-11	3.10E-09
Trabecular meshwork 1	Ralbp1	0.188841358	0.650901071	7.04E-06	0.000202539
Trabecular meshwork 1	Spats2	0.192339804	0.355269951	5.91E-09	3.30E-07
Trabecular meshwork 1	Tnfsf9	0.193906804	0.373956493	1.33E-06	4.58E-05
Trabecular meshwork 1	Id1	0.196728681	0.609641126	2.55E-05	0.000619371
Trabecular meshwork 1	Slc16a1	0.202353985	0.32697246	7.54E-08	3.37E-06
Trabecular meshwork 1	Lima1	0.203097866	1.001360049	1.50E-07	6.31E-06
Trabecular meshwork 1	Hbegf	0.203122783	0.69024037	0.000178	0.003148071
Trabecular meshwork 1	Klhl21	0.219715486	0.43702856	4.16E-08	1.96E-06
Trabecular meshwork 1	Gsn	0.22086346	2.458410453	0.0015283	0.01722014
Trabecular meshwork 1	Gpx3	0.227713557	0.205019067	2.30E-11	2.02E-09
Trabecular meshwork 1	Ebf1	0.233969878	0.313236921	1.13E-08	6.08E-07
Trabecular meshwork 1	Socs3	0.248280643	1.266324573	0.0036984	0.034431238
Trabecular meshwork 1	H2-T23	0.250816383	0.457048424	6.26E-10	4.15E-08
Trabecular meshwork 1	Fst	0.253646104	0.569082643	4.37E-06	0.000131808
Trabecular meshwork 1	Thbs2	0.258742621	0.751761428	1.13E-07	4.88E-06
Trabecular meshwork 1	Akap12	0.268948084	1.043092365	1.52E-06	5.15E-05
Trabecular meshwork 1	Peg3	0.273358284	0.717202934	1.79E-09	1.12E-07
Trabecular meshwork 1	Col12a1	0.275311695	0.627812294	1.21E-08	6.48E-07
Trabecular meshwork 1	Sparcl1	0.282486797	0.37156946	1.89E-09	1.18E-07
Trabecular meshwork 1	Id2	0.287651889	0.751048509	7.00E-09	3.87E-07
Trabecular meshwork 1	Scube1	0.289804811	0.555168179	1.60E-14	2.07E-12
Trabecular meshwork 1	Cd200	0.294321653	1.15393288	4.76E-09	2.73E-07
Trabecular meshwork 1	Cstb	0.311777925	2.103753131	1.96E-06	6.52E-05
Trabecular meshwork 1	Tm4sf1	0.31863813	1.371480865	0.0004594	0.00673266
Trabecular meshwork 1	Crip1	0.323213776	1.025142226	1.55E-07	6.48E-06
Trabecular meshwork 1	Ugdh	0.330287563	1.232408396	1.85E-06	6.20E-05

Trabecular meshwork 1	Zfp36	0.337391035	1.486110218	5.44E-06	0.000160669
Trabecular meshwork 1	Hspd1	0.342554302	1.04958419	2.69E-17	4.97E-15
Trabecular meshwork 1	Has1	0.352749604	0.91608705	1.03E-08	5.61E-07
Trabecular meshwork 1	S100a6	0.358032282	2.812721256	4.98E-11	4.02E-09
Trabecular meshwork 1	Igfbp5	0.359916503	3.496901973	8.18E-12	7.76E-10
Trabecular meshwork 1	Il6	0.379189231	0.381618494	5.85E-14	7.21E-12
Trabecular meshwork 1	Col14a1	0.383733921	0.41492446	1.85E-18	3.83E-16
Trabecular meshwork 1	Hsph1	0.39061613	1.776665063	5.02E-15	6.97E-13
Trabecular meshwork 1	Hspb1	0.400874913	1.990073494	1.58E-08	8.34E-07
Trabecular meshwork 1	Irgm1	0.417329862	0.884242315	1.55E-18	3.24E-16
Trabecular meshwork 1	Hspe1	0.459047202	1.355929632	6.48E-20	1.69E-17
Trabecular meshwork 1	Hk2	0.460604534	1.170490732	3.33E-12	3.32E-10
Trabecular meshwork 1	Cst3	0.484467045	2.551824233	3.00E-20	8.26E-18
Trabecular meshwork 1	Hspa1b	0.580651925	1.639258966	3.58E-10	2.42E-08
Trabecular meshwork 1	Lars2	0.580701152	0.802019171	2.05E-33	1.51E-30
Trabecular meshwork 1	Hspa1a	0.732207168	1.746130531	5.95E-12	5.80E-10
Trabecular meshwork 1	Xist	0.772069425	0.953551433	3.17E-45	4.05E-42
Trabecular meshwork 1	Mt2	0.817167798	2.444262876	3.79E-27	1.71E-24
Trabecular meshwork 2	Tsc22d1	-0.827899297	2.532124193	6.77E-16	5.59E-13
Trabecular meshwork 2	Mfap4	-0.819682472	1.668901222	8.11E-14	4.74E-11
Trabecular meshwork 2	Spp1	-0.813523675	1.406722572	2.68E-08	5.70E-06
Trabecular meshwork 2	Mgp	-0.806539886	3.213872899	1.82E-09	5.34E-07
Trabecular meshwork 2	Col3a1	-0.806441504	1.763835561	4.90E-15	3.28E-12
Trabecular meshwork 2	Sparc	-0.804545115	2.892599525	4.05E-25	8.12E-22
Trabecular meshwork 2	Gm13889	-0.768006138	1.465865953	1.98E-06	0.000229977
Trabecular meshwork 2	Neat1	-0.670820892	0.658095418	4.28E-15	3.00E-12
Trabecular meshwork 2	Itih5	-0.634227632	1.146308815	1.39E-08	3.25E-06
Trabecular meshwork 2	Fgl2	-0.608403631	1.586287894	3.19E-09	8.61E-07
Trabecular meshwork 2	Ctsc	-0.548316519	0.868786416	2.75E-08	5.76E-06
Trabecular meshwork 2	Phlda1	-0.498619294	2.700257403	2.01E-06	0.000231097
Trabecular meshwork 2	Bpgm	-0.484259951	1.10926155	6.59E-06	0.000633993
Trabecular meshwork 2	Angptl4	-0.43943901	0.616646778	7.88E-07	0.000105348
Trabecular meshwork 2	Isg15	-0.428552505	2.586255592	0.0015766	0.04498788
Trabecular meshwork 2	Tgfb1	-0.415467243	0.671078539	3.45E-07	5.14E-05
Trabecular meshwork 2	Med24	-0.405417479	0.403880255	8.60E-09	2.16E-06
Trabecular meshwork 2	Igfbp7	-0.403877774	3.657658133	5.91E-07	8.14E-05
Trabecular meshwork 2	Tmem140	-0.396208933	1.087406327	0.0001673	0.008863428
Trabecular meshwork 2	Adora2b	-0.387756781	0.49533536	1.47E-07	2.51E-05
Trabecular meshwork 2	Ddx3y	-0.379572675	0.272466669	1.38E-12	7.47E-10
Trabecular meshwork 2	Ier3	-0.378348249	2.594426202	0.000735	0.026663216
Trabecular meshwork 2	Lbp	-0.376054214	0.659217749	1.02E-06	0.000126189
Trabecular meshwork 2	Col10a1	-0.364326075	0.722975033	0.0001979	0.010054999
Trabecular meshwork 2	Ubb	-0.336028172	3.934072062	9.60E-07	0.000122512
Trabecular meshwork 2	Dcn	-0.334823642	3.626001035	6.50E-05	0.00424356
Trabecular meshwork 2	Id3	-0.318563219	3.098320634	0.0007168	0.026276066
Trabecular meshwork 2	Ckb	-0.310480018	0.832826578	0.0007795	0.027497599
Trabecular meshwork 2	Fmo1	-0.29628913	0.899810261	0.0011618	0.036762786
Trabecular meshwork 2	Igf1	-0.282584932	0.548403165	0.0001955	0.009979223
Trabecular meshwork 2	Krt12	-0.277172087	0.166868908	7.05E-11	3.30E-08
Trabecular meshwork 2	Rgs16	-0.273216191	0.369668656	0.0007641	0.027226812
Trabecular meshwork 2	Ncald	-0.266116312	0.288265414	6.25E-06	0.000609014
Trabecular meshwork 2	Inhba	-0.260303404	0.241827686	0.0001376	0.007606598
Trabecular meshwork 2	Ftl1	-0.257755978	3.672875118	0.0003482	0.01537141
Trabecular meshwork 2	Chchd10	-0.244806155	0.308318082	0.0001704	0.008994443
Trabecular meshwork 2	Vcan	-0.241217638	0.216048144	7.15E-06	0.000682808
Trabecular meshwork 2	Chp2	-0.22588121	0.175048194	3.71E-06	0.000388865
Trabecular meshwork 2	Itih2	-0.205956323	0.345498558	0.0016205	0.045959743
Trabecular meshwork 2	Sox11	-0.169062986	0.313033216	0.001777	0.048629095
Trabecular meshwork 2	Nnmt	-0.127601155	0.149059998	0.0006299	0.024478826
Trabecular meshwork 2	Psmb10	-0.115852653	0.137266952	0.0009728	0.032058118
Trabecular meshwork 2	Scamp2	-0.113373987	0.09650462	6.40E-05	0.004200007
Trabecular meshwork 2	Aimp2	-0.096284381	0.121478971	0.0004577	0.019123756
Trabecular meshwork 2	Leng9	-0.096194409	0.077140949	0.0011089	0.035463608

Trabecular meshwork 2	Hspa4l	-0.071049622	0.128876044	0.0001468	0.007959547
Trabecular meshwork 2	Tmsb10	-0.042854691	3.039957104	0.0007532	0.027044452
Trabecular meshwork 2	Cdkn2d	-0.017506267	0.13138695	0.0005865	0.023001333
Trabecular meshwork 2	Tmsb15b2	0.048080779	0.027643759	0.0013333	0.040139234
Trabecular meshwork 2	Alx1	0.056044485	0.032222444	0.0013333	0.040139234
Trabecular meshwork 2	Nefm	0.057602836	0.033118409	0.0007284	0.026629066
Trabecular meshwork 2	D1Ertd622e	0.068799811	0.04731571	0.0010866	0.034987307
Trabecular meshwork 2	3110039I08Rik	0.069045758	0.426594188	0.0009097	0.030847536
Trabecular meshwork 2	Usp32	0.097249611	0.140055265	0.0007676	0.027283366
Trabecular meshwork 2	Nupl1	0.100622419	0.084288909	0.0017317	0.048331822
Trabecular meshwork 2	Pappa2	0.123221609	0.088412201	0.0005537	0.022084722
Trabecular meshwork 2	Lonrf1	0.128087095	0.123822479	0.0014565	0.042428289
Trabecular meshwork 2	Kirrel	0.148596354	0.121575594	1.28E-05	0.001122647
Trabecular meshwork 2	Snhg9	0.155130743	0.096792407	9.78E-08	1.81E-05
Trabecular meshwork 2	Vtn	0.161275716	0.144896422	0.0009625	0.031869175
Trabecular meshwork 2	Rnf24	0.167304176	0.294593164	0.001794	0.048905759
Trabecular meshwork 2	Ubap2	0.173944536	0.188889608	0.0002016	0.010106102
Trabecular meshwork 2	Arih1	0.179926489	0.688911116	3.54E-05	0.002574511
Trabecular meshwork 2	Tbx15	0.184909314	0.118043876	4.00E-07	5.85E-05
Trabecular meshwork 2	Cystm1	0.194287876	0.408845488	0.0013792	0.040849738
Trabecular meshwork 2	Ptgs2	0.199017157	2.101118424	1.60E-05	0.001367781
Trabecular meshwork 2	Sparcl1	0.20099548	0.59412117	0.0012087	0.037791661
Trabecular meshwork 2	Sirt1	0.204136527	0.368454764	0.0007474	0.026902919
Trabecular meshwork 2	Ch25h	0.211254849	0.514022877	0.0017077	0.047758021
Trabecular meshwork 2	Smoc1	0.216571657	0.702683793	0.0001412	0.007714239
Trabecular meshwork 2	Csrnp1	0.226814888	0.79192109	0.0007997	0.027980301
Trabecular meshwork 2	Tcf21	0.228515839	0.23700862	0.0009519	0.031690848
Trabecular meshwork 2	Rps6ka3	0.236295008	0.465339898	7.92E-05	0.004938849
Trabecular meshwork 2	Nr4a3	0.239245506	0.789199444	0.0003479	0.01537141
Trabecular meshwork 2	Slc5a3	0.243005362	0.461760262	0.0004341	0.018244972
Trabecular meshwork 2	Fbxo30	0.244279386	0.354700473	1.33E-05	0.001161087
Trabecular meshwork 2	Snhg18	0.246835798	0.356036909	3.27E-05	0.002425287
Trabecular meshwork 2	Rnd3	0.246913875	0.782633231	0.0002288	0.010962087
Trabecular meshwork 2	Spry2	0.250324581	0.770700302	0.0004004	0.017086532
Trabecular meshwork 2	Id2	0.260499236	0.513429694	0.0008022	0.027980301
Trabecular meshwork 2	Dact1	0.275252587	0.710233551	0.0011243	0.03587443
Trabecular meshwork 2	Kpna1	0.302615693	0.515818246	9.77E-06	0.000902337
Trabecular meshwork 2	Nop58	0.302618423	0.99766865	2.84E-05	0.0022037
Trabecular meshwork 2	Irs2	0.304863542	1.082806155	2.87E-06	0.000314174
Trabecular meshwork 2	Usp53	0.305015544	0.580331213	9.49E-05	0.005720727
Trabecular meshwork 2	Hbegf	0.305303877	0.582026477	0.0005846	0.02298781
Trabecular meshwork 2	Fst	0.326563086	0.511828082	4.48E-05	0.003128902
Trabecular meshwork 2	Lima1	0.327635677	1.171656564	1.69E-05	0.001422898
Trabecular meshwork 2	Glul	0.341537448	1.106270542	0.0001632	0.008713383
Trabecular meshwork 2	Timp3	0.345882273	2.467129777	0.0003367	0.015055453
Trabecular meshwork 2	Hspa1b	0.359884738	0.84647349	0.0005003	0.020421915
Trabecular meshwork 2	Akap12	0.366798126	1.369432239	0.0016305	0.046151772
Trabecular meshwork 2	Zfp36l2	0.370673726	0.678184075	5.69E-06	0.000562273
Trabecular meshwork 2	Ltbp4	0.371918741	1.150423531	1.30E-06	0.000155682
Trabecular meshwork 2	Thbs1	0.422844391	1.875325712	0.000285	0.012990785
Trabecular meshwork 2	Il6	0.430685489	0.418656453	1.21E-06	0.000146768
Trabecular meshwork 2	Arid5b	0.432560448	1.38061418	9.90E-07	0.000125228
Trabecular meshwork 2	Zfp36	0.446216069	0.867031576	4.09E-05	0.00288642
Trabecular meshwork 2	Plaur	0.455676467	1.224504031	1.18E-06	0.000144313
Trabecular meshwork 2	Mt2	0.469764879	3.175168322	2.57E-07	4.10E-05
Trabecular meshwork 2	Igfbp5	0.480874749	3.350390744	5.92E-05	0.003935604
Trabecular meshwork 2	Ctgf	0.522876081	1.270208694	8.29E-05	0.005127025
Trabecular meshwork 2	Hk2	0.548726905	1.381857857	1.21E-08	2.92E-06
Trabecular meshwork 2	Hspa1a	0.631898138	0.843726583	5.48E-08	1.10E-05
Trabecular meshwork 2	Hspb1	0.652988489	1.421667344	5.84E-08	1.14E-05
Trabecular meshwork 2	Fosb	0.666850283	1.506434005	3.39E-09	8.99E-07
Trabecular meshwork 3	Slc2a1	-0.972811857	1.481542499	1.68E-67	1.31E-64
Trabecular meshwork 3	Igfbp2	-0.948495245	2.580177278	8.51E-36	1.46E-33

Trabecular meshwork 3	Gem	-0.890939317	2.107712629	9.13E-45	2.42E-42
Trabecular meshwork 3	Ifrd1	-0.844046604	2.769932293	4.86E-51	1.70E-48
Trabecular meshwork 3	Cldn10	-0.785427535	0.98227207	9.50E-58	4.94E-55
Trabecular meshwork 3	Ier3	-0.7746442	2.683398382	1.17E-36	2.20E-34
Trabecular meshwork 3	Serpine2	-0.755418659	2.378190773	4.56E-33	6.88E-31
Trabecular meshwork 3	Sdc4	-0.734742063	2.709327229	1.60E-50	5.34E-48
Trabecular meshwork 3	Cited2	-0.678843675	0.959059852	4.92E-41	1.06E-38
Trabecular meshwork 3	Rcan1	-0.642010382	1.087455808	1.32E-43	3.36E-41
Trabecular meshwork 3	Pim1	-0.641404978	1.645036296	5.10E-38	9.95E-36
Trabecular meshwork 3	Nr4a2	-0.637720482	1.40952459	1.26E-39	2.61E-37
Trabecular meshwork 3	Hbegf	-0.63543086	1.737524756	4.07E-23	3.36E-21
Trabecular meshwork 3	Penk	-0.62288585	1.347454937	1.45E-21	1.08E-19
Trabecular meshwork 3	Vim	-0.613373602	3.379984017	1.77E-46	5.19E-44
Trabecular meshwork 3	Atf3	-0.607646352	1.354911518	5.06E-25	4.68E-23
Trabecular meshwork 3	Emb	-0.58219782	1.438395802	7.86E-31	1.05E-28
Trabecular meshwork 3	Adm	-0.540418687	0.80413928	4.86E-30	6.10E-28
Trabecular meshwork 3	Mgarp	-0.53070072	1.105092395	2.03E-30	2.66E-28
Trabecular meshwork 3	Dclk1	-0.517702774	1.233374384	2.20E-25	2.09E-23
Trabecular meshwork 3	Sat1	-0.516716104	1.961926376	7.02E-17	3.53E-15
Trabecular meshwork 3	Hspa5	-0.498455056	2.977149651	4.14E-30	5.38E-28
Trabecular meshwork 3	Gpi1	-0.489003795	0.802831849	1.74E-41	3.82E-39
Trabecular meshwork 3	Higd1a	-0.479348405	1.510031708	9.77E-25	8.85E-23
Trabecular meshwork 3	Gm13889	-0.474204615	0.979545183	5.92E-13	1.90E-11
Trabecular meshwork 3	Csf3	-0.467701408	1.469825094	1.04E-11	2.80E-10
Trabecular meshwork 3	Hilpda	-0.462027561	0.775645621	2.10E-29	2.48E-27
Trabecular meshwork 3	Nkd2	-0.461218486	0.854122503	3.52E-24	3.01E-22
Trabecular meshwork 3	Arl4d	-0.447629734	1.191042133	2.09E-18	1.21E-16
Trabecular meshwork 3	Csrp1	-0.433284309	0.887764806	8.06E-21	5.57E-19
Trabecular meshwork 3	Fjx1	-0.423251668	0.708354483	4.36E-26	4.25E-24
Trabecular meshwork 3	Junb	-0.419883445	2.233020918	1.25E-12	3.79E-11
Trabecular meshwork 3	Mat2a	-0.415529615	1.633741893	1.29E-17	6.87E-16
Trabecular meshwork 3	Tspan6	-0.411143635	0.752833572	9.36E-26	9.06E-24
Trabecular meshwork 3	Ptges	-0.406808202	1.062657784	2.42E-18	1.38E-16
Trabecular meshwork 3	Pnrc1	-0.404211976	1.402618941	2.60E-19	1.60E-17
Trabecular meshwork 3	Cryab	-0.402757022	2.171237619	2.16E-06	2.15E-05
Trabecular meshwork 3	Eln	-0.390761686	0.762852616	2.33E-19	1.45E-17
Trabecular meshwork 3	Zwint	-0.390751444	0.629435432	1.50E-29	1.78E-27
Trabecular meshwork 3	Igfbp4	-0.384244883	1.828687236	1.93E-15	8.42E-14
Trabecular meshwork 3	Id3	-0.376715299	2.66346725	5.43E-12	1.53E-10
Trabecular meshwork 3	Pim3	-0.367676942	0.507868028	1.08E-28	1.22E-26
Trabecular meshwork 3	Col8a1	-0.363375987	1.114969204	1.75E-16	8.50E-15
Trabecular meshwork 3	Krt18	-0.35897013	0.361726017	2.50E-21	1.81E-19
Trabecular meshwork 3	Neat1	-0.35598495	0.597102343	4.04E-22	3.11E-20
Trabecular meshwork 3	Fmod	-0.355332003	2.026026928	6.06E-18	3.31E-16
Trabecular meshwork 3	Ank	-0.351069505	1.217059454	5.79E-12	1.62E-10
Trabecular meshwork 3	Il11	-0.349711082	0.680856518	1.09E-10	2.54E-09
Trabecular meshwork 3	Dusp1	-0.346998682	0.942581869	8.46E-13	2.63E-11
Trabecular meshwork 3	Gja4	-0.343976851	0.434141403	5.04E-19	3.04E-17
Trabecular meshwork 3	Uaca	-0.343561745	0.739608083	3.66E-21	2.58E-19
Trabecular meshwork 3	Efhd2	-0.329303505	0.894999301	2.00E-11	5.16E-10
Trabecular meshwork 3	Pik3r1	-0.327545744	1.154376802	1.75E-14	6.63E-13
Trabecular meshwork 3	Chil1	-0.325046045	1.278663478	3.67E-14	1.34E-12
Trabecular meshwork 3	Klf6	-0.32494896	1.416362261	7.85E-12	2.15E-10
Trabecular meshwork 3	Iigp1	-0.315124068	0.529232905	6.67E-15	2.77E-13
Trabecular meshwork 3	Ctsl	-0.310765903	2.935434608	1.55E-17	8.17E-16
Trabecular meshwork 3	Jun	-0.307934289	1.473101103	1.68E-09	3.19E-08
Trabecular meshwork 3	Dusp5	-0.306249392	0.61538336	1.34E-19	8.54E-18
Trabecular meshwork 3	Nfil3	-0.301726188	0.425363882	1.09E-24	9.74E-23
Trabecular meshwork 3	Nog	-0.30142799	0.452141062	2.42E-20	1.62E-18
Trabecular meshwork 3	Myc	-0.30050718	0.867328271	2.39E-13	7.95E-12
Trabecular meshwork 3	Lmcd1	-0.296861989	0.456585735	1.91E-13	6.47E-12
Trabecular meshwork 3	Phlda1	-0.294455476	0.971329316	4.53E-09	7.93E-08
Trabecular meshwork 3	Mras	-0.293349938	0.547030119	1.05E-18	6.19E-17

Trabecular meshwork 3	Gjb3	-0.285470377	0.387900776	8.17E-19	4.86E-17
Trabecular meshwork 3	Sqstm1	-0.28180361	1.647111913	1.45E-10	3.30E-09
Trabecular meshwork 3	Hmox1	-0.277386765	2.080237591	3.45E-05	0.00024877
Trabecular meshwork 3	Dlk1	-0.267233591	0.462452735	1.62E-12	4.84E-11
Trabecular meshwork 3	Plpp1	-0.264965896	0.504765083	4.99E-18	2.76E-16
Trabecular meshwork 3	Bnip3	-0.259837489	0.45377855	2.44E-18	1.39E-16
Trabecular meshwork 3	Vps37b	-0.255133922	0.440943489	8.93E-16	4.09E-14
Trabecular meshwork 3	Metrnl	-0.25345693	0.695086701	3.40E-10	7.35E-09
Trabecular meshwork 3	Tkt	-0.251081963	0.502768401	4.27E-18	2.38E-16
Trabecular meshwork 3	Pde4b	-0.249791807	1.216595121	7.81E-09	1.30E-07
Trabecular meshwork 3	Crlf1	-0.249270767	0.438047377	7.63E-18	4.15E-16
Trabecular meshwork 3	Myl9	-0.24663533	0.593817605	6.12E-15	2.58E-13
Trabecular meshwork 3	Myoc	-0.244080863	1.432708756	0.0004306	0.002169554
Trabecular meshwork 3	Amot	-0.243637833	0.401302108	9.89E-17	4.87E-15
Trabecular meshwork 3	Plk2	-0.242040965	0.664203679	1.43E-07	1.86E-06
Trabecular meshwork 3	Adra1b	-0.241652025	0.409575233	1.54E-15	6.81E-14
Trabecular meshwork 3	Gja1	-0.238564574	0.858273237	4.07E-09	7.14E-08
Trabecular meshwork 3	Cda	-0.238481688	0.544986415	8.18E-09	1.35E-07
Trabecular meshwork 3	Zfp703	-0.237407412	0.737456053	7.37E-11	1.76E-09
Trabecular meshwork 3	Socs1	-0.236692585	0.499063237	9.01E-11	2.12E-09
Trabecular meshwork 3	Gadd45b	-0.235475187	1.633448701	5.21E-05	0.000355978
Trabecular meshwork 3	Olfm2	-0.23434224	0.403678382	3.14E-13	1.03E-11
Trabecular meshwork 3	Chst5	-0.233614323	0.173644023	9.92E-34	1.51E-31
Trabecular meshwork 3	Mast4	-0.226458191	0.575646699	8.08E-13	2.53E-11
Trabecular meshwork 3	Actb	-0.223379972	2.817193563	2.94E-09	5.30E-08
Trabecular meshwork 3	Gsta4	-0.218781689	0.402302638	1.81E-12	5.32E-11
Trabecular meshwork 3	Rgs2	-0.214999249	0.428344104	7.25E-09	1.22E-07
Trabecular meshwork 3	Sdf2l1	-0.214432443	0.291676918	2.62E-18	1.49E-16
Trabecular meshwork 3	Dynlrb1	-0.213422331	0.400558977	1.92E-17	9.98E-16
Trabecular meshwork 3	Car3	-0.213329058	0.260900279	1.31E-10	3.02E-09
Trabecular meshwork 3	Fam46a	-0.210088586	0.929583038	1.84E-05	0.000142754
Trabecular meshwork 3	Spp1	-0.208428721	0.93119024	4.46E-05	0.000310281
Trabecular meshwork 3	Gdnf	-0.207578194	0.553252995	8.51E-09	1.40E-07
Trabecular meshwork 3	Hey1	-0.206985707	0.279891666	7.31E-15	3.01E-13
Trabecular meshwork 3	Hic1	-0.206125435	0.359061024	1.36E-13	4.67E-12
Trabecular meshwork 3	Zbtb38	-0.205368252	0.417111436	1.82E-13	6.17E-12
Trabecular meshwork 3	Cdo1	-0.202289942	0.567234239	9.60E-08	1.29E-06
Trabecular meshwork 3	Palld	-0.201637636	0.888384897	1.75E-08	2.74E-07
Trabecular meshwork 3	Atp1b1	-0.200352028	0.570636297	2.56E-09	4.67E-08
Trabecular meshwork 3	Pfkfb3	-0.199859883	0.763051664	5.07E-10	1.06E-08
Trabecular meshwork 3	Tpm1	-0.199414668	1.440276597	3.35E-07	3.99E-06
Trabecular meshwork 3	Srxn1	-0.196432999	1.014337128	8.91E-07	9.69E-06
Trabecular meshwork 3	Rmst	-0.192097844	0.302910853	2.34E-14	8.76E-13
Trabecular meshwork 3	Marcksl1	-0.191733459	1.062727277	7.15E-07	7.92E-06
Trabecular meshwork 3	Chac1	-0.191251442	0.302354356	1.22E-12	3.72E-11
Trabecular meshwork 3	Ptgs2os	-0.190402152	0.278835022	1.38E-14	5.36E-13
Trabecular meshwork 3	Akap2	-0.188130469	1.088309799	1.65E-11	4.31E-10
Trabecular meshwork 3	Ahr	-0.18779204	0.278614063	6.19E-13	1.98E-11
Trabecular meshwork 3	Lgals3	-0.185935963	0.324126932	7.67E-09	1.28E-07
Trabecular meshwork 3	Ddit3	-0.185884	0.426297165	3.29E-09	5.86E-08
Trabecular meshwork 3	Vegfa	-0.185643289	1.447549121	2.35E-06	2.31E-05
Trabecular meshwork 3	Egln3	-0.185562738	0.255651989	2.27E-14	8.53E-13
Trabecular meshwork 3	Eif4e	-0.185400617	1.228821032	1.35E-05	0.000108657
Trabecular meshwork 3	C4b	-0.183076344	0.268329017	1.31E-12	3.97E-11
Trabecular meshwork 3	Ezr	-0.1825908	0.739472457	1.90E-07	2.40E-06
Trabecular meshwork 3	Nefm	-0.182558897	0.195561238	5.07E-18	2.79E-16
Trabecular meshwork 3	Hk2	-0.181231441	1.515927393	1.05E-08	1.70E-07
Trabecular meshwork 3	Gadd45a	-0.17541484	1.004212787	0.0014923	0.006118734
Trabecular meshwork 3	Csrp2	-0.175022439	1.378430385	1.27E-05	0.000102938
Trabecular meshwork 3	Maf	-0.174989628	0.589832787	1.83E-07	2.31E-06
Trabecular meshwork 3	Ddit4	-0.17317406	0.306778992	1.39E-11	3.66E-10
Trabecular meshwork 3	Kcnq1ot1	-0.172431329	0.599454219	1.21E-06	1.28E-05
Trabecular meshwork 3	Cthrc1	-0.170034639	0.623168117	2.97E-08	4.45E-07

Trabecular meshwork 3	Procr	-0.165391814	0.253939248	1.40E-08	2.23E-07
Trabecular meshwork 3	Tgfb2	-0.165117243	1.074811123	5.38E-06	4.81E-05
Trabecular meshwork 3	Mgp	-0.164876833	3.462733688	0.0011648	0.004976551
Trabecular meshwork 3	Rap2b	-0.164750694	0.338716216	2.13E-09	3.97E-08
Trabecular meshwork 3	Ifitm1	-0.163759861	0.280750022	1.44E-12	4.33E-11
Trabecular meshwork 3	Sod3	-0.162620303	0.335808852	3.33E-09	5.93E-08
Trabecular meshwork 3	Snapc1	-0.161016978	0.4608013	1.42E-09	2.71E-08
Trabecular meshwork 3	Nfkbia	-0.160946954	1.465296451	0.0063777	0.020692626
Trabecular meshwork 3	Fbxl7	-0.160879031	0.35050591	1.61E-09	3.08E-08
Trabecular meshwork 3	Gpx3	-0.160407952	0.595592559	3.88E-05	0.000274644
Trabecular meshwork 3	Ociad2	-0.159822361	0.311660353	1.26E-09	2.43E-08
Trabecular meshwork 3	Upp1	-0.158398405	0.254328376	2.27E-09	4.18E-08
Trabecular meshwork 3	Aldh3a1	-0.154777157	0.395713894	7.97E-07	8.74E-06
Trabecular meshwork 3	Msmo1	-0.153443679	0.195793154	1.21E-12	3.69E-11
Trabecular meshwork 3	Plpp3	-0.151405762	1.678426136	0.0011345	0.004869357
Trabecular meshwork 3	Hspb1	-0.15078517	2.053469994	4.51E-10	9.51E-09
Trabecular meshwork 3	Ctsc	-0.150425142	0.658651621	1.92E-06	1.93E-05
Trabecular meshwork 3	Gadd45g	-0.149912865	0.942678891	1.63E-07	2.08E-06
Trabecular meshwork 3	H2-Q4	-0.149032129	0.307127635	7.05E-10	1.42E-08
Trabecular meshwork 3	Nrip1	-0.148505735	0.756622141	8.33E-05	0.000534684
Trabecular meshwork 3	Chst2	-0.147745132	0.427543361	2.84E-06	2.74E-05
Trabecular meshwork 3	Ero1l	-0.145727224	0.442481986	1.62E-06	1.67E-05
Trabecular meshwork 3	Foxs1	-0.144714669	0.285894439	1.02E-07	1.36E-06
Trabecular meshwork 3	Rgs16	-0.144315119	0.251839218	2.32E-06	2.29E-05
Trabecular meshwork 3	Pcp4l1	-0.143099271	0.244071367	5.23E-08	7.33E-07
Trabecular meshwork 3	Basp1	-0.142662226	0.578178766	0.0002493	0.001364063
Trabecular meshwork 3	Col4a4	-0.142182954	0.274543689	8.31E-11	1.97E-09
Trabecular meshwork 3	Krt12	-0.14208108	0.162174393	1.64E-22	1.31E-20
Trabecular meshwork 3	Npy	-0.140538288	0.21153015	6.41E-07	7.18E-06
Trabecular meshwork 3	Ier2	-0.140471712	1.151224742	1.31E-08	2.11E-07
Trabecular meshwork 3	Fam110a	-0.139326436	0.145373922	5.31E-10	1.10E-08
Trabecular meshwork 3	Bcl2l11	-0.136285377	0.517010538	0.0005602	0.00269871
Trabecular meshwork 3	Ptgir	-0.135521317	0.283899794	5.12E-08	7.18E-07
Trabecular meshwork 3	Nefl	-0.135247549	0.17161668	6.50E-10	1.32E-08
Trabecular meshwork 3	Ppp1r15a	-0.135017671	1.373009986	0.0037088	0.01325203
Trabecular meshwork 3	Sgk1	-0.133729976	1.015317559	0.0094598	0.028690077
Trabecular meshwork 3	Bmyc	-0.132140239	0.317746031	2.49E-08	3.78E-07
Trabecular meshwork 3	Cxadr	-0.130252304	0.219206705	7.32E-12	2.02E-10
Trabecular meshwork 3	Ftl1	-0.129875149	3.118227566	0.0002379	0.001309527
Trabecular meshwork 3	Tagln	-0.129295068	0.85088282	7.83E-09	1.30E-07
Trabecular meshwork 3	Stx11	-0.128856794	0.408414171	2.21E-05	0.000168263
Trabecular meshwork 3	Itga3	-0.128318851	0.201532028	1.27E-11	3.38E-10
Trabecular meshwork 3	Gpha2	-0.12717266	0.425287084	0.0025327	0.009550547
Trabecular meshwork 3	Zfp503	-0.126837883	0.61234762	1.96E-05	0.000150667
Trabecular meshwork 3	Lncpint	-0.126644993	0.251992363	1.06E-07	1.40E-06
Trabecular meshwork 3	F3	-0.126363168	0.902256487	4.02E-05	0.000283501
Trabecular meshwork 3	Pxdc1	-0.124925938	0.699384324	0.0003222	0.00169624
Trabecular meshwork 3	Fam180a	-0.124537763	0.381291349	0.0007189	0.003313603
Trabecular meshwork 3	Pqlc1	-0.12170449	0.679953589	5.44E-05	0.000368912
Trabecular meshwork 3	Lox	-0.120898762	1.482152861	0.0023538	0.009004252
Trabecular meshwork 3	Acta2	-0.12019895	0.737090189	2.99E-07	3.62E-06
Trabecular meshwork 3	Plxna2	-0.118525877	0.323809416	1.22E-07	1.60E-06
Trabecular meshwork 3	Isg15	-0.117897806	0.332897436	0.0016723	0.006727098
Trabecular meshwork 3	Lif	-0.117715358	0.679824296	2.26E-05	0.000171054
Trabecular meshwork 3	Ctxn3	-0.117565674	0.162644809	7.91E-09	1.31E-07
Trabecular meshwork 3	Hs3st1	-0.11534704	0.179646673	1.26E-05	0.000102281
Trabecular meshwork 3	Slc1a5	-0.115042479	0.299803025	4.35E-09	7.61E-08
Trabecular meshwork 3	Gprc5a	-0.113839576	0.587187137	5.05E-07	5.80E-06
Trabecular meshwork 3	Maff	-0.113604089	1.45067179	0.0001775	0.001022535
Trabecular meshwork 3	Midn	-0.112808556	0.483048226	3.72E-05	0.000265291
Trabecular meshwork 3	Dusp10	-0.11238333	0.253263284	2.58E-07	3.18E-06
Trabecular meshwork 3	Acsl3	-0.110367433	0.699771123	0.0003502	0.00182415
Trabecular meshwork 3	Pou3f3	-0.109846574	0.227450779	8.58E-08	1.16E-06

Trabecular meshwork 3	Meg3	-0.109676754	0.875892099	0.0008648	0.00388741
Trabecular meshwork 3	B4gat1	-0.10960129	0.175761511	9.18E-16	4.17E-14
Trabecular meshwork 3	Zfp771	-0.109482506	0.30200577	2.29E-06	2.26E-05
Trabecular meshwork 3	Egfl6	-0.108374464	0.282496259	1.24E-10	2.87E-09
Trabecular meshwork 3	Gm525	-0.107547094	0.153693027	2.16E-08	3.30E-07
Trabecular meshwork 3	Ppp1r14c	-0.106429156	0.157308813	1.00E-11	2.70E-10
Trabecular meshwork 3	Adh5	-0.104919082	0.296520307	8.83E-10	1.75E-08
Trabecular meshwork 3	Serpine1	-0.103991334	1.00593188	3.28E-06	3.09E-05
Trabecular meshwork 3	Dnajb1	-0.103769299	0.898735172	1.11E-06	1.18E-05
Trabecular meshwork 3	Rtp4	-0.103273314	0.263827026	2.01E-06	2.02E-05
Trabecular meshwork 3	Ncald	-0.101830845	0.147027205	3.28E-10	7.10E-09
Trabecular meshwork 3	Tnfrsf11b	-0.101523678	0.489648058	4.46E-06	4.06E-05
Trabecular meshwork 3	Arl4a	-0.099733761	0.953161728	0.0028098	0.010465961
Trabecular meshwork 3	Adrb2	-0.099603825	0.109285682	4.41E-10	9.31E-09
Trabecular meshwork 3	Fhl2	-0.099271471	0.432220087	0.0002042	0.001151066
Trabecular meshwork 3	Anapc11	-0.097769938	0.406327891	0.0005673	0.002728682
Trabecular meshwork 3	Gpd2	-0.094975023	0.237180313	7.86E-05	0.000507777
Trabecular meshwork 3	Gpr4	-0.094905901	0.125781777	8.13E-09	1.35E-07
Trabecular meshwork 3	Tmem158	-0.094758506	0.284798426	0.0001924	0.0010938
Trabecular meshwork 3	Rnd1	-0.094715944	0.159367021	1.39E-05	0.000111318
Trabecular meshwork 3	Mea1	-0.093720147	0.136952654	3.44E-08	5.08E-07
Trabecular meshwork 3	Vcam1	-0.093634233	0.663193101	1.25E-05	0.000101514
Trabecular meshwork 3	Ptx3	-0.093211785	0.302365341	0.0106317	0.031569047
Trabecular meshwork 3	Ngf	-0.092141487	0.342136953	0.0045769	0.01582629
Trabecular meshwork 3	Col4a3	-0.091728136	0.207117956	1.45E-08	2.30E-07
Trabecular meshwork 3	Ogfrl1	-0.091217589	0.130056241	1.31E-07	1.72E-06
Trabecular meshwork 3	Dpep1	-0.090451638	0.25301498	0.0002516	0.001375578
Trabecular meshwork 3	Anxa1	-0.090201131	0.875962246	0.0131091	0.037421304
Trabecular meshwork 3	Aqp1	-0.08878062	1.020682291	9.40E-13	2.91E-11
Trabecular meshwork 3	Clvs1	-0.08693164	0.173507566	2.13E-10	4.74E-09
Trabecular meshwork 3	Klf2	-0.085628695	0.415066477	0.0013206	0.005529285
Trabecular meshwork 3	Kdm7a	-0.084588389	0.457903091	0.0001163	0.000710028
Trabecular meshwork 3	Ifit1	-0.083641012	0.239768447	7.14E-07	7.91E-06
Trabecular meshwork 3	Gch1	-0.083245393	0.329754849	0.0045131	0.015634787
Trabecular meshwork 3	Gpx7	-0.083060964	0.229329456	3.94E-05	0.000278259
Trabecular meshwork 3	Relt	-0.081850332	0.133950705	1.09E-05	8.98E-05
Trabecular meshwork 3	Nr4a3	-0.080860749	0.423521431	0.002749	0.010258693
Trabecular meshwork 3	Chka	-0.08044485	0.306046766	0.0002729	0.001469994
Trabecular meshwork 3	Clcf1	-0.080145921	0.286214472	0.0015764	0.006405431
Trabecular meshwork 3	Stk17b	-0.079887241	0.808421283	3.23E-07	3.87E-06
Trabecular meshwork 3	Pfn2	-0.079787717	0.286197413	1.02E-06	1.09E-05
Trabecular meshwork 3	Angptl4	-0.079712225	0.214175361	0.0008139	0.003690896
Trabecular meshwork 3	Ankrd37	-0.079457966	0.17515875	1.77E-06	1.80E-05
Trabecular meshwork 3	Dut	-0.079372233	0.260849729	4.03E-05	0.000283852
Trabecular meshwork 3	Ctnnal1	-0.077381379	0.260744979	3.98E-05	0.000280683
Trabecular meshwork 3	Rrad	-0.077241888	0.198314869	0.0002761	0.001481766
Trabecular meshwork 3	F11r	-0.077092226	0.158286509	4.60E-05	0.000318767
Trabecular meshwork 3	Cav1	-0.07692343	0.187788466	8.32E-05	0.000534356
Trabecular meshwork 3	Rasl11b	-0.07641051	0.377387102	0.0108905	0.03218779
Trabecular meshwork 3	Ptger3	-0.076053117	0.109898052	3.51E-06	3.28E-05
Trabecular meshwork 3	S1pr3	-0.075942298	0.280464067	9.95E-06	8.31E-05
Trabecular meshwork 3	Aqp5	-0.075941199	0.205439426	0.0075155	0.023720936
Trabecular meshwork 3	Il1rn	-0.075712155	0.15327272	0.0037661	0.013412461
Trabecular meshwork 3	Aox3	-0.075156179	0.170076894	4.71E-06	4.26E-05
Trabecular meshwork 3	Sgms2	-0.074427556	0.442985562	3.49E-05	0.000250891
Trabecular meshwork 3	Rab20	-0.074241839	0.261146357	0.0005852	0.002795392
Trabecular meshwork 3	Aspscr1	-0.073874486	0.133870224	7.00E-07	7.80E-06
Trabecular meshwork 3	Tmem114	-0.073429809	0.132764325	0.00011	0.000676296
Trabecular meshwork 3	Hist1h1e	-0.072369122	0.198705493	6.97E-06	6.04E-05
Trabecular meshwork 3	Yod1	-0.072047587	0.214688122	0.0014423	0.005951676
Trabecular meshwork 3	Pde10a	-0.071608125	0.118404216	2.41E-05	0.000181016
Trabecular meshwork 3	Rras2	-0.071567204	0.214570159	3.68E-05	0.000263161
Trabecular meshwork 3	Pdzd2	-0.070698831	0.201675564	0.0021368	0.008295971

Trabecular meshwork 3	Gm4841	-0.070530108	0.085262999	1.54E-06	1.60E-05
Trabecular meshwork 3	Sbno2	-0.070144901	0.354823901	1.26E-06	1.33E-05
Trabecular meshwork 3	Sap18	-0.0699728	0.837985699	0.0148666	0.041337354
Trabecular meshwork 3	Rorb	-0.069934052	0.092497436	5.68E-06	5.05E-05
Trabecular meshwork 3	Gas1	-0.069640781	0.635023641	2.44E-05	0.000182883
Trabecular meshwork 3	Cables1	-0.068904142	0.100119021	1.42E-05	0.000113286
Trabecular meshwork 3	Ssh1	-0.068606055	0.889487943	5.28E-12	1.49E-10
Trabecular meshwork 3	Fxyd5	-0.067320262	0.200788521	0.0010195	0.004475683
Trabecular meshwork 3	Snap47	-0.067308874	0.179261615	5.60E-08	7.82E-07
Trabecular meshwork 3	Osgin1	-0.066976727	0.087086342	0.0008609	0.003871136
Trabecular meshwork 3	Tubb2b	-0.066719898	0.317158641	0.0033693	0.012225654
Trabecular meshwork 3	Mia	-0.065467335	0.093132388	1.35E-05	0.000108123
Trabecular meshwork 3	Mfap4	-0.06543521	1.787593463	0.0036835	0.013181772
Trabecular meshwork 3	Bdnf	-0.065145605	0.111545966	0.0001418	0.000842837
Trabecular meshwork 3	Bex4	-0.064631984	0.124243863	1.19E-05	9.69E-05
Trabecular meshwork 3	Sod2	-0.064304033	0.802891556	2.04E-05	0.000156191
Trabecular meshwork 3	Cdc42ep3	-0.064140436	0.172892855	0.0025627	0.009650524
Trabecular meshwork 3	Npnt	-0.064073433	0.109455963	8.37E-05	0.000536941
Trabecular meshwork 3	Ptx4	-0.063417013	0.173887625	2.56E-07	3.16E-06
Trabecular meshwork 3	Sfn	-0.063148137	0.102057746	1.42E-07	1.85E-06
Trabecular meshwork 3	Cnn2	-0.06300747	0.250154813	7.93E-06	6.81E-05
Trabecular meshwork 3	Ptger4	-0.062834136	0.18415725	0.005733	0.018995728
Trabecular meshwork 3	Nrg1	-0.062118913	0.071515485	5.76E-05	0.000387379
Trabecular meshwork 3	Akr1b3	-0.062089755	0.31516598	0.0146027	0.040773192
Trabecular meshwork 3	Fam83g	-0.062015711	0.181902546	0.0002238	0.001242993
Trabecular meshwork 3	Pcsk1n	-0.061696998	0.101876412	3.35E-05	0.000242003
Trabecular meshwork 3	Tpbg	-0.061510043	0.89673784	0.0001972	0.001115544
Trabecular meshwork 3	Cd151	-0.060486143	0.183272394	0.0052181	0.017609666
Trabecular meshwork 3	Tbc1d4	-0.060450579	0.185959875	0.0007869	0.003590322
Trabecular meshwork 3	Tbc1d1	-0.060243157	0.245889459	0.0015005	0.006143398
Trabecular meshwork 3	Exoc7	-0.060216013	0.074873575	5.41E-08	7.56E-07
Trabecular meshwork 3	Klf5	-0.060190492	0.265972673	0.0035484	0.012786125
Trabecular meshwork 3	Clec14a	-0.059945574	0.080926448	7.62E-07	8.40E-06
Trabecular meshwork 3	Tpm2	-0.059457589	0.716209244	8.20E-09	1.35E-07
Trabecular meshwork 3	Col9a3	-0.05918851	0.066492194	2.01E-06	2.02E-05
Trabecular meshwork 3	Glrx	-0.059086191	0.15553946	0.0071062	0.022643825
Trabecular meshwork 3	Ptgs1	-0.058532366	0.090934617	7.01E-05	0.000458941
Trabecular meshwork 3	Irf7	-0.057969664	0.117717258	6.99E-05	0.000457872
Trabecular meshwork 3	Psmb7	-0.05795312	0.461540799	0.010678	0.031672895
Trabecular meshwork 3	Trim3	-0.057806971	0.097161593	1.74E-07	2.21E-06
Trabecular meshwork 3	Slc7a11	-0.057258705	0.155784247	0.0143428	0.040135311
Trabecular meshwork 3	Kcnk2	-0.056894537	0.129539512	0.0034734	0.012558053
Trabecular meshwork 3	Fam13c	-0.055998142	0.099654898	0.0014109	0.005837712
Trabecular meshwork 3	S100a16	-0.055929118	0.493339174	0.0022243	0.008576383
Trabecular meshwork 3	Tmem9b	-0.055405751	0.10807205	8.72E-05	0.000556065
Trabecular meshwork 3	Epcam	-0.054942762	0.098678877	0.001451	0.005977349
Trabecular meshwork 3	Dkk1	-0.054468015	0.037575053	1.31E-06	1.38E-05
Trabecular meshwork 3	Rad52	-0.053538033	0.110844685	0.0027394	0.010231126
Trabecular meshwork 3	Xpnpep1	-0.053472376	0.108254572	6.67E-05	0.000441483
Trabecular meshwork 3	Cib1	-0.053251014	0.108340609	0.0006217	0.002935768
Trabecular meshwork 3	Proser2	-0.05319854	0.1153516	0.0054146	0.018150656
Trabecular meshwork 3	Pigt	-0.052797332	0.177138063	6.06E-07	6.83E-06
Trabecular meshwork 3	Casp3	-0.052743449	0.188744686	0.0176616	0.047445616
Trabecular meshwork 3	Ooep	-0.052496473	0.102929055	0.0041284	0.014540704
Trabecular meshwork 3	Bcar3	-0.052360143	0.246317617	2.27E-05	0.00017171
Trabecular meshwork 3	Dusp4	-0.052330125	0.13907181	0.0005992	0.002849361
Trabecular meshwork 3	Erg28	-0.052319702	0.112233817	6.92E-06	6.00E-05
Trabecular meshwork 3	Smim7	-0.052170371	0.336170545	0.002058	0.008043481
Trabecular meshwork 3	Ednrb	-0.051890146	0.089425239	0.0010056	0.004420032
Trabecular meshwork 3	Mospd2	-0.051796139	0.124291115	1.17E-05	9.60E-05
Trabecular meshwork 3	Cemip	-0.051652383	0.160982809	0.0167758	0.045545563
Trabecular meshwork 3	Slc2a3	-0.05092561	0.078651543	2.47E-06	2.42E-05
Trabecular meshwork 3	Samhd1	-0.050158958	0.301132534	0.0032435	0.011849026

Trabecular meshwork 3	Pmvk	-0.049394988	0.14564785	0.001854	0.007330172
Trabecular meshwork 3	4930523C07Rik	-0.049112831	0.105798182	0.0005542	0.002672635
Trabecular meshwork 3	Tmx2	-0.049071145	0.098843009	0.0001462	0.000865406
Trabecular meshwork 3	Ucp2	-0.04877409	0.081481217	0.000433	0.002179489
Trabecular meshwork 3	Oasl2	-0.048662749	0.122449983	4.66E-05	0.000322271
Trabecular meshwork 3	Wnt4	-0.048434248	0.097650126	0.0033046	0.012043821
Trabecular meshwork 3	Chchd10	-0.048427542	0.116060909	4.76E-06	4.30E-05
Trabecular meshwork 3	Spr	-0.048214149	0.126812339	0.000806	0.003664132
Trabecular meshwork 3	Ndrg2	-0.047821402	0.125570684	0.0009132	0.004073828
Trabecular meshwork 3	Wnt6	-0.047549977	0.066496435	0.0001447	0.000857379
Trabecular meshwork 3	Mnt	-0.047285935	0.105173906	0.0029006	0.010770598
Trabecular meshwork 3	Reps1	-0.046906109	0.160490105	0.0013511	0.005636745
Trabecular meshwork 3	H2-Ke6	-0.046514002	0.106640754	0.0005431	0.002627187
Trabecular meshwork 3	Cyb5a	-0.04627012	0.711999898	1.83E-07	2.31E-06
Trabecular meshwork 3	Sema6a	-0.045714721	0.156877473	0.0004341	0.002182571
Trabecular meshwork 3	Selenoh	-0.045394223	0.195855933	0.0026031	0.009776668
Trabecular meshwork 3	Hspb6	-0.045365697	0.050661538	6.67E-05	0.000441483
Trabecular meshwork 3	St7	-0.044698823	0.080017435	9.03E-05	0.000572827
Trabecular meshwork 3	Cyp2f2	-0.044220218	0.239299946	0.0088684	0.027172434
Trabecular meshwork 3	Lgals3bp	-0.044181889	0.12840024	0.002896	0.010760718
Trabecular meshwork 3	Pantr1	-0.044158539	0.111073081	3.63E-05	0.000259684
Trabecular meshwork 3	Commd7	-0.044002807	0.154600467	0.0157281	0.04326989
Trabecular meshwork 3	Cxxc5	-0.043741721	0.20375719	0.0081254	0.02536084
Trabecular meshwork 3	Fxyd6	-0.043174187	0.639204756	4.46E-05	0.000310389
Trabecular meshwork 3	Car2	-0.041755255	0.076908093	0.0042604	0.014923102
Trabecular meshwork 3	Jag1	-0.041739746	0.298732177	0.005097	0.017269695
Trabecular meshwork 3	Slc4a11	-0.04082625	0.077888973	0.0005679	0.002730357
Trabecular meshwork 3	Ppt2	-0.040802884	0.094125489	0.0041199	0.014514316
Trabecular meshwork 3	Mum1	-0.0402443	0.062790695	0.0001184	0.000720664
Trabecular meshwork 3	N4bp2l1	-0.040192723	0.082329495	0.0044552	0.015481851
Trabecular meshwork 3	Msmp	-0.040048865	0.036227635	6.28E-06	5.52E-05
Trabecular meshwork 3	Sap30	-0.039774896	0.295902431	0.0111605	0.032812941
Trabecular meshwork 3	Mif4gd	-0.039724373	0.134574466	0.0032983	0.012028435
Trabecular meshwork 3	Nr2f1	-0.039453183	0.193039378	0.0015483	0.006311486
Trabecular meshwork 3	Wdr53	-0.039242762	0.101163472	0.0105714	0.031435498
Trabecular meshwork 3	Zswim7	-0.038935176	0.092361925	0.004751	0.016279908
Trabecular meshwork 3	Iqcb1	-0.03880827	0.126528567	0.0002541	0.001386754
Trabecular meshwork 3	Plcg2	-0.038778181	0.037084547	9.09E-05	0.00057625
Trabecular meshwork 3	Fam184a	-0.038457498	0.108174353	0.0001841	0.001054889
Trabecular meshwork 3	Mboat7	-0.038363781	0.113734325	0.0087044	0.02675755
Trabecular meshwork 3	Bex1	-0.038102871	0.167632373	0.0098258	0.029601798
Trabecular meshwork 3	Trf	-0.037752846	0.236786343	0.0007937	0.003616473
Trabecular meshwork 3	Irak2	-0.037384581	0.047316987	0.00105	0.004577813
Trabecular meshwork 3	Josd2	-0.037042054	0.101804286	0.0112404	0.033013445
Trabecular meshwork 3	Zdbf2	-0.03696723	0.157317219	0.0093453	0.028391982
Trabecular meshwork 3	Angpt2	-0.036965903	0.152426555	0.0005735	0.002750836
Trabecular meshwork 3	Pih1d1	-0.036876528	0.102787683	0.0083514	0.025916383
Trabecular meshwork 3	Mipep	-0.036782983	0.057855969	0.0034418	0.012450258
Trabecular meshwork 3	Col23a1	-0.03664235	0.208796569	2.18E-05	0.000166198
Trabecular meshwork 3	Fam214a	-0.036632632	0.147777757	0.0036031	0.012950437
Trabecular meshwork 3	Adcyap1	-0.036518607	0.029281736	0.0001091	0.000671399
Trabecular meshwork 3	Nav1	-0.036445499	0.40807903	0.0049601	0.016877022
Trabecular meshwork 3	Zcchc12	-0.036354587	0.038866168	0.000446	0.002233106
Trabecular meshwork 3	Cdk16	-0.036295128	0.161194677	0.0044119	0.015365669
Trabecular meshwork 3	Krt5	-0.035977026	0.074357347	8.60E-06	7.30E-05
Trabecular meshwork 3	Tifa	-0.03588176	0.064819059	0.0012904	0.005425604
Trabecular meshwork 3	Mustn1	-0.035736914	0.100212435	0.0009537	0.004233044
Trabecular meshwork 3	Flrt2	-0.03569917	0.32126379	0.0002183	0.00121619
Trabecular meshwork 3	Brinp1	-0.035667814	0.091269553	0.0158857	0.043617983
Trabecular meshwork 3	Msx1	-0.03481501	0.128689324	0.0002897	0.001544732
Trabecular meshwork 3	Ecm1	-0.034658453	0.510210696	4.02E-05	0.000283237
Trabecular meshwork 3	Hspb8	-0.034458516	0.394610822	0.0023768	0.009067543
Trabecular meshwork 3	Thbs1	-0.033243833	2.291060674	5.88E-10	1.21E-08

Trabecular meshwork 3	Nampt	-0.031616628	0.506038593	0.0173478	0.046826714
Trabecular meshwork 3	Gjb4	-0.031538109	0.043988362	0.0077674	0.024389635
Trabecular meshwork 3	Tcim	-0.031300778	0.223733028	0.0014176	0.005860441
Trabecular meshwork 3	Fam181b	-0.031156219	0.057459309	0.0009268	0.00412909
Trabecular meshwork 3	2700038G22Rik	-0.030964953	0.062850892	9.35E-05	0.000591417
Trabecular meshwork 3	Dync1i1	-0.030563411	0.064986249	0.0002704	0.001460039
Trabecular meshwork 3	Pank1	-0.030532371	0.053093132	0.0112588	0.033047709
Trabecular meshwork 3	Taz	-0.030221572	0.066208006	2.09E-08	3.20E-07
Trabecular meshwork 3	Ddah2	-0.030085632	0.44133369	0.0021402	0.008304564
Trabecular meshwork 3	Mdm2	-0.030060091	0.368582655	0.0002512	0.001373732
Trabecular meshwork 3	Pomgnt1	-0.029966488	0.068428631	0.0042228	0.014802447
Trabecular meshwork 3	Tada1	-0.029165216	0.035166404	0.0012955	0.005442164
Trabecular meshwork 3	Ccnf	-0.029053525	0.035531108	0.0021691	0.008409741
Trabecular meshwork 3	Magi1	-0.028795406	0.056765015	2.21E-05	0.000168263
Trabecular meshwork 3	Sgsm3	-0.02859424	0.051504765	0.0019798	0.007765904
Trabecular meshwork 3	Pcsk5	-0.028560936	0.304281743	0.0001954	0.001108356
Trabecular meshwork 3	Igsf9b	-0.028471101	0.077796213	0.0027063	0.010115632
Trabecular meshwork 3	Adck5	-0.028458547	0.032034053	0.0010467	0.004566418
Trabecular meshwork 3	Col25a1	-0.028099355	0.028422941	0.0003778	0.001946568
Trabecular meshwork 3	Gstt1	-0.028075758	0.146069189	0.0043924	0.015309132
Trabecular meshwork 3	Ptger1	-0.02788892	0.039078488	0.0043692	0.015249583
Trabecular meshwork 3	Nat6	-0.027695466	0.044227524	0.0011114	0.004786193
Trabecular meshwork 3	Rgs7bp	-0.027474604	0.104881458	0.0045906	0.015865953
Trabecular meshwork 3	Tlnrd1	-0.027030458	0.128344171	0.0154739	0.042702597
Trabecular meshwork 3	Whamm	-0.026648738	0.082879995	0.0041162	0.014504967
Trabecular meshwork 3	Pdgfa	-0.026636668	0.371646058	3.41E-06	3.19E-05
Trabecular meshwork 3	Tsc22d3	-0.026442874	0.265052879	0.0179861	0.048197587
Trabecular meshwork 3	Pigyl	-0.026415267	0.100429389	0.006495	0.021005105
Trabecular meshwork 3	Tacr1	-0.026038463	0.09023518	0.012749	0.03660177
Trabecular meshwork 3	Zfand2b	-0.026029388	0.050387628	0.0062445	0.020349655
Trabecular meshwork 3	Chst1	-0.025534003	0.070780506	0.0034814	0.012580646
Trabecular meshwork 3	H2-Q7	-0.025512353	0.029976985	0.0003149	0.001662871
Trabecular meshwork 3	Lypd2	-0.0250876	0.057054669	0.0130268	0.037224369
Trabecular meshwork 3	Coprs	-0.024947388	0.073925609	0.0025219	0.00952256
Trabecular meshwork 3	Ubac2	-0.024534942	0.087343618	3.88E-05	0.000274644
Trabecular meshwork 3	Spata7	-0.024444971	0.046602092	0.0051865	0.017524163
Trabecular meshwork 3	Rfc2	-0.02419179	0.062693748	0.0177333	0.047620152
Trabecular meshwork 3	Trafd1	-0.024148876	0.176399365	0.0041756	0.014673501
Trabecular meshwork 3	Lbp	-0.023986225	0.728300698	0.0005793	0.002771474
Trabecular meshwork 3	Sprr1a	-0.023883962	0.014352447	4.36E-05	0.000304857
Trabecular meshwork 3	Ccdc134	-0.023592821	0.050536344	0.0033842	0.012270422
Trabecular meshwork 3	Elovl7	-0.023569819	0.033499563	0.0014942	0.006122995
Trabecular meshwork 3	Lgals7	-0.023530899	0.072875819	0.0035712	0.012861997
Trabecular meshwork 3	Cenpa	-0.023519415	0.0481099	0.0004237	0.002141285
Trabecular meshwork 3	Zkscan17	-0.023294586	0.068651268	0.006946	0.022228065
Trabecular meshwork 3	Grasp	-0.023177491	0.298719197	0.0034939	0.012622384
Trabecular meshwork 3	Mical2	-0.022280002	0.13540199	0.0080208	0.025078934
Trabecular meshwork 3	C1rl	-0.02176649	0.033912446	0.0001506	0.000886934
Trabecular meshwork 3	Abcf3	-0.021069311	0.097265696	0.0095824	0.028993046
Trabecular meshwork 3	Trappc6a	-0.020994139	0.160546479	0.0055021	0.018395751
Trabecular meshwork 3	Mfsd2a	-0.020846306	0.035477209	0.0070376	0.022463308
Trabecular meshwork 3	Cry2	-0.020788154	0.099865421	0.0018523	0.007327352
Trabecular meshwork 3	Fbln7	-0.020772521	0.10400971	0.0011117	0.004786193
Trabecular meshwork 3	Dlgap1	-0.020679091	0.018132354	0.0028446	0.010584581
Trabecular meshwork 3	Tinagl1	-0.020612185	0.05559901	0.0156147	0.043000107
Trabecular meshwork 3	Wif1	-0.020053142	0.253688561	1.99E-05	0.000152979
Trabecular meshwork 3	Egr1	-0.020014631	1.392498961	0.0002113	0.001182942
Trabecular meshwork 3	Ltbp2	-0.01994806	0.510261178	1.00E-05	8.36E-05
Trabecular meshwork 3	Gstm6	-0.019932092	0.010292757	4.05E-06	3.73E-05
Trabecular meshwork 3	Sgf29	-0.019475033	0.074904959	0.0016897	0.006789082
Trabecular meshwork 3	Stk19	-0.01941122	0.336380693	0.0112882	0.033091429
Trabecular meshwork 3	C2cd4b	-0.019227059	0.015293671	0.0111944	0.032895329
Trabecular meshwork 3	Met	-0.019215267	0.111595157	0.0111094	0.032690257

Trabecular meshwork 3	Abracl	-0.019161425	0.626776454	0.0089148	0.027296473
Trabecular meshwork 3	Rprd1a	-0.018647483	0.099231366	0.0001887	0.001076931
Trabecular meshwork 3	Kcns3	-0.018599452	0.016573401	0.0131948	0.037612668
Trabecular meshwork 3	Scx	-0.018228018	0.158455493	0.0022015	0.008498984
Trabecular meshwork 3	Frg1	-0.018147632	0.169442493	0.018576	0.049401115
Trabecular meshwork 3	Erdr1	-0.018091206	1.17936319	9.02E-06	7.64E-05
Trabecular meshwork 3	Dcaf12l1	-0.017994799	0.032375329	0.0059692	0.019616417
Trabecular meshwork 3	Rnf14	-0.017977332	0.152808353	0.0050317	0.017075248
Trabecular meshwork 3	Plekhm2	-0.017942278	0.280856795	0.0006737	0.003137094
Trabecular meshwork 3	Mblac2	-0.017329104	0.017968214	0.0182218	0.048717672
Trabecular meshwork 3	Krt14	-0.016836418	0.069554939	0.0045002	0.015610987
Trabecular meshwork 3	Mpi	-0.01673878	0.012762621	0.0067113	0.021619999
Trabecular meshwork 3	Cenpl	-0.016624704	0.034480943	0.0152945	0.042334335
Trabecular meshwork 3	Rab3d	-0.016478805	0.048794319	0.0052203	0.017612979
Trabecular meshwork 3	Nkain3	-0.016465271	0.039011087	0.0152523	0.042242555
Trabecular meshwork 3	Cdk2ap2	-0.016207786	0.242640294	0.0085861	0.026485347
Trabecular meshwork 3	Nuak1	-0.015967917	0.319511441	0.0018557	0.007334416
Trabecular meshwork 3	Tmem80	-0.015382242	0.058469951	7.06E-07	7.85E-06
Trabecular meshwork 3	Calml3	-0.014802782	0.009936057	0.0093217	0.028338461
Trabecular meshwork 3	Gpr146	-0.01452689	0.037581175	0.0100256	0.030068151
Trabecular meshwork 3	Atg9a	-0.014496056	0.072178523	0.0008389	0.00378372
Trabecular meshwork 3	Ccdc163	-0.014085997	0.02471031	0.0004648	0.002309605
Trabecular meshwork 3	Wdr1	-0.013993585	0.34015059	5.57E-05	0.000376073
Trabecular meshwork 3	Arhgef19	-0.013971571	0.044214835	0.0047293	0.016222636
Trabecular meshwork 3	Tmem163	-0.01339629	0.00892018	0.0051477	0.017414061
Trabecular meshwork 3	Pitrm1	-0.013262091	0.209923643	0.0065439	0.021143807
Trabecular meshwork 3	Fgfbp1	-0.013246316	0.012477519	0.009122	0.027833985
Trabecular meshwork 3	Klf16	-0.013078222	0.124703998	0.0015546	0.006333339
Trabecular meshwork 3	Edem2	-0.01233511	0.015663806	0.0046408	0.015976367
Trabecular meshwork 3	Mapkapk3	-0.01187228	0.124620185	0.0131303	0.037474255
Trabecular meshwork 3	Ablim1	-0.01143756	0.32262839	0.0165359	0.045033571
Trabecular meshwork 3	Cyp2d22	-0.011111414	0.05142332	0.0115086	0.033611384
Trabecular meshwork 3	Cnmd	-0.010998012	0.0629809	0.0019461	0.007644654
Trabecular meshwork 3	Gpc3	-0.010809278	0.844188049	2.00E-10	4.48E-09
Trabecular meshwork 3	Dbp	-0.010762509	0.295001451	2.89E-06	2.78E-05
Trabecular meshwork 3	Med1	-0.010253953	0.16335667	0.0110985	0.032690257
Trabecular meshwork 3	Fgf9	-0.00984581	0.021694054	0.017554	0.047219834
Trabecular meshwork 3	Gbp6	-0.009504592	0.139622394	0.0065946	0.02128801
Trabecular meshwork 3	Itpkc	-0.009499635	0.315656826	0.0047126	0.016180178
Trabecular meshwork 3	Kctd11	-0.00947622	0.189784958	0.0007187	0.003313582
Trabecular meshwork 3	AF529169	-0.008734174	0.018251248	0.0046632	0.016041665
Trabecular meshwork 3	Cpn1	-0.008379023	0.009996887	0.0043156	0.015086334
Trabecular meshwork 3	Kcnk1	-0.007897927	0.044186192	0.0141225	0.03964515
Trabecular meshwork 3	Gli1	-0.007866851	0.097081905	0.0013843	0.005748167
Trabecular meshwork 3	Flt3l	-0.00747497	0.022254772	0.001295	0.005441777
Trabecular meshwork 3	Lpin2	-0.007061862	0.263396366	0.0058037	0.019198489
Trabecular meshwork 3	Fam84a	-0.006585785	0.027652924	0.0138624	0.039049479
Trabecular meshwork 3	Fgf13	-0.006489364	0.009333278	0.0068401	0.021953854
Trabecular meshwork 3	Tmprss11a	-0.006136803	0.00573877	0.0010338	0.004517214
Trabecular meshwork 3	Lmnb1	-0.005796372	0.112017513	0.0052749	0.017750336
Trabecular meshwork 3	Stac2	-0.005775895	0.018003697	0.0002952	0.001568727
Trabecular meshwork 3	Slc16a12	-0.00559812	0.006358381	0.0041979	0.014726372
Trabecular meshwork 3	Odc1	-0.005400094	1.675845535	0.0134959	0.038245643
Trabecular meshwork 3	Erc1	-0.005157519	0.326920968	9.47E-05	0.0005975
Trabecular meshwork 3	Ch25h	-0.005075846	0.09933061	0.0035492	0.012786125
Trabecular meshwork 3	Mical3	-0.003988248	0.119335472	0.0051638	0.017464536
Trabecular meshwork 3	Naprt	-0.003110407	0.030842562	0.0080084	0.02505671
Trabecular meshwork 3	Mrps23	-0.002558032	0.121120493	0.0016216	0.006558918
Trabecular meshwork 3	Serf1	-0.002449256	0.304010246	0.0036613	0.013125156
Trabecular meshwork 3	Creb1	-0.002022334	0.15099438	0.0178439	0.047880394
Trabecular meshwork 3	Mme	0.000190785	0.112501907	0.0113419	0.033221273
Trabecular meshwork 3	Epop	0.000213487	0.089481998	0.001779	0.007087595
Trabecular meshwork 3	Mrps25	0.000263609	0.207895907	0.0087209	0.026796248

Trabecular meshwork 3	Lrmda	0.00034494	0.019371118	0.0002964	0.001574353
Trabecular meshwork 3	Gpt2	0.000766305	0.077326411	0.0014874	0.006103858
Trabecular meshwork 3	Thbd	0.000966009	0.247831767	0.0059037	0.019437742
Trabecular meshwork 3	Enpp2	0.001160135	0.190338043	0.0004393	0.002205189
Trabecular meshwork 3	Churc1	0.001306307	0.288324222	0.0018775	0.007404002
Trabecular meshwork 3	Runx1t1	0.001420358	0.049415262	0.0002572	0.001399288
Trabecular meshwork 3	Homer1	0.002480844	0.324000326	0.0028311	0.01053707
Trabecular meshwork 3	Zfp141	0.002607032	0.012611864	0.0086284	0.026578467
Trabecular meshwork 3	Irx5	0.003261237	0.098375928	0.0156744	0.043153513
Trabecular meshwork 3	Lipt2	0.003296175	0.017861347	0.0159857	0.043866847
Trabecular meshwork 3	Zc3hav1	0.003624703	0.296409946	6.07E-06	5.37E-05
Trabecular meshwork 3	Sned1	0.003766699	0.085630376	0.000269	0.001454592
Trabecular meshwork 3	Ccnb1	0.004431928	0.008656862	0.0090175	0.027550971
Trabecular meshwork 3	Sms	0.004690725	0.221015655	0.0023656	0.009036957
Trabecular meshwork 3	Mex3b	0.00473414	0.285514449	0.0002693	0.001455247
Trabecular meshwork 3	Ogfod1	0.004894145	0.056968956	0.0075144	0.023720936
Trabecular meshwork 3	Ddhd1	0.007848764	0.149317205	0.0003061	0.001618575
Trabecular meshwork 3	Npy1r	0.008455004	0.004848324	0.0046023	0.015879013
Trabecular meshwork 3	Tnfsf8	0.008486814	0.004866565	0.0046023	0.015879013
Trabecular meshwork 3	Nek2	0.008498138	0.004873058	0.0046023	0.015879013
Trabecular meshwork 3	Foxl2	0.008758485	0.005022348	0.0046023	0.015879013
Trabecular meshwork 3	Zfp507	0.008835347	0.09739262	0.0168195	0.045637569
Trabecular meshwork 3	Slc30a5	0.008869745	0.392044207	0.0060295	0.01977309
Trabecular meshwork 3	Clp1	0.009644134	0.071580352	0.0070289	0.022452502
Trabecular meshwork 3	H2-DMa	0.009952036	0.034321369	0.0140249	0.039418462
Trabecular meshwork 3	Slc33a1	0.010136917	0.04257779	0.013625	0.038510423
Trabecular meshwork 3	Lgi1	0.010812969	0.008235588	0.0018139	0.007199894
Trabecular meshwork 3	Palm	0.010907953	0.114577151	0.0011546	0.004941879
Trabecular meshwork 3	Fam118a	0.011022411	0.008280001	0.0183462	0.048984917
Trabecular meshwork 3	Astn2	0.011138111	0.008054071	0.0151056	0.041880373
Trabecular meshwork 3	Pcsk2	0.011593264	0.007330668	0.0174254	0.04696399
Trabecular meshwork 3	Dnmbp	0.011646998	0.057864562	0.0128073	0.036716693
Trabecular meshwork 3	Slc23a1	0.011968116	0.008103503	0.0071398	0.022734192
Trabecular meshwork 3	Efhb	0.0119835	0.008324502	0.0112985	0.033107771
Trabecular meshwork 3	Neto2	0.012670654	0.111844729	0.0048226	0.016481237
Trabecular meshwork 3	Pbld1	0.012680866	0.010053158	0.0148168	0.041280507
Trabecular meshwork 3	Enox1	0.012877621	0.009991297	0.0112591	0.033047709
Trabecular meshwork 3	Zeb2	0.012931564	0.382911914	0.0171296	0.046300023
Trabecular meshwork 3	Emid1	0.012972347	0.046129463	0.0003482	0.001816803
Trabecular meshwork 3	Hpgd	0.013044663	0.049149053	0.0016629	0.006694905
Trabecular meshwork 3	Rspo1	0.013089728	0.03086102	0.0004005	0.002041613
Trabecular meshwork 3	Reln	0.013713994	0.009440753	0.0055611	0.018567167
Trabecular meshwork 3	Ms4a4d	0.013871065	0.021656061	0.0085076	0.026279037
Trabecular meshwork 3	Sv2b	0.014137826	0.011827596	0.0181391	0.048544396
Trabecular meshwork 3	Gbp5	0.014188658	0.009693965	0.0070314	0.022455449
Trabecular meshwork 3	Ces1d	0.014421917	0.011542345	0.012273	0.035452704
Trabecular meshwork 3	Adcyap1r1	0.014556755	0.0098928	0.0024577	0.009325249
Trabecular meshwork 3	Six4	0.014718735	0.01992348	0.0157563	0.043338956
Trabecular meshwork 3	Osr1	0.014736475	0.01371537	0.0158428	0.043525759
Trabecular meshwork 3	P2rx7	0.014790446	0.010595571	7.59E-05	0.000491799
Trabecular meshwork 3	Plp1	0.015190078	0.011500574	0.0151832	0.042059296
Trabecular meshwork 3	Mospd1	0.01583158	0.161455529	0.0079217	0.024815136
Trabecular meshwork 3	Tm2d2	0.016409713	0.529072685	0.0107823	0.031941869
Trabecular meshwork 3	Xdh	0.016530187	0.018461776	0.0117072	0.034077718
Trabecular meshwork 3	Pcdhb17	0.016616909	0.028994811	0.0066564	0.021462968
Trabecular meshwork 3	Cdk2	0.016673317	0.046857849	0.0129253	0.036986949
Trabecular meshwork 3	Psmb8	0.016689617	0.101013916	0.0048039	0.016425374
Trabecular meshwork 3	Musk	0.016763496	0.017394911	0.0047472	0.016275067
Trabecular meshwork 3	Vps45	0.016825203	0.025702459	0.0181208	0.048512274
Trabecular meshwork 3	Enpep	0.016965494	0.018796718	0.0070195	0.022437656
Trabecular meshwork 3	Rassf2	0.017111349	0.019331156	0.008587	0.026485347
Trabecular meshwork 3	Cabp1	0.017424309	0.018500887	0.0132526	0.037699133
Trabecular meshwork 3	Riox2	0.017572531	0.024042543	0.0141285	0.039646159

Trabecular meshwork 3	Sertad3	0.017583052	0.060385317	0.0004146	0.002104485
Trabecular meshwork 3	Moxd1	0.017681696	0.010707102	0.000192	0.00109256
Trabecular meshwork 3	Lrrc17	0.01829749	0.018857095	0.0169768	0.045949035
Trabecular meshwork 3	Gabra3	0.01830131	0.019357927	0.0015672	0.006375396
Trabecular meshwork 3	Zim1	0.018422167	0.017491407	0.0158942	0.043632874
Trabecular meshwork 3	Erap1	0.018561528	0.06443137	0.002205	0.008508877
Trabecular meshwork 3	Fosb	0.019171321	1.547305378	0.0004379	0.00219967
Trabecular meshwork 3	Eps8	0.019194181	0.016468925	0.0030867	0.01137696
Trabecular meshwork 3	Tmem19	0.019269884	0.03805166	0.0119049	0.034563706
Trabecular meshwork 3	Entpd1	0.019408604	0.019366697	0.0085825	0.026485347
Trabecular meshwork 3	Tnfrsf21	0.019624378	0.025960242	0.0043835	0.015285735
Trabecular meshwork 3	Tshr	0.019694184	0.016611825	0.003208	0.011758477
Trabecular meshwork 3	Npr1	0.019716939	0.018249516	0.0047459	0.016274525
Trabecular meshwork 3	Bcl2	0.019963674	0.147209999	0.0036016	0.012948138
Trabecular meshwork 3	Agtpbp1	0.020071226	0.056818565	0.0164476	0.044836484
Trabecular meshwork 3	Sema6d	0.020366216	0.023255526	0.0106554	0.031632784
Trabecular meshwork 3	H2-Aa	0.020426662	0.01607108	0.0026479	0.009915615
Trabecular meshwork 3	Efr3b	0.02062827	0.02000749	0.0046253	0.01594644
Trabecular meshwork 3	Spin4	0.020915415	0.04374785	0.0030894	0.011380662
Trabecular meshwork 3	Tppp3	0.020926343	0.132232355	3.85E-05	0.000273112
Trabecular meshwork 3	Tfap2a	0.020990956	0.013019736	9.76E-05	0.000612756
Trabecular meshwork 3	Cdkn2c	0.021272325	0.026380374	0.0184011	0.049068901
Trabecular meshwork 3	Stk39	0.021315371	0.092177088	0.0008493	0.003822932
Trabecular meshwork 3	Doc2b	0.021328524	0.018504463	0.002175	0.008428096
Trabecular meshwork 3	Abca8b	0.021397529	0.024564888	0.01359	0.038434866
Trabecular meshwork 3	C1ql3	0.021591026	0.014177276	0.0001803	0.001037002
Trabecular meshwork 3	Acss3	0.021926261	0.017074538	0.0006088	0.002886
Trabecular meshwork 3	Crym	0.022094897	0.02297756	0.0064444	0.020865681
Trabecular meshwork 3	Chsy1	0.022166571	0.083646848	0.0036572	0.013121201
Trabecular meshwork 3	Uqcc3	0.022471708	0.14401009	0.0118229	0.034343334
Trabecular meshwork 3	Cfb	0.022687452	0.021251469	0.0084382	0.026099196
Trabecular meshwork 3	Camp	0.022857482	0.020760035	0.011432	0.033422155
Trabecular meshwork 3	Ntf3	0.023037608	0.026668391	0.0131619	0.03753408
Trabecular meshwork 3	Myh11	0.023132253	0.023023659	0.0081327	0.025369079
Trabecular meshwork 3	Abcd3	0.023185111	0.08676155	0.0121038	0.0350433
Trabecular meshwork 3	Ccl19	0.023322782	0.062208205	0.0123063	0.035534468
Trabecular meshwork 3	Creb5	0.023761351	0.028142846	0.0056822	0.018849724
Trabecular meshwork 3	Col22a1	0.024267057	0.013915375	7.09E-06	6.13E-05
Trabecular meshwork 3	Col6a6	0.024293505	0.020321375	0.0043752	0.015264133
Trabecular meshwork 3	Fgf21	0.024908165	0.029836026	0.0081069	0.025308693
Trabecular meshwork 3	Hist1h2ap	0.024927626	0.06582862	0.0144397	0.040366172
Trabecular meshwork 3	Agfg2	0.025100822	0.03996647	0.0152909	0.042332556
Trabecular meshwork 3	Lurap1l	0.025211256	0.027975775	0.0006307	0.002968416
Trabecular meshwork 3	Atf1	0.025241441	0.334889052	0.00662	0.021363941
Trabecular meshwork 3	Scn7a	0.025274325	0.024004287	0.0018574	0.007338963
Trabecular meshwork 3	Abca8a	0.025466747	0.035838298	0.0178723	0.047938449
Trabecular meshwork 3	Lepr	0.025637559	0.030919404	0.0045924	0.015868416
Trabecular meshwork 3	Phf13	0.025637786	0.134551093	0.0071782	0.022840987
Trabecular meshwork 3	Mgll	0.026030091	0.115046686	0.0026938	0.010071297
Trabecular meshwork 3	Med14	0.02617265	0.116753474	0.013165	0.037535359
Trabecular meshwork 3	Alx1	0.026354853	0.017474697	9.15E-05	0.000579856
Trabecular meshwork 3	Chd3os	0.026527643	0.055218243	0.0099232	0.02985665
Trabecular meshwork 3	Has2	0.026573644	0.037860639	0.0182346	0.048733108
Trabecular meshwork 3	Mcam	0.026720173	0.040432206	0.016063	0.044027382
Trabecular meshwork 3	Gfod2	0.027049557	0.131669346	0.000532	0.002581829
Trabecular meshwork 3	Cep295nl	0.027122838	0.036863082	0.0095045	0.028794409
Trabecular meshwork 3	Fam43a	0.027222809	0.038030274	0.0064007	0.020741383
Trabecular meshwork 3	Zfp60	0.02736344	0.024232399	0.0003666	0.001897915
Trabecular meshwork 3	Rbp4	0.027529509	0.038035553	0.0073235	0.023229848
Trabecular meshwork 3	Six1	0.02769387	0.028353675	0.0005219	0.002538062
Trabecular meshwork 3	Haus8	0.028223171	0.096884323	0.0121946	0.035260106
Trabecular meshwork 3	Cbx4	0.028340907	0.154383455	1.67E-05	0.00013135
Trabecular meshwork 3	4930402H24Rik	0.028525396	0.084561312	0.0071655	0.022805904

Trabecular meshwork 3	Irgm2	0.028777877	0.060054249	0.0093159	0.028327162
Trabecular meshwork 3	M6pr	0.029200386	0.172661948	0.0134001	0.03803564
Trabecular meshwork 3	Col2a1	0.029453002	0.034345571	0.0064538	0.020891282
Trabecular meshwork 3	Gdf11	0.029842588	0.073041143	0.0048458	0.016549877
Trabecular meshwork 3	Myd88	0.029947987	0.222423171	0.0002687	0.00145387
Trabecular meshwork 3	Clec2d	0.030208181	0.33917919	6.47E-06	5.66E-05
Trabecular meshwork 3	Cxcr4	0.030348149	0.035761644	0.0022673	0.008725408
Trabecular meshwork 3	Scin	0.030388884	0.026235693	0.000211	0.001182167
Trabecular meshwork 3	Cdkn2a	0.030555791	0.019242462	0.0001573	0.000922581
Trabecular meshwork 3	Lin7a	0.030807881	0.018620119	1.73E-07	2.21E-06
Trabecular meshwork 3	Sirt1	0.030932295	0.277984446	6.34E-06	5.56E-05
Trabecular meshwork 3	Pax9	0.030967295	0.01775747	1.22E-07	1.60E-06
Trabecular meshwork 3	Kptn	0.031222023	0.053344381	0.0008022	0.003651899
Trabecular meshwork 3	Arhgef7	0.03133815	0.091446333	0.0187513	0.049801237
Trabecular meshwork 3	Stra6	0.031930863	0.044579105	0.0044374	0.015446897
Trabecular meshwork 3	Cks2	0.032102274	0.189531523	0.0013423	0.005606648
Trabecular meshwork 3	Ugdh	0.032143883	1.165039115	2.39E-08	3.64E-07
Trabecular meshwork 3	Synm	0.032204769	0.031490565	0.0002817	0.001508239
Trabecular meshwork 3	Gata2	0.032355754	0.028132155	0.000109	0.000670831
Trabecular meshwork 3	2810474O19Rik	0.032515196	0.130352781	0.0066464	0.02143567
Trabecular meshwork 3	Cd274	0.03257006	0.046396224	0.0144085	0.04028686
Trabecular meshwork 3	Rhou	0.032671507	0.068489037	0.01017	0.030429686
Trabecular meshwork 3	Atf7ip	0.032922848	0.060316042	0.0086262	0.026578467
Trabecular meshwork 3	Twist2	0.032936472	0.045222899	0.0030396	0.011223942
Trabecular meshwork 3	Sema7a	0.03326876	0.045162602	0.0029555	0.010947701
Trabecular meshwork 3	Gcc2	0.033335015	0.088052789	0.0183665	0.049010957
Trabecular meshwork 3	Apc	0.033534365	0.28338103	0.0003591	0.001866645
Trabecular meshwork 3	Extl3	0.033548134	0.140478906	0.0112717	0.03306379
Trabecular meshwork 3	Hopx	0.03356281	0.049588375	0.0035904	0.012917949
Trabecular meshwork 3	Comp	0.033729791	0.093973784	0.0174386	0.046981546
Trabecular meshwork 3	Plekhb1	0.033966783	0.023462313	3.00E-06	2.87E-05
Trabecular meshwork 3	E130102H24Rik	0.034089961	0.044649093	0.0009783	0.004321766
Trabecular meshwork 3	Rab7b	0.034477917	0.271707024	0.0014471	0.005968051
Trabecular meshwork 3	Kcnj8	0.03465225	0.026586664	0.0001162	0.000709666
Trabecular meshwork 3	Slfn5	0.034664448	0.045810574	0.0063272	0.020571393
Trabecular meshwork 3	Stim2	0.034665333	0.152611572	3.18E-05	0.000231257
Trabecular meshwork 3	C1qtnf3	0.034747902	0.034291834	0.0030379	0.011220653
Trabecular meshwork 3	Igtp	0.035059933	0.143353472	0.0003718	0.001921913
Trabecular meshwork 3	Filip1l	0.035131249	0.045222591	0.0021094	0.008201139
Trabecular meshwork 3	Yae1d1	0.035470238	0.20231019	0.0012244	0.005185318
Trabecular meshwork 3	Klf7	0.035765827	0.189757213	0.0094852	0.02875595
Trabecular meshwork 3	Ndufv3	0.036219462	0.453614941	0.0083036	0.025785003
Trabecular meshwork 3	Ackr3	0.036360669	0.107846724	0.0186348	0.04953884
Trabecular meshwork 3	Abcc9	0.036592977	0.024070277	1.35E-06	1.42E-05
Trabecular meshwork 3	Tead3	0.036696804	0.075188803	0.0073442	0.023279543
Trabecular meshwork 3	Foxj1	0.036729942	0.067899289	0.0001548	0.000909311
Trabecular meshwork 3	Ccdc62	0.036837826	0.025632489	1.31E-07	1.71E-06
Trabecular meshwork 3	Mef2c	0.037065291	0.045707705	0.0009542	0.004233108
Trabecular meshwork 3	Scara3	0.037478238	0.181201955	0.0123992	0.03575854
Trabecular meshwork 3	Pgm2l1	0.037629709	0.081362369	0.0003648	0.00189127
Trabecular meshwork 3	Edil3	0.038432836	0.067994517	0.0034677	0.012540578
Trabecular meshwork 3	Prrg3	0.038655054	0.035740739	2.25E-05	0.000170887
Trabecular meshwork 3	Hhip	0.039131608	0.068508149	0.0078684	0.024684663
Trabecular meshwork 3	Calcrl	0.039224814	0.087387554	0.0139987	0.039373756
Trabecular meshwork 3	Shox2	0.039290539	0.029126341	6.57E-07	7.36E-06
Trabecular meshwork 3	Ralgps2	0.039433605	0.048763645	2.26E-05	0.00017148
Trabecular meshwork 3	Atad2	0.039666366	0.15813011	0.0012347	0.005224155
Trabecular meshwork 3	Camkk2	0.039757317	0.109563695	0.013153	0.037516195
Trabecular meshwork 3	Dleu2	0.039834443	0.033389774	2.67E-05	0.00019805
Trabecular meshwork 3	Hs3st6	0.039857614	0.032054075	4.94E-05	0.000339597
Trabecular meshwork 3	Ubr1	0.039930496	0.10294518	0.0038503	0.013677723
Trabecular meshwork 3	Igf2bp2	0.040004678	0.150569943	0.0074266	0.023487622
Trabecular meshwork 3	Hlf	0.040261982	0.122244548	0.0007212	0.00332205

Trabecular meshwork 3	Gsc	0.040543509	0.024931869	1.06E-06	1.13E-05
Trabecular meshwork 3	Foxp2	0.040758832	0.09522822	0.0167311	0.045459142
Trabecular meshwork 3	Pcolce2	0.040772273	0.074042579	0.0148343	0.041305888
Trabecular meshwork 3	Rnf213	0.040944629	0.257916243	0.0002742	0.001474919
Trabecular meshwork 3	Cspp1	0.04109913	0.108792449	0.0061751	0.020151537
Trabecular meshwork 3	Parm1	0.04111565	0.166847052	0.0056401	0.018745437
Trabecular meshwork 3	Acap2	0.041222794	0.332640586	0.0005628	0.002710721
Trabecular meshwork 3	Gclc	0.041363556	0.288143214	0.0132506	0.037699133
Trabecular meshwork 3	Srpx	0.041530502	0.24265893	0.0119165	0.034579444
Trabecular meshwork 3	Ephb2	0.04158008	0.086861613	0.0035724	0.012862892
Trabecular meshwork 3	Slc7a2	0.041611124	0.055262353	0.0008157	0.003695382
Trabecular meshwork 3	Cir1	0.041901097	0.073474247	0.0008656	0.00388858
Trabecular meshwork 3	Ehbp1l1	0.041969222	0.124322528	0.013815	0.038953415
Trabecular meshwork 3	Alg10b	0.042010087	0.063774013	0.0004891	0.002405104
Trabecular meshwork 3	44263	0.042127743	0.064473423	0.001082	0.004683987
Trabecular meshwork 3	Zfp367	0.042147381	0.071972642	0.0021977	0.008492755
Trabecular meshwork 3	Atf7	0.042516597	0.126398239	0.0023672	0.009037936
Trabecular meshwork 3	Bmp7	0.042795583	0.030655059	3.96E-07	4.64E-06
Trabecular meshwork 3	Gbf1	0.042866634	0.176785774	0.0031803	0.01166959
Trabecular meshwork 3	Pdgfra	0.043144179	0.70310328	2.04E-05	0.000156217
Trabecular meshwork 3	Sh3pxd2b	0.043283456	0.13570312	0.0080729	0.025213818
Trabecular meshwork 3	Fbxo45	0.043401734	0.097785705	0.0024095	0.009181971
Trabecular meshwork 3	Pi15	0.043527706	0.055507813	0.0008095	0.003675475
Trabecular meshwork 3	Kcne4	0.043572169	0.121252148	0.0066672	0.021492684
Trabecular meshwork 3	2810403D21Rik	0.043686492	0.03696436	8.67E-07	9.46E-06
Trabecular meshwork 3	Kdr	0.043895896	0.042718497	9.97E-06	8.32E-05
Trabecular meshwork 3	Gprc5b	0.04407712	0.052679583	8.76E-05	0.000557921
Trabecular meshwork 3	Osr2	0.044210709	0.043916177	3.04E-05	0.000222541
Trabecular meshwork 3	Tbx15	0.044230166	0.027065875	7.61E-09	1.27E-07
Trabecular meshwork 3	Plekho2	0.044245737	0.078710673	0.0026135	0.009802387
Trabecular meshwork 3	Rspo3	0.044421639	0.114849948	0.0060368	0.019787823
Trabecular meshwork 3	Hmcn2	0.044458804	0.059926574	0.0011366	0.004875945
Trabecular meshwork 3	Acp5	0.044617378	0.056648444	0.0003017	0.001598698
Trabecular meshwork 3	Fhl1	0.044641677	0.190550553	0.0002005	0.001133083
Trabecular meshwork 3	Etv3	0.044709347	0.12077463	0.0056209	0.018704064
Trabecular meshwork 3	Hmgcs2	0.045078444	0.042540726	2.96E-06	2.83E-05
Trabecular meshwork 3	Cp	0.045082072	0.053332496	0.0001954	0.001108356
Trabecular meshwork 3	Sgpp1	0.045088686	0.073906426	0.0009638	0.004269775
Trabecular meshwork 3	Apcdd1	0.045124369	0.07142887	6.01E-05	0.000402817
Trabecular meshwork 3	Srgn	0.045163538	0.077172027	0.0065832	0.021261137
Trabecular meshwork 3	Gm973	0.045533235	0.061731181	0.000462	0.002298641
Trabecular meshwork 3	Rpia	0.045905215	0.075994722	0.0005546	0.00267364
Trabecular meshwork 3	Ptch1	0.046179467	0.125561433	0.0178841	0.047960707
Trabecular meshwork 3	3110039I08Rik	0.046222636	0.567330938	0.0001467	0.000867617
Trabecular meshwork 3	Mier3	0.046563996	0.237327902	1.15E-05	9.40E-05
Trabecular meshwork 3	Bms1	0.046974877	0.144233766	0.0174839	0.047067391
Trabecular meshwork 3	Scd1	0.047006913	0.228700999	7.74E-07	8.52E-06
Trabecular meshwork 3	Chodl	0.047009887	0.030656992	6.78E-08	9.33E-07
Trabecular meshwork 3	Gng11	0.047328525	0.381486917	2.68E-05	0.000198676
Trabecular meshwork 3	Rasl11a	0.047329642	0.114694627	0.0055847	0.018623876
Trabecular meshwork 3	Dlg4	0.047579004	0.095509808	0.0009008	0.004022502
Trabecular meshwork 3	Dcbld2	0.047648395	0.13468578	0.0062633	0.020401474
Trabecular meshwork 3	Ptpn9	0.047758626	0.138544102	0.0002528	0.001380737
Trabecular meshwork 3	Mpv17	0.047910192	0.112316533	0.0021246	0.008253317
Trabecular meshwork 3	Axin2	0.048101197	0.040726639	1.05E-07	1.39E-06
Trabecular meshwork 3	Six2	0.04838409	0.041224137	3.16E-06	2.99E-05
Trabecular meshwork 3	Gbp2	0.048395196	0.178871374	0.0163502	0.044622899
Trabecular meshwork 3	Cxcl5	0.048509585	0.05154256	8.10E-05	0.000521509
Trabecular meshwork 3	Sos2	0.048614994	0.10536889	0.001796	0.007144988
Trabecular meshwork 3	Wwp2	0.048950049	0.084234141	0.0005755	0.002757201
Trabecular meshwork 3	Kat2b	0.048966277	0.078708445	0.0005061	0.00247825
Trabecular meshwork 3	Ccdc117	0.049242431	0.105285563	0.0017725	0.007069928
Trabecular meshwork 3	Mxd4	0.049296856	0.296054379	0.0001419	0.000842837

Trabecular meshwork 3	Thy1	0.049392295	0.045009874	1.41E-05	0.000112765
Trabecular meshwork 3	Enc1	0.04963103	0.12670319	0.015067	0.041804797
Trabecular meshwork 3	Sulf1	0.049709398	0.268338964	0.0137814	0.038882108
Trabecular meshwork 3	Atxn7	0.049907308	0.160440006	0.0010199	0.004475837
Trabecular meshwork 3	Zc3h7b	0.050101337	0.131110605	0.001606	0.006509005
Trabecular meshwork 3	Mmgt1	0.0506618	0.077433776	0.0001597	0.000934281
Trabecular meshwork 3	Efnb2	0.050666523	0.101054567	0.0003765	0.00194177
Trabecular meshwork 3	Sertad4	0.051055337	0.130934674	0.0039338	0.01394952
Trabecular meshwork 3	Steap4	0.051083061	0.208894432	0.0001597	0.000934281
Trabecular meshwork 3	Smdt1	0.051334852	0.927601287	0.0131506	0.037516195
Trabecular meshwork 3	Adcy7	0.051502782	0.112613945	0.0033499	0.012183851
Trabecular meshwork 3	Irx2	0.051540286	0.054159763	5.71E-06	5.08E-05
Trabecular meshwork 3	Mgst1	0.051901043	0.22984662	0.0005406	0.002618074
Trabecular meshwork 3	Serping1	0.052176704	1.613133163	0.0012496	0.005280728
Trabecular meshwork 3	Abca1	0.052391743	0.125461884	0.0006304	0.002968073
Trabecular meshwork 3	Aard	0.0524955	0.037294032	1.58E-06	1.63E-05
Trabecular meshwork 3	Nup54	0.053119863	0.177006791	0.0020399	0.007985953
Trabecular meshwork 3	Nckap5l	0.053458774	0.097887333	0.0007266	0.003343737
Trabecular meshwork 3	Dtx3l	0.053866875	0.123824351	0.0011949	0.005078986
Trabecular meshwork 3	Dapk1	0.054250254	0.113473696	0.0001253	0.000756626
Trabecular meshwork 3	Arnt	0.054388545	0.132951847	0.0001787	0.001028545
Trabecular meshwork 3	Rai1	0.054518143	0.099889424	9.77E-05	0.000612874
Trabecular meshwork 3	Ak3	0.054977232	0.156786517	0.0084061	0.026043451
Trabecular meshwork 3	C2	0.054989387	0.04813336	1.80E-06	1.83E-05
Trabecular meshwork 3	Tmem123	0.05502372	0.118885716	0.0002109	0.00118194
Trabecular meshwork 3	Nin	0.055705153	0.067941857	9.40E-06	7.91E-05
Trabecular meshwork 3	Mest	0.055779748	0.236044152	0.0109766	0.032387619
Trabecular meshwork 3	Mid1	0.057072069	0.415343847	6.69E-05	0.000442418
Trabecular meshwork 3	Adamts5	0.057275759	0.089966501	0.0001739	0.001003422
Trabecular meshwork 3	Fam129b	0.057325156	0.444072016	0.0081867	0.025506628
Trabecular meshwork 3	Lrrc32	0.057813561	0.136528806	0.0003844	0.001976648
Trabecular meshwork 3	Cdr2	0.058380272	0.284271781	0.0005948	0.002832676
Trabecular meshwork 3	Il4ra	0.058530864	0.119475811	0.0004181	0.002117491
Trabecular meshwork 3	Tnfaip2	0.058609524	0.127741156	0.0010287	0.004500386
Trabecular meshwork 3	Dhrs3	0.058867909	0.094283506	0.0001293	0.000779109
Trabecular meshwork 3	Ep400	0.059090072	0.318777704	0.0002546	0.001388398
Trabecular meshwork 3	Wnt5a	0.059574757	0.300921594	1.12E-05	9.24E-05
Trabecular meshwork 3	Slc26a2	0.059779844	0.122857938	0.0002782	0.001491931
Trabecular meshwork 3	St3gal1	0.059798733	0.103693409	0.0001037	0.000644536
Trabecular meshwork 3	Mdn1	0.060100111	0.105191972	0.0001124	0.000687606
Trabecular meshwork 3	Por	0.060169376	0.530187594	2.90E-05	0.000214053
Trabecular meshwork 3	Fzd2	0.060449224	0.161377286	0.0013196	0.00552692
Trabecular meshwork 3	Wfdc1	0.061031847	0.18006073	0.0060097	0.019726472
Trabecular meshwork 3	Cebpa	0.061159278	0.161427143	0.0039428	0.01397805
Trabecular meshwork 3	Col6a5	0.061572047	0.041438825	1.02E-06	1.09E-05
Trabecular meshwork 3	Apopt1	0.061935342	0.154646327	0.0012855	0.005407995
Trabecular meshwork 3	Ccne1	0.061942922	0.116905697	0.0004424	0.002218783
Trabecular meshwork 3	Tnrc6b	0.061953564	0.263743442	0.0030689	0.011316939
Trabecular meshwork 3	Angptl2	0.061982668	0.253228563	0.0072438	0.023023619
Trabecular meshwork 3	Vit	0.062216861	0.101226586	1.51E-05	0.000120208
Trabecular meshwork 3	Nppc	0.062904035	0.084570872	8.62E-05	0.000550592
Trabecular meshwork 3	Id1	0.063533388	0.538654737	0.0003431	0.001792015
Trabecular meshwork 3	Adarb1	0.06380438	0.161369278	0.0001432	0.000849882
Trabecular meshwork 3	Wnt9a	0.063887193	0.060298748	1.48E-07	1.91E-06
Trabecular meshwork 3	Zfc3h1	0.064385287	0.142470083	0.0002949	0.001567459
Trabecular meshwork 3	Nrep	0.064406484	0.110209808	5.11E-05	0.000349894
Trabecular meshwork 3	Cdon	0.064457776	0.134483186	0.0003881	0.001992146
Trabecular meshwork 3	Heca	0.064710874	0.235257203	1.57E-06	1.62E-05
Trabecular meshwork 3	Hmgn5	0.064825066	0.214774243	0.0015107	0.006179559
Trabecular meshwork 3	Col5a3	0.065568647	0.11176875	0.0001562	0.000916904
Trabecular meshwork 3	Rbfox2	0.065732483	0.451877722	2.85E-09	5.15E-08
Trabecular meshwork 3	Zfp384	0.065748328	0.08379616	1.00E-06	1.08E-05
Trabecular meshwork 3	Tmem132c	0.065825485	0.111605756	5.02E-06	4.51E-05

Trabecular meshwork 3	Enpp1	0.066571887	0.157410755	0.0013195	0.00552692
Trabecular meshwork 3	Col15a1	0.066830325	0.197442187	0.0146068	0.040776416
Trabecular meshwork 3	Slc39a14	0.067008702	0.145582949	0.0001051	0.000652152
Trabecular meshwork 3	Cish	0.067042832	0.230868641	0.0012208	0.005173028
Trabecular meshwork 3	Agl	0.067343273	0.06365991	1.38E-07	1.80E-06
Trabecular meshwork 3	Slc16a10	0.067376876	0.084236641	2.43E-06	2.38E-05
Trabecular meshwork 3	Rnf103	0.067458691	0.173063706	0.0003493	0.001820934
Trabecular meshwork 3	Ell2	0.067820331	0.334564587	0.0049053	0.016710875
Trabecular meshwork 3	Igsf3	0.068446955	0.208398886	0.0002333	0.001287711
Trabecular meshwork 3	Tbx3	0.068541911	0.101606513	7.14E-06	6.16E-05
Trabecular meshwork 3	Atp8b1	0.068808058	0.085640747	2.63E-06	2.55E-05
Trabecular meshwork 3	Tspan11	0.069084635	0.111700328	6.73E-05	0.000444389
Trabecular meshwork 3	Pde12	0.069170091	0.189283939	0.0013227	0.005536618
Trabecular meshwork 3	Fbxo32	0.069473208	0.145858889	0.0006919	0.003212034
Trabecular meshwork 3	G530011O06Rik	0.069510212	0.391850932	0.0004928	0.002421566
Trabecular meshwork 3	Lgi2	0.070854665	0.14945757	0.0005866	0.002800221
Trabecular meshwork 3	Rnd3	0.07109215	0.47521416	0.0005125	0.00250015
Trabecular meshwork 3	Fam198b	0.071280536	0.132099118	0.0001332	0.000799754
Trabecular meshwork 3	Supt16	0.071643119	0.301368717	8.45E-05	0.000541225
Trabecular meshwork 3	Mir99ahg	0.072282229	0.097357033	1.01E-05	8.37E-05
Trabecular meshwork 3	Ar	0.073112488	0.235697455	0.005743	0.019020171
Trabecular meshwork 3	Itih5	0.073132351	0.355316204	0.0096424	0.029155701
Trabecular meshwork 3	Tgm2	0.073274482	0.171326479	0.0007338	0.003371067
Trabecular meshwork 3	Zdhhc8	0.073924553	0.140334583	2.95E-05	0.000217104
Trabecular meshwork 3	Add3	0.074473303	0.302094284	7.88E-07	8.66E-06
Trabecular meshwork 3	Bmp4	0.074543352	0.119412162	4.49E-05	0.00031186
Trabecular meshwork 3	Smad4	0.07478156	0.343827767	8.31E-06	7.08E-05
Trabecular meshwork 3	Fkbp5	0.074907879	0.099437001	2.98E-07	3.60E-06
Trabecular meshwork 3	Spry2	0.075058179	0.650553383	0.0016565	0.00667488
Trabecular meshwork 3	Lamb1	0.076164902	0.176735832	1.64E-05	0.00012903
Trabecular meshwork 3	Snai1	0.076419994	0.122242696	5.56E-05	0.000375533
Trabecular meshwork 3	Adam19	0.076556247	0.124322327	3.32E-06	3.12E-05
Trabecular meshwork 3	Fam102b	0.077324093	0.137337072	2.83E-07	3.44E-06
Trabecular meshwork 3	Fst	0.077421585	0.749471553	0.0044568	0.015483518
Trabecular meshwork 3	Ccl11	0.077485959	0.067152574	9.44E-06	7.94E-05
Trabecular meshwork 3	Col11a2	0.077636146	0.086954491	8.95E-06	7.58E-05
Trabecular meshwork 3	Mettl7a1	0.077783888	0.071438839	1.31E-09	2.52E-08
Trabecular meshwork 3	Crabp1	0.079130764	0.062388476	1.63E-06	1.67E-05
Trabecular meshwork 3	Pla2g7	0.079428414	0.056247537	1.06E-06	1.14E-05
Trabecular meshwork 3	Fth1	0.080368648	3.629466857	0.0061163	0.019996918
Trabecular meshwork 3	Thbs3	0.08198462	0.143540279	1.70E-06	1.74E-05
Trabecular meshwork 3	Ly6e	0.082074614	0.312341737	0.0008318	0.003757262
Trabecular meshwork 3	Ednra	0.082437012	0.17819161	9.52E-05	0.000599993
Trabecular meshwork 3	Paip2b	0.082477067	0.165824853	5.79E-06	5.15E-05
Trabecular meshwork 3	Rgcc	0.082773217	0.495604713	9.11E-06	7.70E-05
Trabecular meshwork 3	Peg10	0.083000468	0.166595231	2.66E-05	0.000197966
Trabecular meshwork 3	Foxd2os	0.08463162	0.122352593	4.34E-07	5.04E-06
Trabecular meshwork 3	Arhgap31	0.084688271	0.211536217	1.05E-05	8.68E-05
Trabecular meshwork 3	Cdkn1c	0.085550565	1.317248248	0.0007049	0.003263043
Trabecular meshwork 3	Tgfbr3	0.0860413	0.167002062	2.55E-07	3.15E-06
Trabecular meshwork 3	Nr2f2	0.08604245	0.165794925	5.30E-05	0.000360678
Trabecular meshwork 3	Sulf2	0.086455245	0.13115105	1.88E-08	2.91E-07
Trabecular meshwork 3	Hexim1	0.087004543	0.387249529	1.52E-06	1.57E-05
Trabecular meshwork 3	Clec3b	0.08975653	0.241354186	0.0024496	0.009300055
Trabecular meshwork 3	Sorcs2	0.090135765	0.159234711	5.85E-06	5.19E-05
Trabecular meshwork 3	Lsp1	0.090237971	0.393070458	5.22E-06	4.68E-05
Trabecular meshwork 3	Pdcd11	0.090377145	0.158563658	9.03E-07	9.80E-06
Trabecular meshwork 3	Il33	0.090514705	0.068452034	9.77E-11	2.29E-09
Trabecular meshwork 3	C1qtnf2	0.091171169	0.311404305	8.26E-05	0.000530695
Trabecular meshwork 3	Ndufa3	0.091284392	0.304266518	4.95E-05	0.00034048
Trabecular meshwork 3	Fzd7	0.09234389	0.208663351	1.19E-05	9.68E-05
Trabecular meshwork 3	Pi16	0.092731226	0.399619883	7.12E-05	0.000464674
Trabecular meshwork 3	Serpina3n	0.092999084	0.134023371	3.57E-06	3.34E-05

Trabecular meshwork 3	Ssc5d	0.09311525	0.106906287	3.94E-08	5.75E-07
Trabecular meshwork 3	Pgf	0.09361925	0.089333709	2.46E-08	3.73E-07
Trabecular meshwork 3	Kctd12	0.093798938	0.39551736	0.0043364	0.015151425
Trabecular meshwork 3	Tnxb	0.093948708	0.095822609	7.55E-10	1.52E-08
Trabecular meshwork 3	Rgmb	0.095161996	0.140386341	8.24E-08	1.12E-06
Trabecular meshwork 3	Ddx18	0.095483112	0.293242007	6.16E-05	0.00041196
Trabecular meshwork 3	Inmt	0.095508484	0.05853202	3.87E-10	8.26E-09
Trabecular meshwork 3	Cops9	0.096119178	0.435134009	3.19E-05	0.000232002
Trabecular meshwork 3	Ntn1	0.096247593	0.142510181	4.42E-07	5.13E-06
Trabecular meshwork 3	Anpep	0.097450401	0.146708433	1.71E-08	2.69E-07
Trabecular meshwork 3	Cyp26b1	0.098660487	0.158252767	3.49E-06	3.26E-05
Trabecular meshwork 3	B4galt5	0.100913277	0.153824154	2.78E-08	4.19E-07
Trabecular meshwork 3	Pttg1	0.104629387	0.250258617	9.16E-08	1.23E-06
Trabecular meshwork 3	S100b	0.1048779	0.131555383	1.56E-08	2.46E-07
Trabecular meshwork 3	Igfbp7	0.10490051	2.853225817	0.0003175	0.001674038
Trabecular meshwork 3	Medag	0.10592995	0.347178672	0.0002094	0.001175551
Trabecular meshwork 3	Tubb2a	0.106924629	1.02013094	0.0054048	0.018122443
Trabecular meshwork 3	Tmsb10	0.1086138	3.09612345	0.0010582	0.004605037
Trabecular meshwork 3	Egfr	0.109229554	0.240449612	1.45E-06	1.51E-05
Trabecular meshwork 3	Il6	0.111293636	0.137522233	8.60E-07	9.40E-06
Trabecular meshwork 3	Zfp36l2	0.112736182	0.851762718	1.34E-05	0.000107871
Trabecular meshwork 3	Ccnb1ip1	0.114172499	0.077878212	2.27E-14	8.53E-13
Trabecular meshwork 3	Epyc	0.114400348	0.075273627	8.87E-15	3.59E-13
Trabecular meshwork 3	Slc16a1	0.11531105	0.256919513	3.12E-07	3.74E-06
Trabecular meshwork 3	Gdf10	0.116181267	0.228645587	8.71E-07	9.50E-06
Trabecular meshwork 3	Col11a1	0.116767794	0.62692464	2.53E-06	2.47E-05
Trabecular meshwork 3	Sox9	0.116880227	0.503784724	0.0047632	0.016317956
Trabecular meshwork 3	1500015O10Rik	0.119063271	1.671060623	2.35E-06	2.32E-05
Trabecular meshwork 3	Olfml2b	0.119404676	0.175389212	5.87E-12	1.64E-10
Trabecular meshwork 3	Sema3c	0.119756743	0.176106834	6.75E-09	1.14E-07
Trabecular meshwork 3	Ccl7	0.119940218	0.355261628	0.0011617	0.004964876
Trabecular meshwork 3	Cilp	0.121347156	0.147307379	2.08E-07	2.60E-06
Trabecular meshwork 3	Hsp90aa1	0.121616876	2.733470562	8.20E-07	8.97E-06
Trabecular meshwork 3	Tnc	0.122459298	0.202975333	7.56E-07	8.34E-06
Trabecular meshwork 3	Smoc2	0.123159647	0.202069145	2.74E-07	3.35E-06
Trabecular meshwork 3	Twsg1	0.123652496	0.421449826	1.48E-07	1.92E-06
Trabecular meshwork 3	Hes1	0.123698163	0.626868515	0.0001595	0.000933992
Trabecular meshwork 3	Itpripl2	0.123931143	0.222816072	1.38E-08	2.19E-07
Trabecular meshwork 3	Crtc2	0.124163212	0.142051958	4.31E-14	1.56E-12
Trabecular meshwork 3	Igfbp3	0.124229073	0.288437955	0.0004796	0.002366887
Trabecular meshwork 3	Epha4	0.125160449	0.264444068	2.53E-07	3.12E-06
Trabecular meshwork 3	Ndrg1	0.125565889	0.551397253	4.12E-07	4.81E-06
Trabecular meshwork 3	Impad1	0.126296132	0.280123395	1.68E-10	3.83E-09
Trabecular meshwork 3	Tob1	0.126712464	0.484199659	2.01E-05	0.00015436
Trabecular meshwork 3	Cstb	0.128054686	1.723007304	0.0004779	0.002361342
Trabecular meshwork 3	Bhlhe41	0.129531729	0.364591404	8.66E-06	7.35E-05
Trabecular meshwork 3	Ier5	0.132257417	1.348061281	0.0010186	0.004473905
Trabecular meshwork 3	H2-T23	0.135122523	0.261445968	2.25E-10	4.97E-09
Trabecular meshwork 3	Klhl21	0.136989411	0.535566566	1.13E-05	9.25E-05
Trabecular meshwork 3	Igf1	0.137232061	0.72745867	3.78E-10	8.07E-09
Trabecular meshwork 3	Taf1d	0.138008288	0.454058334	3.26E-10	7.07E-09
Trabecular meshwork 3	Frmd6	0.139810347	0.439284052	1.26E-05	0.000102364
Trabecular meshwork 3	Fos	0.140321986	0.892978326	0.0013408	0.005603713
Trabecular meshwork 3	Med13	0.141752177	0.513101341	2.36E-11	6.01E-10
Trabecular meshwork 3	Dpt	0.142296429	0.106071304	1.33E-17	7.03E-16
Trabecular meshwork 3	Ptn	0.145432842	1.194403032	1.56E-06	1.62E-05
Trabecular meshwork 3	Akap12	0.147168358	0.450718455	3.86E-05	0.000273822
Trabecular meshwork 3	Fgf2	0.147247771	0.240758858	2.04E-10	4.55E-09
Trabecular meshwork 3	Ntrk2	0.147765477	0.15309237	1.06E-13	3.69E-12
Trabecular meshwork 3	Csf1	0.148241865	0.535687744	0.0001074	0.000662674
Trabecular meshwork 3	Reck	0.15137299	0.256432724	6.68E-11	1.60E-09
Trabecular meshwork 3	Txnip	0.152709718	0.173740416	8.11E-11	1.93E-09
Trabecular meshwork 3	Id2	0.153056352	0.43078935	8.18E-06	6.99E-05

Trabecular meshwork 3	Sfrp2	0.153960522	0.634125999	0.0001324	0.000795998
Trabecular meshwork 3	Angptl7	0.157993424	0.272156657	2.19E-07	2.74E-06
Trabecular meshwork 3	Ecm2	0.158376397	0.229053324	2.99E-12	8.62E-11
Trabecular meshwork 3	Has1	0.161589427	0.300681024	2.89E-08	4.35E-07
Trabecular meshwork 3	Cd34	0.162971593	0.236721143	9.88E-11	2.31E-09
Trabecular meshwork 3	Postn	0.167646894	1.043412327	2.58E-11	6.50E-10
Trabecular meshwork 3	Hmcn1	0.169849072	0.440364431	2.58E-13	8.58E-12
Trabecular meshwork 3	Myadm	0.170665632	0.181750739	3.19E-19	1.95E-17
Trabecular meshwork 3	Cxcl12	0.171129943	0.402638674	1.60E-07	2.04E-06
Trabecular meshwork 3	Crispld2	0.173680516	0.640306646	6.16E-06	5.43E-05
Trabecular meshwork 3	Socs3	0.175681899	0.468980926	6.42E-06	5.62E-05
Trabecular meshwork 3	S100a4	0.177005646	0.404141329	2.07E-08	3.18E-07
Trabecular meshwork 3	Cd200	0.182594146	0.639791191	8.29E-06	7.07E-05
Trabecular meshwork 3	Ccl2	0.183048425	0.827680796	0.007011	0.022420796
Trabecular meshwork 3	Kera	0.186677407	0.203968695	2.80E-12	8.11E-11
Trabecular meshwork 3	Btg2	0.187726276	0.675753472	5.27E-09	9.08E-08
Trabecular meshwork 3	Gsn	0.19031822	2.018394983	6.16E-12	1.72E-10
Trabecular meshwork 3	Hist1h2bc	0.194015838	0.333294633	1.36E-12	4.12E-11
Trabecular meshwork 3	Sparcl1	0.195069506	0.266590003	3.04E-13	9.97E-12
Trabecular meshwork 3	Nupr1	0.196623448	1.515799239	0.0004241	0.002141639
Trabecular meshwork 3	Zfp36l1	0.19687371	1.156117171	2.81E-10	6.13E-09
Trabecular meshwork 3	Glul	0.198971798	0.83607001	6.69E-12	1.86E-10
Trabecular meshwork 3	Fgl2	0.211674521	0.508514544	1.67E-07	2.13E-06
Trabecular meshwork 3	Irgm1	0.213318786	0.333528469	4.45E-15	1.90E-13
Trabecular meshwork 3	H19	0.217697156	0.311162273	2.98E-13	9.79E-12
Trabecular meshwork 3	Aspn	0.218809226	0.413196472	5.13E-10	1.06E-08
Trabecular meshwork 3	Gfpt2	0.218902013	0.457479057	1.30E-10	3.01E-09
Trabecular meshwork 3	Cygb	0.220157763	0.410213713	4.92E-10	1.03E-08
Trabecular meshwork 3	Rnase4	0.221288145	0.51675037	3.35E-16	1.58E-14
Trabecular meshwork 3	Mmp3	0.223793091	0.328159744	3.14E-10	6.82E-09
Trabecular meshwork 3	Cilp2	0.224132545	0.383943891	7.13E-10	1.44E-08
Trabecular meshwork 3	Cfh	0.237077196	0.474104392	1.24E-14	4.84E-13
Trabecular meshwork 3	Zfp36	0.240658525	0.715305572	2.70E-09	4.90E-08
Trabecular meshwork 3	Ppp1r2	0.241986829	1.2493444	3.09E-09	5.55E-08
Trabecular meshwork 3	Cst3	0.242834646	2.700771269	5.54E-10	1.14E-08
Trabecular meshwork 3	Angptl1	0.245038888	0.235349115	1.20E-21	8.98E-20
Trabecular meshwork 3	Col16a1	0.252524952	1.155562782	2.06E-12	6.03E-11
Trabecular meshwork 3	Pthlh	0.257026581	0.22997511	1.65E-22	1.32E-20
Trabecular meshwork 3	Adamts1	0.277281488	0.689861358	2.05E-11	5.27E-10
Trabecular meshwork 3	Apod	0.292623066	1.662117774	4.33E-17	2.21E-15
Trabecular meshwork 3	Tgfbi	0.315757298	0.501703783	1.83E-20	1.23E-18
Trabecular meshwork 3	Sfrp4	0.319883114	0.190496846	1.20E-36	2.22E-34
Trabecular meshwork 3	Clic4	0.323359305	0.78384678	7.51E-24	6.31E-22
Trabecular meshwork 3	Col6a2	0.325151476	0.97398415	2.85E-18	1.61E-16
Trabecular meshwork 3	Igf2	0.330903723	0.575368332	9.13E-25	8.33E-23
Trabecular meshwork 3	Col5a1	0.331414829	0.732075031	6.66E-24	5.63E-22
Trabecular meshwork 3	Rarres2	0.342133299	1.031795606	9.97E-16	4.49E-14
Trabecular meshwork 3	Mfap5	0.346583296	0.624733097	1.88E-19	1.19E-17
Trabecular meshwork 3	Ebf1	0.34861011	0.306238136	1.92E-34	3.06E-32
Trabecular meshwork 3	Mt2	0.360068607	2.104365402	2.20E-13	7.36E-12
Trabecular meshwork 3	Ctgf	0.371154508	1.487851988	4.29E-08	6.18E-07
Trabecular meshwork 3	S100a6	0.400071266	3.188806473	1.39E-24	1.23E-22
Trabecular meshwork 3	Crip1	0.408056728	1.063458444	6.12E-16	2.85E-14
Trabecular meshwork 3	Col3a1	0.409137724	1.894714235	4.93E-15	2.10E-13
Trabecular meshwork 3	Mt1	0.415906633	2.951369374	2.03E-09	3.80E-08
Trabecular meshwork 3	Igfbp6	0.428326792	1.433509932	1.72E-12	5.09E-11
Trabecular meshwork 3	Col14a1	0.470972309	0.593487236	1.57E-31	2.19E-29
Trabecular meshwork 3	Thbs4	0.476521803	0.846040938	2.29E-17	1.18E-15
Trabecular meshwork 3	Hspe1	0.488300572	1.332705761	2.99E-53	1.27E-50
Trabecular meshwork 3	Ltbp4	0.501351555	1.030842916	2.62E-34	4.09E-32
Trabecular meshwork 3	Mmp2	0.545015355	1.023245706	4.47E-43	1.12E-40
Trabecular meshwork 3	Col6a1	0.545534132	1.450279912	1.01E-34	1.63E-32
Trabecular meshwork 3	Xist	0.54728354	0.849447374	1.29E-47	3.85E-45

Trabecular meshwork 3	Igfbp5	0.626320115	1.986962257	4.88E-21	3.41E-19
Trabecular meshwork 3	Lars2	0.668218675	0.963236725	2.64E-75	2.85E-72
Trabecular meshwork 3	Col1a1	0.691317481	3.307154582	4.35E-27	4.59E-25
Trabecular meshwork 3	Hspa1b	0.875297996	1.072875104	1.40E-42	3.38E-40
Trabecular meshwork 3	Hspa1a	0.925512282	1.103118152	1.23E-41	2.78E-39
Uveal	Csrp1	-0.715574914	2.269225625	1.01E-28	3.01E-26
Uveal	Ifrd1	-0.631958078	3.26484526	1.41E-22	2.57E-20
Uveal	Hspa5	-0.588428939	3.402170129	1.72E-33	7.30E-31
Uveal	Gsn	-0.569191284	1.753553523	9.84E-30	3.21E-27
Uveal	Slc2a1	-0.556110023	1.516893306	3.93E-26	9.69E-24
Uveal	Lmna	-0.534583804	2.974429846	5.56E-40	3.00E-37
Uveal	Pim1	-0.529694678	1.785405445	2.05E-25	4.64E-23
Uveal	Herpud1	-0.529421686	1.761946242	1.29E-25	3.01E-23
Uveal	Rcan1	-0.488753521	1.24895589	7.15E-18	9.05E-16
Uveal	Junb	-0.478069199	2.762697799	1.14E-12	8.16E-11
Uveal	Mat2a	-0.470602972	2.362208993	3.55E-14	3.04E-12
Uveal	Manf	-0.47013098	1.6739477	3.11E-19	4.36E-17
Uveal	Pqlc1	-0.46627161	1.204724007	1.33E-21	2.24E-19
Uveal	Neat1	-0.456472486	0.67253744	3.17E-24	6.64E-22
Uveal	Actg1	-0.407026737	2.662180811	6.08E-20	9.07E-18
Uveal	Penk	-0.398657945	1.370152714	3.69E-07	1.22E-05
Uveal	Ier3	-0.385458721	2.479907349	7.09E-10	3.49E-08
Uveal	Ubc	-0.38339391	2.205931828	1.71E-16	1.95E-14
Uveal	Icam1	-0.381900585	1.71875477	1.39E-16	1.62E-14
Uveal	Gem	-0.368817919	2.596930984	6.06E-08	2.28E-06
Uveal	Pde10a	-0.366292086	0.621023969	3.31E-19	4.61E-17
Uveal	Sdc4	-0.34965229	2.883853993	3.99E-15	3.89E-13
Uveal	Sparc	-0.345783504	2.991288983	4.47E-19	6.03E-17
Uveal	Dusp1	-0.33962214	1.041766964	2.46E-11	1.44E-09
Uveal	Plekhf1	-0.338608586	0.564524158	3.95E-20	5.96E-18
Uveal	Arl4d	-0.330305788	1.091191759	7.73E-10	3.79E-08
Uveal	Ogfrl1	-0.322977454	1.0948411	2.84E-09	1.32E-07
Uveal	Dnajb1	-0.318170146	1.026774227	1.30E-11	8.00E-10
Uveal	Phlda1	-0.316938858	1.763763127	7.47E-07	2.31E-05
Uveal	Itm2a	-0.316610114	1.32676113	2.18E-11	1.31E-09
Uveal	Serpine2	-0.288936352	2.845015901	8.76E-08	3.22E-06
Uveal	Mpp7	-0.28837149	0.315916949	3.60E-22	6.31E-20
Uveal	Igf1	-0.287344832	0.447521214	1.60E-11	9.75E-10
Uveal	Lypd1	-0.285212732	0.705093977	7.44E-09	3.24E-07
Uveal	Klf4	-0.28412235	1.626305127	1.56E-06	4.50E-05
Uveal	Ddit3	-0.282728175	0.623307575	8.11E-12	5.22E-10
Uveal	Gpi1	-0.280314988	0.829359332	1.79E-14	1.57E-12
Uveal	Bdnf	-0.277728591	0.599254547	7.23E-09	3.18E-07
Uveal	Mfap2	-0.275566035	0.76205077	6.90E-11	3.81E-09
Uveal	Ptn	-0.268403339	1.464680586	8.34E-07	2.54E-05
Uveal	Atf3	-0.266500153	1.072403043	4.24E-06	0.000110771
Uveal	Sat1	-0.265561731	2.26383549	2.59E-05	0.000571911
Uveal	Metrnl	-0.260921036	1.172458348	1.04E-05	0.000249238
Uveal	Ctsc	-0.257900564	1.49618701	1.09E-05	0.000260122
Uveal	Fam46a	-0.254946991	1.273962491	2.04E-05	0.00046246
Uveal	Pim3	-0.254425649	0.690247838	4.53E-11	2.60E-09
Uveal	Enpp2	-0.25082993	0.257761366	3.91E-13	2.94E-11
Uveal	Oaf	-0.250206671	1.606474743	4.30E-06	0.000111947
Uveal	Foxc2	-0.250078471	0.233203955	5.78E-24	1.14E-21
Uveal	Litaf	-0.248380069	0.777545902	1.84E-09	8.72E-08
Uveal	Edn3	-0.235194448	0.67829443	5.95E-09	2.65E-07
Uveal	Ecm1	-0.233572127	1.442493088	7.85E-06	0.000192304
Uveal	Wnt4	-0.23244816	0.475953791	1.94E-09	9.17E-08
Uveal	Fjx1	-0.230660069	0.822056387	1.61E-05	0.00037313
Uveal	Cited2	-0.227114597	1.239534314	0.0003437	0.005194355
Uveal	Tsc22d1	-0.226936954	2.425535972	5.90E-07	1.87E-05
Uveal	Ndufs2	-0.224243354	0.534222918	2.52E-10	1.32E-08
Uveal	Gja1	-0.220014444	0.826761279	2.39E-06	6.57E-05

Uveal	Jun	-0.214808081	1.290452783	0.0026126	0.02683127
Uveal	Ncald	-0.213259842	0.621516605	8.36E-08	3.09E-06
Uveal	Got1	-0.213259254	0.343550458	2.31E-11	1.37E-09
Uveal	Id3	-0.21025199	3.257182948	0.0002353	0.003802439
Uveal	Kcnq1ot1	-0.207123438	0.664263329	2.47E-06	6.76E-05
Uveal	Cebpb	-0.206326352	2.817247166	0.0001253	0.002215435
Uveal	Uap1	-0.20475752	0.738419879	9.20E-07	2.79E-05
Uveal	Rgs16	-0.204670191	0.401189592	8.90E-07	2.70E-05
Uveal	Medag	-0.203055947	0.676535226	1.52E-05	0.000353284
Uveal	Procr	-0.201172721	0.836618907	0.0036915	0.035064633
Uveal	Serpinb6a	-0.200988416	0.772782947	1.58E-06	4.52E-05
Uveal	Lum	-0.200104627	1.917931878	0.0002334	0.003793144
Uveal	Sod3	-0.19635596	0.452599838	5.87E-09	2.62E-07
Uveal	Ddx3y	-0.193813306	0.502374747	1.63E-08	6.78E-07
Uveal	Eif4ebp1	-0.189125706	0.744163439	5.87E-05	0.001179271
Uveal	Stc1	-0.183782024	0.442523366	0.0003408	0.005168567
Uveal	Ptgir	-0.183098966	0.710542396	5.16E-05	0.001053711
Uveal	Srxn1	-0.182749636	1.001644621	0.0008256	0.010633227
Uveal	Meg3	-0.182410396	0.810740083	1.43E-05	0.000334196
Uveal	Myl9	-0.181198851	1.154585057	0.0012374	0.014864903
Uveal	Cyp51	-0.177503439	0.598581184	1.52E-05	0.000352762
Uveal	Msmo1	-0.177378222	0.305827971	5.60E-11	3.17E-09
Uveal	Foxs1	-0.173133345	0.429711261	9.95E-07	3.00E-05
Uveal	Rap2b	-0.172216121	0.658950307	0.0001724	0.002888674
Uveal	Col11a1	-0.17177843	0.312888139	2.66E-10	1.38E-08
Uveal	Dbp	-0.169528334	0.240469372	1.72E-11	1.05E-09
Uveal	Krt12	-0.166311606	0.191831363	8.94E-15	8.20E-13
Uveal	C4b	-0.163433585	0.649970073	0.0001885	0.003128861
Uveal	Zfp131	-0.161760631	0.732775473	0.0003007	0.004696025
Uveal	Spsb2	-0.157340479	0.429847003	3.60E-07	1.20E-05
Uveal	Gpr137b	-0.15600107	0.535546304	0.0003051	0.004737489
Uveal	Marcksl1	-0.155940518	1.31985732	0.0023337	0.024914674
Uveal	Pdha1	-0.153397096	0.582476351	8.28E-05	0.001582036
Uveal	Gja4	-0.152270926	0.527285127	0.0004727	0.006798913
Uveal	Plagl1	-0.150807039	0.792056437	0.0022599	0.024219296
Uveal	Cmbl	-0.146479202	0.230868417	1.82E-08	7.50E-07
Uveal	Ctxn3	-0.145712095	0.281306585	4.21E-06	0.000110191
Uveal	Kctd1	-0.145568698	0.586031754	5.37E-05	0.001087218
Uveal	D10Wsu102e	-0.14418194	0.685616534	0.0001721	0.002888051
Uveal	Ddit4	-0.143702331	0.346723461	7.25E-06	0.000179
Uveal	Slco3a1	-0.141742677	0.221979414	9.78E-08	3.57E-06
Uveal	Rasl11b	-0.139162463	0.442143048	0.0002939	0.00460999
Uveal	Itih5	-0.137961456	0.444437102	0.000392	0.005792772
Uveal	D16Ertd472e	-0.13220812	0.355850967	9.09E-05	0.00170532
Uveal	H2-Q4	-0.130545425	0.558129805	0.000816	0.010521726
Uveal	Mgarp	-0.12724654	0.668029408	0.0010042	0.012491427
Uveal	Prkca	-0.125309363	0.334046348	9.71E-05	0.001796402
Uveal	Crlf1	-0.125084427	0.417580144	0.0012378	0.014864903
Uveal	Hic1	-0.124729486	0.443418364	0.0002276	0.003710861
Uveal	Wnt6	-0.121264198	0.464275111	0.0010069	0.012509921
Uveal	Rab20	-0.119680353	0.376825041	0.0005175	0.007309455
Uveal	Rnf126	-0.119330774	0.268307433	5.44E-06	0.000138559
Uveal	Gm4841	-0.11812486	0.33781928	0.0026491	0.027087115
Uveal	Cited1	-0.118087805	0.230354513	0.0013995	0.016414037
Uveal	Mzt2	-0.117611479	0.394552853	0.0019562	0.021489464
Uveal	Ctsl	-0.116104383	2.906984506	0.0008434	0.010843561
Uveal	Creld2	-0.114160304	0.298256037	9.94E-05	0.001822548
Uveal	Fstl3	-0.113377437	0.343082611	0.0007035	0.009299336
Uveal	Ptgs2os	-0.111049528	0.272585357	0.000219	0.003587605
Uveal	Wnt10a	-0.11052832	0.116978144	1.34E-07	4.80E-06
Uveal	Fdps	-0.110124952	0.297711157	0.0004173	0.006115979
Uveal	Rap2a	-0.110118254	0.306399326	0.000415	0.00608864
Uveal	Slc16a3	-0.10921869	0.303500528	0.0004799	0.006867443

Uveal	Tmem37	-0.109147426	0.266029384	4.14E-05	0.000866419
Uveal	Tubb2b	-0.10658207	0.256077312	0.0002262	0.003693391
Uveal	Fos	-0.106274844	1.161459481	9.25E-06	0.000223222
Uveal	Atp1a2	-0.10358651	0.179137702	0.0001244	0.002210139
Uveal	Pja1	-0.103120611	0.358636841	0.0011994	0.014528
Uveal	Fscn1	-0.102011745	0.366501839	0.0034885	0.033476032
Uveal	Sdf2l1	-0.096555002	0.381205202	0.0050052	0.044502029
Uveal	Sqle	-0.095094046	0.280966209	0.0026941	0.027427546
Uveal	Pi16	-0.094941022	0.236060001	0.0005446	0.007622448
Uveal	P2ry14	-0.093686393	0.205613038	0.0007617	0.009944952
Uveal	Opa3	-0.093456015	0.205253728	0.0002413	0.003885073
Uveal	Fam110b	-0.090116347	0.134800616	2.47E-06	6.76E-05
Uveal	Adra2a	-0.087097899	0.131947658	0.0001293	0.00226955
Uveal	Sfn	-0.086429722	0.115875992	1.82E-06	5.17E-05
Uveal	Slc43a3	-0.086232298	0.1801528	0.000549	0.007646866
Uveal	4930523C07Rik	-0.085045284	0.217866866	0.0042126	0.03890825
Uveal	Rab3a	-0.083941999	0.129234976	3.76E-05	0.000794413
Uveal	Ramp3	-0.079875195	0.086157038	8.88E-08	3.24E-06
Uveal	Usp2	-0.078686678	0.09817171	6.80E-06	0.00016951
Uveal	Gadd45a	-0.078426102	1.012946438	0.0040001	0.037214944
Uveal	Airn	-0.077898559	0.147208402	0.0003961	0.005840994
Uveal	Vcan	-0.076145738	0.153180309	0.0014702	0.017040342
Uveal	Tmem119	-0.072851227	0.185221891	0.0047205	0.042590612
Uveal	Lamb1	-0.072467456	0.408263515	0.0004278	0.006249252
Uveal	Cldn5	-0.071434738	0.062134865	4.65E-06	0.000119891
Uveal	Ralgds	-0.070377287	0.167375619	0.0037059	0.035121703
Uveal	Dusp3	-0.070356876	0.134007169	0.0002778	0.004376722
Uveal	Lpar1	-0.070293126	0.105583745	9.85E-05	0.001812122
Uveal	Fdft1	-0.067881999	0.137118992	0.0011938	0.014473248
Uveal	Pdk3	-0.067508935	0.214802305	0.0027228	0.027679551
Uveal	Ucp2	-0.066696772	0.16126366	0.0008129	0.010498379
Uveal	Ppt2	-0.065709598	0.141466575	0.0036556	0.034793587
Uveal	Ms4a4d	-0.062528141	0.073164346	1.36E-05	0.00032004
Uveal	Slc9a3r1	-0.061774273	0.098532772	0.000651	0.008728784
Uveal	Aspscr1	-0.059833517	0.125259397	0.0027584	0.027840195
Uveal	Rbp4	-0.057104549	0.08692854	0.0009122	0.011537497
Uveal	Lrp1b	-0.056346608	0.119534718	7.06E-05	0.001383376
Uveal	Meox1	-0.056314114	0.043279232	4.67E-07	1.51E-05
Uveal	Tmem132c	-0.054963362	0.117185721	0.0041955	0.038801494
Uveal	Adamts16	-0.052615632	0.046573026	7.21E-07	2.23E-05
Uveal	Milr1	-0.051888036	0.108883208	0.0058203	0.049702713
Uveal	Tppp3	-0.051373124	0.141769521	0.0056413	0.048677719
Uveal	Ttc39c	-0.050765375	0.098045634	0.0051838	0.045598376
Uveal	Hsd11b1	-0.049391789	0.067139716	0.0004262	0.006232777
Uveal	Rit2	-0.048719805	0.030855877	4.89E-10	2.49E-08
Uveal	Il13ra2	-0.047149017	0.038014421	2.77E-05	0.000607287
Uveal	B3gat2	-0.042916341	0.064422934	0.0043684	0.039875352
Uveal	Abi3bp	-0.038798648	1.724220225	0.0007127	0.009394696
Uveal	Pnpo	-0.037755372	0.046325654	0.0027449	0.02776369
Uveal	Kcnq3	-0.03334518	0.02884173	0.0002354	0.003802439
Uveal	Ptger3	-0.032743892	0.070237459	0.0053568	0.04657658
Uveal	Upp1	-0.032590877	0.123304121	0.0039117	0.036659439
Uveal	Mfap5	-0.032131664	0.364216155	0.0038617	0.036312846
Uveal	Sorcs3	-0.028117408	0.02265554	3.28E-05	0.000700536
Uveal	Stra6	-0.026995096	0.025566698	0.0028298	0.028417807
Uveal	Bmp7	-0.026785799	0.025842128	0.0007722	0.010047767
Uveal	Bdkrb1	-0.02602389	0.024916714	0.0031496	0.03072803
Uveal	Ccr1	-0.023584281	0.014936711	1.17E-05	0.000277783
Uveal	Tnfaip8	-0.014254518	0.289598467	0.0024693	0.025847871
Uveal	Nop53	-0.003775925	0.496522283	0.0054568	0.047406033
Uveal	Il11	-0.001507576	0.644818697	0.0058376	0.049819772
Uveal	Ccl7	0.005520473	0.427790843	0.0013502	0.015968668
Uveal	Emb	0.006541245	0.96372955	0.0024934	0.026014343

Uveal	H2-Q7	0.008152645	0.010875321	0.0019054	0.021079515
Uveal	Nrn1	0.013154616	0.005933339	0.0049378	0.044013607
Uveal	Sox13	0.01417025	0.009418976	0.0024016	0.025319584
Uveal	Ccl5	0.015030686	0.00828288	0.005059	0.044809812
Uveal	Zik1	0.016054752	0.00788096	0.0024804	0.025928428
Uveal	Ctla2a	0.018285552	0.007540913	0.0007227	0.009491626
Uveal	Naif1	0.021013075	0.011955962	0.0033713	0.032528646
Uveal	St8sia2	0.021406627	0.013313705	0.004321	0.03951956
Uveal	Bmp5	0.023021097	0.016151947	0.0055699	0.048150524
Uveal	Zfp383	0.024701461	0.018153509	0.0055615	0.048136718
Uveal	Il33	0.024877519	0.014400915	0.0001689	0.002853212
Uveal	Ephb1	0.025062812	0.01573893	0.0023953	0.025302942
Uveal	Rbbp5	0.026593035	0.021491453	0.0017829	0.019976427
Uveal	Rnf112	0.026800141	0.018561858	0.0022695	0.024303564
Uveal	Slco4a1	0.030664586	0.028577418	0.0016691	0.018836773
Uveal	Crispld1	0.035150773	0.028491327	0.0019985	0.021902626
Uveal	Inmt	0.03551001	0.052979122	0.0022044	0.023842563
Uveal	Zfp60	0.035784034	0.030161028	0.0023315	0.024910358
Uveal	Mid2	0.036954074	0.038917097	0.003344	0.0322874
Uveal	Ndrg4	0.037496091	0.032391217	0.0006506	0.008728784
Uveal	Slc7a8	0.039338154	0.068387046	0.0048854	0.043624254
Uveal	Npr3	0.040036994	0.046838642	0.0042037	0.038851972
Uveal	Slc39a8	0.041894538	0.036533371	0.0003318	0.00507419
Uveal	Pthlh	0.041974377	0.075403377	0.0033417	0.0322874
Uveal	Adam19	0.042333523	0.070643029	0.0026529	0.027094864
Uveal	Znrf3	0.042716197	0.048575228	0.0023554	0.025013325
Uveal	Clk2	0.042783447	0.060687246	0.003534	0.033843429
Uveal	Tnc	0.044157374	0.044550976	0.0002608	0.00414161
Uveal	Trim24	0.044501964	0.089540496	0.0022159	0.023943171
Uveal	Sct	0.044656354	0.034407266	0.00024	0.003868039
Uveal	Agl	0.044997636	0.04787526	0.0008538	0.010936477
Uveal	Slc26a7	0.046209373	0.056571581	0.0013043	0.015557098
Uveal	Eif2ak3	0.046335838	0.039913982	0.0001805	0.003002146
Uveal	Perp	0.046879647	0.090570472	0.0016055	0.018324725
Uveal	Akap1	0.047611697	0.066380232	0.0022542	0.024176273
Uveal	Slc16a10	0.048312167	0.065794871	0.0035767	0.034158826
Uveal	Gid4	0.048677103	0.091532089	0.0014585	0.016936515
Uveal	Heyl	0.051636761	0.059846982	0.0008553	0.010945403
Uveal	Snai2	0.051640459	0.078143372	0.0029408	0.029239002
Uveal	Ccl11	0.052133977	0.045696092	0.0045889	0.04162197
Uveal	Tfpi2	0.052160277	0.038933878	0.0010818	0.01330921
Uveal	Nol8	0.052449166	0.074462947	0.0001095	0.001993876
Uveal	Cxcr4	0.053411478	0.049565537	9.05E-05	0.001701038
Uveal	Dnajc13	0.053614515	0.092430398	0.0034221	0.03290621
Uveal	Col14a1	0.055154068	0.070677341	0.0035308	0.033835727
Uveal	Plekhg3	0.056964527	0.072283304	5.02E-05	0.001026844
Uveal	Pcdh10	0.057000443	0.081583435	0.0005856	0.008027945
Uveal	Notch3	0.05720273	0.076328219	0.0005736	0.007880652
Uveal	Rab26os	0.058462061	0.042706466	7.74E-07	2.37E-05
Uveal	Ebf1	0.059042927	0.052545399	1.79E-05	0.0004104
Uveal	Mcm6	0.060613406	0.100743163	0.0014143	0.016571602
Uveal	Arhgap28	0.061476639	0.095489398	0.0003633	0.005438203
Uveal	Zfp367	0.062094276	0.066407732	4.82E-05	0.000991603
Uveal	C77080	0.062672674	0.096611079	0.0010045	0.012491427
Uveal	Larp4b	0.062772381	0.137363789	0.0056711	0.048846959
Uveal	Tubb2a	0.063476261	0.836609047	0.0052746	0.046137013
Uveal	Enc1	0.064409024	0.150307884	0.0001833	0.003045554
Uveal	Ipmk	0.064554283	0.116107277	0.0020437	0.022327823
Uveal	Tnfaip2	0.06607857	0.060861819	1.95E-05	0.000443882
Uveal	Arl4c	0.068048999	0.081759345	0.0002079	0.003433937
Uveal	Epha4	0.068339163	0.147871694	0.0046725	0.042266121
Uveal	S1pr1	0.068802506	0.129620146	0.0018346	0.02045773
Uveal	Ifit3b	0.069217839	0.107125014	0.0013834	0.016252733

Uveal	Kera	0.069274581	0.064385298	1.79E-05	0.000411226
Uveal	Itpripl2	0.071462741	0.166123729	0.0056069	0.048410599
Uveal	Hist1h2ap	0.07334548	0.057809456	7.10E-07	2.21E-05
Uveal	Ptgs2	0.073482986	1.783649872	0.0015742	0.017996432
Uveal	Thbs4	0.073627765	0.055603384	9.07E-06	0.000219067
Uveal	Cx3cl1	0.073720259	0.176459999	0.0047659	0.042834712
Uveal	Synm	0.073889766	0.089128516	5.00E-05	0.001023833
Uveal	B4galt5	0.075265918	0.097131813	4.00E-05	0.000839752
Uveal	Creg2	0.075691588	0.080767629	6.75E-06	0.000168845
Uveal	Pttg1	0.075863532	0.182188672	0.0045692	0.041519374
Uveal	Adarb1	0.076565951	0.136418816	0.0005667	0.007808108
Uveal	Fam98b	0.078762114	0.118640793	5.83E-05	0.001173038
Uveal	Angptl7	0.07896342	0.049224531	4.74E-10	2.42E-08
Uveal	H2-T23	0.080625065	0.139876877	0.0002341	0.003794375
Uveal	Slc30a5	0.083414474	0.168316063	0.0007543	0.009860272
Uveal	Gstp1	0.084225859	0.265992019	0.002841	0.028469259
Uveal	Fkbp5	0.08604707	0.096487904	1.10E-06	3.25E-05
Uveal	Rc3h2	0.086831604	0.172300276	0.0003132	0.004843225
Uveal	Fgfr3	0.087224043	0.246856999	0.0036965	0.035087841
Uveal	Ak1	0.087746685	0.152658016	8.84E-05	0.001668457
Uveal	Mirg	0.088185123	0.221852724	0.000703	0.009299336
Uveal	Fgf2	0.09056535	0.142388561	1.14E-05	0.000270603
Uveal	Il6	0.090647979	0.187371904	0.0037076	0.035121703
Uveal	Lacc1	0.09072826	0.108163203	3.47E-06	9.18E-05
Uveal	Slc16a1	0.090832227	0.245680909	0.0033377	0.032279049
Uveal	Rictor	0.091413744	0.203149471	0.0002757	0.004349082
Uveal	Fzd7	0.093114164	0.15542633	9.40E-05	0.001745449
Uveal	Pak1	0.09312325	0.183352193	0.0002107	0.003471861
Uveal	Foxl1	0.09381307	0.119558375	7.74E-06	0.000190072
Uveal	Myc	0.095377613	0.377715909	0.0051577	0.045397806
Uveal	Fabp3	0.095601942	0.222870066	0.0025137	0.026102434
Uveal	Amotl2	0.095646314	0.182329425	0.0003385	0.005143571
Uveal	Btbd3	0.095673384	0.24887287	0.0021625	0.023443396
Uveal	Cda	0.096448177	0.191569464	0.002375	0.025202018
Uveal	Peg10	0.097573271	0.163294556	0.0002005	0.003320179
Uveal	Rsbn1l	0.098763451	0.220067213	0.0001646	0.002791647
Uveal	Oasl2	0.09945511	0.294382798	0.002428	0.025496445
Uveal	Nudt4	0.099934458	0.693207011	0.0010912	0.013403306
Uveal	Angpt1	0.099991202	0.321860252	0.002662	0.027140105
Uveal	Tmcc3	0.100636481	0.245191466	0.0005056	0.007191143
Uveal	Myo10	0.100885278	0.278137103	0.0009298	0.011718235
Uveal	Dusp5	0.103760678	0.344304578	0.0042655	0.039208073
Uveal	Phldb2	0.103914778	0.354129886	0.0005109	0.007244807
Uveal	Aspn	0.10465404	0.201226333	0.0004631	0.006668516
Uveal	Gng11	0.10616098	0.444288879	0.0028719	0.028692032
Uveal	Bcl2l11	0.107064081	0.301539831	0.0018069	0.020212714
Uveal	Limd1	0.107444792	0.245845461	0.0001225	0.002183233
Uveal	Hist1h2bc	0.107903295	0.295377548	0.0008628	0.011003917
Uveal	Sox9	0.108652833	0.308888671	0.0024991	0.026046914
Uveal	Brd2	0.110438459	0.878148729	0.0050262	0.044575344
Uveal	Atp2a2	0.114629655	1.569736543	0.0052465	0.045987637
Uveal	Txnip	0.115713967	0.249526051	0.0022408	0.024085299
Uveal	Basp1	0.117803633	0.374282318	0.0013767	0.016214598
Uveal	Acsl3	0.118295749	0.692297297	0.0017589	0.019723072
Uveal	Plod2	0.118378957	0.28563986	0.0003317	0.00507419
Uveal	Peg3	0.119257443	0.504790956	0.00463	0.041936244
Uveal	Hmgn5	0.121742183	0.263576179	5.72E-05	0.001155391
Uveal	S100b	0.124376618	0.295284944	3.66E-05	0.000776184
Uveal	Map3k8	0.127068488	0.360293652	0.0001695	0.002857051
Uveal	Ralbp1	0.127887085	0.542205497	0.00165	0.018728615
Uveal	Col6a1	0.132976291	0.807931254	0.0035677	0.034095749
Uveal	St5	0.138921696	0.284514957	2.37E-06	6.55E-05
Uveal	Tpbg	0.14074354	0.802845975	0.004941	0.044014805

Uveal	Ntn4	0.142195231	0.27134798	3.91E-06	0.000102728
Uveal	Pfkfb3	0.14229499	0.498144086	0.0006459	0.008711187
Uveal	Itga5	0.142412964	0.48849312	9.70E-06	0.000233564
Uveal	Snhg9	0.144822711	0.081852392	5.98E-19	7.92E-17
Uveal	Map1b	0.147763242	0.368059126	5.32E-05	0.001082703
Uveal	Cd44	0.150954104	0.38625051	9.77E-05	0.001802932
Uveal	Efhd2	0.153444343	0.58332412	0.0024708	0.025847871
Uveal	Fam107b	0.153824504	0.676463361	0.0013382	0.015867876
Uveal	Tnfrsf11b	0.156226523	0.347005279	0.0001649	0.002792817
Uveal	Il1r1	0.156474796	0.510964614	0.0001179	0.002116512
Uveal	Abracl	0.158996447	0.984120899	0.0048471	0.043425584
Uveal	Apod	0.159517338	0.636577324	8.47E-05	0.001610527
Uveal	Rnd3	0.160899377	0.778127491	0.000857	0.010957459
Uveal	Gdf10	0.162357674	0.225426738	6.16E-09	2.74E-07
Uveal	Hmgb2	0.162728749	0.560583784	3.26E-06	8.65E-05
Uveal	Tgfb3	0.169757716	0.320622514	5.21E-08	1.99E-06
Uveal	Ank	0.172434642	0.73894985	0.0001161	0.002087574
Uveal	Fgl2	0.177676036	0.741643498	0.0007882	0.010236115
Uveal	Fdx1	0.179481087	0.957351378	0.0014563	0.016925104
Uveal	Denr	0.182188714	0.386323447	6.29E-08	2.36E-06
Uveal	Wnt11	0.183322585	0.460875867	5.73E-07	1.82E-05
Uveal	Plec	0.184281997	0.571836785	2.28E-06	6.35E-05
Uveal	Wdr43	0.185720742	0.296472027	2.68E-11	1.55E-09
Uveal	Ifit1	0.187319738	0.614211602	0.0003413	0.005168567
Uveal	Crip1	0.191237097	0.31309368	1.18E-08	5.00E-07
Uveal	Pam	0.193383338	1.494875469	0.00056	0.007735877
Uveal	Cstb	0.19401948	1.726525115	0.0007252	0.009506389
Uveal	Gpc3	0.195205106	0.679185265	1.54E-05	0.000357115
Uveal	Spry2	0.199476772	0.792281253	2.12E-05	0.000479706
Uveal	Tmsb10	0.19970857	3.472213165	3.87E-05	0.00081675
Uveal	Col5a1	0.200508431	0.391844327	8.77E-10	4.29E-08
Uveal	P4ha1	0.200655606	0.769120616	2.54E-06	6.88E-05
Uveal	Csf1	0.205118061	0.411632981	1.06E-06	3.14E-05
Uveal	Xist	0.205438155	0.748260918	1.18E-25	2.80E-23
Uveal	Sparcl1	0.205524752	0.180184358	3.69E-13	2.78E-11
Uveal	Zfp36	0.205767925	0.744226588	0.0001266	0.00223073
Uveal	Hspa1b	0.206380999	0.763815952	0.0002636	0.00418095
Uveal	Ogn	0.208119507	0.826442657	3.10E-05	0.000667807
Uveal	Gm13889	0.208319412	2.037053142	0.0013211	0.015691287
Uveal	Fzd1	0.208784809	0.517872157	5.57E-08	2.12E-06
Uveal	Bst2	0.214552635	0.685486182	2.26E-06	6.31E-05
Uveal	Id2	0.221928139	0.475770201	1.85E-07	6.51E-06
Uveal	Igf2	0.223241234	0.517161008	4.35E-10	2.23E-08
Uveal	Hspa1a	0.226234441	0.754646748	0.0008316	0.010700422
Uveal	Ssh1	0.237573263	0.406633932	1.33E-12	9.40E-11
Uveal	Cfh	0.237876942	0.388208251	1.94E-11	1.17E-09
Uveal	Fndc1	0.245979553	0.745277394	1.31E-08	5.51E-07
Uveal	Thbs2	0.255024921	0.676638612	2.90E-09	1.34E-07
Uveal	Zfp36l2	0.272522718	0.822226569	1.79E-09	8.50E-08
Uveal	Ltbp4	0.277088144	0.649156363	6.82E-11	3.78E-09
Uveal	Emp1	0.285575558	1.85311441	9.67E-09	4.19E-07
Uveal	Hmcn1	0.287172737	0.563710116	4.11E-12	2.75E-10
Uveal	S100a10	0.28846148	1.251070359	1.00E-06	3.01E-05
Uveal	Hspe1	0.29413738	1.05008374	7.50E-14	6.05E-12
Uveal	Cd200	0.298655426	0.609982687	9.76E-11	5.29E-09
Uveal	Fst	0.302550394	0.671167659	2.58E-07	8.83E-06
Uveal	Igfbp5	0.305098396	2.505746057	4.27E-08	1.66E-06
Uveal	Col12a1	0.316809083	0.878146143	1.14E-11	7.16E-10
Uveal	S100a13	0.327780574	0.650860213	1.90E-16	2.15E-14
Uveal	Col1a1	0.32924242	1.874178049	1.29E-11	8.00E-10
Uveal	Plaur	0.330985155	0.485181306	4.23E-16	4.63E-14
Uveal	Igfbp2	0.331034713	1.750307687	6.02E-06	0.00015177
Uveal	Ltbp1	0.344730423	0.960299581	9.00E-20	1.32E-17

Uveal	A2m	0.353790125	0.526050614	1.19E-22	2.19E-20
Uveal	Tm4sf1	0.363689991	0.728202151	5.21E-08	1.99E-06
Uveal	Timp3	0.368598018	1.665652832	3.51E-12	2.37E-10
Uveal	Mt2	0.371578497	1.444780743	1.86E-07	6.53E-06
Uveal	Cryab	0.382822129	2.101568004	4.72E-05	0.000973451
Uveal	Thbs1	0.505047742	1.964630716	8.85E-14	7.10E-12
Uveal	Lars2	0.674995303	0.843447191	7.91E-66	1.01E-62
Corneal stromal keratocytes	Mfap4	-0.649411429	2.665295863	1.37E-190	6.21E-188
Corneal stromal keratocytes	Ifi27l2a	-0.477926895	0.889822115	1.41E-65	8.52E-64
Corneal stromal keratocytes	Crabp1	-0.402008503	0.709966056	6.63E-81	5.97E-79
Corneal stromal keratocytes	Cthrc1	-0.39939894	0.94189408	1.95E-80	1.74E-78
Corneal stromal keratocytes	Col3a1	-0.352408178	1.486629933	8.91E-42	2.21E-40
Corneal stromal keratocytes	Sparc	-0.341069764	5.462064908	1.45E-281	1.35E-278
Corneal stromal keratocytes	Meg3	-0.330696415	1.416126455	6.81E-55	2.80E-53
Corneal stromal keratocytes	Fibin	-0.323826127	0.628154437	4.23E-73	3.16E-71
Corneal stromal keratocytes	Rarres2	-0.312954643	0.67372328	1.38E-77	1.15E-75
Corneal stromal keratocytes	Nupr1	-0.295321642	3.372143811	8.44E-39	1.81E-37
Corneal stromal keratocytes	Fjx1	-0.289653026	0.923154159	1.05E-41	2.59E-40
Corneal stromal keratocytes	Nnmt	-0.281723591	0.757829419	9.26E-52	3.40E-50
Corneal stromal keratocytes	Hmox1	-0.28066126	2.471695118	8.22E-16	4.25E-15
Corneal stromal keratocytes	Srxn1	-0.268573796	1.257153459	1.60E-27	1.81E-26
Corneal stromal keratocytes	Neat1	-0.266891399	0.968675826	3.75E-39	8.20E-38
Corneal stromal keratocytes	Mdh1	-0.257309027	0.614706861	3.71E-73	2.79E-71
Corneal stromal keratocytes	Camk2d	-0.25362686	0.909225891	8.27E-49	2.68E-47
Corneal stromal keratocytes	Actg1	-0.253381738	2.341104693	1.55E-43	4.12E-42
Corneal stromal keratocytes	Ubb	-0.251081601	3.219094602	2.94E-36	5.53E-35
Corneal stromal keratocytes	Mdk	-0.238843584	0.840069194	4.46E-49	1.46E-47
Corneal stromal keratocytes	Phlda1	-0.237664426	0.924846601	6.92E-31	9.61E-30
Corneal stromal keratocytes	Cst3	-0.230577219	1.888538013	4.75E-42	1.19E-40
Corneal stromal keratocytes	Chac1	-0.223703585	0.535602286	4.64E-40	1.06E-38
Corneal stromal keratocytes	Ifitm3	-0.21942126	2.501572756	1.54E-27	1.74E-26
Corneal stromal keratocytes	Cpxm1	-0.218814736	0.686247882	4.02E-46	1.18E-44
Corneal stromal keratocytes	Bst2	-0.214488984	0.789309137	4.85E-46	1.42E-44
Corneal stromal keratocytes	Fam46a	-0.208060131	0.838667551	1.20E-32	1.84E-31
Corneal stromal keratocytes	Kcnq1ot1	-0.205375881	0.416215342	4.81E-56	2.06E-54
Corneal stromal keratocytes	Gng11	-0.198438048	0.969161875	5.96E-36	1.10E-34
Corneal stromal keratocytes	Dnajb1	-0.197128964	0.722500937	5.74E-46	1.67E-44
Corneal stromal keratocytes	Gm13889	-0.196824517	0.915397428	2.29E-09	7.05E-09
Corneal stromal keratocytes	Dlk1	-0.193293331	0.428266771	4.10E-46	1.20E-44
Corneal stromal keratocytes	Clic1	-0.193215286	1.481002084	3.21E-29	4.00E-28
Corneal stromal keratocytes	Lgmn	-0.1879373	0.342763586	4.89E-39	1.06E-37
Corneal stromal keratocytes	Ndufa4l2	-0.18454194	0.871011162	1.03E-14	4.89E-14
Corneal stromal keratocytes	Ckb	-0.177957927	0.739210708	8.62E-37	1.66E-35
Corneal stromal keratocytes	Ier2	-0.175627127	0.815271008	5.97E-24	5.44E-23
Corneal stromal keratocytes	Id1	-0.174238218	0.817898964	2.45E-13	1.04E-12
Corneal stromal keratocytes	Tnfrsf12a	-0.172650307	0.952651722	8.37E-25	8.03E-24
Corneal stromal keratocytes	Ednrb	-0.172374092	0.357393535	5.13E-41	1.22E-39
Corneal stromal keratocytes	Jun	-0.167325816	1.143835264	8.29E-17	4.59E-16
Corneal stromal keratocytes	Col9a1	-0.164907565	0.357597759	2.17E-36	4.10E-35
Corneal stromal keratocytes	Csrp2	-0.157760109	0.665750018	6.15E-33	9.69E-32
Corneal stromal keratocytes	Id3	-0.155693637	2.151011349	4.95E-09	1.49E-08
Corneal stromal keratocytes	MT-ATP6	-0.155576592	3.577558575	1.05E-45	3.03E-44
Corneal stromal keratocytes	Cfl1	-0.155254708	0.942846101	3.49E-31	4.96E-30
Corneal stromal keratocytes	Slc38a1	-0.150794918	0.423500817	3.60E-36	6.75E-35
Corneal stromal keratocytes	Sdhb	-0.150564523	0.427097323	1.44E-44	4.00E-43
Corneal stromal keratocytes	Gpi1	-0.145970096	0.868945817	3.36E-21	2.55E-20
Corneal stromal keratocytes	Rab34	-0.14249833	0.313443741	9.09E-47	2.75E-45
Corneal stromal keratocytes	Ddx39b	-0.139043502	0.457624336	1.77E-33	2.88E-32
Corneal stromal keratocytes	Ubtd1	-0.132272088	0.355079773	1.93E-29	2.44E-28
Corneal stromal keratocytes	Ercc1	-0.131558503	0.341701943	1.72E-32	2.62E-31
Corneal stromal keratocytes	Jpt1	-0.131216304	0.393770412	1.23E-39	2.75E-38
Corneal stromal keratocytes	Akt1	-0.130621392	0.431147876	7.01E-37	1.36E-35
Corneal stromal keratocytes	Cryzl2	-0.129425412	0.262099262	2.05E-41	4.97E-40

Corneal stromal keratocytes	Eif4e	-0.129418666	1.133568866	2.69E-21	2.05E-20
Corneal stromal keratocytes	Arpc3	-0.129256627	1.018680766	1.02E-25	1.03E-24
Corneal stromal keratocytes	Cda	-0.125819792	0.874826518	2.25E-06	5.18E-06
Corneal stromal keratocytes	Gch1	-0.124689486	0.382251062	2.65E-20	1.88E-19
Corneal stromal keratocytes	Cdkn1c	-0.123216265	0.40985112	4.09E-17	2.33E-16
Corneal stromal keratocytes	Sqstm1	-0.122949432	0.851799401	7.10E-20	4.87E-19
Corneal stromal keratocytes	H2afz	-0.122743948	1.433226491	3.08E-20	2.18E-19
Corneal stromal keratocytes	Tmsb4x	-0.122576524	3.535646367	4.55E-14	2.05E-13
Corneal stromal keratocytes	Ttc8	-0.120858512	0.340352915	1.48E-22	1.23E-21
Corneal stromal keratocytes	Adm	-0.120233513	0.640692833	1.89E-09	5.88E-09
Corneal stromal keratocytes	Bad	-0.119080798	0.260872439	2.00E-49	6.65E-48
Corneal stromal keratocytes	Bpgm	-0.1182276	0.673299446	8.41E-13	3.42E-12
Corneal stromal keratocytes	Ier3	-0.117518473	2.936702175	3.21E-06	7.25E-06
Corneal stromal keratocytes	H3f3b	-0.115935411	2.625833864	3.66E-10	1.21E-09
Corneal stromal keratocytes	Ptgs2os	-0.115579553	0.250742234	5.76E-30	7.55E-29
Corneal stromal keratocytes	Hilpda	-0.115352651	0.342365361	3.32E-16	1.76E-15
Corneal stromal keratocytes	Timp1	-0.114575416	0.297282562	6.35E-21	4.70E-20
Corneal stromal keratocytes	Mgp	-0.113600332	3.14875789	7.42E-07	1.80E-06
Corneal stromal keratocytes	Osgep	-0.112331453	0.211575259	2.14E-44	5.88E-43
Corneal stromal keratocytes	Spp1	-0.111734518	0.257780809	2.89E-08	8.05E-08
Corneal stromal keratocytes	Lyz2	-0.11088391	0.201384452	3.82E-56	1.66E-54
Corneal stromal keratocytes	Tac1	-0.106998363	0.639712256	0.0001182	0.00022513
Corneal stromal keratocytes	Hist1h2bc	-0.106697162	0.474418639	2.00E-26	2.10E-25
Corneal stromal keratocytes	Marcksl1	-0.106114986	1.259803835	8.88E-06	1.91E-05
Corneal stromal keratocytes	Mat2a	-0.105406818	1.226386917	4.11E-21	3.10E-20
Corneal stromal keratocytes	Txn1	-0.104691397	1.6986771	9.47E-26	9.54E-25
Corneal stromal keratocytes	Rtp4	-0.104409659	0.232390075	1.53E-23	1.35E-22
Corneal stromal keratocytes	Tnmd	-0.103895821	0.425884743	7.97E-12	3.00E-11
Corneal stromal keratocytes	Snrpc	-0.102877976	0.517803915	1.59E-29	2.02E-28
Corneal stromal keratocytes	Acaa1a	-0.102404639	0.232963722	2.20E-38	4.64E-37
Corneal stromal keratocytes	Mrpl51	-0.101990485	0.368316838	2.75E-28	3.27E-27
Corneal stromal keratocytes	Cd34	-0.10145674	0.273250788	1.57E-35	2.85E-34
Corneal stromal keratocytes	Nudt9	-0.099957208	0.948006103	2.45E-29	3.09E-28
Corneal stromal keratocytes	Mrpl14	-0.098853838	0.266602053	6.84E-28	7.93E-27
Corneal stromal keratocytes	Ctsl	-0.097705233	2.996546212	1.47E-05	3.10E-05
Corneal stromal keratocytes	Aldh1a1	-0.094393071	0.383545969	1.51E-11	5.55E-11
Corneal stromal keratocytes	Cfh	-0.093929798	0.506562173	9.96E-21	7.29E-20
Corneal stromal keratocytes	Baiap2	-0.093804134	0.454255991	5.46E-14	2.44E-13
Corneal stromal keratocytes	Adh5	-0.092645166	0.310574551	2.62E-36	4.93E-35
Corneal stromal keratocytes	Psmd4	-0.092135362	0.670544967	1.05E-23	9.37E-23
Corneal stromal keratocytes	Nav2	-0.09205168	0.256982905	2.94E-32	4.46E-31
Corneal stromal keratocytes	Klf2	-0.091381829	0.360090325	4.11E-11	1.47E-10
Corneal stromal keratocytes	Cited2	-0.091275697	0.444728327	8.49E-15	4.04E-14
Corneal stromal keratocytes	Fmo1	-0.091204437	0.280133826	1.38E-15	6.99E-15
Corneal stromal keratocytes	Mfap5	-0.089168668	0.453697909	6.84E-08	1.84E-07
Corneal stromal keratocytes	Pltp	-0.088757735	0.186918443	3.22E-32	4.87E-31
Corneal stromal keratocytes	Enho	-0.088133537	0.180986548	1.29E-26	1.38E-25
Corneal stromal keratocytes	Crip2	-0.087380974	0.259913996	1.46E-16	7.93E-16
Corneal stromal keratocytes	Sptssa	-0.087211795	0.45082577	9.20E-21	6.76E-20
Corneal stromal keratocytes	Ccnd3	-0.086903173	0.244707123	7.81E-29	9.57E-28
Corneal stromal keratocytes	Pdgfa	-0.085638472	0.336330194	1.37E-22	1.13E-21
Corneal stromal keratocytes	Snhg12	-0.085479075	0.393340692	6.21E-24	5.65E-23
Corneal stromal keratocytes	Eif4e2	-0.085219564	0.392412718	6.11E-22	4.86E-21
Corneal stromal keratocytes	Zyx	-0.084544108	0.432609958	2.11E-16	1.13E-15
Corneal stromal keratocytes	Ccrl2	-0.084289962	0.14943057	9.51E-23	8.01E-22
Corneal stromal keratocytes	Rgs2	-0.082417346	0.719193531	7.53E-15	3.60E-14
Corneal stromal keratocytes	Tagln2	-0.081940987	0.398347392	1.47E-14	6.89E-14
Corneal stromal keratocytes	Notch2	-0.081914536	0.975120825	1.02E-08	2.98E-08
Corneal stromal keratocytes	Thbd	-0.081230049	0.196836367	7.21E-17	4.03E-16
Corneal stromal keratocytes	Rgs3	-0.080952135	0.241308803	2.76E-13	1.17E-12
Corneal stromal keratocytes	Tgfb1	-0.080549974	0.580263771	2.68E-18	1.65E-17
Corneal stromal keratocytes	Zfand5	-0.080084422	0.736163148	1.17E-12	4.72E-12
Corneal stromal keratocytes	Dusp1	-0.079213918	0.687722186	2.19E-08	6.17E-08

Corneal stromal keratocytes	Ldhb	-0.078781857	0.153957862	1.78E-28	2.13E-27
Corneal stromal keratocytes	Dbp	-0.077171275	0.452855988	1.39E-07	3.64E-07
Corneal stromal keratocytes	Zfp703	-0.076970104	0.723367551	4.74E-12	1.82E-11
Corneal stromal keratocytes	Gsta4	-0.076635243	0.253437022	4.93E-20	3.43E-19
Corneal stromal keratocytes	Lxn	-0.07658034	0.131136091	3.87E-30	5.12E-29
Corneal stromal keratocytes	Blvrb	-0.075492391	0.279291177	3.98E-17	2.27E-16
Corneal stromal keratocytes	Pja1	-0.075259164	0.29622157	6.81E-20	4.69E-19
Corneal stromal keratocytes	Ngf	-0.074555398	0.502887818	2.37E-07	6.05E-07
Corneal stromal keratocytes	Ifit3	-0.073768722	0.189059514	9.83E-21	7.20E-20
Corneal stromal keratocytes	Klf4	-0.072491691	1.05678396	2.51E-13	1.07E-12
Corneal stromal keratocytes	Rab3ip	-0.072313279	0.128711723	6.61E-27	7.20E-26
Corneal stromal keratocytes	Myl9	-0.071613929	0.283678677	2.46E-19	1.64E-18
Corneal stromal keratocytes	Lyar	-0.071188077	0.337115682	1.41E-20	1.02E-19
Corneal stromal keratocytes	Tuba1c	-0.071073083	0.375875234	2.95E-16	1.57E-15
Corneal stromal keratocytes	Fgf21	-0.070252063	0.531409626	0.0003089	0.000561087
Corneal stromal keratocytes	Ypel5	-0.070236982	0.302570561	4.24E-14	1.92E-13
Corneal stromal keratocytes	Ap4s1	-0.069907976	0.239130082	4.77E-21	3.57E-20
Corneal stromal keratocytes	Dusp9	-0.067689936	0.129896913	3.93E-14	1.78E-13
Corneal stromal keratocytes	Pold4	-0.067368724	0.248342047	7.51E-17	4.19E-16
Corneal stromal keratocytes	Ptn	-0.067225314	0.436352832	2.24E-25	2.22E-24
Corneal stromal keratocytes	Vcam1	-0.066948825	0.948988226	8.73E-20	5.98E-19
Corneal stromal keratocytes	Vamp3	-0.066891994	0.319693654	1.25E-16	6.82E-16
Corneal stromal keratocytes	Slc39a1	-0.066255523	0.776150074	1.19E-10	4.11E-10
Corneal stromal keratocytes	2210016F16Rik	-0.065667409	0.118761148	7.60E-23	6.45E-22
Corneal stromal keratocytes	Cldn10	-0.065249399	0.147804704	6.47E-12	2.46E-11
Corneal stromal keratocytes	Plk2	-0.065150595	0.486178069	1.67E-10	5.68E-10
Corneal stromal keratocytes	Lncpint	-0.065107934	0.168334526	8.74E-16	4.50E-15
Corneal stromal keratocytes	Clu	-0.064463317	0.403610037	9.80E-23	8.24E-22
Corneal stromal keratocytes	Palld	-0.064211135	0.195113903	1.39E-15	7.03E-15
Corneal stromal keratocytes	Ccdc3	-0.063568003	0.14887805	3.74E-33	5.98E-32
Corneal stromal keratocytes	Tmem126a	-0.063492459	0.279302385	7.76E-21	5.73E-20
Corneal stromal keratocytes	Serhl	-0.062304452	0.095131383	6.49E-24	5.88E-23
Corneal stromal keratocytes	Josd2	-0.061992545	0.177460403	7.01E-22	5.57E-21
Corneal stromal keratocytes	Mkrn1	-0.061254794	0.249908459	1.43E-18	8.98E-18
Corneal stromal keratocytes	Ccl19	-0.061001961	0.141151844	1.21E-19	8.21E-19
Corneal stromal keratocytes	Gamt	-0.060751392	0.136213299	9.58E-24	8.58E-23
Corneal stromal keratocytes	Rap2b	-0.059346413	0.220312741	1.33E-14	6.23E-14
Corneal stromal keratocytes	Cyfip1	-0.05895592	0.20897807	1.31E-21	1.02E-20
Corneal stromal keratocytes	Apex1	-0.058562066	0.165671122	1.12E-16	6.16E-16
Corneal stromal keratocytes	Klf7	-0.057310669	0.168956146	6.52E-19	4.21E-18
Corneal stromal keratocytes	Capg	-0.057129436	0.310598273	6.56E-11	2.31E-10
Corneal stromal keratocytes	Nfic	-0.056679599	0.328606828	7.39E-23	6.27E-22
Corneal stromal keratocytes	Phb	-0.055510899	0.295954781	2.39E-17	1.38E-16
Corneal stromal keratocytes	Cpe	-0.055435549	0.141668128	6.06E-28	7.03E-27
Corneal stromal keratocytes	Pnrc1	-0.055005987	0.62430502	5.31E-21	3.96E-20
Corneal stromal keratocytes	Dctpp1	-0.054574286	0.108048688	7.44E-23	6.31E-22
Corneal stromal keratocytes	Rgs7bp	-0.053937788	0.270430133	4.93E-11	1.75E-10
Corneal stromal keratocytes	Ptpre	-0.05352894	0.141951929	5.21E-14	2.33E-13
Corneal stromal keratocytes	Gadd45g	-0.053404898	0.4297941	2.76E-19	1.83E-18
Corneal stromal keratocytes	Dnajc7	-0.053156536	0.1766768	9.34E-18	5.57E-17
Corneal stromal keratocytes	Amdhd2	-0.053108975	0.084083548	2.27E-28	2.70E-27
Corneal stromal keratocytes	Hsp90aa1	-0.052997806	1.9225048	3.90E-08	1.07E-07
Corneal stromal keratocytes	Dclk1	-0.052888787	0.230143863	2.65E-20	1.88E-19
Corneal stromal keratocytes	Map1b	-0.052401448	0.193729228	3.62E-17	2.07E-16
Corneal stromal keratocytes	Rrp15	-0.052348642	0.299042495	2.26E-17	1.31E-16
Corneal stromal keratocytes	Sesn3	-0.052142096	0.239012574	7.81E-16	4.04E-15
Corneal stromal keratocytes	Mpp7	-0.051908018	0.112552316	4.35E-19	2.84E-18
Corneal stromal keratocytes	Osgin1	-0.051856731	0.181513908	2.56E-05	5.27E-05
Corneal stromal keratocytes	Atg10	-0.051490246	0.408842139	2.23E-16	1.20E-15
Corneal stromal keratocytes	Glrx	-0.050189946	0.153693949	5.69E-10	1.85E-09
Corneal stromal keratocytes	Tpm2	-0.049471725	0.294831354	3.14E-13	1.32E-12
Corneal stromal keratocytes	Olfm2	-0.049247375	0.356979327	0.0053785	0.008326496
Corneal stromal keratocytes	Exoc7	-0.048963962	0.088234869	1.19E-18	7.53E-18

Corneal stromal keratocytes	Aspscr1	-0.048898124	0.094469952	1.38E-19	9.35E-19
Corneal stromal keratocytes	Sgk1	-0.048755546	0.534476183	1.27E-07	3.33E-07
Corneal stromal keratocytes	Rsrp1	-0.048304614	0.417217375	3.25E-14	1.48E-13
Corneal stromal keratocytes	Rhob	-0.047926099	0.270424163	2.68E-09	8.22E-09
Corneal stromal keratocytes	Palm	-0.047744281	0.114972926	1.16E-22	9.73E-22
Corneal stromal keratocytes	Otub1	-0.047339717	0.201853821	3.82E-14	1.73E-13
Corneal stromal keratocytes	Gdnf	-0.04711048	0.13902738	6.64E-16	3.45E-15
Corneal stromal keratocytes	Top1	-0.047070379	2.368808707	3.63E-16	1.92E-15
Corneal stromal keratocytes	Adra2c	-0.0468233	0.045727678	4.25E-27	4.70E-26
Corneal stromal keratocytes	Klf13	-0.046641209	0.238712646	9.31E-18	5.55E-17
Corneal stromal keratocytes	Sumo3	-0.046629074	0.235798812	9.52E-14	4.17E-13
Corneal stromal keratocytes	Ttc14	-0.045958611	0.344487286	6.96E-13	2.85E-12
Corneal stromal keratocytes	Zpr1	-0.045305494	0.166814828	5.19E-16	2.72E-15
Corneal stromal keratocytes	Armcx2	-0.044955867	0.205453182	1.26E-10	4.32E-10
Corneal stromal keratocytes	Cdo1	-0.044492874	0.152478015	2.98E-16	1.58E-15
Corneal stromal keratocytes	Relb	-0.04415175	0.102277003	6.10E-17	3.43E-16
Corneal stromal keratocytes	Grpel2	-0.043976833	0.123898753	7.52E-17	4.19E-16
Corneal stromal keratocytes	Srsf3	-0.043222312	0.587085439	3.72E-10	1.23E-09
Corneal stromal keratocytes	Rgs16	-0.042756955	0.219053552	1.23E-08	3.56E-08
Corneal stromal keratocytes	Slc7a11	-0.042475232	0.159735164	5.44E-08	1.48E-07
Corneal stromal keratocytes	Oasl2	-0.041852255	0.110751936	2.61E-15	1.29E-14
Corneal stromal keratocytes	Id2	-0.041358153	0.608434344	2.09E-19	1.40E-18
Corneal stromal keratocytes	Irf7	-0.041111994	0.101600003	1.14E-17	6.77E-17
Corneal stromal keratocytes	Mipep	-0.04086676	0.142999026	9.52E-09	2.79E-08
Corneal stromal keratocytes	Desi1	-0.040749314	0.204374151	7.68E-10	2.46E-09
Corneal stromal keratocytes	Igtp	-0.040694148	0.123896687	1.62E-07	4.21E-07
Corneal stromal keratocytes	Otor	-0.040341997	0.086622385	6.58E-26	6.69E-25
Corneal stromal keratocytes	Atg3	-0.040290355	0.262793508	7.38E-15	3.53E-14
Corneal stromal keratocytes	Plin2	-0.040048685	0.104763298	9.52E-10	3.02E-09
Corneal stromal keratocytes	Vsnl1	-0.040045273	0.040946636	7.91E-21	5.82E-20
Corneal stromal keratocytes	Nfkbil1	-0.039641416	0.130604044	2.51E-11	9.08E-11
Corneal stromal keratocytes	Ube2e1	-0.039411406	0.310832009	3.08E-13	1.30E-12
Corneal stromal keratocytes	D10Wsu102e	-0.039368281	0.360203008	1.06E-06	2.53E-06
Corneal stromal keratocytes	Tlcd2	-0.038970545	0.098322723	1.61E-16	8.74E-16
Corneal stromal keratocytes	Dusp14	-0.038854474	0.330582178	0.0011373	0.001924618
Corneal stromal keratocytes	Trpm1	-0.038741517	0.074731817	5.84E-11	2.06E-10
Corneal stromal keratocytes	Sephs2	-0.038695926	0.096232369	4.12E-19	2.70E-18
Corneal stromal keratocytes	Procr	-0.038468357	0.257949336	0.0029738	0.004772501
Corneal stromal keratocytes	Sbds	-0.037968637	0.31720169	1.86E-19	1.25E-18
Corneal stromal keratocytes	Rgcc	-0.037798022	1.330320051	1.38E-21	1.07E-20
Corneal stromal keratocytes	Smim13	-0.037775207	0.163604882	4.10E-11	1.47E-10
Corneal stromal keratocytes	Nbl1	-0.037641511	0.495669487	7.97E-05	0.000154771
Corneal stromal keratocytes	Ang	-0.036934448	0.046112554	8.46E-19	5.42E-18
Corneal stromal keratocytes	Rassf1	-0.036732332	0.263202269	2.16E-09	6.68E-09
Corneal stromal keratocytes	Sltm	-0.03633295	0.445252177	1.29E-19	8.71E-19
Corneal stromal keratocytes	Ccdc102a	-0.036316572	0.06584818	2.14E-17	1.25E-16
Corneal stromal keratocytes	Arl4a	-0.035853835	0.238403206	4.30E-10	1.41E-09
Corneal stromal keratocytes	Tbcc	-0.035704587	0.326170796	4.62E-18	2.82E-17
Corneal stromal keratocytes	Htr1d	-0.035694357	0.028226855	7.16E-22	5.67E-21
Corneal stromal keratocytes	Tubb2a	-0.035690861	0.268025348	1.34E-16	7.29E-16
Corneal stromal keratocytes	Macrod1	-0.035665417	0.078146655	3.31E-13	1.39E-12
Corneal stromal keratocytes	Bex4	-0.035464486	0.026394079	1.39E-24	1.32E-23
Corneal stromal keratocytes	Ltf	-0.035235169	0.036854238	2.22E-13	9.47E-13
Corneal stromal keratocytes	Hist1h1e	-0.03521337	0.198797366	6.43E-06	1.41E-05
Corneal stromal keratocytes	Ak2	-0.035157898	0.242953186	4.12E-13	1.72E-12
Corneal stromal keratocytes	Spsb2	-0.035077288	0.112866094	7.45E-11	2.61E-10
Corneal stromal keratocytes	Ubtf	-0.035011364	0.280473854	6.72E-08	1.81E-07
Corneal stromal keratocytes	Tpgs2	-0.034840916	0.090209846	1.62E-16	8.78E-16
Corneal stromal keratocytes	Ddit3	-0.034609024	0.358243839	0.0005213	0.000921747
Corneal stromal keratocytes	Vamp5	-0.034415238	0.101140043	2.52E-12	9.89E-12
Corneal stromal keratocytes	Gstp1	-0.034136742	0.265312318	1.31E-08	3.78E-08
Corneal stromal keratocytes	Car3	-0.033977633	0.037845175	8.92E-05	0.00017215
Corneal stromal keratocytes	Il17rc	-0.033870483	0.070699575	1.63E-15	8.21E-15

Corneal stromal keratocytes	Cpne2	-0.033821433	0.136615117	0.0001674	0.000313135
Corneal stromal keratocytes	Fbxl15	-0.033139347	0.070096937	6.48E-12	2.46E-11
Corneal stromal keratocytes	Borcs6	-0.033032789	0.106425139	3.93E-13	1.64E-12
Corneal stromal keratocytes	Rhou	-0.0328154	0.11276102	3.79E-09	1.15E-08
Corneal stromal keratocytes	Hs3st5	-0.032779071	0.069195162	1.08E-15	5.53E-15
Corneal stromal keratocytes	Elf1	-0.032697524	0.316707054	9.42E-20	6.42E-19
Corneal stromal keratocytes	Hist1h1c	-0.032312066	0.128203184	0.0025795	0.00418115
Corneal stromal keratocytes	Rbm25	-0.031440315	0.528505955	3.16E-22	2.56E-21
Corneal stromal keratocytes	1500015O10Rik	-0.031138737	0.198036334	2.42E-16	1.30E-15
Corneal stromal keratocytes	Glyat	-0.031007429	0.052676586	4.14E-11	1.48E-10
Corneal stromal keratocytes	1110004E09Rik	-0.030958079	0.138259418	7.98E-11	2.79E-10
Corneal stromal keratocytes	3830406C13Rik	-0.030787444	0.137407825	2.32E-11	8.43E-11
Corneal stromal keratocytes	Spata7	-0.030767477	0.063853467	5.98E-08	1.62E-07
Corneal stromal keratocytes	Nrip1	-0.030656928	0.332526178	2.22E-11	8.06E-11
Corneal stromal keratocytes	Mras	-0.030543081	0.255523149	0.0121522	0.017924426
Corneal stromal keratocytes	Npy	-0.030359712	0.145165365	0.0027708	0.004471255
Corneal stromal keratocytes	Mtap	-0.030221887	0.095779506	2.69E-10	8.99E-10
Corneal stromal keratocytes	Tyms	-0.029781928	0.057855098	6.17E-11	2.18E-10
Corneal stromal keratocytes	Metrnl	-0.029567112	0.42647722	3.95E-06	8.82E-06
Corneal stromal keratocytes	Gadd45a	-0.029523403	0.729101898	5.42E-07	1.33E-06
Corneal stromal keratocytes	Cyb5r3	-0.029467688	0.322439618	8.43E-16	4.35E-15
Corneal stromal keratocytes	Iws1	-0.029441196	0.159757435	9.58E-15	4.54E-14
Corneal stromal keratocytes	Ddx55	-0.029371438	0.049044236	3.40E-13	1.42E-12
Corneal stromal keratocytes	Rcor1	-0.029105489	0.215775231	5.12E-11	1.82E-10
Corneal stromal keratocytes	Gipc1	-0.028913656	0.07147745	1.90E-09	5.89E-09
Corneal stromal keratocytes	Telo2	-0.028912791	0.046708683	7.12E-14	3.15E-13
Corneal stromal keratocytes	Mmp12	-0.028791685	0.035841909	0.0025381	0.004118811
Corneal stromal keratocytes	Kcns1	-0.02868663	0.085380807	3.46E-09	1.05E-08
Corneal stromal keratocytes	Cbx6	-0.028573081	0.213644493	3.59E-12	1.39E-11
Corneal stromal keratocytes	Clvs1	-0.028513045	0.353043052	8.26E-14	3.64E-13
Corneal stromal keratocytes	Rom1	-0.028424016	0.084372933	1.72E-12	6.82E-12
Corneal stromal keratocytes	Pfdn6	-0.028360131	0.284460451	2.86E-10	9.52E-10
Corneal stromal keratocytes	Vat1	-0.028214211	0.429549821	3.83E-11	1.37E-10
Corneal stromal keratocytes	Trappc9	-0.028041302	0.108738528	1.31E-10	4.50E-10
Corneal stromal keratocytes	Ttl	-0.028029701	0.069496446	9.86E-17	5.44E-16
Corneal stromal keratocytes	Mrps6	-0.027971995	0.147713847	3.53E-05	7.15E-05
Corneal stromal keratocytes	Rangrf	-0.027967467	0.052894535	2.49E-10	8.35E-10
Corneal stromal keratocytes	Tmem158	-0.027660689	0.117157876	1.06E-10	3.65E-10
Corneal stromal keratocytes	Cdkn1a	-0.027571059	1.613185235	7.79E-13	3.18E-12
Corneal stromal keratocytes	Mgll	-0.02752769	0.101389715	2.18E-07	5.59E-07
Corneal stromal keratocytes	Sox4	-0.027481946	0.169711499	6.29E-11	2.22E-10
Corneal stromal keratocytes	Bola1	-0.026582625	0.178023279	9.22E-08	2.45E-07
Corneal stromal keratocytes	Tnfaip6	-0.02637692	0.332864037	3.77E-08	1.04E-07
Corneal stromal keratocytes	Sox11	-0.026341553	0.046832342	2.30E-22	1.88E-21
Corneal stromal keratocytes	Khdrbs3	-0.026153958	0.150597729	1.23E-09	3.87E-09
Corneal stromal keratocytes	Ppp1r12c	-0.02601907	0.218636242	2.16E-10	7.28E-10
Corneal stromal keratocytes	Mgat4b	-0.025879586	0.077494231	8.86E-13	3.60E-12
Corneal stromal keratocytes	Cdc27	-0.025857595	0.128491066	2.17E-06	5.00E-06
Corneal stromal keratocytes	Rbm18	-0.025853594	0.265767745	1.82E-14	8.44E-14
Corneal stromal keratocytes	Taf12	-0.025843342	0.1549195	4.16E-12	1.61E-11
Corneal stromal keratocytes	Clec3b	-0.025841177	0.111591159	7.02E-10	2.26E-09
Corneal stromal keratocytes	Tysnd1	-0.025797083	0.054207101	5.30E-11	1.88E-10
Corneal stromal keratocytes	S1pr3	-0.02576577	0.11882507	4.83E-07	1.19E-06
Corneal stromal keratocytes	Myd88	-0.025677286	0.137599592	6.49E-12	2.47E-11
Corneal stromal keratocytes	Ccdc80	-0.02565078	0.252420586	6.46E-17	3.62E-16
Corneal stromal keratocytes	Chd1	-0.025363941	0.287090024	3.78E-17	2.16E-16
Corneal stromal keratocytes	Sfn	-0.025170087	0.097635388	3.08E-24	2.86E-23
Corneal stromal keratocytes	Lmo7	-0.025056502	0.114676479	2.16E-05	4.48E-05
Corneal stromal keratocytes	Qtrt1	-0.024530119	0.059124254	2.23E-08	6.29E-08
Corneal stromal keratocytes	Asf1a	-0.024343413	0.133461299	1.82E-07	4.70E-07
Corneal stromal keratocytes	Letm1	-0.024047981	0.179676974	5.07E-11	1.80E-10
Corneal stromal keratocytes	Diablo	-0.024019551	0.105705315	1.34E-10	4.58E-10
Corneal stromal keratocytes	Fah	-0.023910133	0.067127192	7.58E-13	3.09E-12

Corneal stromal keratocytes	Psmb8	-0.023847336	0.091810855	3.19E-09	9.70E-09
Corneal stromal keratocytes	Hspa2	-0.023731977	0.192134819	8.40E-06	1.82E-05
Corneal stromal keratocytes	Abracl	-0.023713192	0.380459639	1.30E-10	4.47E-10
Corneal stromal keratocytes	Zfp148	-0.023508587	0.262060097	6.24E-08	1.69E-07
Corneal stromal keratocytes	Rap1gap	-0.023451115	0.052741038	5.99E-09	1.79E-08
Corneal stromal keratocytes	Emc2	-0.023372718	0.110673867	3.88E-07	9.67E-07
Corneal stromal keratocytes	Lrrc42	-0.023312504	0.08531429	1.07E-10	3.69E-10
Corneal stromal keratocytes	Tox4	-0.023252733	0.288296311	5.55E-17	3.12E-16
Corneal stromal keratocytes	Gna14	-0.0232167	0.028198178	2.33E-15	1.16E-14
Corneal stromal keratocytes	Zfp950	-0.023055195	0.073790969	2.54E-06	5.82E-06
Corneal stromal keratocytes	Csrp1	-0.023012108	0.161428222	1.20E-10	4.12E-10
Corneal stromal keratocytes	Skil	-0.022850134	0.590310206	2.44E-10	8.19E-10
Corneal stromal keratocytes	Etnk1	-0.022796518	0.287090148	2.13E-08	6.02E-08
Corneal stromal keratocytes	Yae1d1	-0.022629605	0.267697425	3.87E-16	2.04E-15
Corneal stromal keratocytes	Fem1b	-0.022330495	0.415072924	1.02E-16	5.64E-16
Corneal stromal keratocytes	Cxxc1	-0.02180494	0.109915884	1.20E-08	3.48E-08
Corneal stromal keratocytes	Nop9	-0.021703263	0.094071471	1.53E-08	4.38E-08
Corneal stromal keratocytes	Nsmf	-0.021608264	0.103313562	1.61E-08	4.62E-08
Corneal stromal keratocytes	Isg15	-0.021450396	0.145450584	5.23E-05	0.000103923
Corneal stromal keratocytes	Vmac	-0.021412885	0.047685174	1.36E-07	3.56E-07
Corneal stromal keratocytes	Cav1	-0.021258038	0.133979133	2.77E-05	5.67E-05
Corneal stromal keratocytes	Fndc4	-0.020881361	0.279851236	4.44E-10	1.45E-09
Corneal stromal keratocytes	Txnrd1	-0.020878258	1.149003256	9.58E-16	4.92E-15
Corneal stromal keratocytes	Ctdspl	-0.020823557	0.099778304	2.98E-05	6.09E-05
Corneal stromal keratocytes	Krt15	-0.020692201	0.012052124	3.14E-21	2.38E-20
Corneal stromal keratocytes	Pcdh17	-0.020598477	0.055492984	1.42E-10	4.85E-10
Corneal stromal keratocytes	Zfp277	-0.020302344	0.049787339	5.14E-05	0.000102322
Corneal stromal keratocytes	Mrpl3	-0.02019987	0.133664468	1.70E-08	4.86E-08
Corneal stromal keratocytes	Tsc22d1	-0.019938307	0.575484599	1.49E-05	3.15E-05
Corneal stromal keratocytes	Znrf1	-0.019795866	0.144973011	1.44E-07	3.77E-07
Corneal stromal keratocytes	Stk17b	-0.019660841	0.174353811	2.88E-12	1.12E-11
Corneal stromal keratocytes	Ppa1	-0.019568439	0.683399308	3.04E-16	1.62E-15
Corneal stromal keratocytes	Mt3	-0.019556981	0.046088312	0.0002009	0.000372924
Corneal stromal keratocytes	Nat14	-0.019556244	0.020701599	6.43E-10	2.07E-09
Corneal stromal keratocytes	Hipk2	-0.019519513	0.064251471	7.42E-09	2.20E-08
Corneal stromal keratocytes	Ccdc94	-0.01949227	0.058493726	1.29E-09	4.06E-09
Corneal stromal keratocytes	Zfp704	-0.019486271	0.100325056	5.53E-10	1.80E-09
Corneal stromal keratocytes	Tcim	-0.019391985	0.039027456	1.01E-05	2.17E-05
Corneal stromal keratocytes	Fbxo22	-0.019391545	0.087687911	5.41E-05	0.000107255
Corneal stromal keratocytes	Foxs1	-0.019382447	0.030705331	1.51E-07	3.94E-07
Corneal stromal keratocytes	Chka	-0.019271313	0.110003829	8.73E-09	2.57E-08
Corneal stromal keratocytes	Ptgir	-0.019248528	0.329067821	0.0006784	0.001179469
Corneal stromal keratocytes	Purg	-0.019230292	0.048606327	2.17E-09	6.69E-09
Corneal stromal keratocytes	Myg1	-0.019191013	0.04840797	7.05E-07	1.71E-06
Corneal stromal keratocytes	Ppih	-0.019163653	0.040059904	3.52E-09	1.07E-08
Corneal stromal keratocytes	Tfpt	-0.019159625	0.081551501	5.57E-09	1.67E-08
Corneal stromal keratocytes	Acvrl1	-0.019041477	0.077229577	3.16E-13	1.33E-12
Corneal stromal keratocytes	Rarg	-0.018876689	0.130145329	1.81E-05	3.78E-05
Corneal stromal keratocytes	Adrb2	-0.018861488	0.030611279	9.34E-07	2.24E-06
Corneal stromal keratocytes	Col14a1	-0.018850066	0.378161556	1.57E-20	1.13E-19
Corneal stromal keratocytes	Efnb1	-0.018803788	0.331029041	2.53E-11	9.16E-11
Corneal stromal keratocytes	Snx29	-0.018717728	0.078005579	0.0027886	0.004498303
Corneal stromal keratocytes	Dnm2	-0.018494082	0.180367142	3.54E-11	1.27E-10
Corneal stromal keratocytes	Rnd2	-0.018381978	0.093620911	4.03E-05	8.10E-05
Corneal stromal keratocytes	Hddc2	-0.01836417	0.073637308	2.65E-08	7.41E-08
Corneal stromal keratocytes	Krt12	-0.018304866	0.17365261	5.38E-56	2.30E-54
Corneal stromal keratocytes	Mapk12	-0.018198728	0.046593889	7.52E-08	2.02E-07
Corneal stromal keratocytes	Qpctl	-0.01817766	0.049032941	5.44E-07	1.33E-06
Corneal stromal keratocytes	Akap8l	-0.017963671	0.112234811	4.36E-11	1.56E-10
Corneal stromal keratocytes	Gphn	-0.017949347	0.077857694	2.40E-10	8.06E-10
Corneal stromal keratocytes	Fam117a	-0.017940287	0.044974951	1.79E-05	3.74E-05
Corneal stromal keratocytes	Cd248	-0.017911459	0.131991209	1.77E-08	5.05E-08
Corneal stromal keratocytes	Med24	-0.017871694	0.090150592	6.99E-06	1.53E-05

Corneal stromal keratocytes	Pdcd2l	-0.017831733	0.073631325	5.15E-06	1.14E-05
Corneal stromal keratocytes	3110039I08Rik	-0.017773561	0.399954112	2.19E-08	6.17E-08
Corneal stromal keratocytes	Coq10a	-0.017736668	0.040642971	1.86E-08	5.29E-08
Corneal stromal keratocytes	Rbm8a	-0.017729458	0.287948391	1.50E-10	5.12E-10
Corneal stromal keratocytes	Tmem38a	-0.017723091	0.042199051	1.92E-09	5.97E-09
Corneal stromal keratocytes	Acbd4	-0.01762563	0.05404432	2.25E-07	5.75E-07
Corneal stromal keratocytes	Ms4a4d	-0.017618384	0.033590336	1.54E-06	3.60E-06
Corneal stromal keratocytes	Eef1akmt2	-0.017542864	0.118758649	0.000612	0.001070561
Corneal stromal keratocytes	Mypop	-0.017479101	0.019932506	5.00E-11	1.77E-10
Corneal stromal keratocytes	Socs1	-0.017473649	0.292476679	0.0046711	0.007292096
Corneal stromal keratocytes	Ube2e2	-0.017412896	0.153638144	7.21E-06	1.57E-05
Corneal stromal keratocytes	2810001G20Rik	-0.017343834	0.069624649	5.14E-07	1.27E-06
Corneal stromal keratocytes	Dcaf11	-0.017335043	0.135225665	5.79E-10	1.88E-09
Corneal stromal keratocytes	1600014C10Rik	-0.017013378	0.051028304	2.04E-14	9.43E-14
Corneal stromal keratocytes	Rfng	-0.016866169	0.061482124	2.62E-05	5.39E-05
Corneal stromal keratocytes	Yod1	-0.01684108	0.146222455	5.94E-06	1.31E-05
Corneal stromal keratocytes	Spin2c	-0.016780187	0.041242228	0.0002153	0.000398458
Corneal stromal keratocytes	Comt	-0.016740758	0.137701691	7.43E-11	2.61E-10
Corneal stromal keratocytes	Rbbp8	-0.016728095	0.054117792	1.81E-11	6.62E-11
Corneal stromal keratocytes	Sult1a1	-0.016698071	0.030353023	9.36E-09	2.75E-08
Corneal stromal keratocytes	Blvra	-0.016654831	0.039992903	1.32E-06	3.13E-06
Corneal stromal keratocytes	Hps1	-0.016563507	0.032052592	4.94E-08	1.35E-07
Corneal stromal keratocytes	Kpna4	-0.01654232	0.260362004	7.55E-17	4.20E-16
Corneal stromal keratocytes	Pmel	-0.016501361	0.023587872	1.33E-06	3.13E-06
Corneal stromal keratocytes	Zfp524	-0.0164371	0.02935371	2.68E-08	7.48E-08
Corneal stromal keratocytes	Akr1b8	-0.016404609	0.046757256	0.00677	0.010325244
Corneal stromal keratocytes	Nrtn	-0.01633638	0.045481254	2.41E-06	5.54E-06
Corneal stromal keratocytes	6030419C18Rik	-0.016252019	0.053042061	1.01E-05	2.16E-05
Corneal stromal keratocytes	Ccdc136	-0.016250975	0.084701626	0.0001437	0.000270984
Corneal stromal keratocytes	Pard6a	-0.016233958	0.086974988	5.33E-05	0.00010572
Corneal stromal keratocytes	Thoc6	-0.016209514	0.052096847	3.85E-09	1.17E-08
Corneal stromal keratocytes	Ctsc	-0.016168962	0.087629999	3.61E-08	9.95E-08
Corneal stromal keratocytes	Dcps	-0.016102483	0.031147437	5.43E-12	2.08E-11
Corneal stromal keratocytes	Ube2d1	-0.01609009	0.060739139	1.98E-06	4.58E-06
Corneal stromal keratocytes	Ccl8	-0.015997723	0.046804304	0.0147668	0.021527667
Corneal stromal keratocytes	H1f0	-0.015938331	0.150573242	2.00E-07	5.15E-07
Corneal stromal keratocytes	Gsr	-0.015867268	0.090267856	3.44E-06	7.73E-06
Corneal stromal keratocytes	Fosl1	-0.015791193	0.582344342	3.86E-09	1.17E-08
Corneal stromal keratocytes	Tap1	-0.015782004	0.125679629	2.93E-09	8.93E-09
Corneal stromal keratocytes	Fbrs	-0.015663078	0.091444108	4.81E-05	9.59E-05
Corneal stromal keratocytes	Ehd4	-0.015640469	0.190466181	4.22E-09	1.27E-08
Corneal stromal keratocytes	Ajap1	-0.015400826	0.041369396	9.51E-06	2.04E-05
Corneal stromal keratocytes	Tbc1d19	-0.015349903	0.051953801	1.68E-05	3.53E-05
Corneal stromal keratocytes	Hmg20a	-0.015302878	0.089183807	3.69E-10	1.22E-09
Corneal stromal keratocytes	Ccdc71l	-0.015275745	0.149610549	9.71E-06	2.08E-05
Corneal stromal keratocytes	Zfp146	-0.015259406	0.079960595	1.01E-05	2.16E-05
Corneal stromal keratocytes	Ifit3b	-0.015198835	0.055026677	7.49E-09	2.22E-08
Corneal stromal keratocytes	Angptl4	-0.015050068	0.028139026	4.13E-05	8.29E-05
Corneal stromal keratocytes	Tmem126b	-0.014969493	0.031975737	1.74E-06	4.05E-06
Corneal stromal keratocytes	Ppm1d	-0.014967193	0.052669783	0.000507	0.000897003
Corneal stromal keratocytes	Rnf181	-0.014964251	0.093548247	0.000304	0.000552717
Corneal stromal keratocytes	Trim47	-0.014959851	0.050970087	0.003099	0.004958659
Corneal stromal keratocytes	Lhfpl3	-0.014903277	0.012019574	6.17E-12	2.35E-11
Corneal stromal keratocytes	Tradd	-0.014883323	0.037649006	3.37E-07	8.48E-07
Corneal stromal keratocytes	Cxcl16	-0.014881927	0.077843333	5.81E-13	2.39E-12
Corneal stromal keratocytes	Scube2	-0.01486287	0.039830878	5.94E-07	1.45E-06
Corneal stromal keratocytes	Gadd45b	-0.014862091	0.816335332	6.83E-05	0.000133638
Corneal stromal keratocytes	Faap24	-0.014743923	0.02436356	0.0001135	0.000216515
Corneal stromal keratocytes	Sprr1a	-0.014719519	0.014444382	8.07E-12	3.04E-11
Corneal stromal keratocytes	Trmt5	-0.014697734	0.032358824	1.51E-07	3.93E-07
Corneal stromal keratocytes	Tmem175	-0.014669898	0.057920965	2.72E-08	7.62E-08
Corneal stromal keratocytes	Pgm2	-0.014668593	0.222005989	4.36E-09	1.31E-08
Corneal stromal keratocytes	Rcan1	-0.01450733	0.509834466	0.0294168	0.040917757

Corneal stromal keratocytes	Flnc	-0.014480124	0.218403242	7.54E-05	0.000146749
Corneal stromal keratocytes	Zfyve19	-0.014410827	0.029701971	6.61E-08	1.78E-07
Corneal stromal keratocytes	Ap4m1	-0.014381387	0.024012573	5.25E-09	1.57E-08
Corneal stromal keratocytes	Mknk2	-0.014316059	0.075574886	1.31E-06	3.09E-06
Corneal stromal keratocytes	Arid4b	-0.014231089	0.272022721	2.35E-11	8.54E-11
Corneal stromal keratocytes	Slc10a3	-0.014199385	0.029294252	2.71E-10	9.04E-10
Corneal stromal keratocytes	Pdlim2	-0.014111363	0.142631574	1.17E-12	4.72E-12
Corneal stromal keratocytes	Akip1	-0.014037557	0.059741552	0.0001416	0.000267043
Corneal stromal keratocytes	E030030I06Rik	-0.013992613	0.009668656	3.58E-12	1.39E-11
Corneal stromal keratocytes	Lgals3	-0.013972019	0.089941644	0.0018895	0.003113603
Corneal stromal keratocytes	Mettl15	-0.013958427	0.027699379	3.41E-05	6.92E-05
Corneal stromal keratocytes	Mto1	-0.01393065	0.039089716	4.67E-07	1.15E-06
Corneal stromal keratocytes	Ch25h	-0.013903844	0.120384703	0.0001195	0.000227432
Corneal stromal keratocytes	Inpp5f	-0.013863321	0.043241338	0.000101	0.000193662
Corneal stromal keratocytes	Nasp	-0.013798593	0.208932987	3.73E-12	1.44E-11
Corneal stromal keratocytes	Xylt2	-0.013750681	0.034176128	3.36E-07	8.44E-07
Corneal stromal keratocytes	Pttg1	-0.013642905	0.175149061	5.03E-07	1.24E-06
Corneal stromal keratocytes	Smarca2	-0.013488405	0.279795621	3.53E-14	1.61E-13
Corneal stromal keratocytes	Pycard	-0.013486261	0.044550303	5.10E-10	1.66E-09
Corneal stromal keratocytes	Wdr4	-0.013452651	0.095176207	1.51E-09	4.74E-09
Corneal stromal keratocytes	Aard	-0.013409145	0.040833539	0.0003208	0.000581712
Corneal stromal keratocytes	Uck2	-0.013357205	0.293218235	7.17E-09	2.13E-08
Corneal stromal keratocytes	Nelfcd	-0.013355402	0.021503003	3.21E-05	6.52E-05
Corneal stromal keratocytes	Lrrc32	-0.01332039	0.280592573	0.0002514	0.000461197
Corneal stromal keratocytes	Cks1b	-0.013319993	0.124395149	9.25E-05	0.000178068
Corneal stromal keratocytes	Irf9	-0.01331638	0.207442409	5.50E-07	1.35E-06
Corneal stromal keratocytes	Nmi	-0.013226554	0.070922734	5.44E-09	1.63E-08
Corneal stromal keratocytes	0610009B22Rik	-0.013215899	0.08767929	2.97E-07	7.50E-07
Corneal stromal keratocytes	Dleu2	-0.01319966	0.032687863	6.53E-08	1.76E-07
Corneal stromal keratocytes	Emid1	-0.01316066	0.011175338	1.22E-10	4.18E-10
Corneal stromal keratocytes	Gabarapl1	-0.01309397	0.344891981	1.20E-15	6.12E-15
Corneal stromal keratocytes	Nudcd1	-0.013078183	0.053343608	8.40E-06	1.82E-05
Corneal stromal keratocytes	Ankrd39	-0.012961786	0.050830714	2.94E-08	8.18E-08
Corneal stromal keratocytes	Cyp4f13	-0.012934226	0.043465557	1.65E-06	3.85E-06
Corneal stromal keratocytes	Higd1a	-0.012755481	1.770518699	7.76E-30	1.01E-28
Corneal stromal keratocytes	Nfil3	-0.012567522	0.088304055	1.76E-05	3.69E-05
Corneal stromal keratocytes	Retreg1	-0.012561051	0.030201858	0.0065304	0.009979371
Corneal stromal keratocytes	2410022M11Rik	-0.012530711	0.03086884	9.06E-05	0.000174715
Corneal stromal keratocytes	Gpr27	-0.012527418	0.044948153	6.09E-06	1.34E-05
Corneal stromal keratocytes	Ly6g6d	-0.01252594	0.016113264	6.88E-08	1.85E-07
Corneal stromal keratocytes	Pacs1	-0.01249425	0.046084733	1.75E-06	4.07E-06
Corneal stromal keratocytes	Ddhd2	-0.012491716	0.094305414	0.0001068	0.000204304
Corneal stromal keratocytes	Rbm15	-0.012476814	0.169351723	4.37E-08	1.20E-07
Corneal stromal keratocytes	Tet3	-0.012399589	0.238871118	3.35E-07	8.41E-07
Corneal stromal keratocytes	Optc	-0.012299922	0.018864962	5.55E-05	0.000109868
Corneal stromal keratocytes	Otud3	-0.012285323	0.091277654	0.0214442	0.030471233
Corneal stromal keratocytes	2700081O15Rik	-0.012276688	0.046888303	1.58E-07	4.12E-07
Corneal stromal keratocytes	Igfbp7	-0.012217236	2.422130446	2.62E-05	5.38E-05
Corneal stromal keratocytes	Plscr4	-0.012203019	0.048888916	1.09E-07	2.89E-07
Corneal stromal keratocytes	Sostdc1	-0.01216	0.01816036	4.08E-05	8.20E-05
Corneal stromal keratocytes	Rbm43	-0.012092291	0.032128022	0.0002396	0.000440407
Corneal stromal keratocytes	Lhfpl2	-0.012050097	0.455574756	1.39E-17	8.20E-17
Corneal stromal keratocytes	Tsc22d2	-0.012020342	0.326532177	1.36E-12	5.43E-12
Corneal stromal keratocytes	Apex2	-0.012008807	0.043058436	0.0002203	0.000407329
Corneal stromal keratocytes	Tspo	-0.011891055	0.475728451	9.36E-09	2.75E-08
Corneal stromal keratocytes	B230217C12Rik	-0.01185494	0.018549589	1.05E-05	2.25E-05
Corneal stromal keratocytes	Rasal2	-0.011830677	0.068234898	2.33E-13	9.93E-13
Corneal stromal keratocytes	S100a3	-0.01181758	0.020070393	0.0056092	0.008655451
Corneal stromal keratocytes	Polr3gl	-0.011814341	0.081631409	4.25E-09	1.28E-08
Corneal stromal keratocytes	Gas1	-0.011769975	0.308879221	1.00E-05	2.15E-05
Corneal stromal keratocytes	Gigyf1	-0.011752164	0.047513299	1.63E-09	5.08E-09
Corneal stromal keratocytes	Smco3	-0.011700498	0.028153796	2.38E-07	6.08E-07
Corneal stromal keratocytes	Sardh	-0.011656568	0.025352541	3.43E-05	6.96E-05

Corneal stromal keratocytes	Aptx	-0.011594285	0.065991599	1.37E-08	3.97E-08
Corneal stromal keratocytes	Ptgr1	-0.011502363	0.021842433	3.73E-06	8.37E-06
Corneal stromal keratocytes	Upk3bl	-0.011489143	0.023162798	6.29E-08	1.70E-07
Corneal stromal keratocytes	Faim	-0.011483817	0.09064731	1.04E-05	2.23E-05
Corneal stromal keratocytes	Clec14a	-0.011420531	0.030323677	8.77E-06	1.89E-05
Corneal stromal keratocytes	Eif2b2	-0.011404225	0.102996957	2.54E-07	6.46E-07
Corneal stromal keratocytes	Bbc3	-0.01140388	0.038850856	2.98E-11	1.08E-10
Corneal stromal keratocytes	Cd93	-0.011382395	0.041514091	8.00E-06	1.73E-05
Corneal stromal keratocytes	Tubg1	-0.011377368	0.032266318	6.66E-07	1.62E-06
Corneal stromal keratocytes	Calcoco1	-0.011359731	0.050730972	9.52E-05	0.000183042
Corneal stromal keratocytes	Tpd52l1	-0.011340005	0.052299412	1.68E-05	3.52E-05
Corneal stromal keratocytes	Tmx4	-0.011324994	0.234472281	8.10E-11	2.83E-10
Corneal stromal keratocytes	Six4	-0.011323053	0.039729693	9.56E-06	2.05E-05
Corneal stromal keratocytes	Phldb2	-0.011310603	0.223528022	5.88E-10	1.90E-09
Corneal stromal keratocytes	Trim25	-0.011303868	0.166616013	5.19E-07	1.28E-06
Corneal stromal keratocytes	Wdr35	-0.01123294	0.04025169	5.82E-08	1.58E-07
Corneal stromal keratocytes	H2-Q7	-0.011230655	0.03380009	1.53E-05	3.23E-05
Corneal stromal keratocytes	Ly6e	-0.011229261	0.228082895	4.80E-16	2.52E-15
Corneal stromal keratocytes	Klf5	-0.011222591	0.063265315	2.31E-07	5.90E-07
Corneal stromal keratocytes	Mgat4c	-0.011213037	0.015690258	9.61E-10	3.05E-09
Corneal stromal keratocytes	Gbp3	-0.011134485	0.052926659	2.10E-06	4.86E-06
Corneal stromal keratocytes	Slc25a35	-0.011083226	0.017934616	5.19E-09	1.56E-08
Corneal stromal keratocytes	Parp12	-0.011030206	0.065249046	1.20E-06	2.85E-06
Corneal stromal keratocytes	Lrrc75a	-0.010931109	0.095999968	7.31E-06	1.59E-05
Corneal stromal keratocytes	Rapsn	-0.010856391	0.021474041	0.0018584	0.00306512
Corneal stromal keratocytes	Tceal5	-0.010853294	0.036423157	0.0206483	0.029417684
Corneal stromal keratocytes	Tarbp2	-0.01082267	0.029453951	4.96E-06	1.10E-05
Corneal stromal keratocytes	Cactin	-0.010814766	0.065164723	7.00E-06	1.53E-05
Corneal stromal keratocytes	Tfpi2	-0.01081021	0.037929097	0.0005365	0.000947004
Corneal stromal keratocytes	Ccl27a	-0.010780594	0.073484175	0.0016907	0.002799975
Corneal stromal keratocytes	Mid1ip1	-0.010631763	0.08656353	0.0285803	0.039837078
Corneal stromal keratocytes	Sipa1	-0.010628023	0.035220212	1.18E-06	2.80E-06
Corneal stromal keratocytes	Batf	-0.010614852	0.033096617	0.0018532	0.003057162
Corneal stromal keratocytes	Cacng4	-0.010592491	0.013059152	1.17E-05	2.49E-05
Corneal stromal keratocytes	Ankrd13c	-0.01058877	0.208025787	4.33E-09	1.30E-08
Corneal stromal keratocytes	Crtc1	-0.010540347	0.035735953	0.0001596	0.000299067
Corneal stromal keratocytes	1110032F04Rik	-0.01048912	0.019547112	1.48E-06	3.47E-06
Corneal stromal keratocytes	Hic1	-0.010472017	0.066935242	0.0006593	0.001148682
Corneal stromal keratocytes	Abcg2	-0.010469336	0.038484438	1.21E-06	2.88E-06
Corneal stromal keratocytes	Klc3	-0.010460259	0.018917523	0.0003002	0.000546061
Corneal stromal keratocytes	Rita1	-0.010426035	0.030989469	8.74E-06	1.89E-05
Corneal stromal keratocytes	Katnb1	-0.010371281	0.022075625	0.0024425	0.003973765
Corneal stromal keratocytes	Gdpd1	-0.010365152	0.021836462	1.41E-05	2.97E-05
Corneal stromal keratocytes	Evi2a	-0.010340499	0.016334549	0.0150766	0.021945119
Corneal stromal keratocytes	Camk2n2	-0.010336535	0.038142464	4.31E-07	1.07E-06
Corneal stromal keratocytes	Dusp5	-0.010325719	0.232001255	0.0073887	0.011216501
Corneal stromal keratocytes	Slc4a11	-0.010299155	0.014246637	1.59E-06	3.72E-06
Corneal stromal keratocytes	Shd	-0.010202068	0.023491609	0.0043148	0.006771284
Corneal stromal keratocytes	Klk8	-0.010145311	0.00930804	1.32E-07	3.46E-07
Corneal stromal keratocytes	Tram1l1	-0.010098182	0.009877077	1.21E-08	3.52E-08
Corneal stromal keratocytes	Galk2	-0.010086899	0.054117494	0.0001062	0.000203289
Corneal stromal keratocytes	Mmp3	-0.010068626	0.380300435	0.0010194	0.001734695
Corneal stromal keratocytes	Twf2	-0.010042494	0.029506519	1.19E-06	2.84E-06
Corneal stromal keratocytes	44443	-0.009993339	0.032978529	0.0001028	0.000197075
Corneal stromal keratocytes	Mast4	-0.009989895	0.68934137	1.26E-08	3.65E-08
Corneal stromal keratocytes	Krt18	-0.009948936	0.018362433	0.0320117	0.044211725
Corneal stromal keratocytes	Mospd1	-0.009886497	0.058240283	0.0125123	0.018424638
Corneal stromal keratocytes	Abcb8	-0.009855892	0.040986157	3.78E-08	1.04E-07
Corneal stromal keratocytes	Dyrk3	-0.009842919	0.013935876	3.89E-05	7.83E-05
Corneal stromal keratocytes	Eya2	-0.009811393	0.022770105	2.60E-06	5.94E-06
Corneal stromal keratocytes	Pcdh7	-0.009785431	0.099372488	4.35E-08	1.19E-07
Corneal stromal keratocytes	Uri1	-0.009765653	0.157032319	3.64E-06	8.16E-06
Corneal stromal keratocytes	Gjc1	-0.009673809	0.088345091	2.24E-05	4.64E-05

Corneal stromal keratocytes	Krt222	-0.009671467	0.015975574	2.85E-05	5.83E-05
Corneal stromal keratocytes	Fam111a	-0.00952808	0.040153767	0.0020365	0.003343949
Corneal stromal keratocytes	Afdn	-0.009526281	0.110472585	4.55E-06	1.01E-05
Corneal stromal keratocytes	Rgs17	-0.009471333	0.02150655	1.04E-05	2.23E-05
Corneal stromal keratocytes	Ccdc39	-0.009410131	0.016078393	1.32E-05	2.79E-05
Corneal stromal keratocytes	N6amt1	-0.009361261	0.090327373	0.0001131	0.000215837
Corneal stromal keratocytes	Scnm1	-0.009319233	0.036362256	0.000122	0.000231853
Corneal stromal keratocytes	Kbtbd4	-0.009223543	0.041417934	0.0095081	0.014230743
Corneal stromal keratocytes	Hsf2	-0.00917358	0.041820117	6.36E-05	0.000124735
Corneal stromal keratocytes	Pced1a	-0.009118502	0.027786945	0.0052875	0.008201456
Corneal stromal keratocytes	Enkd1	-0.009023888	0.013388147	8.38E-06	1.81E-05
Corneal stromal keratocytes	Ints6	-0.008965909	0.050656886	1.89E-05	3.94E-05
Corneal stromal keratocytes	Vps9d1	-0.008962544	0.031327954	2.19E-05	4.55E-05
Corneal stromal keratocytes	Gjb4	-0.008955089	0.020440187	0.0023533	0.003839838
Corneal stromal keratocytes	Cdkn2aipnl	-0.008900212	0.028204738	4.61E-05	9.22E-05
Corneal stromal keratocytes	Nsun5	-0.008826315	0.041332242	8.97E-05	0.000173036
Corneal stromal keratocytes	Dct	-0.008778229	0.015741815	7.90E-06	1.71E-05
Corneal stromal keratocytes	Klc4	-0.008726119	0.033367585	0.0004088	0.000731119
Corneal stromal keratocytes	Mcoln1	-0.008720558	0.040776559	0.0036601	0.005798864
Corneal stromal keratocytes	Pvt1	-0.00871958	0.026264984	0.0025089	0.004076209
Corneal stromal keratocytes	Slc38a6	-0.008680434	0.024239242	0.0006014	0.00105412
Corneal stromal keratocytes	Trim24	-0.008671534	0.038274427	0.0015445	0.002570628
Corneal stromal keratocytes	Slc27a3	-0.008652826	0.015148007	0.0001605	0.000300785
Corneal stromal keratocytes	Itga7	-0.008581077	0.017446164	0.0030251	0.004849187
Corneal stromal keratocytes	Gprc5c	-0.008533711	0.017051046	0.000915	0.001565639
Corneal stromal keratocytes	Golga1	-0.008465513	0.059335211	3.39E-06	7.64E-06
Corneal stromal keratocytes	Lrch4	-0.008462267	0.021604344	0.0022176	0.003626822
Corneal stromal keratocytes	Nkd2	-0.008455952	0.413109187	2.60E-07	6.62E-07
Corneal stromal keratocytes	Tspan33	-0.008449226	0.013886572	1.20E-05	2.55E-05
Corneal stromal keratocytes	Nfatc2	-0.008444926	0.208261046	0.0062882	0.009634333
Corneal stromal keratocytes	Wdsub1	-0.008438114	0.030429552	0.0003749	0.000673626
Corneal stromal keratocytes	Yipf2	-0.008429389	0.049899151	6.71E-06	1.47E-05
Corneal stromal keratocytes	Zfp7	-0.008423034	0.013734399	0.0219108	0.031080672
Corneal stromal keratocytes	Ucp2	-0.008407205	0.020717433	1.44E-06	3.38E-06
Corneal stromal keratocytes	Fbxl17	-0.008341237	0.085345853	2.41E-06	5.53E-06
Corneal stromal keratocytes	Wdtc1	-0.008322001	0.138892145	8.52E-06	1.84E-05
Corneal stromal keratocytes	Nsf	-0.008301891	0.09300592	0.001603	0.002661036
Corneal stromal keratocytes	Zbed3	-0.00829296	0.058608769	2.19E-06	5.05E-06
Corneal stromal keratocytes	Limd1	-0.008271645	0.34999019	1.11E-07	2.94E-07
Corneal stromal keratocytes	Ogfrl1	-0.008253226	0.084577575	2.46E-05	5.08E-05
Corneal stromal keratocytes	Wfdc2	-0.008173062	0.012543247	0.0023645	0.00385726
Corneal stromal keratocytes	Cdc25a	-0.008156697	0.038645235	5.27E-06	1.17E-05
Corneal stromal keratocytes	Dlg2	-0.008150964	0.039467364	0.0010068	0.00171467
Corneal stromal keratocytes	Zfp945	-0.008125796	0.060809503	0.0001297	0.00024571
Corneal stromal keratocytes	Cck	-0.008111895	0.018835335	0.0041694	0.006559854
Corneal stromal keratocytes	Pold3	-0.008074566	0.049851808	0.0026359	0.004267262
Corneal stromal keratocytes	Txnrd2	-0.008010339	0.028293661	0.0046704	0.007291733
Corneal stromal keratocytes	Spint2	-0.007993155	0.016061371	5.92E-08	1.60E-07
Corneal stromal keratocytes	Syn2	-0.007975783	0.011493961	0.000403	0.000721365
Corneal stromal keratocytes	Evc	-0.007959274	0.063314633	1.18E-05	2.51E-05
Corneal stromal keratocytes	Lrwd1	-0.007933205	0.024521256	0.0002418	0.000444273
Corneal stromal keratocytes	Fxyd5	-0.007883158	0.121335818	6.74E-08	1.82E-07
Corneal stromal keratocytes	C030013C21Rik	-0.007853232	0.021326854	0.0031096	0.004973383
Corneal stromal keratocytes	Tbl3	-0.007798268	0.063959084	5.47E-05	0.000108304
Corneal stromal keratocytes	Rpain	-0.00779555	0.062559082	0.0050413	0.007835133
Corneal stromal keratocytes	Zfp120	-0.007777561	0.039078115	0.0147768	0.021539952
Corneal stromal keratocytes	Ebf4	-0.007711589	0.024103133	0.002029	0.003332308
Corneal stromal keratocytes	Wdr60	-0.007685929	0.030076731	1.57E-06	3.69E-06
Corneal stromal keratocytes	Capn5	-0.007650188	0.045707905	0.0019284	0.003174589
Corneal stromal keratocytes	Plekhn1	-0.007626294	0.027810192	0.0002247	0.000414803
Corneal stromal keratocytes	Taok3	-0.007590779	0.096850182	1.47E-07	3.85E-07
Corneal stromal keratocytes	Cep44	-0.007589959	0.036206614	0.0001812	0.000337575
Corneal stromal keratocytes	Arhgdig	-0.007580473	0.012151922	0.0001426	0.000268982

Corneal stromal keratocytes	Ankrd44	-0.007553887	0.043016281	2.88E-09	8.78E-09
Corneal stromal keratocytes	Chn2	-0.007543694	0.014574293	0.0001115	0.000212766
Corneal stromal keratocytes	Cmtm8	-0.007489194	0.0074352	2.96E-07	7.47E-07
Corneal stromal keratocytes	Rhd	-0.007482219	0.018109002	3.21E-06	7.24E-06
Corneal stromal keratocytes	Polr1b	-0.007464544	0.040135518	0.0094864	0.014204375
Corneal stromal keratocytes	Zer1	-0.007439217	0.024004095	1.42E-05	2.99E-05
Corneal stromal keratocytes	1110059G10Rik	-0.007362859	0.076637726	0.0020365	0.003343949
Corneal stromal keratocytes	Tox2	-0.007280036	0.020069029	0.000232	0.000427564
Corneal stromal keratocytes	Mturn	-0.007265434	0.030046921	0.0002015	0.000373994
Corneal stromal keratocytes	Pik3r1	-0.007262786	0.343312764	7.41E-12	2.80E-11
Corneal stromal keratocytes	Inppl1	-0.007231395	0.046187991	4.18E-05	8.39E-05
Corneal stromal keratocytes	Ttc23	-0.00722672	0.026217792	0.0002233	0.00041235
Corneal stromal keratocytes	Sptan1	-0.007169098	0.440728657	1.62E-19	1.09E-18
Corneal stromal keratocytes	Egr1	-0.007142673	0.69240103	3.25E-10	1.08E-09
Corneal stromal keratocytes	Zfp691	-0.007138838	0.026264739	2.35E-05	4.86E-05
Corneal stromal keratocytes	Sgsm3	-0.007137281	0.076431566	9.66E-06	2.07E-05
Corneal stromal keratocytes	Bcl2	-0.00710045	0.031444657	0.0333851	0.046022532
Corneal stromal keratocytes	Hebp2	-0.007073197	0.023573097	7.18E-06	1.57E-05
Corneal stromal keratocytes	Srd5a1	-0.007045889	0.010617717	5.43E-05	0.000107551
Corneal stromal keratocytes	Slc25a38	-0.007036686	0.055799135	4.28E-05	8.59E-05
Corneal stromal keratocytes	Pde5a	-0.007031441	0.01424965	0.0002309	0.000425551
Corneal stromal keratocytes	Pear1	-0.006934381	0.016974665	0.0002046	0.000379499
Corneal stromal keratocytes	Usp11	-0.006885821	0.026778811	0.0013844	0.0023171
Corneal stromal keratocytes	Slc6a8	-0.006876128	0.335055639	1.21E-10	4.18E-10
Corneal stromal keratocytes	Smad1	-0.006868242	0.232131199	1.33E-06	3.14E-06
Corneal stromal keratocytes	Vac14	-0.006800749	0.026427643	9.34E-05	0.000179719
Corneal stromal keratocytes	Dcaf4	-0.006609794	0.015692443	0.0076258	0.011546383
Corneal stromal keratocytes	Setdb1	-0.006594597	0.061118498	0.0084436	0.012710645
Corneal stromal keratocytes	Las1l	-0.006533569	0.11595264	0.0002824	0.000515084
Corneal stromal keratocytes	4921504A21Rik	-0.006487812	0.010224364	0.0176518	0.025424587
Corneal stromal keratocytes	Igf2bp3	-0.00648495	0.009169243	5.99E-05	0.000117942
Corneal stromal keratocytes	Tpm1	-0.006460492	0.512460342	6.01E-08	1.63E-07
Corneal stromal keratocytes	Pbx2	-0.006378124	0.038659491	7.48E-06	1.63E-05
Corneal stromal keratocytes	Ankrd29	-0.006333274	0.021614915	0.013752	0.020146504
Corneal stromal keratocytes	Magi2	-0.006239835	0.041034043	0.0009355	0.001599335
Corneal stromal keratocytes	Ints10	-0.00620537	0.035877766	0.0003422	0.000617974
Corneal stromal keratocytes	Fbxl8	-0.006167889	0.009293734	0.0005985	0.001049581
Corneal stromal keratocytes	Spef1	-0.006164326	0.036742774	0.0049651	0.007723555
Corneal stromal keratocytes	Efs	-0.006157763	0.035294867	0.0021672	0.003548071
Corneal stromal keratocytes	Sbsn	-0.006124888	0.043017711	0.005927	0.009117827
Corneal stromal keratocytes	Sin3a	-0.006086588	0.037668858	2.24E-07	5.75E-07
Corneal stromal keratocytes	Tmem140	-0.006076169	0.046953562	0.0001487	0.000279649
Corneal stromal keratocytes	Trim12c	-0.005969234	0.023664591	0.0009199	0.001573819
Corneal stromal keratocytes	Adam8	-0.005902749	0.012707669	0.0157239	0.022799774
Corneal stromal keratocytes	Trpt1	-0.005887822	0.014742139	0.000632	0.001103667
Corneal stromal keratocytes	Flt3l	-0.005881297	0.022227876	0.0192884	0.02760352
Corneal stromal keratocytes	Gpsm1	-0.00586378	0.035569406	0.0004053	0.00072507
Corneal stromal keratocytes	Alkbh2	-0.005856977	0.039126934	0.0021989	0.003597063
Corneal stromal keratocytes	Fmo5	-0.005843223	0.010680697	0.0023662	0.003859542
Corneal stromal keratocytes	Acsf2	-0.005821755	0.021041582	0.0023265	0.00379971
Corneal stromal keratocytes	Fastkd2	-0.005769682	0.029842159	0.0053387	0.008271788
Corneal stromal keratocytes	Ppp1r9a	-0.005747983	0.033496611	3.39E-05	6.86E-05
Corneal stromal keratocytes	Kcns3	-0.005741025	0.008165123	5.31E-08	1.44E-07
Corneal stromal keratocytes	Shisa2	-0.00572083	0.014067372	3.42E-08	9.44E-08
Corneal stromal keratocytes	Fbxo25	-0.005710796	0.018880254	0.0172021	0.024812508
Corneal stromal keratocytes	Galm	-0.005710378	0.009064575	0.0145112	0.021183553
Corneal stromal keratocytes	Aldh3b1	-0.005707193	0.016823348	0.0100608	0.01499881
Corneal stromal keratocytes	Fam220a	-0.005695768	0.013726237	5.40E-05	0.000107123
Corneal stromal keratocytes	Ppp1r3g	-0.005668514	0.007514221	0.0190089	0.027236824
Corneal stromal keratocytes	Smarcd2	-0.005651291	0.044358247	0.0042357	0.006659801
Corneal stromal keratocytes	Wdr34	-0.00564756	0.027734639	0.0045587	0.007127898
Corneal stromal keratocytes	Ecm1	-0.005637999	0.13876605	1.30E-06	3.06E-06
Corneal stromal keratocytes	Pcgf6	-0.005608113	0.040553061	0.0163852	0.02370723

Corneal stromal keratocytes	Kat8	-0.005605655	0.048285107	0.02401	0.033826092
Corneal stromal keratocytes	Odf2	-0.005592417	0.057473818	0.005741	0.008845215
Corneal stromal keratocytes	Lonrf3	-0.005587784	0.052037804	0.0043124	0.006768291
Corneal stromal keratocytes	Tmem179b	-0.005577069	0.010001778	1.95E-05	4.07E-05
Corneal stromal keratocytes	Cenps	-0.005550203	0.007080894	0.0125624	0.018492552
Corneal stromal keratocytes	Gbp7	-0.005547531	0.321976822	3.55E-07	8.89E-07
Corneal stromal keratocytes	Alkbh8	-0.005428538	0.066857248	0.0130051	0.019116108
Corneal stromal keratocytes	Man1a2	-0.005413691	0.213431382	1.85E-10	6.26E-10
Corneal stromal keratocytes	Cnksr3	-0.005403475	0.034650907	0.0003439	0.000620872
Corneal stromal keratocytes	Mad2l1bp	-0.005301491	0.025721099	0.0006525	0.00113776
Corneal stromal keratocytes	Vars2	-0.00521828	0.011990319	0.0355799	0.048832424
Corneal stromal keratocytes	Inpp4b	-0.005138744	0.027668057	0.0005446	0.000959999
Corneal stromal keratocytes	Sod2	-0.005125567	0.8497263	8.23E-29	1.01E-27
Corneal stromal keratocytes	4930523C07Rik	-0.005123305	0.041275304	0.0169312	0.024449407
Corneal stromal keratocytes	Rab3il1	-0.005087123	0.026286987	0.0023799	0.003879682
Corneal stromal keratocytes	Gbp6	-0.005084205	0.07385291	0.0237013	0.033461597
Corneal stromal keratocytes	Stox2	-0.005066936	0.038582048	7.65E-08	2.05E-07
Corneal stromal keratocytes	Tnnt1	-0.005066882	0.053272391	2.56E-05	5.27E-05
Corneal stromal keratocytes	Mink1	-0.005063528	0.060012449	0.0002543	0.00046615
Corneal stromal keratocytes	Notch1	-0.005051013	0.049953795	0.0001895	0.000352603
Corneal stromal keratocytes	Dgkz	-0.005032837	0.033161213	0.000329	0.000595457
Corneal stromal keratocytes	Kmt5c	-0.005010726	0.0107361	0.0015473	0.002574965
Corneal stromal keratocytes	Lbp	-0.004951503	0.031596464	1.04E-05	2.23E-05
Corneal stromal keratocytes	Gprasp2	-0.004931768	0.01329107	5.65E-05	0.000111744
Corneal stromal keratocytes	Strada	-0.004904011	0.022765267	0.0008117	0.001398353
Corneal stromal keratocytes	Gm11627	-0.004868659	0.012505457	0.0003912	0.000701382
Corneal stromal keratocytes	Ache	-0.004864459	0.006524449	0.0001401	0.000264344
Corneal stromal keratocytes	Entpd2	-0.004825796	0.057316517	2.43E-07	6.19E-07
Corneal stromal keratocytes	Zdhhc1	-0.004815581	0.041501953	0.0001713	0.000320108
Corneal stromal keratocytes	Rps6kc1	-0.004789211	0.033625014	0.0028946	0.004655969
Corneal stromal keratocytes	Taf8	-0.004789063	0.03066505	0.0002666	0.000487519
Corneal stromal keratocytes	Cln6	-0.004779517	0.022814081	0.0030004	0.004813387
Corneal stromal keratocytes	Arhgef5	-0.004772293	0.036448813	6.88E-08	1.85E-07
Corneal stromal keratocytes	Ldb2	-0.004756618	0.014634336	0.006509	0.009949852
Corneal stromal keratocytes	Zfc3h1	-0.004745069	0.088240115	1.66E-06	3.87E-06
Corneal stromal keratocytes	Ippk	-0.00468986	0.114698199	8.23E-06	1.78E-05
Corneal stromal keratocytes	Tsen54	-0.004681991	0.024473745	0.0001093	0.000208837
Corneal stromal keratocytes	Smc6	-0.004668039	0.283563657	1.01E-08	2.96E-08
Corneal stromal keratocytes	Mpv17l	-0.004667316	0.021452658	0.0043806	0.006869154
Corneal stromal keratocytes	Abcc1	-0.004659532	0.088945502	2.37E-05	4.89E-05
Corneal stromal keratocytes	Asb13	-0.00459782	0.015537821	0.0158324	0.022940536
Corneal stromal keratocytes	Fbln7	-0.004596181	0.03866464	0.0001331	0.00025178
Corneal stromal keratocytes	Morc3	-0.004577537	0.061746365	0.0034801	0.005526208
Corneal stromal keratocytes	Klhl8	-0.004576437	0.014533098	0.0027361	0.004419803
Corneal stromal keratocytes	Rgs11	-0.004567287	0.0131326	0.0001951	0.000362679
Corneal stromal keratocytes	Gucy1a3	-0.004548433	0.013417479	2.84E-09	8.69E-09
Corneal stromal keratocytes	Nyap2	-0.004540849	0.007184182	0.0004537	0.0008071
Corneal stromal keratocytes	Car13	-0.004538189	0.015099546	0.0098364	0.014689186
Corneal stromal keratocytes	Mettl22	-0.004527853	0.025305334	0.001954	0.003215026
Corneal stromal keratocytes	Adra2a	-0.004485757	0.042859482	0.0009236	0.001579514
Corneal stromal keratocytes	D330041H03Rik	-0.004447604	0.008963962	0.0258628	0.036294288
Corneal stromal keratocytes	Zfp101	-0.004427592	0.011664061	0.0025937	0.004202322
Corneal stromal keratocytes	Vezt	-0.004391975	0.053957043	0.0016469	0.002730771
Corneal stromal keratocytes	Lpar6	-0.004385188	0.02425615	0.0176602	0.025434067
Corneal stromal keratocytes	H1fx	-0.004330472	0.024016731	0.0318217	0.043966632
Corneal stromal keratocytes	Samhd1	-0.004329186	0.083177998	1.79E-06	4.17E-06
Corneal stromal keratocytes	Lgalsl	-0.004325979	0.146999612	5.32E-07	1.31E-06
Corneal stromal keratocytes	Mettl18	-0.004254388	0.011329007	0.0054677	0.00845104
Corneal stromal keratocytes	Vegfd	-0.004251737	0.014062852	0.0058046	0.008938391
Corneal stromal keratocytes	Sh3bp1	-0.004249944	0.009084192	0.0136191	0.019962253
Corneal stromal keratocytes	Cntrl	-0.00423611	0.014970651	0.0041604	0.006547167
Corneal stromal keratocytes	Gatb	-0.004216428	0.010101022	0.0050396	0.007833436
Corneal stromal keratocytes	Phf13	-0.004211597	0.037611632	0.0028171	0.004540844

Corneal stromal keratocytes	Lalba	-0.004174968	0.028482381	0.0044304	0.006941803
Corneal stromal keratocytes	Cep112	-0.004133454	0.050359522	0.0004186	0.000747932
Corneal stromal keratocytes	Dusp16	-0.004116424	0.042449339	8.46E-06	1.83E-05
Corneal stromal keratocytes	Ankrd27	-0.004107974	0.023676125	0.0208298	0.029661129
Corneal stromal keratocytes	Dennd1a	-0.004087957	0.074781514	0.0004427	0.000788971
Corneal stromal keratocytes	Ccdc163	-0.004030551	0.01156596	0.0142822	0.020866685
Corneal stromal keratocytes	Serp2	-0.003971457	0.005935697	0.0079365	0.011981953
Corneal stromal keratocytes	Prkcg	-0.003961372	0.018340232	0.0070378	0.010715131
Corneal stromal keratocytes	Scnn1b	-0.003949757	0.006703624	0.0179314	0.025782321
Corneal stromal keratocytes	Pisd	-0.003940994	0.030568498	0.0004489	0.000799179
Corneal stromal keratocytes	Anks1	-0.003926872	0.075517155	0.0095256	0.014255325
Corneal stromal keratocytes	Mbd5	-0.003917096	0.037637739	0.0064731	0.009899331
Corneal stromal keratocytes	Fam109a	-0.003904952	0.029686337	0.0044452	0.006962685
Corneal stromal keratocytes	Pik3cd	-0.003903429	0.007186821	4.42E-05	8.86E-05
Corneal stromal keratocytes	Sphk2	-0.003897898	0.032808658	0.0336222	0.046326587
Corneal stromal keratocytes	Ptpdc1	-0.003895411	0.03590555	2.36E-05	4.87E-05
Corneal stromal keratocytes	Ist1	-0.003894139	0.249986741	1.66E-14	7.73E-14
Corneal stromal keratocytes	Rtl5	-0.003873137	0.02152103	0.0131588	0.019319778
Corneal stromal keratocytes	Hspa12a	-0.003822635	0.018410421	1.06E-05	2.26E-05
Corneal stromal keratocytes	Gm6225	-0.003796058	0.011025264	0.0148295	0.021607808
Corneal stromal keratocytes	Srgap2	-0.003791329	0.076288821	7.89E-06	1.71E-05
Corneal stromal keratocytes	Cdh2	-0.003755095	0.012951941	0.0007594	0.001313673
Corneal stromal keratocytes	Bckdha	-0.003753391	0.056691176	0.0001556	0.000292013
Corneal stromal keratocytes	C2cd4b	-0.003735336	0.011278395	0.0020545	0.003371968
Corneal stromal keratocytes	Lyst	-0.003709858	0.03345819	0.0046918	0.007322716
Corneal stromal keratocytes	Isoc2a	-0.003697421	0.02130992	0.0246407	0.034669371
Corneal stromal keratocytes	Krt17	-0.003692331	0.006428835	6.32E-05	0.000124095
Corneal stromal keratocytes	Ormdl1	-0.003688301	0.012725778	0.0127461	0.018755147
Corneal stromal keratocytes	Rcor3	-0.003681945	0.0396293	0.005476	0.008462985
Corneal stromal keratocytes	Ascc2	-0.00365037	0.047034518	0.004664	0.007282558
Corneal stromal keratocytes	Susd2	-0.003622946	0.010965652	0.0004649	0.000825746
Corneal stromal keratocytes	Scara3	-0.003617727	0.065791653	2.09E-06	4.83E-06
Corneal stromal keratocytes	Smc5	-0.003615778	0.091832349	6.91E-06	1.51E-05
Corneal stromal keratocytes	Ifrd1	-0.003559887	2.221429383	3.46E-06	7.79E-06
Corneal stromal keratocytes	Scmh1	-0.003510382	0.088542764	0.0071317	0.010847422
Corneal stromal keratocytes	Rpusd2	-0.003465353	0.010600829	0.0139082	0.020349881
Corneal stromal keratocytes	Stip1	-0.003413709	0.333090518	2.83E-14	1.30E-13
Corneal stromal keratocytes	Hps6	-0.003412639	0.013067941	0.030043	0.041673183
Corneal stromal keratocytes	Unc93b1	-0.003407503	0.039322511	6.54E-05	0.000128161
Corneal stromal keratocytes	Agbl3	-0.0033684	0.033278417	0.0034032	0.005412112
Corneal stromal keratocytes	Tpcn2	-0.003368189	0.026973018	0.0005879	0.001032033
Corneal stromal keratocytes	Kansl1l	-0.003347155	0.058162782	0.0002945	0.000536379
Corneal stromal keratocytes	Atl1	-0.003343685	0.021944805	0.0043898	0.006882766
Corneal stromal keratocytes	Ppargc1a	-0.003338737	0.011182868	0.004849	0.007555578
Corneal stromal keratocytes	Zfp971	-0.003338543	0.008742414	0.0020567	0.003375072
Corneal stromal keratocytes	Lrfn1	-0.003336997	0.006228256	0.0024576	0.003995693
Corneal stromal keratocytes	Nr1h3	-0.003318671	0.018004391	0.0021531	0.00352592
Corneal stromal keratocytes	Cldn7	-0.003289745	0.019858445	5.89E-08	1.60E-07
Corneal stromal keratocytes	Snx10	-0.003273331	0.026060142	0.0030327	0.004860241
Corneal stromal keratocytes	Nrg1	-0.003236261	0.03300379	0.0152174	0.02213396
Corneal stromal keratocytes	Immp2l	-0.003205807	0.008480315	0.017283	0.024924111
Corneal stromal keratocytes	Hdac10	-0.003179511	0.011952803	0.0003723	0.000669106
Corneal stromal keratocytes	Tnfrsf10b	-0.003175376	0.05402896	0.0351959	0.048347929
Corneal stromal keratocytes	Mcm3ap	-0.003155586	0.036138379	0.0002813	0.000513173
Corneal stromal keratocytes	Tfap4	-0.003155234	0.01466486	0.0157008	0.02277097
Corneal stromal keratocytes	Urah	-0.003133761	0.014675266	0.0005944	0.001042632
Corneal stromal keratocytes	Epha4	-0.00309121	0.068362583	9.39E-06	2.02E-05
Corneal stromal keratocytes	Kif26a	-0.003089715	0.014267375	0.0007734	0.001336218
Corneal stromal keratocytes	Zfp780b	-0.003068611	0.049999436	0.0227812	0.032224155
Corneal stromal keratocytes	2810474O19Rik	-0.003053964	0.049480434	0.0003923	0.000702806
Corneal stromal keratocytes	Zbtb38	-0.003049523	0.16200476	1.47E-07	3.85E-07
Corneal stromal keratocytes	Nmnat3	-0.003044684	0.011803788	0.0238429	0.033614163
Corneal stromal keratocytes	Zpbp	-0.002989108	0.006708567	0.0032115	0.005126339

Corneal stromal keratocytes	Kif5a	-0.002981449	0.007502725	0.0105979	0.015756065
Corneal stromal keratocytes	Pbx3	-0.002919195	0.04292853	0.0007751	0.00133863
Corneal stromal keratocytes	Unc79	-0.002911832	0.009959408	0.0146035	0.021307211
Corneal stromal keratocytes	C330013E15Rik	-0.002900421	0.010297203	0.0009009	0.001543027
Corneal stromal keratocytes	Wdr63	-0.002895833	0.009556641	0.0127582	0.01877101
Corneal stromal keratocytes	Fmo2	-0.002890163	0.041537711	0.000362	0.0006521
Corneal stromal keratocytes	Rarb	-0.002883648	0.023427662	0.0001263	0.00023952
Corneal stromal keratocytes	Ankhd1	-0.002847269	0.140689001	2.04E-05	4.25E-05
Corneal stromal keratocytes	Krt6a	-0.002826465	0.012350849	6.49E-06	1.42E-05
Corneal stromal keratocytes	Slc9a5	-0.002822359	0.027015379	0.0001421	0.000267997
Corneal stromal keratocytes	Dynlt1a	-0.00279794	0.009909169	0.0007629	0.001319512
Corneal stromal keratocytes	Egfr	-0.002776646	0.083388038	4.11E-06	9.19E-06
Corneal stromal keratocytes	Tmem186	-0.002776161	0.031052029	0.0120214	0.017742756
Corneal stromal keratocytes	Chd3os	-0.002760919	0.010273471	0.0117566	0.017370165
Corneal stromal keratocytes	Iqck	-0.002713225	0.00707835	0.0140878	0.020601914
Corneal stromal keratocytes	Abcc5	-0.002703128	0.078937748	1.59E-06	3.73E-06
Corneal stromal keratocytes	Tldc1	-0.002700559	0.016125696	0.0141179	0.020639522
Corneal stromal keratocytes	Sfrp2	-0.002687617	0.071281152	0.0180212	0.025898205
Corneal stromal keratocytes	Hexim1	-0.002648052	0.152589597	0.0015157	0.002527253
Corneal stromal keratocytes	Atf7ip	-0.002647111	0.026226852	0.016739	0.024181813
Corneal stromal keratocytes	Ppp1r12a	-0.002608675	0.22664166	2.72E-13	1.15E-12
Corneal stromal keratocytes	Nipal3	-0.0025824	0.010738687	0.0186787	0.026785457
Corneal stromal keratocytes	Rragd	-0.002559544	0.073789611	3.84E-05	7.74E-05
Corneal stromal keratocytes	Aatk	-0.002535851	0.024023618	0.0007197	0.00124756
Corneal stromal keratocytes	Samd9l	-0.002488776	0.079330369	3.23E-06	7.29E-06
Corneal stromal keratocytes	Ggnbp2	-0.002474367	0.353790554	5.49E-17	3.10E-16
Corneal stromal keratocytes	Trim11	-0.002467269	0.052045059	0.0002266	0.000418054
Corneal stromal keratocytes	Tnip1	-0.002445326	0.034004811	0.0010406	0.001768923
Corneal stromal keratocytes	Flt4	-0.00243978	0.009127128	0.0331686	0.04574652
Corneal stromal keratocytes	Il18	-0.002437801	0.007914298	0.0083551	0.012588214
Corneal stromal keratocytes	Arrdc1	-0.002430275	0.010427959	0.0031679	0.005061026
Corneal stromal keratocytes	Hmgxb4	-0.002428717	0.054536277	1.22E-06	2.88E-06
Corneal stromal keratocytes	Kifc3	-0.002425407	0.02783861	0.00077	0.00133045
Corneal stromal keratocytes	Ccdc61	-0.002415973	0.00734442	0.0076148	0.011532338
Corneal stromal keratocytes	Sgk3	-0.002398998	0.020370562	0.0088841	0.013342272
Corneal stromal keratocytes	Cdca7	-0.00239066	0.016346269	0.0086184	0.012960742
Corneal stromal keratocytes	Trim21	-0.002354676	0.033530223	0.0070809	0.010777155
Corneal stromal keratocytes	Cdh4	-0.002336287	0.010366532	5.02E-05	9.99E-05
Corneal stromal keratocytes	Ephb6	-0.002327782	0.022080795	0.0168624	0.02435255
Corneal stromal keratocytes	Fmn2	-0.002182651	0.005020021	0.0063566	0.009733954
Corneal stromal keratocytes	Anxa3	-0.002155143	0.065347639	0.0203203	0.028979691
Corneal stromal keratocytes	Map3k12	-0.002154625	0.015276701	0.0060189	0.00924607
Corneal stromal keratocytes	Usp45	-0.002153887	0.039676153	0.005489	0.008482123
Corneal stromal keratocytes	Iqcb1	-0.002132609	0.050835068	0.0032629	0.00520315
Corneal stromal keratocytes	Ago4	-0.002126306	0.014852635	0.007079	0.010775509
Corneal stromal keratocytes	C4b	-0.002096801	0.01867384	0.0006079	0.00106447
Corneal stromal keratocytes	Glrb	-0.002092933	0.119155062	0.0325736	0.044961287
Corneal stromal keratocytes	Plxna3	-0.002061078	0.019520634	0.0088143	0.013241702
Corneal stromal keratocytes	Exog	-0.002034241	0.018546689	0.006314	0.009670763
Corneal stromal keratocytes	Capn1	-0.002015426	0.070962039	2.22E-06	5.11E-06
Corneal stromal keratocytes	Notch4	-0.001980425	0.006128081	0.0056872	0.008769104
Corneal stromal keratocytes	Rarres1	-0.00196392	0.010356372	0.0014052	0.002349634
Corneal stromal keratocytes	Lgals7	-0.001901702	0.019111532	0.0030341	0.004861999
Corneal stromal keratocytes	Yeats2	-0.001874115	0.045875721	0.0236362	0.033376427
Corneal stromal keratocytes	Zfyve26	-0.001867767	0.024265374	0.0042493	0.006677433
Corneal stromal keratocytes	Tnfrsf11b	-0.001836458	0.066720404	0.0058204	0.008961611
Corneal stromal keratocytes	Kif13a	-0.001817374	0.057241036	4.66E-05	9.31E-05
Corneal stromal keratocytes	Vopp1	-0.001809443	0.047642421	0.0114732	0.016979982
Corneal stromal keratocytes	Gdf11	-0.001806203	0.057238119	0.0024067	0.003919299
Corneal stromal keratocytes	Cat	-0.001798951	0.059784696	0.0008736	0.001499672
Corneal stromal keratocytes	Dysf	-0.001793569	0.010227929	0.0305244	0.042286582
Corneal stromal keratocytes	Tada2a	-0.001785193	0.031108952	0.0002476	0.000454549
Corneal stromal keratocytes	Bcl10	-0.00178443	0.481903063	1.71E-13	7.38E-13

Corneal stromal keratocytes	Wdr19	-0.001768505	0.006444754	0.0301867	0.041864161
Corneal stromal keratocytes	Anxa1	-0.001742091	0.87658959	3.96E-18	2.43E-17
Corneal stromal keratocytes	Poli	-0.001734588	0.014388082	0.0014374	0.002400657
Corneal stromal keratocytes	B230216N24Rik	-0.001674723	0.018479107	0.0115588	0.017095859
Corneal stromal keratocytes	Prdm6	-0.00165881	0.007373883	0.0001317	0.000249311
Corneal stromal keratocytes	Gimap6	-0.001649967	0.01877061	0.0235068	0.033200466
Corneal stromal keratocytes	Amer1	-0.001632721	0.019402485	0.0093686	0.01403537
Corneal stromal keratocytes	Meis1	-0.001625315	0.00652523	0.0037637	0.005950969
Corneal stromal keratocytes	Atp6v0d1	-0.001599761	0.322969814	4.44E-09	1.34E-08
Corneal stromal keratocytes	Tma16	-0.001592597	0.186678758	5.13E-10	1.67E-09
Corneal stromal keratocytes	Dusp10	-0.001584419	0.310308483	9.77E-08	2.60E-07
Corneal stromal keratocytes	Fam20a	-0.001565345	0.0169919	0.0009508	0.001623836
Corneal stromal keratocytes	Pfas	-0.001560951	0.022151163	0.0084332	0.012697722
Corneal stromal keratocytes	Aste1	-0.001545157	0.03127646	3.46E-05	7.02E-05
Corneal stromal keratocytes	Slc43a1	-0.00154339	0.01801354	0.0009947	0.001695165
Corneal stromal keratocytes	1110004F10Rik	-0.001491288	0.461604523	5.42E-15	2.62E-14
Corneal stromal keratocytes	Usp18	-0.001483045	0.028243773	1.35E-05	2.86E-05
Corneal stromal keratocytes	Ankrd13a	-0.001478911	0.102297559	0.0001695	0.000316887
Corneal stromal keratocytes	Zfp958	-0.001472029	0.032108591	0.0023751	0.003872741
Corneal stromal keratocytes	1110038F14Rik	-0.001470662	0.169090051	4.30E-05	8.62E-05
Corneal stromal keratocytes	S100a14	-0.001465064	0.012389667	0.0186009	0.026682065
Corneal stromal keratocytes	Nit1	-0.001455205	0.047001098	0.0008159	0.001404856
Corneal stromal keratocytes	Rtel1	-0.001439676	0.011894473	0.0081457	0.012284584
Corneal stromal keratocytes	Plxnd1	-0.001437747	0.024200284	0.0098661	0.014730427
Corneal stromal keratocytes	Carhsp1	-0.001388935	0.128473133	1.46E-06	3.43E-06
Corneal stromal keratocytes	Lpl	-0.001315001	0.028229753	0.0054286	0.008398011
Corneal stromal keratocytes	Timmdc1	-0.00129552	0.052428784	0.0039757	0.006267736
Corneal stromal keratocytes	Hs3st3b1	-0.001293357	0.02489491	0.0180697	0.025959845
Corneal stromal keratocytes	Fam192a	-0.001282155	0.100873386	0.0022264	0.003640842
Corneal stromal keratocytes	Palmd	-0.001270198	0.018774931	0.0003298	0.000596822
Corneal stromal keratocytes	F11r	-0.001256659	0.008734685	0.0060871	0.009348783
Corneal stromal keratocytes	Malt1	-0.00121921	0.013578795	0.0019945	0.003277261
Corneal stromal keratocytes	Vps45	-0.00121103	0.033548946	0.0049685	0.007727922
Corneal stromal keratocytes	Slurp1	-0.001176625	0.029180887	1.57E-11	5.76E-11
Corneal stromal keratocytes	Epb41l5	-0.001125988	0.007448519	0.0040605	0.006395038
Corneal stromal keratocytes	Nfkbie	-0.001118351	0.023324599	0.021192	0.030140205
Corneal stromal keratocytes	Exosc9	-0.001117932	0.069866884	0.0020722	0.003399349
Corneal stromal keratocytes	Kif16b	-0.001096842	0.046382527	0.0108042	0.016042346
Corneal stromal keratocytes	Tmem88	-0.001079184	0.01146872	0.0068125	0.010385549
Corneal stromal keratocytes	Tmem135	-0.001062288	0.046179654	0.0005862	0.001029286
Corneal stromal keratocytes	Cobll1	-0.001058177	0.027859228	0.0018929	0.003118704
Corneal stromal keratocytes	Usp47	-0.001027062	0.319622089	1.49E-08	4.27E-08
Corneal stromal keratocytes	Atad5	-0.001015453	0.006633149	0.0022703	0.003710415
Corneal stromal keratocytes	Clstn3	-0.00098777	0.018092117	0.0048863	0.007610205
Corneal stromal keratocytes	Chad	-0.000965003	0.017505484	3.06E-06	6.92E-06
Corneal stromal keratocytes	Asprv1	-0.00092579	0.009551506	0.0229627	0.032461296
Corneal stromal keratocytes	Ctnnal1	-0.000922761	0.033935981	0.000721	0.001249474
Corneal stromal keratocytes	Tbc1d4	-0.000839969	0.024654764	0.0006089	0.001066051
Corneal stromal keratocytes	Tmc6	-0.000770986	0.047016998	0.0155755	0.022610299
Corneal stromal keratocytes	Mafb	-0.000746523	0.081354712	2.93E-05	5.98E-05
Corneal stromal keratocytes	Rpa1	-0.00073566	0.042846283	0.0130834	0.01921916
Corneal stromal keratocytes	Gimap9	-0.000730802	0.012258362	0.003222	0.005142466
Corneal stromal keratocytes	Dpyd	-0.00072467	0.008747463	0.012441	0.018328042
Corneal stromal keratocytes	Ripk2	-0.000704974	0.033940013	0.0044076	0.006908784
Corneal stromal keratocytes	Stk19	-0.000667741	0.319050875	2.14E-07	5.49E-07
Corneal stromal keratocytes	Hus1	-0.000660509	0.029838719	0.0167503	0.024195652
Corneal stromal keratocytes	Mcm7	-0.000576481	0.035123667	0.0023911	0.003896123
Corneal stromal keratocytes	Mapkapk3	-0.000467982	0.110218377	0.0013935	0.002330626
Corneal stromal keratocytes	Ints4	-0.000445244	0.03026293	0.0023406	0.003821396
Corneal stromal keratocytes	P2rx4	-0.000436193	0.064945642	0.0009138	0.001564103
Corneal stromal keratocytes	Snx1	-0.00039071	0.11662179	8.10E-07	1.95E-06
Corneal stromal keratocytes	Zmym2	-0.000347809	0.068934971	0.0001485	0.000279344
Corneal stromal keratocytes	Tom1l1	-0.000335464	0.019397783	0.0029396	0.004723459

Corneal stromal keratocytes	Plekho1	-0.000260353	0.014249164	0.0001631	0.000305561
Corneal stromal keratocytes	Cldn4	-0.000248885	0.010743238	1.03E-07	2.74E-07
Corneal stromal keratocytes	Mre11a	-0.000243635	0.021916456	0.0020471	0.00336018
Corneal stromal keratocytes	Sirt6	-0.000235171	0.018692173	0.0357107	0.04897842
Corneal stromal keratocytes	Mal	-0.000180479	0.010994932	0.0024319	0.003957566
Corneal stromal keratocytes	G0s2	-0.000178898	0.182992652	3.54E-06	7.95E-06
Corneal stromal keratocytes	E2f5	-0.000168348	0.042310391	0.0002132	0.000394957
Corneal stromal keratocytes	Tnnc1	-0.000160904	0.029953967	0.0202137	0.028842313
Corneal stromal keratocytes	Dusp8	-0.000156986	0.007349195	0.0171853	0.024790888
Corneal stromal keratocytes	Ccdc138	-0.00014761	0.015150521	0.0007245	0.001255024
Corneal stromal keratocytes	Serpinb5	-0.000133445	0.012020223	0.0031093	0.004973383
Corneal stromal keratocytes	Frem2	-0.000118849	0.006287629	0.0147861	0.021551306
Corneal stromal keratocytes	Rgs12	-9.93E-05	0.035166348	0.0002961	0.000538916
Corneal stromal keratocytes	Rbp2	-5.59E-05	0.004734299	2.11E-05	4.39E-05
Corneal stromal keratocytes	Slc36a1	-3.30E-05	0.018483523	0.0252289	0.035440115
Corneal stromal keratocytes	Nipsnap3b	-3.02E-05	0.022475404	0.0005005	0.000885978
Corneal stromal keratocytes	Zfp955b	-5.39E-06	0.074230393	0.0009697	0.001654394
Corneal stromal keratocytes	Bnc2	1.25E-05	0.033920028	0.0026114	0.004230001
Corneal stromal keratocytes	Prcd	2.17E-05	0.031789617	0.000966	0.001648208
Corneal stromal keratocytes	Tmem38b	5.62E-05	0.060695337	0.0006159	0.001076778
Corneal stromal keratocytes	Tmem267	7.30E-05	0.100220361	4.81E-10	1.57E-09
Corneal stromal keratocytes	Cds2	8.33E-05	0.073933471	0.0004564	0.000811507
Corneal stromal keratocytes	Zscan29	0.000105109	0.017919408	0.0203852	0.02906646
Corneal stromal keratocytes	Lama4	0.000155177	0.055160838	7.14E-06	1.56E-05
Corneal stromal keratocytes	Maml3	0.000198459	0.080293442	0.0136118	0.019955783
Corneal stromal keratocytes	Ptpn14	0.000247617	0.028843675	0.0041684	0.006559079
Corneal stromal keratocytes	Hs3st3a1	0.000325495	0.013477641	0.0188525	0.027020913
Corneal stromal keratocytes	Cox6a2	0.000328608	0.006308707	0.0169497	0.02447355
Corneal stromal keratocytes	Tmod1	0.000333686	0.012903806	0.0029745	0.004773048
Corneal stromal keratocytes	Rtn1	0.000345104	0.010131951	0.0077952	0.011782537
Corneal stromal keratocytes	Atp1b1	0.000355805	0.05401894	0.0001394	0.000263113
Corneal stromal keratocytes	Prkaa2	0.00037627	0.006271096	0.0356646	0.048919958
Corneal stromal keratocytes	Zfp715	0.000378767	0.044885954	0.009036	0.013560241
Corneal stromal keratocytes	Lypd3	0.000468031	0.007468321	0.0007473	0.001293324
Corneal stromal keratocytes	Ccnd1	0.000478342	0.06805823	6.80E-07	1.65E-06
Corneal stromal keratocytes	Fam76b	0.000483536	0.066891298	0.0337878	0.046541063
Corneal stromal keratocytes	2610528A11Rik	0.000491404	0.023972773	1.29E-05	2.73E-05
Corneal stromal keratocytes	Pik3cb	0.00056155	0.006266226	0.0079941	0.012065966
Corneal stromal keratocytes	Lamb1	0.000570699	0.057988481	4.14E-06	9.23E-06
Corneal stromal keratocytes	Id4	0.000608256	0.035160146	0.0035076	0.005567291
Corneal stromal keratocytes	E2f1	0.000629214	0.014694532	0.007652	0.011581052
Corneal stromal keratocytes	Ppfibp2	0.000652543	0.024070048	0.0239925	0.03380478
Corneal stromal keratocytes	Mmp14	0.000663557	0.405371132	2.32E-08	6.51E-08
Corneal stromal keratocytes	Nudt4	0.000682067	0.365463075	6.85E-13	2.81E-12
Corneal stromal keratocytes	Stom	0.000683411	0.050371954	0.0047837	0.007459503
Corneal stromal keratocytes	Epha5	0.000690182	0.004372683	0.0316279	0.043716077
Corneal stromal keratocytes	Egfl7	0.000724219	0.015908377	0.0015156	0.002527253
Corneal stromal keratocytes	Pml	0.00072497	0.030400492	0.0011929	0.002011904
Corneal stromal keratocytes	Bcam	0.000747162	0.004021883	0.0246708	0.034704748
Corneal stromal keratocytes	Naalad2	0.000771849	0.038020379	0.0015187	0.002531572
Corneal stromal keratocytes	Ralgps2	0.000774777	0.02036775	0.0272735	0.03814511
Corneal stromal keratocytes	Rnf216	0.000776474	0.130552263	1.05E-05	2.25E-05
Corneal stromal keratocytes	Dhx40	0.000779661	0.091546478	1.61E-05	3.39E-05
Corneal stromal keratocytes	Il17d	0.000806909	0.01834918	0.0180145	0.02589124
Corneal stromal keratocytes	Paox	0.000809682	0.019842949	0.0004436	0.000790391
Corneal stromal keratocytes	Rb1cc1	0.00083063	0.405360107	2.51E-10	8.41E-10
Corneal stromal keratocytes	Itgb4	0.000867099	0.006121472	0.0325934	0.044984204
Corneal stromal keratocytes	Phtf2	0.000877412	0.041311622	0.002768	0.00446718
Corneal stromal keratocytes	Dapp1	0.000883153	0.013767168	0.0243752	0.034316433
Corneal stromal keratocytes	Sytl1	0.000894912	0.004849554	0.0348287	0.047871539
Corneal stromal keratocytes	Zdhhc7	0.000935591	0.031661972	0.0108259	0.016067798
Corneal stromal keratocytes	Slc24a3	0.001015723	0.007436145	0.0187445	0.026874329
Corneal stromal keratocytes	Tmem229b	0.00103196	0.013554446	0.0138634	0.020290687

Corneal stromal keratocytes	Fes	0.001089107	0.029180482	0.0046451	0.007253889
Corneal stromal keratocytes	Pawr	0.001100565	0.018399856	0.0105254	0.015656499
Corneal stromal keratocytes	Kank2	0.001174546	0.058288163	0.0001025	0.000196495
Corneal stromal keratocytes	Lbh	0.001176619	0.101651044	2.75E-07	6.96E-07
Corneal stromal keratocytes	Tsc22d3	0.001203127	0.509897237	7.09E-15	3.40E-14
Corneal stromal keratocytes	Phactr2	0.00120548	0.086752211	2.56E-05	5.27E-05
Corneal stromal keratocytes	Plekhb1	0.001464631	0.037556794	0.0028649	0.00461178
Corneal stromal keratocytes	Peg13	0.00159286	0.021605852	0.026766	0.037479398
Corneal stromal keratocytes	St3gal4	0.001683955	0.014199102	0.0103719	0.015447883
Corneal stromal keratocytes	Thada	0.001747763	0.019231045	0.0120778	0.017822126
Corneal stromal keratocytes	Prcc	0.001773674	0.062665008	0.0013832	0.002315328
Corneal stromal keratocytes	Mustn1	0.001835899	0.020548462	0.0302862	0.041985581
Corneal stromal keratocytes	Pinx1	0.001859513	0.099771996	7.61E-06	1.66E-05
Corneal stromal keratocytes	Klf9	0.001882966	1.750772833	5.19E-07	1.28E-06
Corneal stromal keratocytes	Dmd	0.001898451	0.045631083	0.0021426	0.003509935
Corneal stromal keratocytes	Ptger4	0.001923029	0.011460799	0.0095694	0.014319397
Corneal stromal keratocytes	Lrfn4	0.001926288	0.025722049	0.0115491	0.017083296
Corneal stromal keratocytes	Ggta1	0.001959324	0.008403638	0.0238404	0.033614026
Corneal stromal keratocytes	C2	0.001972843	0.014510633	0.0329066	0.045411895
Corneal stromal keratocytes	Fam135a	0.001979077	0.038430374	0.0091559	0.013731367
Corneal stromal keratocytes	Ttll4	0.002046446	0.035680835	0.0005137	0.000908494
Corneal stromal keratocytes	Chml	0.002108903	0.038286848	1.40E-05	2.97E-05
Corneal stromal keratocytes	44262	0.002109013	0.196077341	6.80E-10	2.19E-09
Corneal stromal keratocytes	Rbpms	0.002125582	0.130333382	3.04E-05	6.21E-05
Corneal stromal keratocytes	Lrig2	0.002132967	0.032611733	0.003459	0.005495792
Corneal stromal keratocytes	Ifit1	0.002143433	0.140163572	5.63E-06	1.24E-05
Corneal stromal keratocytes	Il10rb	0.002178412	0.02434161	0.0005221	0.000922845
Corneal stromal keratocytes	Angptl7	0.002183792	2.656276661	0.0098469	0.014703357
Corneal stromal keratocytes	Enc1	0.002196323	0.097079423	0.0018691	0.003081956
Corneal stromal keratocytes	Cox6b2	0.002196681	0.019128892	0.0049416	0.007688691
Corneal stromal keratocytes	Armc9	0.002250692	0.065386661	0.0023898	0.00389487
Corneal stromal keratocytes	Vcan	0.002327308	0.022294289	0.0003968	0.00071061
Corneal stromal keratocytes	Evpl	0.002366532	0.007217298	0.0251712	0.035366187
Corneal stromal keratocytes	Lmo2	0.002419346	0.014699421	0.0020436	0.003354705
Corneal stromal keratocytes	Gsta2	0.002425368	0.005267722	0.0005289	0.000934403
Corneal stromal keratocytes	Fbxo33	0.002436184	0.086432549	7.14E-05	0.000139329
Corneal stromal keratocytes	Gxylt1	0.002519403	0.061492488	0.0003276	0.000593035
Corneal stromal keratocytes	Cbr2	0.002527674	0.007761245	8.89E-05	0.000171545
Corneal stromal keratocytes	Atp1a2	0.002564889	0.017719721	0.0129218	0.019001667
Corneal stromal keratocytes	Larp1b	0.002581004	0.206094258	5.26E-05	0.000104531
Corneal stromal keratocytes	Camta1	0.002591062	0.261001443	2.68E-06	6.11E-06
Corneal stromal keratocytes	Hspb8	0.002612112	0.023108025	0.0014967	0.002497257
Corneal stromal keratocytes	Styx	0.002682912	0.020511509	0.007104	0.010809992
Corneal stromal keratocytes	Adora2b	0.002691672	0.019667501	0.0042492	0.006677433
Corneal stromal keratocytes	Nrk	0.00269873	0.009632127	0.0033548	0.005338275
Corneal stromal keratocytes	Trim29	0.002727755	0.006193359	0.021182	0.030129115
Corneal stromal keratocytes	Tfip11	0.002764839	0.083634887	0.0010231	0.001740528
Corneal stromal keratocytes	Map2k3os	0.002775522	0.024062334	0.0275386	0.038495925
Corneal stromal keratocytes	Epcam	0.002852607	0.206227683	0.0058011	0.008933917
Corneal stromal keratocytes	Jarid2	0.00285433	0.229768314	3.09E-09	9.40E-09
Corneal stromal keratocytes	Eogt	0.002858488	0.014696765	0.0190521	0.02729311
Corneal stromal keratocytes	Chchd10	0.002860823	0.096638609	0.0237555	0.033506591
Corneal stromal keratocytes	Usp25	0.002866326	0.060115447	0.0107257	0.015937637
Corneal stromal keratocytes	Echdc1	0.002924933	0.048069487	0.0073309	0.011134734
Corneal stromal keratocytes	Slc25a48	0.002950305	0.004132006	0.008142	0.01228039
Corneal stromal keratocytes	Asnsd1	0.00300871	0.167391777	2.29E-06	5.27E-06
Corneal stromal keratocytes	Zc3h4	0.003014205	0.03620635	0.0008781	0.00150692
Corneal stromal keratocytes	Hspd1	0.003103432	0.916816832	2.30E-28	2.73E-27
Corneal stromal keratocytes	Rfwd3	0.003103524	0.029155455	0.0187866	0.026929193
Corneal stromal keratocytes	Pkdcc	0.00310563	0.03414378	0.0212129	0.030163926
Corneal stromal keratocytes	Rrad	0.003140464	0.014221538	0.0003208	0.000581712
Corneal stromal keratocytes	Cebpa	0.003205382	0.033045109	0.0011809	0.001993355
Corneal stromal keratocytes	Mfn1	0.00329611	0.106294665	5.60E-05	0.000110831

Corneal stromal keratocytes	Xab2	0.003297079	0.060944431	0.0077919	0.011778951
Corneal stromal keratocytes	Ksr1	0.003300194	0.026833059	0.0064299	0.009838614
Corneal stromal keratocytes	Gatad2a	0.003319801	0.248060992	1.72E-08	4.92E-08
Corneal stromal keratocytes	Casp8ap2	0.00339323	0.071059726	0.0182935	0.026257215
Corneal stromal keratocytes	Tspan14	0.003398273	0.025862699	0.0145266	0.021203819
Corneal stromal keratocytes	Pygm	0.003404752	0.014046954	0.0071305	0.010847422
Corneal stromal keratocytes	Fxyd3	0.003450511	0.015120384	0.0005547	0.000976957
Corneal stromal keratocytes	Lingo1	0.003475996	0.116913884	2.33E-07	5.94E-07
Corneal stromal keratocytes	Pan3	0.003615746	0.102095183	6.03E-08	1.63E-07
Corneal stromal keratocytes	Foxl1	0.003638342	0.007088435	0.0241839	0.034060723
Corneal stromal keratocytes	Sos2	0.003641467	0.071282954	0.0050147	0.007797251
Corneal stromal keratocytes	Tshz3	0.003659463	0.028568861	0.0233497	0.032988435
Corneal stromal keratocytes	Mrps27	0.003678055	0.037651026	0.0063114	0.009667873
Corneal stromal keratocytes	Net1	0.003714176	0.009459982	2.49E-05	5.13E-05
Corneal stromal keratocytes	Slc9a6	0.003718281	0.085681788	0.0031203	0.004988737
Corneal stromal keratocytes	Dnajc13	0.003758173	0.142640489	0.0003519	0.000634795
Corneal stromal keratocytes	Mtx3	0.003759571	0.031019102	0.022191	0.031440068
Corneal stromal keratocytes	Il13ra1	0.003763582	0.02010026	0.0220581	0.031276984
Corneal stromal keratocytes	A930011G23Rik	0.003773655	0.020777153	0.0003296	0.000596586
Corneal stromal keratocytes	Hpf1	0.003819211	0.238933435	8.75E-11	3.04E-10
Corneal stromal keratocytes	Bend3	0.003827704	0.026600593	0.0352428	0.048398089
Corneal stromal keratocytes	Fbxl18	0.003833527	0.018792179	0.0067873	0.010350463
Corneal stromal keratocytes	Tacstd2	0.003964632	0.009165312	0.0085192	0.01282034
Corneal stromal keratocytes	Fut1	0.004000766	0.03689324	0.0099466	0.014839534
Corneal stromal keratocytes	Gprasp1	0.00406892	0.053449151	0.0052445	0.008136569
Corneal stromal keratocytes	Ltc4s	0.00407506	0.00356456	0.0266048	0.037279614
Corneal stromal keratocytes	Gm14296	0.004204039	0.037065242	0.0020145	0.00330938
Corneal stromal keratocytes	Gmcl1	0.00421784	0.055240079	0.0217488	0.030863355
Corneal stromal keratocytes	Pcp4l1	0.004220367	0.010863686	0.0029555	0.004746818
Corneal stromal keratocytes	Bdnf	0.004239783	0.041055855	0.002267	0.003705474
Corneal stromal keratocytes	Krt8	0.004375267	0.008412846	0.0341668	0.047021639
Corneal stromal keratocytes	Calml3	0.004375357	0.012521713	1.06E-11	3.95E-11
Corneal stromal keratocytes	Eml1	0.004379243	0.041325791	0.0001957	0.000363571
Corneal stromal keratocytes	Zbtb41	0.00438432	0.092802811	0.0016689	0.002766231
Corneal stromal keratocytes	Zfp644	0.004388628	0.240096129	1.07E-09	3.38E-09
Corneal stromal keratocytes	Ccnf	0.004390371	0.008050502	0.0338549	0.046619871
Corneal stromal keratocytes	Dio2	0.004395224	0.018634982	0.0062065	0.00951649
Corneal stromal keratocytes	Pibf1	0.004515605	0.048337835	0.0164569	0.023800451
Corneal stromal keratocytes	Sema4c	0.00452353	0.013314175	0.0036567	0.005794171
Corneal stromal keratocytes	Iqsec1	0.004566561	0.0799517	5.69E-05	0.000112378
Corneal stromal keratocytes	Zbtb44	0.004567097	0.082899007	1.86E-05	3.89E-05
Corneal stromal keratocytes	Lmcd1	0.004570581	0.053467279	0.0019886	0.003267855
Corneal stromal keratocytes	Tns4	0.00457942	0.009209649	0.003086	0.004940656
Corneal stromal keratocytes	Fgf18	0.00459565	0.412238054	1.44E-05	3.04E-05
Corneal stromal keratocytes	Ankrd17	0.004624553	0.382266651	2.74E-13	1.16E-12
Corneal stromal keratocytes	Oasl1	0.004649933	0.007488234	0.0023479	0.00383185
Corneal stromal keratocytes	Gps2	0.004653365	0.110856517	0.0005392	0.000951414
Corneal stromal keratocytes	Naaladl2	0.004668113	0.026827786	0.0032039	0.00511592
Corneal stromal keratocytes	Dsc3	0.0047121	0.005663637	0.0304634	0.042210399
Corneal stromal keratocytes	Cbfa2t2	0.004717101	0.052318019	0.0121272	0.017889389
Corneal stromal keratocytes	Mycbp2	0.004721566	0.378233006	1.22E-20	8.86E-20
Corneal stromal keratocytes	Mylk	0.00474559	0.042768558	0.0001316	0.000249172
Corneal stromal keratocytes	Atg2a	0.00480858	0.059378616	0.0021525	0.003525319
Corneal stromal keratocytes	Gdpd3	0.004826264	0.004137592	0.029695	0.041231183
Corneal stromal keratocytes	Taf6	0.004830525	0.060310255	0.0006094	0.001066506
Corneal stromal keratocytes	Slfn2	0.004830547	0.063259714	0.0002358	0.000434038
Corneal stromal keratocytes	Wdr70	0.004870775	0.179501086	1.93E-06	4.48E-06
Corneal stromal keratocytes	Ifi47	0.004903314	0.011094712	0.0209915	0.029876274
Corneal stromal keratocytes	Cd53	0.004909131	0.003729072	0.0302029	0.041874225
Corneal stromal keratocytes	Syn3	0.004924112	0.024414777	0.0082715	0.012466366
Corneal stromal keratocytes	Fam210b	0.004927875	0.104663882	0.0040972	0.006451379
Corneal stromal keratocytes	Herc4	0.004930537	0.044417595	0.0031681	0.005061026
Corneal stromal keratocytes	Zc3h10	0.004964525	0.056411566	0.0087386	0.013130757

Corneal stromal keratocytes	Lrrc49	0.004997432	0.113360358	2.06E-06	4.77E-06
Corneal stromal keratocytes	Agrn	0.004997512	0.046982506	0.0012377	0.002084237
Corneal stromal keratocytes	Reps1	0.005021022	0.070606078	0.0005814	0.001021629
Corneal stromal keratocytes	Paxip1	0.005028002	0.034939528	0.0205479	0.029283547
Corneal stromal keratocytes	Setdb2	0.005032854	0.006687973	0.024322	0.034244934
Corneal stromal keratocytes	Cadm4	0.005085022	0.004185116	0.0274719	0.038410265
Corneal stromal keratocytes	Optn	0.005097737	0.063627117	7.46E-05	0.000145232
Corneal stromal keratocytes	Cd82	0.005110496	0.038793562	0.0065629	0.010027984
Corneal stromal keratocytes	Itgam	0.005139203	0.003510998	0.0218995	0.031067777
Corneal stromal keratocytes	Acta2	0.005167456	0.083699623	6.62E-06	1.45E-05
Corneal stromal keratocytes	A4galt	0.005184799	0.003753028	0.012977	0.019078851
Corneal stromal keratocytes	Hspa1l	0.005193197	0.004703269	0.0076703	0.011607507
Corneal stromal keratocytes	Lmna	0.005198417	2.572234805	7.01E-05	0.000136982
Corneal stromal keratocytes	Msh3	0.005220121	0.030034051	0.0137129	0.020091355
Corneal stromal keratocytes	Trim41	0.005225266	0.059316868	0.0044079	0.006908784
Corneal stromal keratocytes	Arhgap32	0.005226618	0.071143468	0.0016901	0.002799356
Corneal stromal keratocytes	Arhgap20	0.005230819	0.039387811	0.0007363	0.001274742
Corneal stromal keratocytes	Fli1	0.005249456	0.0193046	0.0004089	0.000731276
Corneal stromal keratocytes	Mgst3	0.005251518	0.065487135	0.0036928	0.005845478
Corneal stromal keratocytes	Wdr7	0.005329704	0.023613453	0.0055511	0.008569564
Corneal stromal keratocytes	Hook2	0.005375317	0.010636058	0.0290775	0.040485841
Corneal stromal keratocytes	Fas	0.005421485	0.100057103	0.0082715	0.012466366
Corneal stromal keratocytes	Nceh1	0.005455431	0.041165531	0.0149983	0.021844702
Corneal stromal keratocytes	Ism1	0.005475906	0.0067565	0.0259411	0.036378656
Corneal stromal keratocytes	Slc25a25	0.005495702	0.060446717	0.0036917	0.005844982
Corneal stromal keratocytes	Prc1	0.005534285	0.00443746	0.0027726	0.004473643
Corneal stromal keratocytes	Slc7a6os	0.005551629	0.066597245	0.0099094	0.014788745
Corneal stromal keratocytes	Arl15	0.005551663	0.039392211	0.0026622	0.004306274
Corneal stromal keratocytes	Ckm	0.005658466	0.003785789	0.0045869	0.007169375
Corneal stromal keratocytes	Dll1	0.005661528	0.006072132	0.0272798	0.038149398
Corneal stromal keratocytes	Ptgfr	0.005673244	0.005749184	0.0141447	0.020676589
Corneal stromal keratocytes	1810024B03Rik	0.005702094	0.024821247	0.02632	0.036891634
Corneal stromal keratocytes	Bcl2a1b	0.00576583	0.005627784	0.0074748	0.01133867
Corneal stromal keratocytes	Gpc6	0.005798273	0.04977206	2.59E-06	5.92E-06
Corneal stromal keratocytes	Saa3	0.005807047	0.003942373	0.0042374	0.006660999
Corneal stromal keratocytes	Nov	0.005809865	0.008253692	0.0362643	0.049679449
Corneal stromal keratocytes	Tspan11	0.005819028	0.010179518	0.0314683	0.04351265
Corneal stromal keratocytes	Sidt2	0.005885104	0.086213245	3.35E-07	8.41E-07
Corneal stromal keratocytes	Gstm1	0.005914634	0.267283288	3.50E-05	7.09E-05
Corneal stromal keratocytes	Mdfic	0.00599944	0.095277369	0.0025111	0.004079359
Corneal stromal keratocytes	Nmt2	0.006072376	0.094236984	2.77E-06	6.31E-06
Corneal stromal keratocytes	Fry	0.006082255	0.014660069	0.0051059	0.007930217
Corneal stromal keratocytes	Pcdh18	0.006109343	0.074498922	4.76E-05	9.50E-05
Corneal stromal keratocytes	Rgs1	0.006256946	0.006143691	0.0099494	0.014842123
Corneal stromal keratocytes	Cdk6	0.006270041	0.013450723	0.0021436	0.003511098
Corneal stromal keratocytes	Elf3	0.006295554	0.00631831	0.0202208	0.028849588
Corneal stromal keratocytes	Casp3	0.006304608	0.166656562	2.44E-05	5.04E-05
Corneal stromal keratocytes	Cilp	0.006309025	0.05496189	0.0038065	0.006013838
Corneal stromal keratocytes	Aqp5	0.00631624	0.036279674	7.36E-10	2.37E-09
Corneal stromal keratocytes	Sfxn2	0.006335126	0.029858904	0.0161918	0.023439573
Corneal stromal keratocytes	Ccdc50	0.006335434	0.267765519	1.45E-08	4.16E-08
Corneal stromal keratocytes	N4bp2	0.006355225	0.082212899	1.53E-06	3.59E-06
Corneal stromal keratocytes	Tmlhe	0.006403122	0.008101554	0.0029099	0.004678422
Corneal stromal keratocytes	Ddit4	0.006490964	0.403758404	0.0001172	0.000223326
Corneal stromal keratocytes	Abcb1b	0.006551288	0.009965031	0.0250396	0.0351918
Corneal stromal keratocytes	Col6a6	0.00655819	0.007812132	0.0242145	0.03410047
Corneal stromal keratocytes	Hopx	0.006561821	0.017731052	0.0231538	0.032714948
Corneal stromal keratocytes	Tlr3	0.006649719	0.021278442	0.0029611	0.00475415
Corneal stromal keratocytes	Dapl1	0.006680487	0.017900331	6.57E-06	1.44E-05
Corneal stromal keratocytes	MT-COX2	0.006687892	3.492280062	0.0003019	0.00054919
Corneal stromal keratocytes	Lyn	0.006694864	0.006080639	0.0161389	0.023370041
Corneal stromal keratocytes	Bcap29	0.006696019	0.023245975	0.0348175	0.047860813
Corneal stromal keratocytes	Lce1g	0.006701381	0.006526774	0.0356301	0.048896483

Corneal stromal keratocytes	Pom121	0.006713631	0.178328817	4.87E-13	2.02E-12
Corneal stromal keratocytes	Fzd4	0.006724633	0.022892552	0.0299825	0.041597385
Corneal stromal keratocytes	Dynlt1b	0.006736931	0.007529846	0.0007007	0.001216747
Corneal stromal keratocytes	Grin1	0.006745239	0.00743224	0.0004485	0.00079847
Corneal stromal keratocytes	Sde2	0.006749356	0.586544056	2.77E-14	1.27E-13
Corneal stromal keratocytes	Pecam1	0.006772133	0.009059828	0.0249581	0.035080831
Corneal stromal keratocytes	Ipo8	0.006775007	0.052727018	0.0059982	0.009218191
Corneal stromal keratocytes	2900026A02Rik	0.006812867	0.046046181	1.12E-05	2.40E-05
Corneal stromal keratocytes	Tnni2	0.00682527	0.006346472	0.0219997	0.031197413
Corneal stromal keratocytes	Mc4r	0.006870481	0.010718047	0.0314882	0.043533807
Corneal stromal keratocytes	Clip2	0.006879226	0.04621971	0.0010711	0.001817171
Corneal stromal keratocytes	Alx4	0.006890508	0.007647524	0.014082	0.020595669
Corneal stromal keratocytes	Nabp1	0.00693915	0.035908083	0.0095064	0.014229715
Corneal stromal keratocytes	Zfp395	0.007004851	0.067283914	0.0003797	0.000681673
Corneal stromal keratocytes	1110038B12Rik	0.007007019	0.285360384	6.13E-06	1.35E-05
Corneal stromal keratocytes	Rnf168	0.00700813	0.085530726	0.0053538	0.008292302
Corneal stromal keratocytes	BC028528	0.007100927	0.022083684	0.0073061	0.011100617
Corneal stromal keratocytes	Sorbs1	0.007101285	0.022123777	0.0014734	0.002459908
Corneal stromal keratocytes	Hpgds	0.007115784	0.009011552	0.0075529	0.011446117
Corneal stromal keratocytes	Acta1	0.007146026	0.004521983	1.84E-06	4.28E-06
Corneal stromal keratocytes	Aldh1a3	0.007151727	0.009991399	0.0057256	0.008825418
Corneal stromal keratocytes	Sh3kbp1	0.007155688	0.061802534	0.0026228	0.004246975
Corneal stromal keratocytes	Magi3	0.007157432	0.062269287	0.0027445	0.004430697
Corneal stromal keratocytes	Adamts5	0.007242192	0.039712914	0.0202487	0.02888638
Corneal stromal keratocytes	Ccni	0.007267526	0.235370577	1.40E-10	4.80E-10
Corneal stromal keratocytes	Neurl4	0.007287857	0.026318939	0.015006	0.021853668
Corneal stromal keratocytes	9430038I01Rik	0.007308838	0.040093147	0.0074104	0.011245752
Corneal stromal keratocytes	Aif1	0.00731654	0.007968615	0.0195122	0.027900988
Corneal stromal keratocytes	Nufip2	0.007371164	0.382325988	1.26E-13	5.50E-13
Corneal stromal keratocytes	Ccne2	0.007380967	0.011683331	0.0174612	0.025170778
Corneal stromal keratocytes	Plce1	0.007389814	0.017665875	0.0118426	0.017491733
Corneal stromal keratocytes	Igsf9b	0.007404308	0.095563824	0.0015886	0.002638337
Corneal stromal keratocytes	Dcaf17	0.007410701	0.02639906	0.0332432	0.045844925
Corneal stromal keratocytes	Rimklb	0.007457369	0.012460386	0.0155737	0.022610039
Corneal stromal keratocytes	Cox7a1	0.007506966	0.01500018	0.0167294	0.024170447
Corneal stromal keratocytes	Prr5l	0.007532653	0.050999137	0.0008869	0.001521025
Corneal stromal keratocytes	Ifi204	0.007538888	0.009068082	0.0305151	0.042277909
Corneal stromal keratocytes	Adam22	0.007541167	0.037351895	0.0153514	0.02230806
Corneal stromal keratocytes	Matn2	0.007581055	0.018908993	0.0071231	0.010837836
Corneal stromal keratocytes	Csnk1g1	0.007623463	0.055315692	0.0023881	0.003892483
Corneal stromal keratocytes	Zfp512	0.00762418	0.053408663	0.0002135	0.000395293
Corneal stromal keratocytes	Fam193b	0.007632321	0.053175547	0.0004006	0.000717078
Corneal stromal keratocytes	Gpr34	0.007633256	0.004961231	0.0044968	0.007039536
Corneal stromal keratocytes	Sox18	0.007669957	0.004853526	5.15E-05	0.000102371
Corneal stromal keratocytes	Paip2b	0.007673793	0.074062782	0.0341105	0.046948749
Corneal stromal keratocytes	Bmp4	0.007685332	0.01220702	0.0023319	0.003807993
Corneal stromal keratocytes	Plekhg3	0.007759981	0.00848059	0.0077301	0.011690571
Corneal stromal keratocytes	Tuba4a	0.007774461	0.022950597	0.0013403	0.002248426
Corneal stromal keratocytes	Ptar1	0.007802315	0.140244964	4.35E-06	9.68E-06
Corneal stromal keratocytes	Gtf2e1	0.007802699	0.039789546	0.0092731	0.01390129
Corneal stromal keratocytes	Aldh1a2	0.007826444	0.025419218	0.0016969	0.002809181
Corneal stromal keratocytes	Mettl7a1	0.007830152	0.041025385	0.0009376	0.001602582
Corneal stromal keratocytes	Dcp1a	0.007862654	0.037644859	0.0015573	0.002590451
Corneal stromal keratocytes	Calcrl	0.00787125	0.161406674	0.0003639	0.000655234
Corneal stromal keratocytes	Ctla2a	0.007923149	0.007659792	0.0054072	0.008367651
Corneal stromal keratocytes	Ar	0.007970938	0.038721713	0.0024605	0.003999886
Corneal stromal keratocytes	Atp11c	0.007986158	0.024701474	0.0035965	0.005704653
Corneal stromal keratocytes	Coro1a	0.008008699	0.0083045	0.0124415	0.018328042
Corneal stromal keratocytes	Rasa1	0.008069143	0.236217378	4.54E-13	1.89E-12
Corneal stromal keratocytes	Plekha4	0.008073484	0.020759423	0.0113415	0.016795724
Corneal stromal keratocytes	Ccng2	0.008084832	0.029213714	0.0212694	0.030232006
Corneal stromal keratocytes	Slc16a9	0.008087963	0.012545581	0.0128089	0.018841658
Corneal stromal keratocytes	Lcp1	0.008102169	0.006598368	0.0024966	0.004056626

Corneal stromal keratocytes	Nr2f1	0.008224322	0.023876052	0.0004936	0.000874563
Corneal stromal keratocytes	Plek	0.008228642	0.012594049	0.0221672	0.031412675
Corneal stromal keratocytes	Vps37b	0.008267531	0.095365679	0.0363114	0.049734193
Corneal stromal keratocytes	Celf2	0.008285137	0.039653313	0.0012531	0.002108559
Corneal stromal keratocytes	Cd52	0.008367605	0.007489696	0.0031625	0.005053866
Corneal stromal keratocytes	Dennd5b	0.008374467	0.062931018	0.0003103	0.000563468
Corneal stromal keratocytes	Tspan7	0.008449444	0.10192176	3.04E-05	6.21E-05
Corneal stromal keratocytes	Bach1	0.008512889	0.311944271	1.27E-10	4.35E-10
Corneal stromal keratocytes	Scara5	0.008544071	0.013256282	0.0178738	0.025702156
Corneal stromal keratocytes	Edn1	0.008545748	0.012364901	0.0206018	0.029357351
Corneal stromal keratocytes	Arid5a	0.008551677	0.129869314	2.91E-06	6.60E-06
Corneal stromal keratocytes	Exoc2	0.008582731	0.044706878	0.0147135	0.021452113
Corneal stromal keratocytes	Map3k6	0.008584605	0.02671349	0.0337081	0.046436618
Corneal stromal keratocytes	Ptpn4	0.008632176	0.034024933	0.0158735	0.022997719
Corneal stromal keratocytes	Slc22a5	0.00863413	0.013763443	0.0073584	0.011171614
Corneal stromal keratocytes	Ncoa2	0.008649497	0.040561393	0.0246696	0.034704748
Corneal stromal keratocytes	Fcor	0.008681593	0.020735268	0.0170488	0.024606625
Corneal stromal keratocytes	Xylt1	0.008684505	0.042232035	0.0059911	0.009208339
Corneal stromal keratocytes	Pcdh1	0.008699279	0.014711915	0.0030961	0.004955178
Corneal stromal keratocytes	Adamts9	0.008707885	0.050955538	0.0010819	0.001834647
Corneal stromal keratocytes	Slc4a3	0.008725902	0.033954744	0.0013413	0.002249772
Corneal stromal keratocytes	Plagl2	0.008727788	0.041521451	0.0097976	0.014633129
Corneal stromal keratocytes	Gdf15	0.008762366	0.072753744	0.0124163	0.018294803
Corneal stromal keratocytes	Mtcl1	0.008766133	0.033594561	0.0026049	0.004220023
Corneal stromal keratocytes	Foxf1	0.008777769	0.007223789	2.51E-05	5.17E-05
Corneal stromal keratocytes	Ccl5	0.008778344	0.008904011	0.0011132	0.001885357
Corneal stromal keratocytes	Slc7a6	0.008798614	0.060987063	0.0007188	0.0012462
Corneal stromal keratocytes	Il17rd	0.008822867	0.033127507	0.0062996	0.009650845
Corneal stromal keratocytes	Kmt2a	0.008831222	0.348326973	3.26E-07	8.20E-07
Corneal stromal keratocytes	Tmem106a	0.008837748	0.015824758	0.0052888	0.008202541
Corneal stromal keratocytes	Trem2	0.008843572	0.007585173	0.0086091	0.012948629
Corneal stromal keratocytes	Glce	0.00889338	0.049543535	0.0006398	0.001116936
Corneal stromal keratocytes	Krt14	0.008943203	0.078876191	3.16E-11	1.14E-10
Corneal stromal keratocytes	Tnnt3	0.008951928	0.011033311	0.0234663	0.033146504
Corneal stromal keratocytes	Mical3	0.008976674	0.060901874	0.0075894	0.011497541
Corneal stromal keratocytes	Zfp62	0.009020017	0.088212644	2.09E-07	5.37E-07
Corneal stromal keratocytes	Armc10	0.009036655	0.02693568	0.0164804	0.02383026
Corneal stromal keratocytes	Cotl1	0.009060978	0.066251779	0.0003557	0.000641589
Corneal stromal keratocytes	Gng4	0.009092281	0.011710828	0.0018498	0.003052043
Corneal stromal keratocytes	S100a2	0.009130195	0.025245061	0.0228065	0.0322502
Corneal stromal keratocytes	Ttr	0.009160351	0.012777596	0.00615	0.009434046
Corneal stromal keratocytes	Pip5k1c	0.009225974	0.044634099	1.21E-06	2.87E-06
Corneal stromal keratocytes	Kat6b	0.009233419	0.084251031	6.64E-06	1.45E-05
Corneal stromal keratocytes	Fgf7	0.00929539	0.023479168	0.0061259	0.009401123
Corneal stromal keratocytes	Ets1	0.009307458	0.275001954	3.57E-07	8.94E-07
Corneal stromal keratocytes	Cd83	0.009370955	0.019906651	0.0056444	0.008706975
Corneal stromal keratocytes	Akap1	0.009370975	0.03601254	0.0035034	0.00556124
Corneal stromal keratocytes	Ebf1	0.009412187	0.060529615	0.0003332	0.000602665
Corneal stromal keratocytes	Slc1a2	0.00949303	0.019109386	0.0068125	0.010385549
Corneal stromal keratocytes	Ddx10	0.009495513	0.096746662	1.06E-05	2.26E-05
Corneal stromal keratocytes	Vit	0.009545771	0.016480233	0.0016937	0.002804244
Corneal stromal keratocytes	Tmprss11g	0.009550798	0.011357037	0.0028699	0.004618947
Corneal stromal keratocytes	Zfp652	0.009584847	0.127930416	0.0002038	0.000377967
Corneal stromal keratocytes	Ly86	0.009588241	0.006504962	0.001032	0.001755074
Corneal stromal keratocytes	Pla2g7	0.009598111	0.007504305	0.0002875	0.000523899
Corneal stromal keratocytes	Parp4	0.009643201	0.03798516	0.0352846	0.048446011
Corneal stromal keratocytes	Osr2	0.009659905	0.010635085	0.0050287	0.007817359
Corneal stromal keratocytes	Mtmr9	0.009700781	0.064572909	0.008291	0.012493056
Corneal stromal keratocytes	Slc7a2	0.009719882	0.024725096	0.0280153	0.039116514
Corneal stromal keratocytes	Btaf1	0.009827062	0.103693941	2.03E-06	4.71E-06
Corneal stromal keratocytes	Pim3	0.009841019	0.120905651	9.39E-05	0.000180584
Corneal stromal keratocytes	Pappa2	0.009846057	0.007688851	0.003685	0.005835777
Corneal stromal keratocytes	Filip1l	0.009856051	0.013612059	0.0032958	0.005249578

Corneal stromal keratocytes	Mtf1	0.0098761	0.091468625	0.0002627	0.000480931
Corneal stromal keratocytes	Nox4	0.009921493	0.030206702	0.0204858	0.029200981
Corneal stromal keratocytes	Alas1	0.009925368	0.06309813	0.0006283	0.001097529
Corneal stromal keratocytes	Wisp1	0.009971883	0.430202318	4.42E-10	1.45E-09
Corneal stromal keratocytes	Laptm5	0.009984509	0.007669528	0.0026484	0.004286459
Corneal stromal keratocytes	Rnf152	0.010006221	0.023412193	0.0121991	0.017991783
Corneal stromal keratocytes	Slc8a2	0.010015733	0.014696209	0.0011393	0.001927451
Corneal stromal keratocytes	Trim37	0.010022594	0.077177648	0.0007118	0.001234466
Corneal stromal keratocytes	Ccdc152	0.010138763	0.008906582	0.003167	0.00506045
Corneal stromal keratocytes	Scyl2	0.010214351	0.066786194	0.0002581	0.000472682
Corneal stromal keratocytes	Pgf	0.010325998	0.014280223	0.0073288	0.011132817
Corneal stromal keratocytes	H2-Eb1	0.010333095	0.019651547	0.0051172	0.007946078
Corneal stromal keratocytes	Hip1r	0.010336225	0.020807306	0.0054115	0.008373413
Corneal stromal keratocytes	Ecm2	0.010361712	0.066047101	6.60E-05	0.000129297
Corneal stromal keratocytes	Csf1r	0.010380473	0.012328513	0.0057284	0.008828692
Corneal stromal keratocytes	Tiam1	0.010412288	0.029650858	0.0005565	0.000980023
Corneal stromal keratocytes	Dnajc24	0.010413885	0.085087791	0.0017652	0.002916126
Corneal stromal keratocytes	Cxcr4	0.010417859	0.013433645	0.0007217	0.001250603
Corneal stromal keratocytes	Slmap	0.010468705	0.105472416	7.37E-07	1.79E-06
Corneal stromal keratocytes	Taf4	0.010503622	0.027572559	0.0049291	0.007670987
Corneal stromal keratocytes	Sned1	0.010503876	0.027270376	0.0115805	0.017126231
Corneal stromal keratocytes	Myl1	0.010519253	0.006656552	1.23E-05	2.61E-05
Corneal stromal keratocytes	Mef2c	0.010591178	0.02752115	0.0011017	0.001866439
Corneal stromal keratocytes	Abcc9	0.010595337	0.007478319	0.0001344	0.000254122
Corneal stromal keratocytes	Nedd9	0.010615901	0.10065827	0.0035981	0.005706458
Corneal stromal keratocytes	Abtb2	0.010626516	0.038991097	0.0032801	0.005226342
Corneal stromal keratocytes	Sap30	0.010654216	0.079872068	0.0035364	0.005611818
Corneal stromal keratocytes	Myof	0.010674621	0.044717261	1.26E-05	2.68E-05
Corneal stromal keratocytes	Atp1b2	0.010749959	0.025346875	0.0249194	0.035029843
Corneal stromal keratocytes	Ripor3	0.010823546	0.012620429	0.000183	0.000340913
Corneal stromal keratocytes	Lsm14b	0.010826838	0.039556578	0.0097978	0.014633129
Corneal stromal keratocytes	Slc26a2	0.010915096	0.06295897	0.022756	0.03219175
Corneal stromal keratocytes	Snx33	0.011009408	0.013224138	9.33E-05	0.000179562
Corneal stromal keratocytes	2900060B14Rik	0.011016896	0.025834181	0.0030612	0.004903654
Corneal stromal keratocytes	Tnnc2	0.011041082	0.008832356	0.0012479	0.002100609
Corneal stromal keratocytes	Sema4b	0.011042159	0.020609753	0.0170944	0.024664794
Corneal stromal keratocytes	Erc1	0.011145668	0.193710596	5.24E-08	1.43E-07
Corneal stromal keratocytes	Tmem47	0.011226858	0.053084008	0.0081493	0.012288736
Corneal stromal keratocytes	Cxcl5	0.011332413	0.02471195	0.0343933	0.04730864
Corneal stromal keratocytes	Synj1	0.01136032	0.041157676	0.0298917	0.041479684
Corneal stromal keratocytes	Yes1	0.011431317	0.074755712	0.0008397	0.001444165
Corneal stromal keratocytes	Slpi	0.011473341	0.019200961	0.0002215	0.000409383
Corneal stromal keratocytes	Irak3	0.011479613	0.029109649	0.0009061	0.001551471
Corneal stromal keratocytes	Atxn7	0.011504891	0.080725773	0.0071393	0.010857805
Corneal stromal keratocytes	Ifi207	0.011514369	0.009048007	0.0007736	0.00133632
Corneal stromal keratocytes	Nup107	0.011528718	0.018979717	0.0005362	0.000946472
Corneal stromal keratocytes	Cenpf	0.011542084	0.020801815	0.0316326	0.043718259
Corneal stromal keratocytes	Egr2	0.0115483	0.033399161	0.0011695	0.001975346
Corneal stromal keratocytes	C3	0.011598116	0.009525822	0.0001336	0.000252686
Corneal stromal keratocytes	Emcn	0.01185192	0.01567558	0.0002283	0.000420928
Corneal stromal keratocytes	Col6a5	0.011909579	0.008344125	5.29E-05	0.000104958
Corneal stromal keratocytes	Lpcat4	0.01191484	0.010672877	8.50E-09	2.50E-08
Corneal stromal keratocytes	Ddr1	0.011952174	0.049098898	0.0010555	0.001792376
Corneal stromal keratocytes	Il15ra	0.011964401	0.048509837	0.0015616	0.002597295
Corneal stromal keratocytes	Tdp2	0.011991467	0.070729032	8.35E-05	0.000161607
Corneal stromal keratocytes	Dsp	0.012007875	0.01972455	0.0002931	0.000533888
Corneal stromal keratocytes	Brd9	0.012033267	0.131374762	2.19E-05	4.53E-05
Corneal stromal keratocytes	Znrf2	0.012078186	0.062475627	0.0077204	0.011677061
Corneal stromal keratocytes	Ikzf4	0.012193451	0.0499196	0.0020917	0.003430576
Corneal stromal keratocytes	Hr	0.01219948	0.036539017	0.0159243	0.023068991
Corneal stromal keratocytes	Flt1	0.012224694	0.017343387	0.0028172	0.004540844
Corneal stromal keratocytes	Epn2	0.012248593	0.109006186	0.0083821	0.012627499
Corneal stromal keratocytes	Itga8	0.012330564	0.02550116	0.0012774	0.002146863

Corneal stromal keratocytes	Il23a	0.012400003	0.019121727	0.0144319	0.021074488
Corneal stromal keratocytes	Brd1	0.012442218	0.472714546	5.62E-15	2.72E-14
Corneal stromal keratocytes	Mtmr1	0.012462383	0.05372348	0.00025	0.000458593
Corneal stromal keratocytes	Wnt9a	0.012464367	0.022375649	0.0012311	0.002073861
Corneal stromal keratocytes	Dusp6	0.012564796	0.049307209	0.0021749	0.00356029
Corneal stromal keratocytes	Sfrp1	0.012680248	0.034544812	0.0085915	0.01292641
Corneal stromal keratocytes	Ank2	0.012725227	0.081399591	4.18E-07	1.04E-06
Corneal stromal keratocytes	Myh11	0.012728457	0.014335128	0.0017363	0.002869794
Corneal stromal keratocytes	Ap1ar	0.012738694	0.082423673	0.0001734	0.000323828
Corneal stromal keratocytes	Zcchc8	0.012785204	0.090155857	1.66E-05	3.49E-05
Corneal stromal keratocytes	Dlc1	0.012851368	0.129960074	1.74E-07	4.50E-07
Corneal stromal keratocytes	Cox15	0.012862748	0.023785316	0.0016592	0.002750799
Corneal stromal keratocytes	Atf3	0.012906934	0.536809246	4.22E-06	9.41E-06
Corneal stromal keratocytes	Ccl4	0.012916872	0.009604987	7.71E-11	2.70E-10
Corneal stromal keratocytes	Xrn1	0.012958753	0.1114412	1.97E-06	4.56E-06
Corneal stromal keratocytes	Mbtd1	0.013059476	0.066411694	0.0001792	0.00033391
Corneal stromal keratocytes	Adcy5	0.0131256	0.037459126	0.0010129	0.001724679
Corneal stromal keratocytes	Prnp	0.013129596	0.191250094	5.74E-07	1.41E-06
Corneal stromal keratocytes	Tceanc2	0.013178865	0.047273329	0.007677	0.011616449
Corneal stromal keratocytes	Atxn7l1	0.013193922	0.025101165	0.0006463	0.001127254
Corneal stromal keratocytes	Elac1	0.013285268	0.031697615	0.0033698	0.005360787
Corneal stromal keratocytes	Srgn	0.013356776	0.023495818	0.0045327	0.007094116
Corneal stromal keratocytes	Tbx3	0.013390047	0.033664266	0.0006637	0.001155977
Corneal stromal keratocytes	Chuk	0.013392534	0.097411531	0.000103	0.000197399
Corneal stromal keratocytes	Ccdc73	0.013417848	0.033746612	0.0034855	0.005534106
Corneal stromal keratocytes	Diaph2	0.013457461	0.092410393	0.0003577	0.000644878
Corneal stromal keratocytes	C1qc	0.013537902	0.011896822	0.00061	0.001067321
Corneal stromal keratocytes	Crlf1	0.013627209	0.075422576	0.0003599	0.000648569
Corneal stromal keratocytes	Ace	0.013682571	0.011516931	0.0003087	0.000560944
Corneal stromal keratocytes	Enpp1	0.013758945	0.048916813	0.0041987	0.006603857
Corneal stromal keratocytes	Foxo3	0.013826965	0.13687586	6.00E-05	0.000118143
Corneal stromal keratocytes	Crebrf	0.013830265	0.059610116	5.05E-05	0.000100454
Corneal stromal keratocytes	Taf4b	0.01383731	0.056290577	0.0055465	0.008563489
Corneal stromal keratocytes	Nptx1	0.013845323	0.5331629	2.85E-10	9.48E-10
Corneal stromal keratocytes	Ptpn2	0.013850084	0.125873929	3.69E-06	8.27E-06
Corneal stromal keratocytes	Mylpf	0.013883336	0.016997071	0.0004636	0.000823462
Corneal stromal keratocytes	D6Wsu163e	0.013893131	0.046262496	0.0012631	0.002124753
Corneal stromal keratocytes	Slc6a17	0.013949824	0.016458569	1.94E-05	4.04E-05
Corneal stromal keratocytes	Ptch1	0.014067811	0.038376246	0.0061032	0.009369576
Corneal stromal keratocytes	Col20a1	0.014124542	0.027018766	0.000288	0.000524668
Corneal stromal keratocytes	Cish	0.014222125	0.033218516	0.0003916	0.000701868
Corneal stromal keratocytes	Ly6a	0.01435124	0.050628194	0.000486	0.000861709
Corneal stromal keratocytes	Cx3cr1	0.014385613	0.01942792	0.0162603	0.023531377
Corneal stromal keratocytes	Ccl11	0.014424171	0.024264846	0.0012652	0.002127384
Corneal stromal keratocytes	Ttbk2	0.014482468	0.060514967	0.0194368	0.027801664
Corneal stromal keratocytes	Dbi	0.014486016	1.056448715	9.24E-31	1.27E-29
Corneal stromal keratocytes	Tbc1d1	0.014510088	0.099868857	3.23E-05	6.57E-05
Corneal stromal keratocytes	Epas1	0.014532301	0.115792914	2.09E-08	5.91E-08
Corneal stromal keratocytes	Nck2	0.014533142	0.034096685	0.0009553	0.001631071
Corneal stromal keratocytes	H2-Aa	0.01454772	0.015903086	2.16E-06	4.98E-06
Corneal stromal keratocytes	Igf1	0.014570346	0.113166943	6.53E-13	2.68E-12
Corneal stromal keratocytes	Srf	0.014701416	0.035425332	0.0010866	0.001842215
Corneal stromal keratocytes	Prpf39	0.014721549	0.116918415	1.29E-07	3.39E-07
Corneal stromal keratocytes	Senp2	0.014727154	0.276935296	6.84E-11	2.41E-10
Corneal stromal keratocytes	Hivep3	0.014730851	0.212078513	3.98E-08	1.09E-07
Corneal stromal keratocytes	Dgkh	0.014802605	0.073557058	0.0001648	0.000308602
Corneal stromal keratocytes	Trip4	0.014925173	0.063526148	0.0004542	0.000807757
Corneal stromal keratocytes	Stat2	0.014956001	0.0944305	0.0104469	0.015547954
Corneal stromal keratocytes	Tet2	0.014962693	0.087778354	0.0032684	0.005210078
Corneal stromal keratocytes	Mepce	0.015005014	0.054187412	0.0098775	0.014745837
Corneal stromal keratocytes	Wwp1	0.015007378	0.069518234	0.0010107	0.001721101
Corneal stromal keratocytes	Omd	0.015017879	0.072687394	0.002659	0.004301581
Corneal stromal keratocytes	Col18a1	0.015055836	0.083276366	8.77E-05	0.000169346

Corneal stromal keratocytes	Pde8a	0.015075037	0.022135308	0.0001099	0.000209931
Corneal stromal keratocytes	Tle4	0.015171754	0.091902487	0.0001027	0.000196926
Corneal stromal keratocytes	Cacna1d	0.015212149	0.042216998	0.0055674	0.008592843
Corneal stromal keratocytes	Dact1	0.01522842	0.043831805	0.0157593	0.022846456
Corneal stromal keratocytes	Tnxb	0.015347633	0.09464099	0.0009455	0.001615485
Corneal stromal keratocytes	Lima1	0.015441674	0.703297231	1.85E-15	9.30E-15
Corneal stromal keratocytes	Setbp1	0.015540211	0.162237229	1.07E-09	3.38E-09
Corneal stromal keratocytes	Gpr4	0.015543562	0.135468443	4.59E-05	9.18E-05
Corneal stromal keratocytes	Kcnn3	0.01554479	0.047445039	1.40E-05	2.96E-05
Corneal stromal keratocytes	Gcc1	0.015566396	0.112000799	2.90E-05	5.93E-05
Corneal stromal keratocytes	Cops2	0.015593458	0.306457384	5.36E-11	1.90E-10
Corneal stromal keratocytes	8030462N17Rik	0.01566573	0.105731783	6.07E-05	0.000119417
Corneal stromal keratocytes	Nppc	0.015735139	0.016016066	5.09E-05	0.000101328
Corneal stromal keratocytes	Ankmy1	0.015735625	0.02089401	5.77E-05	0.000113862
Corneal stromal keratocytes	Sema7a	0.01574705	0.025930714	0.0003828	0.00068674
Corneal stromal keratocytes	Aspn	0.015762315	0.054621811	0.0001039	0.000198883
Corneal stromal keratocytes	Pds5b	0.015783652	0.141646055	5.94E-06	1.31E-05
Corneal stromal keratocytes	Ifit2	0.015814917	0.069044522	0.0073329	0.01113665
Corneal stromal keratocytes	Syne2	0.015843085	0.030600347	8.32E-05	0.000160964
Corneal stromal keratocytes	Pax1	0.015902716	0.016939514	3.93E-05	7.92E-05
Corneal stromal keratocytes	Tgfbi	0.016003298	0.871590486	5.88E-12	2.24E-11
Corneal stromal keratocytes	Scube1	0.016004252	0.022040992	0.0001705	0.000318643
Corneal stromal keratocytes	Pnpt1	0.01602184	0.035555606	0.0006822	0.001185681
Corneal stromal keratocytes	Kdm6b	0.016041327	0.729904262	4.29E-18	2.62E-17
Corneal stromal keratocytes	Col8a1	0.016042163	0.092214986	0.003436	0.005461794
Corneal stromal keratocytes	Mpeg1	0.016072327	0.034749767	0.0008765	0.001504437
Corneal stromal keratocytes	Lrp8	0.016087241	0.0492092	0.004226	0.006646049
Corneal stromal keratocytes	Usp53	0.016105562	0.043983404	0.0002544	0.000466319
Corneal stromal keratocytes	Olfml2b	0.016109957	0.016768582	0.0001357	0.000256379
Corneal stromal keratocytes	Zfp87	0.016115448	0.048889635	0.0054308	0.008400551
Corneal stromal keratocytes	Plvap	0.016127469	0.020314294	0.0002156	0.000398936
Corneal stromal keratocytes	Cnot9	0.01619417	0.04910856	0.0006803	0.001182597
Corneal stromal keratocytes	Bicd2	0.016304218	0.199757107	5.63E-12	2.15E-11
Corneal stromal keratocytes	Atp8b1	0.01630661	0.024352331	0.0003255	0.000589345
Corneal stromal keratocytes	Rbbp5	0.016336129	0.039758133	7.09E-05	0.000138347
Corneal stromal keratocytes	Fyn	0.016347043	0.031311602	0.0002016	0.000374051
Corneal stromal keratocytes	Zfp60	0.016463697	0.050535177	0.0013705	0.00229681
Corneal stromal keratocytes	Islr	0.016490541	0.463635826	5.98E-26	6.09E-25
Corneal stromal keratocytes	Naa20	0.016554135	0.297601143	7.83E-07	1.89E-06
Corneal stromal keratocytes	Cd274	0.016624846	0.034188188	0.0001157	0.000220618
Corneal stromal keratocytes	Fcer1g	0.016658954	0.017968321	0.0003425	0.000618469
Corneal stromal keratocytes	Rsad2	0.016676982	0.017108354	0.0008644	0.001484789
Corneal stromal keratocytes	Ypel2	0.016689673	0.094542824	0.0007802	0.001347033
Corneal stromal keratocytes	Trf	0.016716365	0.119378665	0.0005402	0.000953056
Corneal stromal keratocytes	Chst8	0.016748378	0.068993202	0.0006675	0.001161887
Corneal stromal keratocytes	Nkd1	0.016902638	0.095061599	0.0062707	0.009608629
Corneal stromal keratocytes	Esm1	0.016907805	0.016332882	0.0005584	0.000982999
Corneal stromal keratocytes	BC100530	0.016913099	0.028463548	0.0008579	0.001474206
Corneal stromal keratocytes	Camp	0.016991697	0.011393234	1.11E-05	2.37E-05
Corneal stromal keratocytes	Fabp4	0.017017109	0.016054477	0.0002705	0.000494487
Corneal stromal keratocytes	Usp31	0.017018561	0.080202738	0.0032704	0.005212634
Corneal stromal keratocytes	Agfg2	0.017033898	0.079860382	0.0305668	0.042341121
Corneal stromal keratocytes	St5	0.017047046	0.051362019	0.0023486	0.003832615
Corneal stromal keratocytes	Npnt	0.01710392	0.028286635	0.0001686	0.000315326
Corneal stromal keratocytes	Emb	0.017127763	0.049412532	0.0069585	0.010600132
Corneal stromal keratocytes	Alkal2	0.017169255	0.151192325	0.0255196	0.035827034
Corneal stromal keratocytes	Map3k8	0.017173026	0.058735525	0.0046039	0.00719269
Corneal stromal keratocytes	Perp	0.017265242	0.054053397	1.32E-06	3.11E-06
Corneal stromal keratocytes	Mob1a	0.017293677	0.257278901	1.69E-12	6.72E-12
Corneal stromal keratocytes	Bcar3	0.017303045	0.193451909	9.26E-08	2.47E-07
Corneal stromal keratocytes	Hivep1	0.017331253	0.145143685	1.32E-09	4.15E-09
Corneal stromal keratocytes	Map3k1	0.017392981	0.045668559	4.69E-05	9.37E-05
Corneal stromal keratocytes	Fam53c	0.017394333	0.095939818	2.73E-05	5.61E-05

Corneal stromal keratocytes	S1pr1	0.017405038	0.01939874	4.01E-06	8.96E-06
Corneal stromal keratocytes	Wisp2	0.017592814	0.017819073	5.88E-06	1.29E-05
Corneal stromal keratocytes	Tcp11l2	0.017634427	0.106851141	0.0003529	0.000636649
Corneal stromal keratocytes	Ghitm	0.017660969	0.530895287	5.72E-19	3.70E-18
Corneal stromal keratocytes	Mterf3	0.017702544	0.076730568	0.0001183	0.000225215
Corneal stromal keratocytes	Stc1	0.017702863	0.075283863	0.00744	0.011289447
Corneal stromal keratocytes	Olfr655	0.017714386	0.027067073	0.0076157	0.01153235
Corneal stromal keratocytes	G530011O06Rik	0.017720461	0.118669505	0.0004859	0.00086165
Corneal stromal keratocytes	Prss23	0.017724208	0.117325919	3.35E-05	6.80E-05
Corneal stromal keratocytes	C1qb	0.017783613	0.021548368	8.47E-05	0.000163617
Corneal stromal keratocytes	Unkl	0.017849096	0.037479597	0.0001586	0.000297398
Corneal stromal keratocytes	Mfsd4a	0.017955056	0.028449582	6.37E-06	1.40E-05
Corneal stromal keratocytes	Lypd2	0.017967414	0.065087129	6.48E-16	3.37E-15
Corneal stromal keratocytes	Gbp2	0.017975872	0.051665273	0.0108931	0.016160593
Corneal stromal keratocytes	Krt5	0.018074529	0.094604296	7.00E-20	4.81E-19
Corneal stromal keratocytes	Epyc	0.018109121	0.012549957	2.94E-07	7.43E-07
Corneal stromal keratocytes	Flcn	0.018128109	0.059669545	0.0011392	0.001927451
Corneal stromal keratocytes	Heyl	0.018212682	0.038474563	0.0004326	0.000771955
Corneal stromal keratocytes	Bmp2	0.018258892	0.124525889	0.0005567	0.000980189
Corneal stromal keratocytes	Wif1	0.01826716	0.068072244	0.0002421	0.000444894
Corneal stromal keratocytes	Cspg4	0.018458386	0.03589867	0.0002167	0.00040086
Corneal stromal keratocytes	Agtpbp1	0.018565346	0.148759197	0.0002257	0.000416405
Corneal stromal keratocytes	H2-Q4	0.018609787	0.153324423	2.60E-06	5.94E-06
Corneal stromal keratocytes	Strn	0.018627996	0.059560393	0.0004876	0.000864389
Corneal stromal keratocytes	Ppp1r13l	0.018685571	0.130476322	1.64E-06	3.84E-06
Corneal stromal keratocytes	Angpt1	0.018852459	0.038525674	0.0003374	0.000609842
Corneal stromal keratocytes	Cbl	0.018864671	0.149582817	2.33E-05	4.82E-05
Corneal stromal keratocytes	Ednra	0.018868489	0.063801574	0.0008037	0.001385246
Corneal stromal keratocytes	Ltbp2	0.018871152	0.057303219	9.98E-05	0.000191599
Corneal stromal keratocytes	Nhsl1	0.019013341	0.054780075	7.29E-05	0.000141991
Corneal stromal keratocytes	Prpf4b	0.019017184	0.380976107	4.68E-13	1.94E-12
Corneal stromal keratocytes	Dusp18	0.019083462	0.109081907	0.0148498	0.021635128
Corneal stromal keratocytes	Smoc1	0.019166872	0.039817017	0.000181	0.000337138
Corneal stromal keratocytes	Sgms2	0.019247844	0.120362769	1.42E-05	3.01E-05
Corneal stromal keratocytes	Klf3	0.01929244	0.248705897	4.95E-05	9.86E-05
Corneal stromal keratocytes	Hnrnpa1	0.019441495	0.685263655	5.85E-22	4.66E-21
Corneal stromal keratocytes	Col17a1	0.019506311	0.032093736	0.0004742	0.000841612
Corneal stromal keratocytes	2310009B15Rik	0.019612382	0.303138055	1.54E-08	4.42E-08
Corneal stromal keratocytes	Fdx1	0.019616027	0.359340263	8.26E-07	1.99E-06
Corneal stromal keratocytes	Clk4	0.019915021	0.250308999	2.28E-08	6.41E-08
Corneal stromal keratocytes	Adnp	0.020103476	0.252055283	9.26E-07	2.22E-06
Corneal stromal keratocytes	Swap70	0.020445782	0.07399695	2.80E-06	6.37E-06
Corneal stromal keratocytes	Grk2	0.020529089	0.127377051	2.09E-06	4.84E-06
Corneal stromal keratocytes	Cd14	0.020548615	0.026945558	4.40E-05	8.83E-05
Corneal stromal keratocytes	Il17ra	0.020549128	0.044322784	0.0001251	0.000237311
Corneal stromal keratocytes	Arid4a	0.020590758	0.064983032	0.0003828	0.00068674
Corneal stromal keratocytes	Lsp1	0.020662508	0.168320625	3.03E-06	6.86E-06
Corneal stromal keratocytes	Fzd7	0.020679772	0.060915627	0.0001566	0.000293873
Corneal stromal keratocytes	Gpx3	0.020685178	0.025299177	0.0003683	0.000662404
Corneal stromal keratocytes	Eln	0.020692905	0.054056065	0.0237146	0.033476988
Corneal stromal keratocytes	S100a8	0.02074885	0.042437288	0.013085	0.019219595
Corneal stromal keratocytes	Gpkow	0.020781161	0.047293802	7.56E-05	0.000147121
Corneal stromal keratocytes	Litaf	0.020870587	0.51518336	1.42E-06	3.34E-06
Corneal stromal keratocytes	Gtf2a1	0.020899043	0.090672285	0.0003217	0.000583126
Corneal stromal keratocytes	Sirt1	0.020917061	0.10117952	1.50E-06	3.53E-06
Corneal stromal keratocytes	Tyrobp	0.02093901	0.021542727	8.35E-05	0.000161593
Corneal stromal keratocytes	Midn	0.021021853	0.184993245	0.0001237	0.00023483
Corneal stromal keratocytes	Slc9a1	0.02105068	0.238973605	1.18E-07	3.11E-07
Corneal stromal keratocytes	B3gnt2	0.021140148	0.452447579	5.40E-12	2.07E-11
Corneal stromal keratocytes	C1qa	0.021425145	0.022204254	3.63E-05	7.33E-05
Corneal stromal keratocytes	Pde3a	0.021438547	0.112184893	0.0001587	0.000297531
Corneal stromal keratocytes	Nr2f2	0.021457645	0.061900894	1.86E-06	4.32E-06
Corneal stromal keratocytes	Hes1	0.021488003	0.256946489	0.000775	0.001338528

Corneal stromal keratocytes	Arhgef10l	0.021606433	0.043753123	3.02E-05	6.16E-05
Corneal stromal keratocytes	Ifi202b	0.021627308	0.059604012	0.0178209	0.025649715
Corneal stromal keratocytes	Rabgef1	0.02174144	0.224147622	9.41E-06	2.02E-05
Corneal stromal keratocytes	Atg14	0.02188368	0.048624402	1.90E-05	3.96E-05
Corneal stromal keratocytes	Pfkp	0.021891146	0.11422998	0.0023998	0.003908839
Corneal stromal keratocytes	Pdyn	0.021943949	0.031329214	3.29E-05	6.67E-05
Corneal stromal keratocytes	Helz2	0.022001885	0.077012546	1.30E-05	2.76E-05
Corneal stromal keratocytes	Inhba	0.022218612	0.056906431	0.0043234	0.006784011
Corneal stromal keratocytes	MT-ND6	0.022351002	0.058606116	2.13E-05	4.43E-05
Corneal stromal keratocytes	Amotl2	0.022480626	0.116402868	3.39E-07	8.51E-07
Corneal stromal keratocytes	Clk1	0.022537962	0.249285212	7.13E-07	1.73E-06
Corneal stromal keratocytes	Cdc42ep3	0.022553127	0.271173446	3.19E-07	8.03E-07
Corneal stromal keratocytes	Tacc1	0.022744698	0.245725874	2.87E-08	8.00E-08
Corneal stromal keratocytes	Cygb	0.022761028	0.237789786	9.55E-12	3.57E-11
Corneal stromal keratocytes	Fzd5	0.022778986	0.102333571	0.0006092	0.001066407
Corneal stromal keratocytes	Gprc5a	0.023027744	0.164026475	0.0047243	0.007370179
Corneal stromal keratocytes	Syde2	0.023087624	0.074599483	0.0007868	0.001357812
Corneal stromal keratocytes	Rhoj	0.023102189	0.680045095	1.39E-20	1.01E-19
Corneal stromal keratocytes	Pim1	0.023151932	0.686706651	1.43E-08	4.11E-08
Corneal stromal keratocytes	Slc35e2	0.023272985	0.044598012	4.35E-06	9.68E-06
Corneal stromal keratocytes	Dhx9	0.023321352	0.321043033	5.50E-10	1.79E-09
Corneal stromal keratocytes	Trak2	0.023343111	0.175841865	1.66E-05	3.49E-05
Corneal stromal keratocytes	Gas6	0.023477914	0.1716184	0.0010364	0.001762006
Corneal stromal keratocytes	Baz2a	0.023498512	0.088308376	2.20E-06	5.08E-06
Corneal stromal keratocytes	Arid5b	0.023518526	0.228974965	6.14E-12	2.34E-11
Corneal stromal keratocytes	Gpalpp1	0.023570011	0.087732396	2.82E-05	5.78E-05
Corneal stromal keratocytes	Snx25	0.023704865	0.069269663	0.0001274	0.000241632
Corneal stromal keratocytes	Bcl9l	0.02396331	0.072816189	0.0002526	0.000463266
Corneal stromal keratocytes	Nbr1	0.023973746	0.171929482	9.15E-05	0.000176197
Corneal stromal keratocytes	Lmnb1	0.02400752	0.16692791	0.0077376	0.011700539
Corneal stromal keratocytes	Il1r1	0.024075555	0.081241669	4.12E-06	9.20E-06
Corneal stromal keratocytes	Thap1	0.024089353	0.060807077	2.14E-06	4.94E-06
Corneal stromal keratocytes	Rbm22	0.024179587	0.326838184	2.32E-08	6.53E-08
Corneal stromal keratocytes	Pqlc1	0.024185166	0.455320626	1.40E-13	6.09E-13
Corneal stromal keratocytes	Nckap5l	0.02433359	0.038043374	1.01E-06	2.40E-06
Corneal stromal keratocytes	Dync1i1	0.02459858	0.117589626	3.56E-05	7.20E-05
Corneal stromal keratocytes	Cpeb2	0.024658783	0.072721767	4.73E-07	1.17E-06
Corneal stromal keratocytes	Arhgef11	0.024699273	0.03718819	1.29E-08	3.75E-08
Corneal stromal keratocytes	Jag1	0.024861401	0.047342125	2.34E-05	4.84E-05
Corneal stromal keratocytes	Zbtb10	0.025108216	0.072627446	9.35E-06	2.01E-05
Corneal stromal keratocytes	Itpr1	0.025163059	0.20701902	1.92E-07	4.96E-07
Corneal stromal keratocytes	Prkcd	0.025313678	0.161859409	1.91E-05	3.99E-05
Corneal stromal keratocytes	Tmem167b	0.025364385	0.156899338	8.81E-06	1.90E-05
Corneal stromal keratocytes	Adamts4	0.025452881	0.054495471	0.0004291	0.000765999
Corneal stromal keratocytes	Tob1	0.025507101	0.23519365	0.0006437	0.001123087
Corneal stromal keratocytes	Magohb	0.025636372	0.079656121	3.07E-06	6.95E-06
Corneal stromal keratocytes	4921524J17Rik	0.02563942	0.153764601	1.01E-07	2.68E-07
Corneal stromal keratocytes	Clasp2	0.025672672	0.107538236	1.97E-07	5.08E-07
Corneal stromal keratocytes	Bambi	0.02567585	0.07721945	0.0027219	0.004397875
Corneal stromal keratocytes	Plagl1	0.025813887	0.09591602	0.0030944	0.004953001
Corneal stromal keratocytes	Nectin1	0.025890218	0.074897521	3.25E-06	7.32E-06
Corneal stromal keratocytes	Eloa	0.025939217	0.236893259	7.03E-07	1.71E-06
Corneal stromal keratocytes	Col4a3bp	0.026038212	0.085548853	6.33E-06	1.39E-05
Corneal stromal keratocytes	Neto2	0.026493533	0.139694445	3.08E-08	8.55E-08
Corneal stromal keratocytes	Pop4	0.026617976	0.069406784	8.11E-07	1.96E-06
Corneal stromal keratocytes	Ampd2	0.026767758	0.083725591	1.91E-05	4.00E-05
Corneal stromal keratocytes	Socs3	0.026848436	0.313611321	7.90E-05	0.000153418
Corneal stromal keratocytes	Kdm5a	0.026868765	0.192767498	3.49E-10	1.16E-09
Corneal stromal keratocytes	Snrnp200	0.026874298	0.149916187	9.74E-07	2.33E-06
Corneal stromal keratocytes	44450	0.027034159	0.292536445	4.51E-11	1.61E-10
Corneal stromal keratocytes	Rnd3	0.027097248	0.188085651	4.28E-13	1.78E-12
Corneal stromal keratocytes	Fbxo30	0.027309822	0.146954586	9.14E-05	0.000176185
Corneal stromal keratocytes	Itga3	0.027464856	0.08303024	3.28E-05	6.65E-05

Corneal stromal keratocytes	Trim2	0.027514601	0.252632828	2.34E-07	5.97E-07
Corneal stromal keratocytes	Npr3	0.027535566	0.370769356	2.10E-22	1.72E-21
Corneal stromal keratocytes	Cstb	0.027562485	1.051532262	7.09E-08	1.91E-07
Corneal stromal keratocytes	Zc3h7b	0.027671407	0.154390514	1.07E-06	2.56E-06
Corneal stromal keratocytes	Sfrp4	0.027674326	0.020233119	1.37E-08	3.97E-08
Corneal stromal keratocytes	Irs2	0.027773341	0.137388664	1.44E-06	3.39E-06
Corneal stromal keratocytes	Rasl11b	0.027836692	0.229342788	0.000303	0.000550988
Corneal stromal keratocytes	S100a10	0.02808287	1.614499101	5.42E-16	2.83E-15
Corneal stromal keratocytes	Hba-a2	0.028104652	0.039964301	2.15E-07	5.52E-07
Corneal stromal keratocytes	Krt16	0.028108572	0.041268284	4.44E-12	1.71E-11
Corneal stromal keratocytes	Slfn5	0.02835753	0.109064439	0.0001388	0.000261965
Corneal stromal keratocytes	Plxna2	0.028503846	0.290458482	1.24E-09	3.91E-09
Corneal stromal keratocytes	Nab2	0.028706679	0.215323533	2.34E-05	4.84E-05
Corneal stromal keratocytes	Rab8b	0.028709166	0.078028597	5.46E-06	1.20E-05
Corneal stromal keratocytes	Aqp3	0.028798427	0.041991417	2.83E-07	7.17E-07
Corneal stromal keratocytes	Ubash3a	0.028932018	0.039030707	2.76E-06	6.28E-06
Corneal stromal keratocytes	Klf10	0.02899593	0.283393259	2.20E-12	8.68E-12
Corneal stromal keratocytes	Col15a1	0.029083613	0.040102077	2.72E-05	5.58E-05
Corneal stromal keratocytes	Rnf213	0.029092204	0.182163309	9.52E-10	3.02E-09
Corneal stromal keratocytes	Tagln	0.02912658	0.07447049	0.0007418	0.001284106
Corneal stromal keratocytes	Zbtb1	0.029151321	0.186802083	6.66E-10	2.15E-09
Corneal stromal keratocytes	Tmtc1	0.029157412	0.049885523	3.33E-07	8.37E-07
Corneal stromal keratocytes	Iigp1	0.02919216	0.147825572	0.0059849	0.009201805
Corneal stromal keratocytes	Bmpr1a	0.029458432	0.325989035	8.59E-18	5.15E-17
Corneal stromal keratocytes	Nol4l	0.0299077	0.04268268	6.19E-06	1.36E-05
Corneal stromal keratocytes	Dbndd2	0.030034031	0.199788861	2.24E-09	6.90E-09
Corneal stromal keratocytes	Kctd1	0.030129154	0.189591928	1.01E-07	2.68E-07
Corneal stromal keratocytes	Il11	0.030388951	0.115368848	0.0001506	0.000283102
Corneal stromal keratocytes	Mmp19	0.030546771	0.184855282	1.06E-06	2.53E-06
Corneal stromal keratocytes	Fndc1	0.030616918	0.05831722	1.99E-05	4.15E-05
Corneal stromal keratocytes	Sash1	0.030655856	0.235182296	1.72E-15	8.62E-15
Corneal stromal keratocytes	Fnip2	0.030723193	0.05789056	6.86E-09	2.04E-08
Corneal stromal keratocytes	Sowahc	0.030927198	0.15066199	4.10E-05	8.23E-05
Corneal stromal keratocytes	Arhgap29	0.03101789	0.262866323	6.15E-08	1.66E-07
Corneal stromal keratocytes	Prg4	0.031245911	0.066450758	0.0011462	0.001937968
Corneal stromal keratocytes	Sik1	0.031307601	0.083823876	5.33E-06	1.18E-05
Corneal stromal keratocytes	Mdm2	0.031415336	0.506558855	6.57E-08	1.77E-07
Corneal stromal keratocytes	Rin2	0.031498719	0.292908938	7.86E-08	2.11E-07
Corneal stromal keratocytes	Nfatc1	0.031569197	0.303724229	3.38E-10	1.12E-09
Corneal stromal keratocytes	Ppl	0.031699793	0.060452313	3.43E-09	1.04E-08
Corneal stromal keratocytes	Cdkn1b	0.031755206	0.072848285	2.62E-07	6.66E-07
Corneal stromal keratocytes	Ndel1	0.031757361	0.326333428	1.63E-12	6.50E-12
Corneal stromal keratocytes	Myoc	0.031922761	0.050579683	0.0005889	0.001033532
Corneal stromal keratocytes	Gls	0.032707598	0.435582422	1.34E-15	6.76E-15
Corneal stromal keratocytes	Sp1	0.032976124	0.140655686	1.73E-07	4.47E-07
Corneal stromal keratocytes	Znfx1	0.033055461	0.109009678	3.74E-07	9.34E-07
Corneal stromal keratocytes	Fat4	0.03307868	0.052771597	2.94E-08	8.19E-08
Corneal stromal keratocytes	Golga4	0.03326068	0.386196869	5.91E-10	1.91E-09
Corneal stromal keratocytes	Pxdc1	0.033327066	0.80033456	2.05E-08	5.79E-08
Corneal stromal keratocytes	Rabif	0.033338328	0.104617907	1.74E-05	3.66E-05
Corneal stromal keratocytes	Tgfb3	0.033542211	0.177030639	1.94E-05	4.05E-05
Corneal stromal keratocytes	Fcho2	0.03365807	0.134001788	3.09E-07	7.78E-07
Corneal stromal keratocytes	Cd47	0.033936817	0.383018677	6.00E-12	2.29E-11
Corneal stromal keratocytes	Rab22a	0.033954686	0.254145101	2.04E-08	5.78E-08
Corneal stromal keratocytes	Abhd2	0.034196738	0.081200537	3.62E-07	9.06E-07
Corneal stromal keratocytes	Map4k4	0.03422152	0.624994533	3.71E-16	1.96E-15
Corneal stromal keratocytes	Foxo1	0.034391699	0.381138895	3.10E-12	1.21E-11
Corneal stromal keratocytes	Cx3cl1	0.034536197	0.108847972	1.16E-05	2.48E-05
Corneal stromal keratocytes	Gdf10	0.034860666	0.132237827	0.0002029	0.00037648
Corneal stromal keratocytes	Rmnd5a	0.03517035	0.130486838	9.00E-08	2.40E-07
Corneal stromal keratocytes	Map4	0.035277783	0.231994779	1.72E-08	4.91E-08
Corneal stromal keratocytes	Ubash3b	0.035387458	0.171686544	3.49E-06	7.86E-06
Corneal stromal keratocytes	Erbin	0.035457908	0.435470658	1.13E-11	4.22E-11

Corneal stromal keratocytes	Gabpa	0.035727084	0.118000718	6.14E-08	1.66E-07
Corneal stromal keratocytes	Ncoa3	0.035759623	0.196657153	1.16E-11	4.31E-11
Corneal stromal keratocytes	Ccnl1	0.035975014	0.568038422	7.62E-15	3.64E-14
Corneal stromal keratocytes	Cd74	0.036495609	0.04321276	7.77E-10	2.49E-09
Corneal stromal keratocytes	Igsf3	0.03658442	0.124723244	1.65E-07	4.29E-07
Corneal stromal keratocytes	Erf	0.036949926	0.164126204	2.01E-08	5.68E-08
Corneal stromal keratocytes	Ece1	0.036990636	0.168736688	1.89E-08	5.37E-08
Corneal stromal keratocytes	Mxd1	0.037074509	0.073349977	2.14E-08	6.03E-08
Corneal stromal keratocytes	Fmr1	0.037105013	0.269930096	3.94E-09	1.19E-08
Corneal stromal keratocytes	Cd24a	0.037187626	0.11308422	8.82E-06	1.90E-05
Corneal stromal keratocytes	Dtx3l	0.037365776	0.104149394	3.51E-07	8.80E-07
Corneal stromal keratocytes	Slc12a2	0.03748092	0.220348678	4.97E-06	1.10E-05
Corneal stromal keratocytes	Tgif1	0.037560656	0.29536123	9.50E-11	3.29E-10
Corneal stromal keratocytes	Pdpn	0.037570832	0.875277536	3.26E-19	2.15E-18
Corneal stromal keratocytes	Rnf103	0.038063025	0.126690746	4.42E-09	1.33E-08
Corneal stromal keratocytes	Igf2	0.038909957	0.074630884	1.84E-06	4.28E-06
Corneal stromal keratocytes	Pcf11	0.0389404	0.184003383	8.05E-08	2.16E-07
Corneal stromal keratocytes	Ascc3	0.039119921	0.376994534	1.11E-15	5.65E-15
Corneal stromal keratocytes	Trim56	0.039748987	0.160877359	3.32E-08	9.19E-08
Corneal stromal keratocytes	Pthlh	0.039780514	0.044982717	1.37E-08	3.95E-08
Corneal stromal keratocytes	Tmod3	0.039794028	0.326299353	1.99E-12	7.85E-12
Corneal stromal keratocytes	Nfia	0.039803785	0.712028903	5.32E-19	3.45E-18
Corneal stromal keratocytes	Itga1	0.039826572	0.170366988	3.75E-07	9.35E-07
Corneal stromal keratocytes	Cryab	0.03991623	2.262633729	9.77E-07	2.34E-06
Corneal stromal keratocytes	Itga5	0.040111488	0.423530322	2.70E-12	1.06E-11
Corneal stromal keratocytes	Col4a3	0.041117927	0.052756031	8.10E-13	3.30E-12
Corneal stromal keratocytes	Ash1l	0.041653003	0.464901172	3.14E-14	1.44E-13
Corneal stromal keratocytes	Rictor	0.042258157	0.114390028	4.14E-10	1.36E-09
Corneal stromal keratocytes	Pitrm1	0.042274475	0.146085798	1.79E-08	5.10E-08
Corneal stromal keratocytes	Pak1	0.042489904	0.137250525	1.72E-09	5.36E-09
Corneal stromal keratocytes	Ctss	0.042495357	0.030276722	4.58E-12	1.76E-11
Corneal stromal keratocytes	Coch	0.042617869	0.054604407	4.03E-14	1.82E-13
Corneal stromal keratocytes	Zfp451	0.042807626	0.474142449	2.44E-18	1.51E-17
Corneal stromal keratocytes	Abca1	0.043321689	0.087442941	9.05E-10	2.88E-09
Corneal stromal keratocytes	Khdrbs1	0.043488283	0.375168521	7.93E-14	3.49E-13
Corneal stromal keratocytes	Svil	0.043660112	0.320367183	2.78E-10	9.27E-10
Corneal stromal keratocytes	Icam1	0.043704484	0.459319985	4.85E-05	9.66E-05
Corneal stromal keratocytes	Zfp9	0.043773333	0.11511892	8.95E-10	2.85E-09
Corneal stromal keratocytes	Ccnb1ip1	0.044336012	0.04080556	2.11E-11	7.70E-11
Corneal stromal keratocytes	5430416N02Rik	0.044790149	0.186932276	1.33E-10	4.56E-10
Corneal stromal keratocytes	Atf1	0.045051586	0.32816811	1.48E-11	5.44E-11
Corneal stromal keratocytes	Fzd1	0.045184699	0.173249302	1.01E-09	3.21E-09
Corneal stromal keratocytes	Plaur	0.045225431	0.25835001	1.69E-10	5.74E-10
Corneal stromal keratocytes	Khsrp	0.045236931	0.119920637	9.33E-10	2.97E-09
Corneal stromal keratocytes	Zfand2a	0.045420545	0.169198156	6.97E-08	1.87E-07
Corneal stromal keratocytes	Ifitm1	0.04546422	0.177417223	5.71E-07	1.40E-06
Corneal stromal keratocytes	Pcgf2	0.045648912	0.0591013	5.45E-17	3.08E-16
Corneal stromal keratocytes	Rgs5	0.045858887	0.040578654	1.09E-08	3.17E-08
Corneal stromal keratocytes	Pdlim5	0.045960108	0.303663754	6.52E-13	2.67E-12
Corneal stromal keratocytes	Wee1	0.046183082	0.256927525	1.55E-08	4.43E-08
Corneal stromal keratocytes	Paxbp1	0.046452565	0.183402318	3.22E-12	1.25E-11
Corneal stromal keratocytes	Zfp384	0.046586624	0.068463854	1.34E-16	7.30E-16
Corneal stromal keratocytes	Ccl7	0.04674794	0.250701142	0.0018852	0.003107146
Corneal stromal keratocytes	Aida	0.046841893	0.290147941	2.46E-13	1.05E-12
Corneal stromal keratocytes	Sox9	0.04688385	0.158487785	6.51E-09	1.94E-08
Corneal stromal keratocytes	Tgfb2	0.046940607	0.254608825	7.02E-13	2.88E-12
Corneal stromal keratocytes	Zeb2	0.047196508	0.361785001	3.12E-14	1.43E-13
Corneal stromal keratocytes	Mzt2	0.047251766	0.224114204	8.88E-08	2.37E-07
Corneal stromal keratocytes	Bdp1	0.047670975	0.269215201	1.06E-11	3.95E-11
Corneal stromal keratocytes	Nrp2	0.047756776	0.617433937	6.33E-16	3.29E-15
Corneal stromal keratocytes	Rtf1	0.04801065	0.364311266	2.37E-17	1.37E-16
Corneal stromal keratocytes	Gfod2	0.048056876	0.111253193	1.05E-14	4.95E-14
Corneal stromal keratocytes	Kctd12	0.048507185	0.359998177	1.08E-06	2.57E-06

Corneal stromal keratocytes	Fam102b	0.048708867	0.187283503	3.61E-12	1.40E-11
Corneal stromal keratocytes	Ctnnd1	0.048771582	0.16849639	1.76E-08	5.02E-08
Corneal stromal keratocytes	Isca1	0.048996972	0.131441998	1.13E-12	4.54E-12
Corneal stromal keratocytes	Pi16	0.049347701	0.063437628	1.46E-07	3.81E-07
Corneal stromal keratocytes	Cxcl10	0.049542532	0.081150784	1.86E-05	3.89E-05
Corneal stromal keratocytes	Itpkb	0.049623836	0.415517277	1.41E-11	5.22E-11
Corneal stromal keratocytes	Zc3hav1	0.049642341	0.318390127	1.27E-12	5.08E-12
Corneal stromal keratocytes	Ralbp1	0.050069561	0.325784534	4.06E-16	2.14E-15
Corneal stromal keratocytes	Homer1	0.050411827	0.366730158	4.96E-13	2.05E-12
Corneal stromal keratocytes	Trrap	0.050516544	0.154435135	6.62E-12	2.51E-11
Corneal stromal keratocytes	Polr2a	0.051184423	0.228707505	1.25E-11	4.63E-11
Corneal stromal keratocytes	Abi1	0.05127872	0.172224667	7.89E-12	2.97E-11
Corneal stromal keratocytes	Cd200	0.051410123	0.070818918	2.04E-10	6.87E-10
Corneal stromal keratocytes	Fosl2	0.051593134	0.51307468	1.32E-08	3.82E-08
Corneal stromal keratocytes	Lats1	0.051796065	0.248939691	2.03E-11	7.42E-11
Corneal stromal keratocytes	Atp13a3	0.05209668	0.22514505	2.31E-13	9.86E-13
Corneal stromal keratocytes	Sh2d5	0.05278914	0.121141149	1.13E-09	3.58E-09
Corneal stromal keratocytes	Azin1	0.052824237	0.321821807	4.53E-13	1.88E-12
Corneal stromal keratocytes	Tgm2	0.05328032	0.327088569	1.65E-16	8.93E-16
Corneal stromal keratocytes	Jmjd1c	0.053336394	0.44572278	2.36E-19	1.57E-18
Corneal stromal keratocytes	Hnrnpl	0.05356446	0.27840615	4.75E-11	1.69E-10
Corneal stromal keratocytes	Arl5b	0.053572166	0.218299432	1.89E-12	7.48E-12
Corneal stromal keratocytes	Pcolce2	0.053872743	0.179078959	3.68E-05	7.42E-05
Corneal stromal keratocytes	Reck	0.054117006	0.074246221	3.71E-17	2.12E-16
Corneal stromal keratocytes	Wsb1	0.054891782	0.213627289	4.66E-11	1.66E-10
Corneal stromal keratocytes	Uap1	0.055305754	0.68670627	9.82E-30	1.26E-28
Corneal stromal keratocytes	Macf1	0.055701047	0.587429332	2.79E-19	1.85E-18
Corneal stromal keratocytes	Edem3	0.056243705	0.108698666	1.60E-15	8.05E-15
Corneal stromal keratocytes	Dnajb4	0.056396504	0.203581955	5.81E-15	2.81E-14
Corneal stromal keratocytes	Nek7	0.056767475	0.186675151	3.11E-12	1.21E-11
Corneal stromal keratocytes	Foxp1	0.056945624	0.378460416	1.33E-13	5.76E-13
Corneal stromal keratocytes	Runx1	0.057770538	0.158250443	2.19E-10	7.36E-10
Corneal stromal keratocytes	Hspb1	0.057830148	0.866575888	8.47E-10	2.71E-09
Corneal stromal keratocytes	Nfkbiz	0.058412306	0.284088585	1.78E-11	6.53E-11
Corneal stromal keratocytes	Dpysl2	0.058840286	0.260974303	1.29E-13	5.60E-13
Corneal stromal keratocytes	Pbx1	0.059506265	0.199824921	2.49E-16	1.33E-15
Corneal stromal keratocytes	Akap12	0.059613147	0.11487	9.67E-09	2.83E-08
Corneal stromal keratocytes	Ptgs1	0.059732516	0.133910561	7.05E-11	2.48E-10
Corneal stromal keratocytes	Agl	0.060073109	0.068280803	3.08E-27	3.43E-26
Corneal stromal keratocytes	R3hdm2	0.060874358	0.325920735	1.58E-15	7.95E-15
Corneal stromal keratocytes	Dnaja1	0.061696994	1.488658758	1.76E-29	2.23E-28
Corneal stromal keratocytes	Huwe1	0.062279005	0.375729414	9.46E-15	4.49E-14
Corneal stromal keratocytes	Efhd2	0.062387779	0.297659647	1.22E-06	2.89E-06
Corneal stromal keratocytes	Utrn	0.062482695	0.325998029	2.32E-14	1.07E-13
Corneal stromal keratocytes	Cnot6	0.062585583	0.270499531	2.88E-16	1.54E-15
Corneal stromal keratocytes	Nr1d2	0.063032517	0.307174173	2.06E-13	8.84E-13
Corneal stromal keratocytes	Phactr4	0.06349711	0.16748642	1.41E-13	6.11E-13
Corneal stromal keratocytes	Tra2a	0.063525672	0.37425831	4.39E-13	1.83E-12
Corneal stromal keratocytes	Fbln1	0.063648377	0.11088208	4.61E-13	1.91E-12
Corneal stromal keratocytes	Jak2	0.063839665	0.247875716	6.35E-12	2.42E-11
Corneal stromal keratocytes	Fam107b	0.064013916	0.399486542	1.55E-17	9.10E-17
Corneal stromal keratocytes	Mef2a	0.064473197	0.571796353	1.64E-19	1.10E-18
Corneal stromal keratocytes	Irgm1	0.064592637	0.328356952	6.94E-12	2.63E-11
Corneal stromal keratocytes	Pde4d	0.065605508	0.200218578	3.01E-15	1.49E-14
Corneal stromal keratocytes	Fst	0.065767176	0.119285767	5.74E-10	1.86E-09
Corneal stromal keratocytes	Ccl2	0.066083326	1.048709725	0.0114067	0.016886848
Corneal stromal keratocytes	Csrnp1	0.066233731	0.286023915	3.30E-11	1.19E-10
Corneal stromal keratocytes	Postn	0.066684875	0.37622284	3.14E-12	1.22E-11
Corneal stromal keratocytes	Ensa	0.066810909	0.237053339	2.95E-12	1.15E-11
Corneal stromal keratocytes	Plpp3	0.066901733	1.3502476	6.42E-15	3.09E-14
Corneal stromal keratocytes	Clec2d	0.067467442	0.270352776	3.13E-10	1.04E-09
Corneal stromal keratocytes	Lrp6	0.067983005	0.251639621	1.28E-14	6.01E-14
Corneal stromal keratocytes	Tob2	0.068115207	0.162183794	4.64E-14	2.09E-13

Corneal stromal keratocytes	Map9	0.068205548	0.304779348	2.86E-13	1.21E-12
Corneal stromal keratocytes	Serpina3n	0.068261188	0.159782924	1.63E-13	7.05E-13
Corneal stromal keratocytes	Chil1	0.068960119	0.277702186	1.07E-05	2.30E-05
Corneal stromal keratocytes	Hist1h4d	0.069416374	0.122132917	1.31E-18	8.29E-18
Corneal stromal keratocytes	Klhl21	0.070384257	0.256586636	7.78E-12	2.94E-11
Corneal stromal keratocytes	Kremen1	0.070494182	0.137639708	4.31E-18	2.63E-17
Corneal stromal keratocytes	Isy1	0.070735945	0.404074017	1.37E-19	9.24E-19
Corneal stromal keratocytes	Map2	0.070855178	0.08922453	5.96E-27	6.51E-26
Corneal stromal keratocytes	Elmsan1	0.071536959	0.13112807	8.67E-18	5.19E-17
Corneal stromal keratocytes	Lpin2	0.072530562	0.422199715	2.27E-16	1.22E-15
Corneal stromal keratocytes	Ets2	0.072586233	0.445777491	1.89E-16	1.02E-15
Corneal stromal keratocytes	Slc20a1	0.073325572	0.303583516	2.81E-13	1.19E-12
Corneal stromal keratocytes	Serpine2	0.07333471	0.152201473	2.56E-06	5.85E-06
Corneal stromal keratocytes	Slc7a8	0.074166779	0.125541958	3.59E-20	2.51E-19
Corneal stromal keratocytes	Dync1i2	0.074266355	0.415951033	2.44E-22	1.98E-21
Corneal stromal keratocytes	Mid1	0.076042868	0.511054053	5.55E-14	2.48E-13
Corneal stromal keratocytes	Cpeb4	0.077140287	0.418601587	1.25E-17	7.39E-17
Corneal stromal keratocytes	Ackr3	0.077915715	0.179022809	9.38E-16	4.82E-15
Corneal stromal keratocytes	Gclc	0.077956488	0.341304735	4.68E-13	1.94E-12
Corneal stromal keratocytes	Snapc1	0.078463248	0.346363403	1.86E-20	1.33E-19
Corneal stromal keratocytes	Tnc	0.07864386	0.490342161	1.52E-13	6.59E-13
Corneal stromal keratocytes	Hmgb2	0.078988456	0.157609339	2.75E-17	1.59E-16
Corneal stromal keratocytes	Btg2	0.079028501	0.463008814	3.26E-08	9.03E-08
Corneal stromal keratocytes	Cebpd	0.07943077	0.757196486	0.0014392	0.002403386
Corneal stromal keratocytes	Smg1	0.079555346	0.369060327	7.51E-20	5.15E-19
Corneal stromal keratocytes	Il6	0.081067417	0.064687751	1.27E-24	1.21E-23
Corneal stromal keratocytes	Nr4a1	0.081648286	0.237275522	8.18E-09	2.41E-08
Corneal stromal keratocytes	Fgl2	0.082158618	0.2721936	4.85E-06	1.07E-05
Corneal stromal keratocytes	MT-COX3	0.08325294	2.975210563	1.26E-08	3.65E-08
Corneal stromal keratocytes	Picalm	0.084228209	0.43336016	4.74E-22	3.80E-21
Corneal stromal keratocytes	Kitl	0.084554344	0.238112503	5.21E-13	2.15E-12
Corneal stromal keratocytes	Igfbp6	0.084704043	0.190154576	5.26E-16	2.75E-15
Corneal stromal keratocytes	Fam129b	0.085690245	0.417591691	7.60E-24	6.84E-23
Corneal stromal keratocytes	Nampt	0.087827071	0.470429561	2.79E-22	2.27E-21
Corneal stromal keratocytes	MT-ND4	0.087984838	2.687459218	1.69E-07	4.39E-07
Corneal stromal keratocytes	Kpna1	0.088437667	0.322072678	3.92E-19	2.57E-18
Corneal stromal keratocytes	Tnfaip3	0.089253792	0.12681953	2.94E-23	2.56E-22
Corneal stromal keratocytes	2410006H16Rik	0.089307661	1.01233704	4.11E-27	4.55E-26
Corneal stromal keratocytes	Jup	0.089937829	0.194283969	2.02E-22	1.66E-21
Corneal stromal keratocytes	Parp1	0.092143688	0.165147966	4.45E-18	2.71E-17
Corneal stromal keratocytes	Ndrg1	0.093085575	0.172234518	3.07E-18	1.89E-17
Corneal stromal keratocytes	Rps6ka3	0.093262456	0.342990204	1.19E-21	9.32E-21
Corneal stromal keratocytes	Parm1	0.094040531	0.236838735	6.41E-23	5.47E-22
Corneal stromal keratocytes	Btg1	0.094057458	0.853533931	1.56E-19	1.05E-18
Corneal stromal keratocytes	Pla2g5	0.094325387	0.242864464	5.03E-24	4.62E-23
Corneal stromal keratocytes	Slc30a5	0.095308969	0.214370566	5.46E-22	4.36E-21
Corneal stromal keratocytes	Bag3	0.096423035	0.973427668	1.97E-30	2.65E-29
Corneal stromal keratocytes	Mapk6	0.098236431	0.793875006	1.84E-24	1.74E-23
Corneal stromal keratocytes	Rybp	0.098409233	0.34279295	6.11E-24	5.57E-23
Corneal stromal keratocytes	B4galt5	0.098560632	0.217143913	1.57E-21	1.22E-20
Corneal stromal keratocytes	Ank	0.099720223	1.346067913	3.12E-17	1.80E-16
Corneal stromal keratocytes	Acsl3	0.100744148	0.681172245	3.43E-29	4.27E-28
Corneal stromal keratocytes	Slc5a3	0.100890173	0.223340286	8.84E-22	6.96E-21
Corneal stromal keratocytes	Gpc3	0.100960013	0.186176362	2.59E-21	1.98E-20
Corneal stromal keratocytes	Ppp1r15a	0.101454664	0.545083768	2.35E-14	1.08E-13
Corneal stromal keratocytes	Rell1	0.101571132	0.273062382	2.65E-20	1.88E-19
Corneal stromal keratocytes	Osmr	0.101869903	0.447749672	9.52E-20	6.49E-19
Corneal stromal keratocytes	Trip10	0.102984065	0.300069141	5.20E-19	3.38E-18
Corneal stromal keratocytes	Dab2	0.10347546	0.878708507	4.58E-21	3.43E-20
Corneal stromal keratocytes	Arl4d	0.104005172	0.706140909	2.32E-17	1.35E-16
Corneal stromal keratocytes	Crispld2	0.10416239	0.516135096	2.73E-20	1.93E-19
Corneal stromal keratocytes	Ptgis	0.104305391	0.439060805	9.66E-13	3.91E-12
Corneal stromal keratocytes	Csf1	0.105328572	0.351437964	1.52E-13	6.57E-13

Corneal stromal keratocytes	Gem	0.105400393	0.539913417	5.20E-07	1.28E-06
Corneal stromal keratocytes	Insig1	0.106216744	0.994426713	1.72E-26	1.82E-25
Corneal stromal keratocytes	Arih1	0.106724062	0.452869283	3.73E-31	5.28E-30
Corneal stromal keratocytes	Fosb	0.106916561	0.837091499	2.30E-05	4.76E-05
Corneal stromal keratocytes	1810037I17Rik	0.107124185	0.628210524	6.46E-36	1.18E-34
Corneal stromal keratocytes	Col4a5	0.107551167	0.428381557	4.98E-17	2.82E-16
Corneal stromal keratocytes	Zfp948	0.109085486	0.224016932	8.27E-24	7.43E-23
Corneal stromal keratocytes	S100a4	0.109752737	0.476521752	1.64E-12	6.54E-12
Corneal stromal keratocytes	Cflar	0.110429637	0.340704053	8.81E-26	8.90E-25
Corneal stromal keratocytes	Cxcl2	0.111384505	0.13302404	8.87E-28	1.02E-26
Corneal stromal keratocytes	Abi3bp	0.111813923	0.791556289	3.79E-19	2.48E-18
Corneal stromal keratocytes	Pdcd4	0.112175371	0.423666996	3.29E-26	3.41E-25
Corneal stromal keratocytes	Ugcg	0.113097423	0.467741419	4.02E-21	3.03E-20
Corneal stromal keratocytes	Fgf2	0.113906366	0.391197617	9.14E-21	6.72E-20
Corneal stromal keratocytes	Zfp36l2	0.114648546	0.277286507	8.26E-17	4.58E-16
Corneal stromal keratocytes	Sdc4	0.115401823	2.362049337	1.92E-27	2.16E-26
Corneal stromal keratocytes	Lmo4	0.116381551	0.461097705	7.41E-27	8.04E-26
Corneal stromal keratocytes	Akap2	0.117415837	1.20543591	3.80E-26	3.92E-25
Corneal stromal keratocytes	Tpbg	0.117761926	0.528264164	1.27E-16	6.96E-16
Corneal stromal keratocytes	Loxl2	0.119646519	0.362145407	1.27E-25	1.27E-24
Corneal stromal keratocytes	Ldlr	0.12111047	0.702412377	3.44E-45	9.76E-44
Corneal stromal keratocytes	Ltbp4	0.121725365	0.386097419	3.63E-30	4.82E-29
Corneal stromal keratocytes	Scd1	0.12192087	0.26418937	1.69E-21	1.30E-20
Corneal stromal keratocytes	Erdr1	0.122248462	1.409574894	5.58E-31	7.81E-30
Corneal stromal keratocytes	Tm4sf1	0.122405605	0.703842943	7.38E-05	0.000143813
Corneal stromal keratocytes	Ier5	0.124553232	0.75496939	1.38E-24	1.31E-23
Corneal stromal keratocytes	Tnfaip2	0.125661934	0.410091743	4.69E-27	5.17E-26
Corneal stromal keratocytes	Lipg	0.12619922	0.129021673	1.83E-37	3.65E-36
Corneal stromal keratocytes	Rps27rt	0.127011121	0.19349229	9.64E-45	2.70E-43
Corneal stromal keratocytes	Hspe1	0.127064603	1.191707042	2.18E-51	7.88E-50
Corneal stromal keratocytes	Arrdc3	0.128829285	0.663434237	1.04E-26	1.12E-25
Corneal stromal keratocytes	Polr2l	0.130878779	0.575204937	1.53E-39	3.38E-38
Corneal stromal keratocytes	Ezr	0.133150955	0.851078412	4.40E-25	4.29E-24
Corneal stromal keratocytes	Slc2a1	0.134905204	1.145006248	6.26E-24	5.68E-23
Corneal stromal keratocytes	Mbnl1	0.135123762	0.465720834	6.81E-37	1.33E-35
Corneal stromal keratocytes	Sat1	0.135482097	1.560321663	6.23E-09	1.86E-08
Corneal stromal keratocytes	Pfkfb3	0.135984235	0.777403611	1.60E-30	2.16E-29
Corneal stromal keratocytes	Susd6	0.136756804	0.332928714	5.12E-34	8.64E-33
Corneal stromal keratocytes	Zfp36	0.138685733	0.231801928	2.63E-24	2.45E-23
Corneal stromal keratocytes	Cyp2f2	0.138694916	1.712151643	3.34E-44	9.11E-43
Corneal stromal keratocytes	F2r	0.139986281	0.381158984	1.21E-33	2.00E-32
Corneal stromal keratocytes	Ankrd12	0.140360688	0.504585057	1.14E-32	1.76E-31
Corneal stromal keratocytes	Timp3	0.141586166	0.391739208	2.42E-18	1.50E-17
Corneal stromal keratocytes	Plat	0.142906307	0.553634618	2.10E-13	8.98E-13
Corneal stromal keratocytes	Hspa5	0.145092301	2.638886525	1.60E-18	1.00E-17
Corneal stromal keratocytes	Igfbp3	0.145730774	0.518347281	3.93E-15	1.92E-14
Corneal stromal keratocytes	Idi1	0.146047142	0.891120755	2.73E-44	7.46E-43
Corneal stromal keratocytes	Cavin1	0.146197033	0.422213404	4.29E-38	8.86E-37
Corneal stromal keratocytes	Scd2	0.146242026	0.865924716	1.73E-33	2.83E-32
Corneal stromal keratocytes	Ptges	0.14754627	0.826416848	3.79E-24	3.52E-23
Corneal stromal keratocytes	Zfp36l1	0.157031925	0.448903291	3.35E-22	2.71E-21
Corneal stromal keratocytes	Twsg1	0.157476154	0.348263681	6.12E-49	1.99E-47
Corneal stromal keratocytes	Smad7	0.159041938	0.427069745	1.55E-33	2.54E-32
Corneal stromal keratocytes	Spry2	0.16497384	0.393239939	3.32E-31	4.71E-30
Corneal stromal keratocytes	Lif	0.17294645	0.430526036	1.85E-22	1.52E-21
Corneal stromal keratocytes	Nup98	0.173245046	0.360742877	2.49E-46	7.36E-45
Corneal stromal keratocytes	Gsn	0.175020993	0.974143689	6.64E-34	1.11E-32
Corneal stromal keratocytes	Bhlhe41	0.177526982	0.612742107	5.80E-36	1.07E-34
Corneal stromal keratocytes	Aff1	0.178307307	0.406467048	2.68E-49	8.87E-48
Corneal stromal keratocytes	Nfkb1	0.178655724	0.737212799	1.33E-43	3.55E-42
Corneal stromal keratocytes	Adamts1	0.178687371	0.568301565	6.73E-21	4.98E-20
Corneal stromal keratocytes	MT-ND5	0.185990224	1.240906873	1.44E-45	4.14E-44
Corneal stromal keratocytes	Hbegf	0.187015205	1.693957614	5.08E-12	1.95E-11

Corneal stromal keratocytes	MT-ND3	0.187713732	1.101322368	1.21E-46	3.63E-45
Corneal stromal keratocytes	Igfbp5	0.190796481	1.316003975	5.64E-30	7.41E-29
Corneal stromal keratocytes	Cebpb	0.191678509	1.560216384	1.53E-26	1.62E-25
Corneal stromal keratocytes	Dst	0.197009163	1.348911441	6.73E-52	2.49E-50
Corneal stromal keratocytes	Gfpt2	0.198472935	0.694181421	5.41E-35	9.54E-34
Corneal stromal keratocytes	Pam	0.20106823	1.017335631	2.91E-48	9.27E-47
Corneal stromal keratocytes	Has1	0.203477528	0.21894416	1.57E-61	8.47E-60
Corneal stromal keratocytes	Nr4a2	0.208747128	0.832061577	2.02E-32	3.07E-31
Corneal stromal keratocytes	Ctgf	0.218196678	0.406685952	3.31E-23	2.86E-22
Corneal stromal keratocytes	Pde4b	0.218754284	0.679455134	2.22E-33	3.60E-32
Corneal stromal keratocytes	Cemip	0.222274305	0.422569192	9.35E-52	3.43E-50
Corneal stromal keratocytes	Hsph1	0.222649844	1.046445462	1.01E-43	2.71E-42
Corneal stromal keratocytes	Gpha2	0.223708808	0.846364168	1.87E-18	1.17E-17
Corneal stromal keratocytes	Ero1l	0.224414013	0.837291977	3.26E-32	4.91E-31
Corneal stromal keratocytes	Cxcl1	0.236843475	0.357726963	1.08E-32	1.67E-31
Corneal stromal keratocytes	Odc1	0.237125199	2.625863257	2.57E-38	5.38E-37
Corneal stromal keratocytes	MT-ND1	0.248166904	1.93229606	2.72E-65	1.63E-63
Corneal stromal keratocytes	Klf6	0.250488492	1.007518874	3.09E-55	1.29E-53
Corneal stromal keratocytes	Junb	0.255298911	0.986305912	1.20E-18	7.59E-18
Corneal stromal keratocytes	Glul	0.256681506	0.644136403	1.26E-51	4.59E-50
Corneal stromal keratocytes	Nr4a3	0.256785305	0.434341732	2.30E-53	8.81E-52
Corneal stromal keratocytes	Nfkbia	0.258649007	1.041362823	3.42E-38	7.09E-37
Corneal stromal keratocytes	Ugdh	0.26429164	0.818339984	2.64E-47	8.16E-46
Corneal stromal keratocytes	Cyr61	0.265863466	0.69407944	7.94E-34	1.32E-32
Corneal stromal keratocytes	F3	0.266059537	0.66224988	7.54E-50	2.56E-48
Corneal stromal keratocytes	MT-ATP8	0.266564266	0.908344014	5.93E-59	2.80E-57
Corneal stromal keratocytes	Ywhaz	0.282198834	1.050359192	4.09E-112	6.17E-110
Corneal stromal keratocytes	Hspa1b	0.285005329	0.556163645	9.12E-22	7.17E-21
Corneal stromal keratocytes	MT-ND4L	0.287047811	1.696495636	1.10E-63	6.25E-62
Corneal stromal keratocytes	Maff	0.293646591	0.967278061	1.26E-39	2.81E-38
Corneal stromal keratocytes	Hba-a1	0.29525741	0.384073925	8.37E-27	9.02E-26
Corneal stromal keratocytes	Hspa1a	0.311043828	0.618664234	9.71E-21	7.12E-20
Corneal stromal keratocytes	Nr1d1	0.314094725	0.630224169	7.08E-94	8.35E-92
Corneal stromal keratocytes	Bhlhe40	0.317522963	0.619663108	3.28E-68	2.18E-66
Corneal stromal keratocytes	Hbb-b1	0.323367449	0.401120001	9.28E-28	1.06E-26
Corneal stromal keratocytes	Ptgs2	0.373522782	2.096927884	8.51E-61	4.46E-59
Corneal stromal keratocytes	Xist	0.423028521	0.555325112	1.08E-132	2.17E-130
Corneal stromal keratocytes	Emp1	0.427517039	2.803598176	9.62E-141	2.21E-138
Corneal stromal keratocytes	Vegfa	0.455876165	1.580795191	5.28E-155	1.48E-152
Corneal stromal keratocytes	Hk2	0.53598668	1.613197499	1.53E-140	3.47E-138
Corneal stromal keratocytes	Lars2	0.541957591	1.032277085	7.00E-175	2.46E-172
Corneal stromal keratocytes	Mt2	0.557617104	2.484528012	2.32E-71	1.68E-69
Corneal stromal keratocytes	Mt1	0.574030757	3.123149807	4.75E-47	1.45E-45
Corneal stromal keratocytes	Thbs1	0.624527511	2.898408835	1.31E-125	2.35E-123
Corneal stromal keratocytes	S100a6	0.632334182	3.368421238	2.22E-253	1.56E-250
Corneal stromal keratocytes	Serpine1	0.639342233	1.301285835	7.44E-144	1.83E-141
SC and lymphatic ECs	Jun	-1.14260149	2.658120092	0.0007395	0.186373698
SC and lymphatic ECs	Hspa1b	-1.121403368	2.939403139	0.0076088	0.561297423
SC and lymphatic ECs	Junb	-0.98362669	2.644956271	0.0001452	0.069384066
SC and lymphatic ECs	Hspb1	-0.955979661	2.560247897	0.0097413	0.627877173
SC and lymphatic ECs	Jund	-0.942106736	2.630965941	3.63E-05	0.042418181
SC and lymphatic ECs	Ifitm2	-0.904783249	1.826575655	0.0002874	0.109056119
SC and lymphatic ECs	Fos	-0.834401129	1.172832311	0.0132296	0.684052662
SC and lymphatic ECs	Dusp1	-0.813816482	1.266964013	0.001851	0.30935919
SC and lymphatic ECs	Fxyd6	-0.793469639	2.234127002	0.0073266	0.561297423
SC and lymphatic ECs	Phlda1	-0.779454454	0.974660754	0.0061333	0.515598347
SC and lymphatic ECs	Cldn5	-0.745865685	2.732608458	0.0156683	0.70277205
SC and lymphatic ECs	Rgs16	-0.7195119	1.497692344	0.0115838	0.653110096
SC and lymphatic ECs	Ramp2	-0.712845559	1.36088075	0.0045076	0.439461102
SC and lymphatic ECs	Gadd45g	-0.704180422	1.556613468	0.0383467	0.886901138
SC and lymphatic ECs	Apold1	-0.695396002	1.205306128	0.01393	0.684052662
SC and lymphatic ECs	Socs3	-0.628910827	0.934228234	0.0200219	0.739084634
SC and lymphatic ECs	Cavin3	-0.603332571	2.408889034	0.0128621	0.684052662

SC and lymphatic ECs	Ftl1	-0.584998923	3.214914763	0.0029094	0.378201169
SC and lymphatic ECs	Ier2	-0.571919013	1.079496083	0.0285776	0.807244846
SC and lymphatic ECs	Cdc42ep2	-0.510900071	0.591524906	0.0035767	0.405541196
SC and lymphatic ECs	Rhob	-0.506802514	1.847897528	0.0432424	0.945029406
SC and lymphatic ECs	Hes1	-0.504731147	1.090413179	0.0314162	0.846613344
SC and lymphatic ECs	Gm13889	-0.503300409	0.63397966	0.0421312	0.941846108
SC and lymphatic ECs	Impdh2	-0.497632693	0.702742728	0.0037321	0.406157572
SC and lymphatic ECs	Calm1	-0.453186567	3.071434207	0.0228547	0.771900508
SC and lymphatic ECs	Mrps6	-0.45054674	0.524998314	0.0063322	0.527624929
SC and lymphatic ECs	Bambi	-0.420098408	0.516455638	0.0182862	0.739084634
SC and lymphatic ECs	Palm	-0.394815131	0.481409086	0.0124614	0.672869952
SC and lymphatic ECs	Hmgn2	-0.393511181	0.562748064	0.012641	0.679951656
SC and lymphatic ECs	Crlf2	-0.383768725	0.628794124	0.0368323	0.871776615
SC and lymphatic ECs	Gtf2b	-0.381557697	0.493009884	0.0068018	0.542557216
SC and lymphatic ECs	Serpine2	-0.311769527	0.385869318	0.046291	0.952161119
SC and lymphatic ECs	Mustn1	-0.258172876	0.272629593	0.0038704	0.417976073
SC and lymphatic ECs	Tmem51	-0.252155593	0.225258646	0.0167093	0.723178887
SC and lymphatic ECs	Ctsh	-0.243251683	0.222350278	0.0277104	0.797865466
SC and lymphatic ECs	Rcn3	-0.242351519	0.467599543	0.0148507	0.684052662
SC and lymphatic ECs	Ggh	-0.229199145	0.281992944	0.0171192	0.723751107
SC and lymphatic ECs	Cdkn1c	-0.228964405	0.201893813	0.0161065	0.712631446
SC and lymphatic ECs	Pmm2	-0.183725911	0.144599096	0.0023406	0.352782284
SC and lymphatic ECs	Dnpep	-0.180385086	0.141969744	0.0017008	0.29478206
SC and lymphatic ECs	Prkca	-0.176234564	0.138703129	0.0032112	0.393423375
SC and lymphatic ECs	Timm50	-0.174668342	0.137470454	0.0043929	0.438749309
SC and lymphatic ECs	Hsd17b11	-0.167982023	0.132208073	0.0032112	0.393423375
SC and lymphatic ECs	Dyrk3	-0.162913911	0.128219282	0.0110747	0.629464566
SC and lymphatic ECs	Ccdc77	-0.161946412	0.127457825	0.0059932	0.506859548
SC and lymphatic ECs	Cenpa	-0.159942856	0.125880952	0.0150074	0.684052662
SC and lymphatic ECs	Slc50a1	-0.151949584	0.119589951	0.0059932	0.506859548
SC and lymphatic ECs	Nit2	-0.151287406	0.119068792	0.0059932	0.506859548
SC and lymphatic ECs	Foxred1	-0.149892203	0.117970715	0.0059932	0.506859548
SC and lymphatic ECs	5930412G12Rik	-0.148180681	0.116623684	0.0081562	0.561297423
SC and lymphatic ECs	Rprd1a	-0.147122062	0.115790512	0.0081562	0.561297423
SC and lymphatic ECs	Casp6	-0.141400457	0.111287396	0.0059932	0.506859548
SC and lymphatic ECs	Zbtb24	-0.136048918	0.107075538	0.0081562	0.561297423
SC and lymphatic ECs	Edn1	-0.135725749	0.106821192	0.0370097	0.871776615
SC and lymphatic ECs	Smpd2	-0.132525836	0.104302742	0.0274246	0.7938445
SC and lymphatic ECs	Sugp2	-0.126270839	0.099379827	0.0203014	0.739084634
SC and lymphatic ECs	Nubpl	-0.124933394	0.098327208	0.0274246	0.7938445
SC and lymphatic ECs	Htra1	-0.124053789	0.097634927	0.0274246	0.7938445
SC and lymphatic ECs	Hsf2	-0.122913949	0.096737831	0.0150074	0.684052662
SC and lymphatic ECs	Fam174b	-0.120386302	0.094748479	0.0150074	0.684052662
SC and lymphatic ECs	Zfp580	-0.118375887	0.093166207	0.0150074	0.684052662
SC and lymphatic ECs	Cog5	-0.118358051	0.09315217	0.0150074	0.684052662
SC and lymphatic ECs	Armc8	-0.117744492	0.092669276	0.0110747	0.629464566
SC and lymphatic ECs	Tpx2	-0.115188365	0.09065751	0.0499174	0.952161119
SC and lymphatic ECs	Npl	-0.114847116	0.090388934	0.0274246	0.7938445
SC and lymphatic ECs	Fabp4	-0.114740216	0.090304799	0.0499174	0.952161119
SC and lymphatic ECs	Med24	-0.114342775	0.089991999	0.0110747	0.629464566
SC and lymphatic ECs	Bcat2	-0.111963834	0.088119684	0.0110747	0.629464566
SC and lymphatic ECs	Vkorc1l1	-0.110993697	0.142517152	0.0277909	0.797865466
SC and lymphatic ECs	Abcd3	-0.109797204	0.086414466	0.0370097	0.871776615
SC and lymphatic ECs	Gdpd1	-0.10689897	0.084133448	0.0110747	0.629464566
SC and lymphatic ECs	Vps52	-0.106057388	0.083471092	0.0203014	0.739084634
SC and lymphatic ECs	Sorbs3	-0.105212407	0.082806061	0.0203014	0.739084634
SC and lymphatic ECs	Alad	-0.103518237	0.081472686	0.0499174	0.952161119
SC and lymphatic ECs	Rida	-0.103513998	0.08146935	0.0370097	0.871776615
SC and lymphatic ECs	Alkbh6	-0.102511985	0.080680729	0.0150074	0.684052662
SC and lymphatic ECs	Wdr6	-0.099926542	0.07864589	0.0150074	0.684052662
SC and lymphatic ECs	Mthfd2l	-0.097006355	0.076347594	0.0274246	0.7938445
SC and lymphatic ECs	Dlat	-0.096878724	0.076247144	0.0370097	0.871776615
SC and lymphatic ECs	Fxn	-0.096424154	0.075889381	0.0274246	0.7938445

SC and lymphatic ECs	Zkscan14	-0.093570942	0.073643797	0.0203014	0.739084634
SC and lymphatic ECs	Spryd3	-0.093274256	0.073410294	0.0499174	0.952161119
SC and lymphatic ECs	Fbxo33	-0.092816851	0.073050299	0.0370097	0.871776615
SC and lymphatic ECs	Tyw5	-0.091851832	0.072290793	0.0499174	0.952161119
SC and lymphatic ECs	Ccdc157	-0.091423436	0.07195363	0.0499174	0.952161119
SC and lymphatic ECs	Cpped1	-0.089899792	0.070754466	0.0274246	0.7938445
SC and lymphatic ECs	Atl1	-0.089783644	0.070663053	0.0274246	0.7938445
SC and lymphatic ECs	Amz2	-0.088111483	0.069347001	0.0499174	0.952161119
SC and lymphatic ECs	44441	-0.086241354	0.06787514	0.0370097	0.871776615
SC and lymphatic ECs	Mtg1	-0.085893605	0.067601448	0.0499174	0.952161119
SC and lymphatic ECs	Nab1	-0.082703931	0.261403721	0.0182231	0.739084634
SC and lymphatic ECs	Ccl19	-0.081181762	0.063893053	0.0499174	0.952161119
SC and lymphatic ECs	Decr1	-0.078822958	0.062036588	0.0499174	0.952161119
SC and lymphatic ECs	D930016D06Rik	-0.078389979	0.061695817	0.0370097	0.871776615
SC and lymphatic ECs	Aldh3a2	-0.07552479	0.059440807	0.0274246	0.7938445
SC and lymphatic ECs	B9d1	-0.07532443	0.059283116	0.0499174	0.952161119
SC and lymphatic ECs	Trmt61b	-0.074838454	0.058900635	0.0499174	0.952161119
SC and lymphatic ECs	Snapc2	-0.074504377	0.058637704	0.0499174	0.952161119
SC and lymphatic ECs	Epm2aip1	-0.073470725	0.133983389	0.0246629	0.7938445
SC and lymphatic ECs	Uckl1	-0.073028518	0.057476148	0.0370097	0.871776615
SC and lymphatic ECs	Fam212a	-0.071886976	0.056577712	0.0499174	0.952161119
SC and lymphatic ECs	Wrn	-0.071183494	0.056024046	0.0499174	0.952161119
SC and lymphatic ECs	Crebzf	-0.070164028	0.133654125	0.0255357	0.7938445
SC and lymphatic ECs	Prkra	-0.03913051	0.159758382	0.0005412	0.165135669
SC and lymphatic ECs	Armcx2	-0.019935019	0.094122034	0.0148888	0.684052662
SC and lymphatic ECs	Vps11	-0.013373982	0.133934363	0.0341423	0.871776615
SC and lymphatic ECs	Oxct1	-0.012209213	0.24950872	0.0014316	0.264452377
SC and lymphatic ECs	Per1	0.012756735	0.094793993	0.0426329	0.942352393
SC and lymphatic ECs	Krt12	0.012836656	0.18710584	0.0098449	0.62823651
SC and lymphatic ECs	Traf1	0.013940243	0.152730083	0.0289233	0.80887286
SC and lymphatic ECs	Meis2	0.024454891	0.059185532	0.048719	0.952161119
SC and lymphatic ECs	Dopey2	0.040076983	0.125669349	0.0059064	0.506859548
SC and lymphatic ECs	Ankrd26	0.06068338	0.05538695	0.0280545	0.802087868
SC and lymphatic ECs	Gfpt1	0.060721401	0.046595199	0.0194499	0.739084634
SC and lymphatic ECs	Mgme1	0.06247249	0.09870225	0.041826	0.93651488
SC and lymphatic ECs	Ezr	0.064825478	0.077941169	0.0400851	0.912003494
SC and lymphatic ECs	Pdgfb	0.068198722	0.11491048	0.0221439	0.760093623
SC and lymphatic ECs	Zfp951	0.070277476	0.0149665	0.021216	0.739084634
SC and lymphatic ECs	Nxph4	0.08118694	0.017289811	0.021216	0.739084634
SC and lymphatic ECs	Tead3	0.08378558	0.017843225	0.021216	0.739084634
SC and lymphatic ECs	Pde4a	0.086800674	0.018485329	0.021216	0.739084634
SC and lymphatic ECs	Brca1	0.087738655	0.018685084	0.021216	0.739084634
SC and lymphatic ECs	Tchh	0.091128324	0.019406958	0.021216	0.739084634
SC and lymphatic ECs	Neurl1a	0.102157962	0.021755862	0.021216	0.739084634
SC and lymphatic ECs	Lbhd1	0.120100959	0.025577056	0.021216	0.739084634
SC and lymphatic ECs	Gins1	0.121205757	0.025812337	0.0035979	0.405541196
SC and lymphatic ECs	Tubb2a	0.14292241	0.364693999	0.0448072	0.952161119
SC and lymphatic ECs	Slc24a1	0.145078478	0.037049718	0.0154991	0.701908664
SC and lymphatic ECs	Cep135	0.147459079	0.036768903	0.0149822	0.684052662
SC and lymphatic ECs	Anks1	0.151813675	0.119524629	0.032158	0.858076846
SC and lymphatic ECs	Senp3	0.163205204	0.053075876	0.0253881	0.7938445
SC and lymphatic ECs	Zbtb38	0.168431756	0.378745419	0.0328937	0.8619954
SC and lymphatic ECs	Slc16a2	0.169697313	0.038189169	0.0411208	0.926636217
SC and lymphatic ECs	Crebl2	0.170778483	0.059149155	0.0083384	0.564148993
SC and lymphatic ECs	BC037034	0.176475109	0.037582662	0.000608	0.174204936
SC and lymphatic ECs	Tlr4	0.179058107	0.069931817	0.0308866	0.843613901
SC and lymphatic ECs	Mettl14	0.193630683	0.069593127	0.0435898	0.947301288
SC and lymphatic ECs	Zfp366	0.205830073	0.07919456	0.0272874	0.7938445
SC and lymphatic ECs	Insr	0.215853194	0.313706013	0.0261307	0.7938445
SC and lymphatic ECs	Usp18	0.223786252	0.353292426	0.0044313	0.438749309
SC and lymphatic ECs	Parp3	0.224455512	0.111298774	0.0379693	0.882534869
SC and lymphatic ECs	Mpp6	0.234169084	0.330396177	0.0319554	0.854517146
SC and lymphatic ECs	Gbe1	0.237452858	0.191578639	0.0215981	0.745246892

SC and lymphatic ECs	Lnx2	0.238102086	0.084810432	0.019638	0.739084634
SC and lymphatic ECs	Myo9b	0.239006794	0.108698187	0.0312844	0.846244724
SC and lymphatic ECs	Smarcc1	0.239154276	0.256373066	0.0453512	0.952161119
SC and lymphatic ECs	Klhl2	0.244514924	0.147513744	0.0317675	0.85111468
SC and lymphatic ECs	Col6a2	0.24661773	0.131679569	0.0359067	0.871776615
SC and lymphatic ECs	Otud7b	0.247250614	0.141055955	0.0431776	0.945029406
SC and lymphatic ECs	Mafk	0.250858442	0.628368134	0.0014901	0.271680217
SC and lymphatic ECs	Snhg6	0.251071243	0.086669119	0.0133701	0.684052662
SC and lymphatic ECs	Zfp598	0.251327214	0.286365273	0.0483448	0.952161119
SC and lymphatic ECs	Arhgap32	0.260939074	0.137538091	0.0163663	0.720271374
SC and lymphatic ECs	Gapvd1	0.264551495	0.332255877	0.0242572	0.7938445
SC and lymphatic ECs	Ripk2	0.266429794	0.187197559	0.0465058	0.952161119
SC and lymphatic ECs	Mrps30	0.271549941	0.145710392	0.0431314	0.945029406
SC and lymphatic ECs	Tmem181a	0.286498223	0.09476572	0.0084263	0.564148993
SC and lymphatic ECs	Smg1	0.28805343	0.546596034	0.0179986	0.739084634
SC and lymphatic ECs	Rai14	0.294599522	0.628121258	0.0110468	0.629464566
SC and lymphatic ECs	Khnyn	0.295205364	0.102885795	0.0020944	0.336994168
SC and lymphatic ECs	Msl2	0.300195873	0.152568577	0.0231956	0.773282627
SC and lymphatic ECs	Col6a1	0.302243386	0.11422754	0.0173106	0.725443431
SC and lymphatic ECs	Gbp7	0.311867603	0.952401336	0.0031615	0.393423375
SC and lymphatic ECs	Aida	0.313454999	0.33274874	0.0322593	0.858076846
SC and lymphatic ECs	Ivns1abp	0.315435619	0.58629764	0.0464672	0.952161119
SC and lymphatic ECs	Psmb9	0.321461949	0.163225165	0.01358	0.684052662
SC and lymphatic ECs	Sema7a	0.331930744	0.174175553	0.0196145	0.739084634
SC and lymphatic ECs	Hnrnpu	0.331983826	1.212572602	0.0007141	0.186373698
SC and lymphatic ECs	Arhgap6	0.337584755	0.343715056	0.0472492	0.952161119
SC and lymphatic ECs	Lum	0.337634366	0.125000632	0.0025538	0.364333702
SC and lymphatic ECs	Aars	0.342073144	0.297631299	0.0366359	0.871776615
SC and lymphatic ECs	Ankrd28	0.354964631	0.255392609	0.0160402	0.712620768
SC and lymphatic ECs	Snrpf	0.356456627	0.636013465	0.0249942	0.7938445
SC and lymphatic ECs	Nr1d1	0.357240448	0.445244524	0.0124191	0.672869952
SC and lymphatic ECs	Ccnyl1	0.370591236	0.232811818	0.037517	0.876374365
SC and lymphatic ECs	Ripk1	0.376308723	0.65327939	0.0223514	0.763483716
SC and lymphatic ECs	Igfbp2	0.377062665	0.23069637	0.0026577	0.37312029
SC and lymphatic ECs	Jak2	0.380451891	0.522804553	0.0352567	0.871776615
SC and lymphatic ECs	Gbp5	0.392147067	0.339001734	0.0388489	0.89275766
SC and lymphatic ECs	Cflar	0.395396531	0.616324019	0.047122	0.952161119
SC and lymphatic ECs	2200002D01Rik	0.396079009	0.348588929	0.038872	0.89275766
SC and lymphatic ECs	Maf	0.416913448	0.811618012	0.0039354	0.421746973
SC and lymphatic ECs	Dcn	0.423069261	0.323158263	0.0051399	0.477872926
SC and lymphatic ECs	Cd274	0.435862803	0.183408087	0.0011551	0.245704423
SC and lymphatic ECs	Irgm2	0.443506227	0.429817494	0.0010092	0.217972035
SC and lymphatic ECs	Smad7	0.452939646	0.689540231	0.0049379	0.465259973
SC and lymphatic ECs	Chd2	0.457738352	0.292135387	0.0047797	0.453397513
SC and lymphatic ECs	Riok3	0.467453026	0.536265509	0.0063592	0.527624929
SC and lymphatic ECs	Trim56	0.477021409	0.401746764	0.0032227	0.393423375
SC and lymphatic ECs	Slc7a1	0.479162863	0.288639209	0.001001	0.217972035
SC and lymphatic ECs	Hnrnph1	0.48143712	0.779042985	0.0072957	0.561297423
SC and lymphatic ECs	Ifi203	0.490048113	0.543310337	0.0270371	0.7938445
SC and lymphatic ECs	Ndufa1	0.49098682	0.597610573	0.0005597	0.165135669
SC and lymphatic ECs	Slk	0.49822332	0.76577865	0.0001362	0.068311719
SC and lymphatic ECs	Sppl2a	0.502262121	0.300330636	0.0019629	0.324193875
SC and lymphatic ECs	Pfkfb3	0.504048699	1.049837266	0.0468351	0.952161119
SC and lymphatic ECs	Slco2a1	0.511989691	0.751096674	0.0035664	0.405541196
SC and lymphatic ECs	A2m	0.51650474	0.205727114	7.26E-06	0.014563133
SC and lymphatic ECs	Ranbp2	0.517544538	0.586058646	0.0069235	0.549150943
SC and lymphatic ECs	H2-T23	0.546918027	0.846353454	0.0094307	0.617439355
SC and lymphatic ECs	Cd74	0.551390238	0.288402255	0.0232647	0.773282627
SC and lymphatic ECs	Uaca	0.553984032	0.499846708	0.0023225	0.352782284
SC and lymphatic ECs	Ifi202b	0.563098703	0.607788244	0.0036771	0.405541196
SC and lymphatic ECs	Ahnak	0.572305133	1.885705404	0.0210795	0.739084634
SC and lymphatic ECs	AA467197	0.575794542	0.523171	0.0025692	0.364333702
SC and lymphatic ECs	Hnrnpa2b1	0.59473331	1.258007804	0.0035935	0.405541196

SC and lymphatic ECs	Tnfaip2	0.607737017	0.254515843	6.37E-05	0.05587941
SC and lymphatic ECs	Gbp4	0.613945059	0.337348759	0.0077197	0.561297423
SC and lymphatic ECs	Cd200	0.616528866	1.167439927	0.0185605	0.739084634
SC and lymphatic ECs	Hsph1	0.62247615	1.211600659	0.0135702	0.684052662
SC and lymphatic ECs	Col1a2	0.622974687	0.407509228	0.0003614	0.130087169
SC and lymphatic ECs	Irgm1	0.628381964	0.8352465	0.0029808	0.380430981
SC and lymphatic ECs	Man1a	0.673562079	0.836931847	0.0001203	0.067572596
SC and lymphatic ECs	Slfn5	0.676007898	1.313541905	7.82E-05	0.063278332
SC and lymphatic ECs	Timp3	0.681902558	2.12424509	0.0142079	0.684052662
SC and lymphatic ECs	Cxcl10	0.692588133	0.615879524	0.0167375	0.723178887
SC and lymphatic ECs	Dst	0.692917252	1.02542187	0.0013396	0.257627217
SC and lymphatic ECs	Flt1	0.832224236	0.897573397	0.0001125	0.067542692
SC and lymphatic ECs	Mt1	0.86018794	0.815019282	0.0007287	0.186373698
SC and lymphatic ECs	Lars2	0.867600743	0.747414141	0.0001764	0.072819703
SC and lymphatic ECs	Col1a1	0.879672915	0.653977394	2.47E-05	0.038566575
SC and lymphatic ECs	Clic4	0.901414401	1.566284363	2.03E-07	0.000951122
SC and lymphatic ECs	Rnf213	0.928548375	0.908842703	9.81E-05	0.067542692
